# A comprehensive review on the capability of graphene quantum dots-based/involved platforms for the detection of inorganic ions

**DOI:** 10.1039/d5ra04935k

**Published:** 2025-11-24

**Authors:** Prashant Dubey

**Affiliations:** a Centre of Material Sciences, Institute of Interdisciplinary Studies (IIDS), University of Allahabad Prayagraj 211002 Uttar Pradesh India pdubey@allduniv.ac.in pdubey.au@gmail.com

## Abstract

Substantial contamination in the ecosystem (particularly, in waterbodies) due to the disposal of hazardous substances from superfluous industrial byproducts and other human activities is one of the most serious environmental issues. Although water is among the five basic elements required for all the living beings, it is continuously becoming unsafe and unhygienic for the purpose of drinking and household activities. The presence of heavy metal ions and many toxic anions (beyond their permissible concentration) significantly contributes to water pollution. Therefore, it is judicious to detect inorganic ions in order to avoid adverse situations related to the human health and ecological imbalance. Owing to the easy affordability and unique properties of zero-dimensional graphene quantum dots (GQDs), particularly, high/tunable fluorescence, electronic conductivity, electrocatalytic activity, chemiluminescence (CL) and electrochemiluminescence (ECL), GQDs-based/involved platforms are potentially deployed for the detection of inorganic ions with reasonable selectivity and sensitivity. The versatility of these sensing probes includes the possibility of detection through fluorimetric, colorimetric, electrochemical, CL, and ECL techniques. This review aims to comprehensively inspect the deployment of GQDs-based/involved sensors for the recognition of inorganic ions, highlighting different sensing approaches with the development of performance metrics and providing insights into their underlying mechanisms. Furthermore, this collective outlook on GQDs-based/involved sensors for inorganic ions may help to identify shortcomings in the existing knowledge and influence/inspire new research with better prospects.

## Abstract 2

Easily synthesizable GQDs/modified-GQDs or their integrated versions with other components can effectively probe various metal ions and anions *via* fluorimetric, colorimetric, electrochemical, chemiluminescence, and electrochemiluminescence methods.

## Graphical abstract

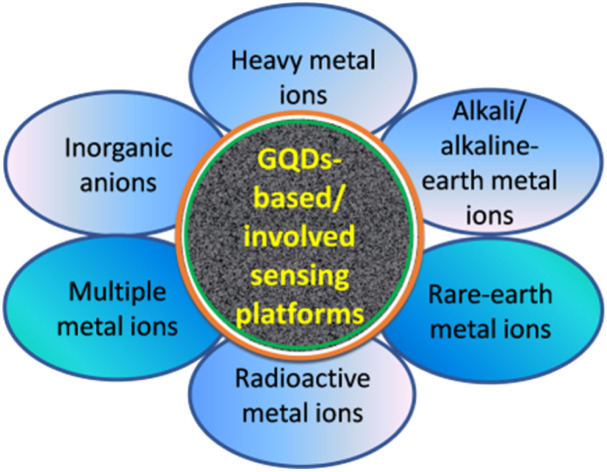

## 1. Introduction

Industrialization has helped to meet the demands of modern civilization; however, it has contributed to the release of a plethora of toxic chemicals and byproducts into the environment, especially in aquatic ecosystems and soil. Heavy metals/metal ions (HMs/HMIs) are some of the toxic substances, which are serious environmental pollutants that cause health hazards to living beings.^1–4^ The chemical production industry, mining & metallurgical industry, and electroplating engineering have significantly contributed to the discharge of various HMs/HMIs into the environment.^5^ These HMs/HMIs enter in the living organisms predominantly by the means of oral route, apart from skin contact and respiration.^6^ Among the metallic contaminants in the natural environment, toxic HMIs such as mercuric ion (Hg^2+^), lead ion (Pb^2+^), cadmium ion (Cd^2+^), arsenic ion (As^3+^) and chromium ion (Cr^6+^/Cr^3+^) have been regarded as the foremost detrimental substances to the living beings and human health.^1–4^ For instance, inorganic and organic mercury (Hg) compounds exhibit neuro-/geno-/immuno- and cardio-toxicity as well as cause damage to the reproductive system, promote cancer and diseases related to pregnancy, and induce damages to pulmonary and renal organs.^7^ Human exposure to lead (Pb) is responsible for damage to the kidneys, brain, reproductive organs, bones, and neurological system.^8^ The toxic effect of Cd^2+^ towards extensive damages at the tissue and cellular levels is induced by oxidative stress, calcium ion (Ca^2+^)-signal disruption, and cellular-signal interference.^9^ Chronic exposure to trivalent As^3+^ induces toxic effects in the liver, kidneys, reproductive organs, and cardiovascular system, which further manifest to cause cancer.^10^ Aside from the carcinogenic nature of Cr^6+^, it has been recognized as a budding neuro-toxicant owing to its deleterious effect on the human brain.^11^ Even though sodium ion (Na^+^), potassium ion (K^+^), Ca^2+^, magnesium ion (Mg^2+^), cupric ion (Cu^2+^), cobalt ion (Co^2+^), zinc ion (Zn^2+^), and ferrous/ferric ions (Fe^2+^/Fe^3+^)^12,13^ metallic cations and phosphate (PO_4_^3−^),^14^ pyrophosphate (P_2_O_7_^4−^),^15^ sulfide (S^2−^),^16^ nitrite (NO_2_^−^),^17^ iodide (I^−^),^18^ and thiocyanate (SCN^−^)^19^ anions are essential for biological activities, they can be a threat beyond their permissible limit. There are proper guidelines set by the World Health Organization (WHO) and Environmental Protection Agency (EPA) for the permissible limits of many of the inorganic contaminants in drinking water (Table 1).^20,21^

**Table 1:** Various HMs and inorganic anions with their permissible limits according to the WHO and EPA^20,21^

HMs/inorganic anions	Permissible limit in µg L^−1^	
	WHO	EPA
Ag	100	—
Al	900	—
As	10	10
Cd	3	5
Cr (total)	50	100
Cu	2000	1300
CN^−^	2000	200
F^−^	1500	4000
Hg	6	2
Ni	70	—
NO_2_^−^	3000	1000
Pb	10	10
U	30	30

Therefore, the assurance of the level of these contaminants in water by proactive and accurate monitoring is crucial to avoid escalating situations and further health risks. Various spectroscopic techniques such as atomic absorption spectroscopy (AAS),^22,23^ inductively coupled plasma-optical emission spectroscopy (ICP-OES),^24,25^ inductively coupled plasma-mass spectroscopy (ICP-MS),^26,27^ X-ray fluorescence spectroscopy,^28,29^ and surface-enhanced Raman scattering spectroscopy^30,31^ have been utilized for the reliable and efficient detection of inorganic ions, particularly HMs. However, these analytical methods are often limited due to the requirement of expensive instrumentation, lengthy processes, complicated sampling protocols, and trained personnel. In contrast, fluorimetric (FL), colorimetric (COL), electrochemical (EC), chemiluminescence (CL), and electrochemiluminescence (ECL) techniques offer the promising and reliable detection of inorganic ions due to their simple operation, short time, low cost, user-friendliness, good precision, and *in situ* analytical capability. However, although semiconductor quantum dots,^32,33^ metallic nanoparticles (NPs),^34–36^ metal oxides,^37–39^ and organic molecules^40–42^ have been employed as functional materials to sense inorganic ions, they possess inherent toxicity, structural instability, and environmental concerns. Hence, the development of sensing platforms with environmentally benign, cost-effective, non-toxic, and easily accessible functional materials is of extreme significance and has become a major research trend in the past few years.

Graphene quantum dots (GQDs) can be referred to as highly crystalline zero-dimensional carbon nanostructures with predominant sp^2^ hybridized carbon frameworks consisting of one or few graphene layers, graphitic in-plane lattice spacing of 0.18 to 0.24 nm, graphitic interlayer spacing of 0.334 nm, and lateral sizes commonly below 10 nm.^43^ Since GQDs were first fabricated in 2008,^44^ different top-down and bottom-up synthetic pathways have been actively explored for the easy and cost-effective production of GQDs (discussed in Section 2). The obtained GQDs usually contain various functional groups at their edges or on the defect sites of their basal planes, which along with their surface/edge states and/or size-based quantum confinement effect generate a strong and stable photoluminescence (PL).^45,46^ Tailoring the physiochemical and photo-physical properties of GQDs *via* surface-functionalization, heteroatom-doping, and structural defects have shown immense possibility to tune the electronic structures, optical properties, and chemical reactivity in modified-GQDs.^47–49^ Additionally, GQDs/modified-GQDs are chemically stable,^50^ possess a high quantum yield (QY),^51^ act as electron transporters,^52^ dispersible/soluble in aqueous medium,^53^ low-toxic,^54^ biodegradable, and biologically compatible.^55^ Consequently, GQDs/modified-GQDs have been shown to be suitable for a diverse range of applications including chemo-sensing,^56^ biosensing and bioimaging,^57^ energy storage-conversion,^58^ photodynamic and photo-thermal therapies,^59^ drug delivery,^60^ electrocatalysis,^61^ and light-emitting diodes.^62^ The promise of GQDs/modified-GQDs in the selective and sensitive detection of almost all types of metal ions (MIs) and various anions *via* FL, COL, EC, CL, and ECL methods is one of the highly explored areas of research, which enables the identification of environmental contaminants in aqueous medium as well as in biological samples and living cells.

In the literature, the HMI sensing applications of GQDs-based systems have been reviewed by some researchers, whereas the recognition of inorganic anions is often neglected or briefly addressed. For instance, Zhou *et al.* (2016)^63^ reviewed the GQDs synthesized *via* various top-down and bottom-up routes for the fluorescence-based detection of inorganic ions, organic molecules, and biomolecules. The optical detection of HMIs using graphene, graphene oxide (GO), GQDs, and doped-GQDs was summarized by Zhang *et al.* (2018).^64^ Li *et al.* (2019)^65^ broadly summarized the sensing applications of GQD- and carbon quantum dot (CQD)-based nanomaterials *via* FL, CL, ECL, and EC methods with specific examples of HMIs. The optical sensing applications of GQDs-based materials for toxic HMIs were further reviewed by Anas *et al.* in 2019.^66^ Li *et al.* (2021)^48^ emphasized the fluorescence-based detection of HMIs, along with the other analytes using doped-GQDs. Revesz *et al.* (2022)^67^ highlighted GQDs-based sensors for the detection of harmful contaminants such as HMIs, along with alkali and alkaline-earth MIs and discussed the various mechanisms involved in the sensing operation. In a recent review article, Wu *et al.* (2025)^68^ emphasized the property regulation of GQDs by heteroatom-doping and surface/edge modification, and furthermore their impact on the turn-off and turn-on based fluorescence sensing of various analytes including specific examples of HMIs and anions. Another recent review by Saisree S. *et al.* (2025)^69^ was dedicated to GQDs-based materials for the EC sensing of toxic HMIs, particularly, Cd^2+^, Pb^2+^, and Hg^2+^ along with the mechanistic details in the detection process.

Notably, single-/dual-heteroatom doped-GQDs including nitrogen-doped GQDs (N-GQDs), sulfur-doped GQDs (S-GQDs), boron-doped GQDs (B-GQDs), nitrogen/sulfur-co-doped GQDs (N,S-GQDs), boron/nitrogen-co-doped GQDs (B,N-GQDs), boron/phosphorus co-doped GQDs (B,P-GQDs), and nitrogen/phosphorus-co-doped GQDs (N,P-GQDs) have been encountered for the fabrication of GQDs-based sensing systems, and furthermore their application to detect inorganic ions at various levels of selectivity/sensitivity. Additionally, the introduction of specific functional groups in the GQDs moiety has shown their relevance to selectively interact with particular inorganic ions, resulting in considerable sensitivity in the detection operation. Moreover, GQDs/modified-GQDs also serve as key components, along with the other functional segments to build GQDs involved sensory architectures for the probing of inorganic ions. Obviously, the detection of inorganic ions (particularly, toxic species in ionic form and biocompatible ions) in aqueous medium and living bodies through GQDs-based/involved simple-effective sensing probes (by utilizing various sensing methods) is an effective strategy. Owing to the continuous progress of this research field, the rationality of the current review is to provide a comprehensive, in-depth, and systematic overview of the GQDs-based/involved platforms utilized in the identification of target analytes, particularly HMIs, biologically important alkali and alkaline-earth MIs, rare-earth MIs, radioactive MIs, and inorganic anions. A brief summary at the end of the discussion for each inorganic ion will provide an understanding, comparison, and identification of suitable nanoprobe/sensing methodologies. Before approaching the sensing aptitudes of GQDs-based/involved systems, this review provides a thorough discussion of the various top-down and bottom-up routes for the synthesis of GQDs/doped-GQDs, their functionalization (covalent and non-covalent) strategies, and relevant properties such as physiochemical, optical, CL, ECL, and EC characteristics. Finally, a collective summary in the form of conclusion and challenges/future prospects of GQDs-based/involved systems is presented in terms of their synthesis, property modulation, and loopholes/improvement of their sensing metrics. We believe that this review article will provide comprehensive information about GQDs-based/involved inorganic ion sensors to identify the research progress in one platform. Moreover, a balanced discussion about the advantages/disadvantages may result in a critical judgement on the capability of GQDs-based/involved systems in the analytical detection of inorganic ions. Consequently, the discussion may be complemented with the additional knowledge and impact from new ideas among scientists engaged in the area of inorganic ion analysis in environmental bodies and living systems.

## 2. Synthesis of GQDs/doped-GQDs

Miniaturization of the large-sized graphitic carbon material through successive cutting *via* chemical/physical means (top-down) or fusion of small organic molecules through condensation/carbonization (bottom-up) may result in the production of GQDs/doped-GQDs. Although GQDs or their doped-counterparts can be effectively as well as controllably synthesized using both approaches, their physical characteristics such as crystallinity, size distribution, surface functionality, colloidal stability, presence of hetero-elements, and emission characteristics entirely depend on the starting precursors and synthetic conditions. The first approach for the fabrication of GQDs is based on the top-down strategy, in which graphene crystallites are cleaved into the desired geometry even up to 10 nm small GQDs through the oxygen plasma etching process.^44^

### 2.1. Top-down approach

A large variety of readily available bulk carbonaceous precursors such as carbon black,^70^ graphite,^71^ carbon nano-onions (CNOs),^72^ graphene sheets (GSs),^73^ fullerene,^74^ carbon nanotubes (CNTs),^75^ carbon shoot,^76^ carbon fibers,^77^ GO,^78^ pyrolyzed biomass,^79^ and coal^80^ can undergo dissociation by means of chemical, hydrothermal (HT), solvothermal (ST), microwave (MW), electrolysis, ultrasonication or physical treatment to produce GQDs/doped-GQDs. Oxidative cutting of the bulk precursors using strong acid oxidants such as H_2_SO_4_/HNO_3_,^71,72,77^ HNO_3_,^81,82^ H_3_PO_4_,^73^ and HClO_4_ (ref. 83) at elevated temperature is the mainstream top-down condition for the synthesis of GQDs, affording various functional groups (–OH, –COOH, and epoxides). For example, oxidative cutting of carbon fiber in concentrated H_2_SO_4_/HNO_3_ (3 : 1 volume ratio) under ultrasonication (1 h), followed by heating (85 °C, 24 h) produced GQDs with an average size of 2.45 nm.^77^ Oxidative cleavage of brewery spent grain (BSG)-derived reduced GO (synthesized *via* the calcination of BSG and ferrocene at 300 °C, 45 min) in the presence of H_2_SO_4_/KMnO_4_ followed by ultrasonication (100 W, 1 h) resulted in GQDs with a size distribution of 10–35 nm.^79^ However, although the oxidative treatment route is widely implemented in the synthesis of GQDs, it is difficult to upscale and is also environmental unfriendly due to the production of harmful gases and inorganic salts during the whole process, along with residual corrosive acid and/or other by-products.

Therefore, the acid-free oxidative cleavage of carbonaceous precursors with Fenton reagent (H_2_O_2_/Fe^3+^),^84^ H_2_O_2_,^80^ alkaline H_2_O_2_,^85^ oxone,^74^ KMnO_4_,^86^ KO_2_,^87^ NaClO,^88^*etc.* has been successfully applied to synthesize GQDs/doped-GQDs. For example, Lyu *et al.*^84^ reported the gram-scale synthesis of oxidized-GQDs (60% product yield) by treating GO powder with H_2_O_2_/FeCl_3_ in an autoclave at 180 °C (8 h). The dissociation of H_2_O_2_ (induced by Fe^3+^/Fe^2+^ catalysis) produced hydroxyl (˙OH) radicals, which attacked the defective carbons of GO to break into well-crystalline GQDs (average size: ∼3.7 nm). Pre-treated coal (600 °C, 1 h, argon (Ar)) was refluxed with H_2_O_2_ (80 °C, 10 h) followed by HT treatment in the presence of HF (120 °C, 12 h) to obtain dual-passivated fluorine (F)/nitrogen co-doped GQDs.^80^ Oxone-assisted opening of C_60_ molecules under ST conditions enabled the acid-free synthesis of blue-emitting GQDs (QY: 23.5%), which were further modified with 2,3-diaminonaphthalene (DAN), resulting in the formation of orange-emissive DAN–GQDs with a QY as high as 52.4%.^74^ The one-step acid-free oxidative cutting of GO sheets with KMnO_4_ under ultrasonication and MW (400 W, 90 °C, 30 min) irradiation afforded GQDs (average size/QY: 2 nm/23.8%) with a product yield up to 81%.^86^ Besides oxidative cutting, the amine,^89^ amine-hydrazine,^90^ hydrazine,^91^ NH_3_,^76^ dimethylformamide (DMF),^92^*N*-methyl-2-pyrrolidone (NMP),^93^*etc.* driven reduction/reductive cutting of oxidized-carbon/bulk carbon also yielded doped- or undoped-GQDs. For example, ST treatment of graphite in NMP solvent (300 °C, 24 h) facilitated simultaneous exfoliation and scission operation, resulting in 1–2 layered N-GQDs.^93^ Alternatively, an HT-treated GO dispersion (180 °C, 24 h) was simply tip-sonicated (100 W, 1 h), resulting in GQDs with an average size as small as ∼1.53 nm. Notably, the average size of GQDs was significantly reduced after tip sonication (average size before sonication was ∼15.7 nm). Moreover, the property of GQDs was further engineered with the inclusion of extra defects by Ar-plasma treatment.^78^

EC synthesis of GQDs is another top-down approach that holds a promise to control the degree of oxidation/cleavage of the carbon precursor by applying an electric potential under ambient conditions both in non-aqueous^94,95^ or aqueous^96,97^ electrolytes without involving toxic oxidizing/reducing agents. In this method, the bulk precursor is applied either as the working electrode or dispersed in a solvent. For example, the EC exfoliation of carbon fibers (anode) in an ionic liquid (IL, 1-butyl-3-methylimidazolium tetrafluoroborate, BMIMBF_4_) electrolyte resulted in the formation of blue-emitting GQDs. Electrical stress under a high applied voltage (6 V) favoured the intercalation of BF_4_^−^ within the layers/edge sites of the carbon fibers to induce corrosion, and eventually the formation of IL-functionalized GQDs. Moreover, by adding 15/30% H_2_O in IL electrolyte, the obtained GQDs showed green-/yellow-emission.^94^ Qiang *et al.*^97^ demonstrated a facile electrochemical trimming to fragment a large GO nanosheet dispersion into graphene nanoribbons, graphene nanosheets (GNSs), and GQDs just by tuning the reaction time to 2, 3, and 5 h, respectively. Here, ˙OH and oxygen (˙O_2_) radicals from the high voltage electrolysis of water (30 V) get intercalated-adsorbed on the fragile portions of GO sheets, accompanied by the disintegration of sheets into smaller fragments.

The application of laser or pulsed laser has also demonstrated to etch bigger-size carbon materials into small size GQDs within a short duration at room temperature.^98,99^ This method follows a one-step environmentally benign process by avoiding harmful chemicals and tedious post-purification protocols. Kang *et al.*^99^ applied pulsed laser exposure (Q-switch Nd:YAG, *λ* = 355 nm, 30 min, room temperature, air) to graphite flakes (dispersed in ethanol/diethylenetriamine (DETA)), which fragmented into N-GQDs. Interestingly, the N-GQDs synthesized without sonication had a much better QY (9.1%) in comparison to sonication-assisted laser irradiation (QY: 4.2%), which is attributed to the effective incorporation of nitrogen element. Plasma-plume induced by the laser irradiation of cavitation bubbles thermally decomposed the starting materials (in the form of carbon clusters/nitrogen molecules), which evaporated-condensed to produce N-doped GNS aggregates, and further fragmentation by pulsed laser into small N-GQDs.

Purely mechanical tailoring of bulk pristine materials into GQDs is also a neat and clean synthetic approach.^100–102^ For example, 44.6 wt% product yield of GQDs from multi-walled CNTs (MWCNTs) was achieved by combining silica-assisted ball milling and sonication-based exfoliation, centrifugation, and filtration, which is inspiring.^101^

### 2.2. Bottom-up approach

Synthesis of GQDs/doped-GQDs *via* the bottom-up approach involves pyrolysis,^103^ HT/ST treatment,^104,105^ MW-assisted carbonization,^106^ MW-assisted HT (MW-HT) treatment,^107^ electron-beam irradiation,^108^ ultraviolet (UV) irradiation,^109^ solution-phase condensation,^110^ or direct current (DC) microplasma treatment^111^ of small precursor molecules. Among them, pyrolysis is one of the straightforward synthetic strategies, in which citric acid (CA),^112^ trisodium citrate (TSC),^113^ glucose,^114^l-glutamic acid (GA),^115^ GA/aspartic acid,^116^*etc.* get thermally decomposed-carbonized at high temperature (above their melting point), resulting in the formation of GQDs or doped-GQDs. For example, solid CA was heated at 200 °C for 30 min under stirring to transform into an orange liquid, which was subsequently dissolved in NaOH solution, followed by pH adjustment to 8.0 and centrifugation to isolate GQDs with an average diameter of ∼2.2 nm.^112^ Doped-GQDs were also synthesized through the pyrolysis method by adding other compounds such as urea,^117^ urea-ammonia sulphate,^118^ glutathione (GSH),^119^ and thiourea (TU)^120^ in CA. For example, N-GQDs and N,S-GQDs were synthesized *via* the infrared (IR)-assisted pyrolysis of CA-urea and CA-urea-ammonia sulphate, respectively, at 260 °C for 10 min. Here, induction-based transfer of heat energy to the precursor *via* electromagnetic radiation is beneficial to achieve a homogeneous and rapid heating process at a ramping rate of 30 °C min^−1^.^118^

The HT and ST synthesis of GQDs or their doped-counterparts using appropriate starting molecular precursors in water and organic solvents, respectively, and subsequent heating under inherent vapour pressure are other facile synthetic approaches, which have been widely employed in the literature. For instance, xylan dissolved in an aqueous solution of NaOH/urea was treated under HT condition (240 °C, 24 h) to obtain single-layered (sl-) N-GQDs (sl-N-GQDs) with an average size/QY of 3.2 nm/23.8%. Attachment of NaOH hydrates to the xylan chain *via* hydrogen-bonding followed by wrapping with urea hydrates led to the growth of a channel inclusion compound. Furthermore, hydrolysis-carbonization of xylan, exfoliation with the assistance of NH_3_ and CO_2_ (generated from the decomposition of urea), and incorporation of nitrogen-containing radicals during the HT process eventually generated sl-N-GQDs.^121^ The one-step ST treatment of gallic acid in absolute ethanol at 160 °C (6 h) produced green-fluorescent GQDs with a mean diameter of 10.1 nm.^122^

The MW-enabled carbonization of organic precursor/biomass extract provides a straightforward, quick, and homogeneous heating process to achieve GQDs/doped-GQDs in a few minutes. Hsieh *et al.*^123^ developed a solid-phase MW-assisted synthetic route (2.45 GHz, 720 W, ≤180 °C, 15 min) to synthesize N-GQDs and B,N-GQDs with a product yield up to 45.1 wt% using CA as the carbon source and urea/boric acid (H_3_BO_3_) as the nitrogen/boron source. The *in situ* nitrogen-doping is distributed in the form of pyrrolic/pyridinic/graphitic nitrogen and amide functional groups, while B_4_C/BCO_2_ bonding types (within GQDs structure or decorated on the skeleton) are assigned to the boron-configuration. An aqueous solution of *Mangifera indica* leaf extract was heated in an MW oven (10 min) to yield red-emissive GQDs under UV irradiation with a QY of 45%.^124^

The bottom-up approach *via* the MW-HT technique by combining MW and HT features is advantageous for the rapid, energy saving, uniform, and efficient preparation of GQDs. Contrary to MW synthesis, where the precursor is irradiated by MW under atmospheric pressure, the MW-HT method relies on the MW-based heating of the starting material in a MW-transparent sealed vessel. As a result, a high temperature is achieved in a short period of time due to the creation of a pressurized environment.^125^ For example, dielectric heating of a 1,3,6-trinitropyrene (TNP)/0.3 M NaOH solution in a confined glass vessel for 3 min under MW irradiation (reaction temperature: 200 °C) resulted in bright yellow-luminescent GQDs (under UV light).^107^

The microplasma technique has been effectively used under ambient conditions without involving harsh chemicals or reaction environment to synthesize GQDs/doped-GQDs. One-dimensional gaseous discharge within the small depth of the plasma–liquid interface can produce reactive species (radicals, ions, electrons, *etc.*) with a high energy density for the nucleation and growth of GQDs from the starting precursor.^111^ The plasma electrochemical synthesis of N-GQDs was demonstrated under ambient conditions using chitosan as the sole precursor. The reaction was performed under DC discharge flowing Ar (discharge current: 5 mA) for 1 h to accumulate N-GQDs (average size: 3.9 nm) at few mm below the plasma–liquid interface. Based on the experimental observations, it was deduced that plasma-generated ˙OH initially cleaves the glycosidic bonds of the long-chain chitosan to generate aldehyde- and carboxylic-containing species, which subsequently reassembled into an aromatic structure to grow N-GQDs with the involvement of solvated electrons (generated by plasma).^126^

Solution-phase chemistry can allow step-wise chemical reactions for the synthesis of GQDs from small organic molecules. For instance, d-glucose is catalytically (acetic acid as the catalyst) transformed into the Amadori product in the presence of hexadecylamine (HDA), followed by spontaneous dehydrolysis in the solution-phase, resulting in the formation of single-crystalline hexagonal-shaped GQDs with low oxygen defects.^110^ Ochi *et al.*^127^ demonstrated the mass-scale synthesis of blue-green fluorescent GQDs (average size: ∼1.4 nm) in an open atmosphere by air-flow reflux heating of phloroglucinol-Na_3_PO_4_·12H_2_O in 1,2-pentanediol (180 °C, 6 h), followed by dialysis and silica-gel chromatographic purification. Here, Na_3_PO_4_·12H_2_O acted as the base catalyst to promote the dehydration–condensation reaction during the synthesis process, resulting in an exceptionally high product yield of 99.4%. Moreover, after silica-gel purification, the QY of GQDs (in ethanol) increased from 54% to 75%. Experimental results revealed that the attachment of 1,2-pentanediol at the edges of GQDs effectively suppressed their aggregation and concentration-induced quenching, and therefore a high QY was achieved.

## 3. Functionalization of GQDs/doped-GQDs

Various functional moieties can be introduced in GQDs during their synthesis process. For example, HT treatment of CA and polyethyleneimine (PEI) resulted in PEI-functionalized N-GQDs.^128^ When CA is HT-treated in the presence of taurine, sulfonic acid group-functionalized GQDs are obtained with high water solubility (3.6 mg mL^−1^).^129^ Furthermore, GQDs/doped-GQDs synthesized *via* the top-down or bottom up approach inherently contain aromatic domains and a range of oxygen-containing functional groups on their surface/edges, which open the possibility to carry out post-modification through various covalent and non-covalent chemistries. The condensation reaction between –COOH and –NH_2_ groups in the presence of 1-ethyl-3-(3-dimethylaminopropyl)-carbodiimide (EDC)/N-hydroxysuccinimide (NHS) system or only with EDC through carbodiimide coupling chemistry can produce an amide linkage between two building blocks and has been extensively utilized in the covalent-functionalization of GQDs. For instance, GQDs synthesized from graphite flakes were coupled with 2,6-diaminopyridine (DAP) *via* EDC/NHS-based coupling reaction, which showed bluish-green emission and a higher QY (13.4%) in comparison to bare GQDs (4.7%).^130^ Recently, GQDs were activated with EDC/*N*-hydroxysulfosuccinimide sodium salt (sulfo-NHS) and coupled with dopamine (DA) to produce DA-functionalized GQDs (DA–GQDs).^131^ Amidation reaction can also be conducted under basic conditions without the involvement of EDC/NHS to functionalize GQDs. For example, the formation of imine bonds after the reaction between –NH_2_ groups of N-GQDs and –COOH groups of pamoic acid (PA) under alkaline conditions confirmed the covalent attachment of PA to the surface of N-GQDs.^132^

The acid chloride formation route relies on the transformation of –COOH groups into highly reactive acid chloride, which can react with amine/alcohol group-containing moieties to generate amide/ester bonding. The activation of the –COOH groups present on the GQDs in the form of acyl chloride, followed by *N*-(rhodamine B) lactam-ethylenediamine (RBD) substitution through the amide linkage in the presence of triethylamine (TEA) resulted in the formation of RBD–GQDs.^133^ Conversely, hydroxyl-functionalized 3,4-ethylenedioxythiophene (EDOT) was condensed with acid chlorides at the edge of GQDs (generated by the transformation of –COOH with oxalyl chloride) *via* ester linkage in the presence of TEA/4-dimethylaminopyridine (DMAP), resulting in EDOT–GQDs.^134^

GQDs can also be functionalized through esterification reaction between the –COOH and –OH groups present on the starting reactants. For instance, the *N*,*N*′-dicyclohexylcarbodiimide (DCC)/DMAP-induced coupling reaction between the –OH groups of GQDs and –COOH groups of the reversible addition–fragmentation chain transfer agent (RAFT) resulted in RAFT–GQDs, which were further integrated with a block copolymer (BCP) to produce multicolor emitting BCP–GQDs.^135^ Conversely, the –COOH groups of GQDs are activated with DCC/DMAP, followed by esterification reaction with the –OH moiety of dimercaprol (DMC) to synthesize DMC–GQDs.^103^ Consecutive Steglich-esterification followed by reductive-esterification condition occurred between pristine GQDs and the 4,4′-(1,2-diphenylethene-1,2-diyl)diphenol (TPE-DOH) rotor molecule to synthesize edge-functionalized TPE–GQDs. The presence of ester (–C(=O)OC–) and ether (–C–O–C–) groups in TPE–GQDs indicated the successful substitution of –COOH/C=O groups at the edge of GQDs *via* esterification/reductive–esterification reaction. Moreover, the existence of four phenyl groups in TPE–GQDs effectively maximize the steric hindrance to inhibit aggregation-induced quenching (AIQ) and result in aggregation-induced emission (AIE) characteristics with a QY as high as 16.8% in the solid state.^136^

Some other covalent-functionalization strategies for the modification of GQDs are as follows: polyvinyl alcohol (PVA) is grafted on the GQDs surface *via* Friedel–Crafts alkylation reaction.^137^ Propargyl bromide is reacted with the –OH groups of GQDs to introduce C≡C triple bonds at the periphery of GQDs, which subsequently cross-linked with azide-functionalized poly(ethylene oxide) *via* Cu^+^-catalyzed click chemistry to result in poly(ethylene glycol) (PEG)-functionalized GQDs (PEG–GQDs).^138^ Recently, the click reaction between the thiol (–SH) groups of cysteine (Cys) and C=C double bonds of GQDs in the presence of azobisisobutyronitrile initiator afforded Cys–GQDs.^139^

Secondary weak interactions such as π–π, ionic, hydrogen-bonding, and van der Waals interactions may offer a simple and rapid protocol for the non-covalent-functionalization of GQDs. Due to the inherent aromatic nature of GQDs, they can conjugate with suitable counterparts *via* π–π stacking. For instance, π–π stacking between GQDs and peptide (PEP)-functionalized AuNPs resulted in the formation of an AuNPs–PEP@GQDs nanoconjugate.^140^ π–π interaction between OH-GQDs and PPy-Br dye resulted in the formation of a ratiometric conjugate for sensing application.^141^ The negative surface charge of GQDs arising from –COOH/–OH groups can allow them to interact with positively charged moieties through ionic or electrostatic interaction. For example, negatively charged GQDs are passivated with xylan and chitosan oligosaccharide *via* electrostatic interactions for sensing and bioimaging applications.^142^

Various functional groups on GQDs can also assist hydrogen-bonding interaction during post-modification or incorporation in other matrices. For instance, the strong hydrogen-bonding ability of GQDs with the –OH groups of cellulose resulted in the formation of a stable GQDs/cellulose membrane with open structure and high water permeability.^143^ Electro-polymerization of aniline in the presence of N-GQDs generated an N-GQDs/polyaniline (N-GQDs/PANI) nanocomposite for the non-invasive detection of glucose, where N-GQDs are electrostatically (preferably hydrogen-bonding) bonded with polymer chains.^144^

van der Waals interaction-based non-covalent functionalization of GQDs is less common in the literature. A short range van der Waals interaction between amine-functionalized GQDs (Am–GQDs) and few-layer MoS_2_ sheets by the simple mixing of two components may be a representative example, where Am-GQD/MoS_2_ heterostructures were probed for Foster-type energy transfer from Am–GQDs to MoS_2_ layers, and consequently the quenching of the fluorescence of Am–GQDs. Shifting of the Fermi level of Am–GQDs towards the conduction band in the van der Waals stacked heterostructures further validated the charge transfer-based quenching mechanism.^145^

## 4. Properties of GQDs/modified-GQDs

### 4.1. Physiochemical characteristics

**Fig. 1:**
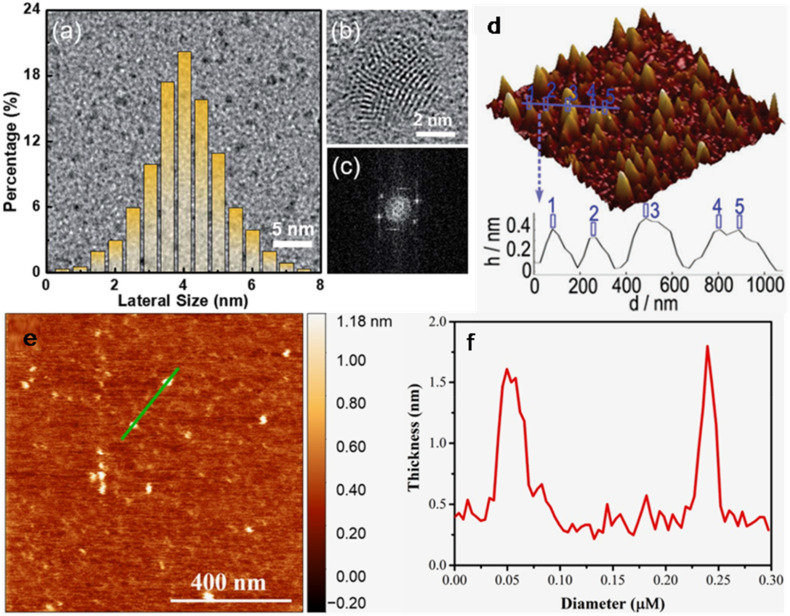
TEM image with size distribution bar-diagram (a), HRTEM image (b), and FFT pattern (c) of N-GQDs. Reproduced/adapted from ref. 147 with permission from The Royal Society of Chemistry, 2019. (d) 3D AFM image and the corresponding height pattern of B,N-GQDs. Reproduced/adapted from ref. 148 with permission from The Royal Society of Chemistry, 2017. Topographical AFM image (e) and corresponding thickness–diameter graph (f) of N-GQDs. Reprinted from ref. 149, copyright 2025, with permission from Elsevier.

GQDs are basically anisotropic sub-domains of graphene with lateral dimensions below 10 nm and thickness of a few nanometres. The high level of crystallinity in GQDs make them different from CQDs due to the presence of predominant sp^2^ carbon domains composed of one or few layers of graphene, while CQDs are less crystalline quasi-spherical particles with a mixture of sp^2^ and sp^3^ hybridized carbon.^146^ Moreover, the structure and chemical features of GQDs can be modified by heteroatom-doping and surface-functionalization.^146^ Transmission electron microscopy (TEM) and high-resolution TEM (HRTEM) are routinely applied to understand the morphology, size distribution, and structural features of GQDs/modified-GQDs. For example, the lateral size/crystallinity of N-GQDs (synthesized from the single precursor *ortho*-phenylenediamine (*o*-PDA), QY: 80%) measured from TEM/HRTEM images (Fig. 1a/1b) depicted the size distribution (average size)/lattice spacing of 1–8 nm (3.8 nm)/0.24 nm (corresponding to (110) diffraction plane), respectively. Moreover, fast Fourier transform (FFT) analysis further confirmed the six-fold symmetry and good crystallinity of the synthesized N-GQDs (Fig. 1c).^147^ Besides, atomic force microscopy (AFM) clearly indicates the number of layers in the GQDs according to the height analysis. The three-dimensional (3D) AFM image and corresponding topographic heights of B,N-GQDs showed nearly uniform heights of ∼0.5 nm (Fig. 1d), indicating the single-layer characteristic of B,N-GQDs.^148^ Alternatively, the thickness of 1–1.5 nm measured from the AFM topographic image of N-GQDs illustrates 3–5 layers of graphene (Fig. 1e and f).^149^

**Fig. 2:**
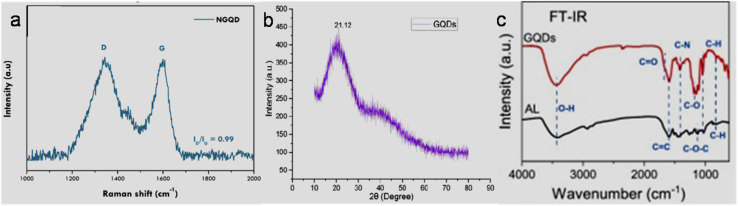
(a) Raman spectrum of N-GQDs showing D and G bands and the *I*_D_/*I*_G_ intensity ratio. Reprinted (adapted) with permission from ref. 150, copyright 2024, the American Chemical Society. (b) PXRD profile of GQDs showing a prominent peak at a 2*θ* value of 21.1°. Reproduced/adapted from ref. 151 with permission from The Royal Society of Chemistry, 2025. (c) FTIR spectra of AL and GQDs synthesized from AL. Reprinted from ref. 152, copyright 2021, with permission from Elsevier.

The crystallinity and disordered characteristics of GQDs/modified-GQDs can also be judged by Raman spectroscopy. For instance, N-GQDs synthesized from a CA/NH_4_OH mixture exhibited the typical Raman peaks of a carbon material at ∼1340 and ∼1600 cm^−1^, corresponding to the D and G bands, respectively (Fig. 2a). Moreover, the large *I*_D_/*I*_G_ peak intensity ratio of 0.99 (Fig. 2a) indicated the presence of significantly high defect levels in the N-GQDs due to the incorporation of nitrogen atoms in their lattice.^150^ The powder X-ray diffraction (PXRD) pattern of GQDs/modified-GQDs generally shows a peak in the 2*θ* range of 20–25° due to the presence of a graphitic structure. For example, MW-synthesized GQDs using *Azadirachta indica* (neem) leaf extract exhibited slightly broad peak at the 2*θ* value of ∼21.12° (Fig. 2b), indicating the graphitic structure of GQDs with a small content of amorphous carbon.^151^ Fourier transform IR (FTIR) and X-ray photoelectron spectroscopy (XPS) are effective tools for the identification of various functional groups and elemental compositions present in GQDs/modified-GQDs. For instance, the FTIR spectra of alkali lignin (AL) and GQDs derived from AL are shown in Fig. 2c. The presence of peaks at 3443/1660/1415 cm^−1^ indicated –OH/–COOH/C–N-enriched GQDs with the successful doping of nitrogen element. The peaks at 1590 (C=C vibration) and 1049/827 cm^−1^ (C–H vibration) are due to the aromatic domains of GQDs. Moreover, the insignificant peak at 1190 cm^−1^ (due to C–O–C stretching vibration) in GQDs compared to AL suggested the breaking of the ether bond and formation of oxygen functionalities during the synthesis process.^152^

**Fig. 3:**
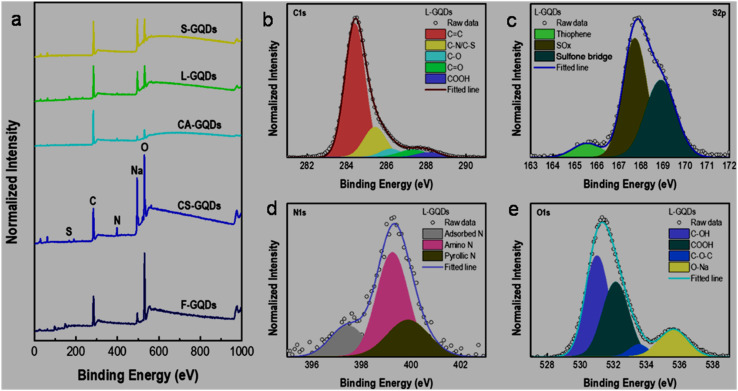
(a) XPS survey scans of five bioresource-synthesized GQDs. High-resolution XPS patterns of lignin-derived L-GQDs corresponding to (b) C 1s, (c) S 2p, (d) N 1s, and (e) O 1s elements. Reprinted (adapted) with permission from ref. 111, copyright 2022, the American Chemical Society.

The full scan XPS spectra of five types of bioresource-derived GQDs indicated the presence of C (283.9 eV), O (530 eV), N (398 eV), and S (167.9 eV) elements, along with the Na element (adsorbed/bonded with GQDs during synthesis because of the involvement of NaOH electrolyte, Fig. 3a). Moreover, the high-resolution XPS results of lignin-derived L-GQDs further confirmed the existence of various bonding features corresponding to C 1s (C=C: 284.4 eV, C–N/C–S: 285.4 eV, C–O: 286.2 eV, C=O: 287.3 eV, and COOH: 288.1 eV; Fig. 3b), S 2p (thiophene: 165.5 eV, SO_*x*_: 167.7 eV, and sulfone bridge: 168.9 eV; Fig. 3c), N 1s (adsorbed N: 397.4 eV, amino nitrogen: 399.3 eV, and pyrrolic nitrogen: 399.9 eV; Fig. 3d), and O 1s (C–OH: 530.9 eV, –COOH: 532.1 eV, C–O–C: 533.5 eV, and O–Na: 535.6 eV; Fig. 3e), complementing the successful synthesis of N,S-GQDs with 74.2/21.2/1.9/2.8 at% of carbon/oxygen/nitrogen/sulfur elements, respectively.^111^

### 4.2. Optical characteristics

**Fig. 4:**
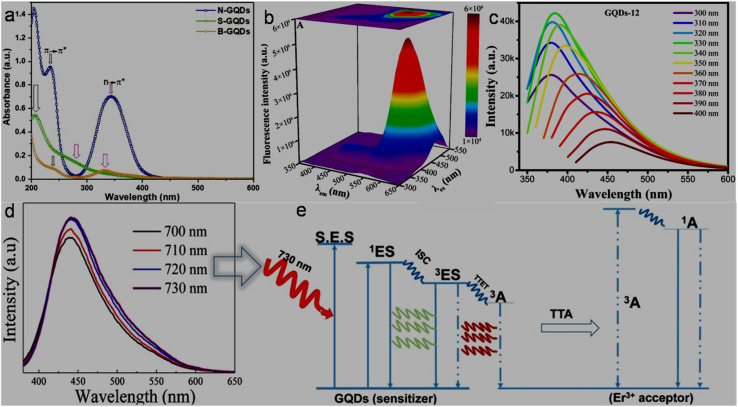
(a) UV-visible absorption spectra of N-GQDs, S-GQDs, and B-GQDs. Reprinted from ref. 153, copyright 2024, with permission from Elsevier. (b) Fluorescence spectra of FA,His,Ser–B,P-GQDs according to different *λ*_ex_ and their 3D mapping. Reproduced/adapted from ref. 166 with permission from The Royal Society of Chemistry, 2024. (c) Fluorescence spectra of GQDs at *λ*_ex_ values from 300 to 400 nm, showing their EDPL characteristic. Reprinted from ref. 167, copyright 2023, with permission from Elsevier. UCPL spectra within the *λ*_ex_ value of 700–730 nm (d) and possible mechanism (e) of Er-GQDs. Reprinted from ref. 171, copyright 2020, with permission from Elsevier.

The UV-visible absorption spectrum of GQDs/modified GQDs generally features strong absorption below 300 nm (UV region) due to the π–π* electronic transitions from their conjugated domain and extended tails towards the visible/near IR (NIR) region (sometimes with shoulder peaks) due to the n–π* transitions from their surface functional groups.^120,121,153^ For instance, the absorption spectra of N-GQDs, S-GQDs, and B-GQDs exhibited characteristic absorption peaks corresponding to the π–π* and n–π* transitions at different positions (Fig. 4a). The peak corresponding to π–π* transition is more prominent in N-GQDs (234 nm) in comparison to S-GQDs (206 nm) and B-GQDs (240 nm), suggesting a relatively high amount of sp^2^ hybridized moieties (C=C backbone) in N-GQDs due to the electron-donating ability of the nitrogen element and less amount of oxygen-containing functional groups.^153^

Room temperature PL is one of the most attractive features of GQDs/modified-GQDs for sensing and other fluorescence-related applications, which usually originates from their non-zero band gap structures due to their confined size effect, chemical doping, and surface passivation/functional groups. These parameters can be effectively tuned to obtain GQDs/modified-GQDs with emission features ranging from deep UV,^154^ to blue,^155^ green,^156^ yellow,^157^ orange,^74^ red,^158^ and NIR.^159^ Apart from their pronounced quantum confinement effect, the abundant edge states and functional groups of GQDs/modified-GQDs play a vital role in dictating their PL properties. Yan *et al.*^160^ demonstrated a gradual narrowing in the band gap of GQDs and their corresponding fluorescence color (green to red) using two different functionalization strategies, as follows: (i) lowering the position of π* orbitals by enlarging the π conjugation system in GQDs through covalent-functionalization with poly-aromatic compounds and (ii) creating an n orbital between the π and π* orbitals of GQDs *via* conjugation with electron-donating functionalities. This precise band gap tailoring from 2.40 eV to 2.05/1.95/1.91/1.88 eV (approach i) and 2.40 eV to 2.08/2.02/1.94 eV (approach ii) opened the possibility to engineer GQDs with different emission-wavelength (*λ*_em_) characteristics. The degree of surface oxidation also influences the optical properties of GQDs. It was observed that by increasing the degree of oxidation in GQDs, their maximum emission peak shifted towards the higher wavelength side due to the presence of more surface defects.^161,162^ Among the three types of N-GQDs (N-GQDs-1, N-GQDs-2, and N-GQDs-3) synthesized *via* the DC microplasma method, the *λ*_em_ of N-GQDs-3 (532 nm) is significantly red-shifted with an enhanced peak intensity in comparison to N-GQDs-1 (459 nm) and N-GQDs-2 (∼468 nm), enabling emission tunable engineering by varying the heteroatom-doping configurations and surface functionalities. XPS results revealed that N-GQDs-3 possessed a larger amount of pyrrolic nitrogen along with an exclusive pyridinic nitrogen configuration, which intensified the electron density, and therefore the band gap narrowing caused a significant red-shift in their *λ*_em_. Moreover, the high pyrrolic nitrogen content in N-GQDs-3 effectively minimized the emissive traps to result a high QY of 30.1% (QYs of N-GQDs-1 and N-GQDs-2: 4.68% and 1.74%).^163^

Both excitation-independent PL (EIPL)^164–166^ and excitation-dependent PL (EDPL)^120,121,149,167^ characteristics are observed in GQDs/modified-GQDs. For example, the appearance of a single emission peak (∼550 nm) with a variation in the excitation wavelength (*λ*_ex_: 300–550 nm) from the functionalized/doped FA,His,Ser–B,P-GQDs (FA: folic acid, His: histidine, and Ser: serine) indicated EIPL behaviour (Fig. 4b) due to the optimized band structure with predominantly single fluorescence center (n–π* transition).^166^ Alternatively, the GQDs synthesized from spent tea leaves showed a gradual red shift in *λ*_em_ with *λ*_ex_ in the range of 300–400 nm (Fig. 4c), which is attributed to the different size effect and presence of oxygen-containing functional groups on the surface of GQDs.^167^ Although the exact mechanism is unclear, the EDPL with multicolor-emission features of GQDs-system is frequently explained by the quantum size effect and surface/edge states.^120,149,167^ GQDs/modified-GQDs may also exert more than one emission peaks at a single *λ*_ex_, which is advantageous for ratiometric fluorescence-based analytical applications. Experimental and theoretical results suggested that the triple emission peaks (599/640/710 nm at *λ*_ex_: 460–640 nm, EIPL behaviour) of red-fluorescent N-GQDs arise from the pyrrolic/pyridinic/amino nitrogen types of emissive states, while graphitic nitrogen in their structure is responsible for their good QY (35%).^168^

The multi-photon activation process may generate an anti-Stokes luminescence (shorter *λ*_em_ than *λ*_ex_) in the form of up-conversion PL (UCPL), which is generally governed by energy transfer, excited-state absorption, and photon avalanche mechanism.^169,170^ The UCPL spectra of erbium (Er)-doped GQDs (Er-GQDs) under *λ*_ex_ of 700–730 nm are shown in Fig. 4d, which depicted emission peaks in the range of 437–442 nm. The UCPL phenomenon is explained based on the triplet–triplet annihilation (TTA) mechanism, which involved three stages, as follows: (i) excitation of sensitizer (GQDs) to singlet excited state (^1^ES), followed by intersystem crossing (ISC) to triplet state (^3^ES), (ii) triplet-type energy transfer (TTET) from GQDs ^3^ES to annihilator/acceptor (here Er^3+^) triplet state (^3^A), and (iii) recombination of two triplet states into one as a relaxation phenomenon to the ground state and the other to the singlet excitation state of an annihilator (^1^A), which actually generates emission (Fig. 4e).^171^

A high QY (ratio of emitted photons with respect to adsorbed photons during radiation-induced process) of GQDs/modified-GQDs is a direct reflection of their high PL intensity, which is relevant to their fluorescence-based application and can be achieved by suitable elemental-doping and surface-functionalization/passivation.

#### 4.2.1. Effect of heteroatom doping

The electronegativity of nitrogen/boron (3.04/2.04) is quite different from carbon (2.55), which in turn effectively alters the electronic nature of doped-GQDs. Nitrogen doping in GQDs is found to be beneficial for a significant enhancement in their QY due to their chemical/electronic structure modulation. For instance, an ultrahigh QY of 99% with 98 nm Stokes shift was achieved in N-GQDs (25.91% nitrogen content, synthesized *via* thermal treatment of GO and PEI).^172^ A better QY of N-GQDs (19.1%, band gap: 0.249 eV) compared to bare GQDs (1.7%, band gap: 2.826 eV) is explained based on the smaller band gap and nitrogen atom-centered electronic transitions in the N-GQDs.^173^ The high QY of HT-synthesized N-GQDs (80.31%) is ascribed to the conversion of their non-radiative centers (–COOH groups) to radiative electron–hole recombination moieties (C–N/–C=N).^153^ The intense quantum confinement and edge effects in the crystalline B-GQDs (4.8% boron content in the form of BC_2_O and BCO_2_) caused localized electron–hole pair generation for the optimum band gaps and an impressive QY of 22.7%.^174^ The electronegativity of sulfur (2.58) is quite close to carbon (2.55). As a result, the charge-transfer in the C–S bonds is expected to be low, and therefore S-GQDs have low QYs (10.6/10.2%).^175,176^ Doping of the phosphorus element in the carbon framework may also modulate the optical property and EC activity of the doped-counterpart. Besides inducing polarization in the P–C bond, coupling between the 2p of carbon and 3p orbitals of phosphorus (in PC_3_ configuration) may promote a near-Fermi level electron density, resulting in a lower band gap and better electron transfer activity.^177^ Phosphorus-doped GQDs (P-GQDs) synthesized through the ST method (precursor: glucose and triphenylphosphine) at 180 °C, 210 °C, and 240 °C showed QYs as high as 26.2%, 37.66%, and 41.84%, respectively. XPS results revealed that P-GQDs synthesized at 240 °C contain a higher amount of phosphorus-element (4.82 at%) with predominant PC_3_-structure for effective polarization and electron redistribution in comparison to that prepared at 180 °C (4.19 at% phosphorus) and 210 °C (4.57 at% phosphorus), which are mainly composed of PO_4_ and PO_3_ bond structures.^177^

Apart from single heteroatom-doping, dual-elemental doping in the form of B,N-GQDs, boron/sulfur co-doped GQDs (B,S-GQDs), N,P-GQDs, and N,S-GQDs has also employed to improve the optical properties of GQDs. The advantage of boron/nitrogen co-doping (0.9/7.5%) in B,N-GQDs can be revealed by their high QY (75%) in comparison to N-GQDs (71%) and B-GQDs (23%).^178^ B,S-GQDs synthesized *via* the pyrolysis of CA, H_3_BO_3_, and 3-mercaptopropionic acid showed much a higher QY (19.8%) in comparison to undoped GQDs (7.5%), which is ascribed to the modulation of their electronic structure and passivation of non-radiative recombination sites.^179^ The one-pot ST treatment of resorcinol and phosphonitrilic chloride trimer yielded green-emitting N,P-GQDs (nitrogen/phosphorus content: 3.05/1.81%) with a QY as high as 58.2%.^180^ The concurrent incorporation of nitrogen and sulfur in GQDs is also beneficial for improving their optical properties. For example, HT treatment of GA in the presence of urea and 1-octanethiol afforded blue-emissive N,S-GQDs with a QY as high as 70%.^181^

The incorporation of MIs in GQDs can also expand the scope of the doping strategy through a synergistic effect to enhance their fluorescence signal and QY. For example, manganese ion (Mn^2+^)-bonded B,N-GQDs showed a much higher QY (30.52%) compared to B,N-GQDs (20.12%), which is ascribed to the confinement effect between the surface functionality and Mn^2+^ to produce a uniform shape/size.^182^ Neodymium (Nd)-doped N-GQDs (Nd,N-GQDs; ∼1 at% Nd) synthesized *via* the MW method showed NIR fluorescence and QY up to 62%.^183^ Terbium (Tb)-doping in the GQDs resulted in almost twice the emission intensity at 452 nm in comparison to the bare GQDs due to the suppression of non-radiative recombination sites, and therefore a high QY (52%).^184^ Recently, Fe^3+^-chelation and nitrogen-doping simultaneously improved the PL intensity of Fe,N-GQDs and the QY was as high as 67%.^185^

#### 4.2.2. Effect of functionalization

Non-covalently or covalently modified GQDs also showed better QY compared to bare GQDs. For example, non-covalent attachment of poly-l-lysine (PLL) on the surface of GQDs resulted in PLL@GQDs, which showed a significantly higher QY (41.33%) in comparison to bare GQDs (18.89%).^186^ An exceptionally high QY of 99.8% was achieved by the *in situ* attachment of d-penicillamine (DPA) over GQDs to minimize structural defects and enhance the quantum size effect.^187^ Benefitting from the large electron-donating circumstances by the covalent attachment of pentaethylenehexamine (PEHA) and DPA on GQDs, the resultant co-functionalized PEHA,DPA–GQDs (amine/hydroxyl/carboxyl groups rich) exhibited strong fluorescence (*λ*_em_: 450 nm) and a QY as high as 90.91%.^188^ The time-dependent density functional theory (DFT) calculation revealed that sp^3^-type functional groups (O, OH or F) on the surface of GQDs can significantly improve the PL intensity as well as QY of functionalized GQDs due to the restriction of excited carriers on the graphitic layers and enlargement of the transition dipole moment during radiative recombination.^189^

### 4.3. CL and ECL characteristics

Besides PL, CL and electro-generated CL (ECL) are other intriguing features of GQDs/modified-GQDs for analytical purpose. CL emissions originate when a substance absorbs chemical energy (produced during chemical reaction) to reach the excited state, and then returns to the ground state. A variety of chemical initiators such as H_2_O_2_,^190^ permanganate-sulfite,^191^ and cerium ions (Ce^4+^),^192^ along with GQDs/modified-GQDs can produce strong CL signals. Three types of GQDs (bare GQDs, N-GQDs, and N,S-GQDs) utilized the exothermic energy released by the chemical reaction between bis(2,4,5-trichloro-6-carbopentoxyphenyl) oxalate and H_2_O_2_ in the presence of a base catalyst (sodium salicylate) to produce yellowish-white, green, and blue CL emissions. Among them, N,S-GQDs showed 5/2.5-fold higher CL efficiency than bare GQDs/N-GQDs, demonstrating that dual-doping is favorable to create efficient intrinsic-emissive surface states for substantial CL signal.^193^

**Fig. 5:**
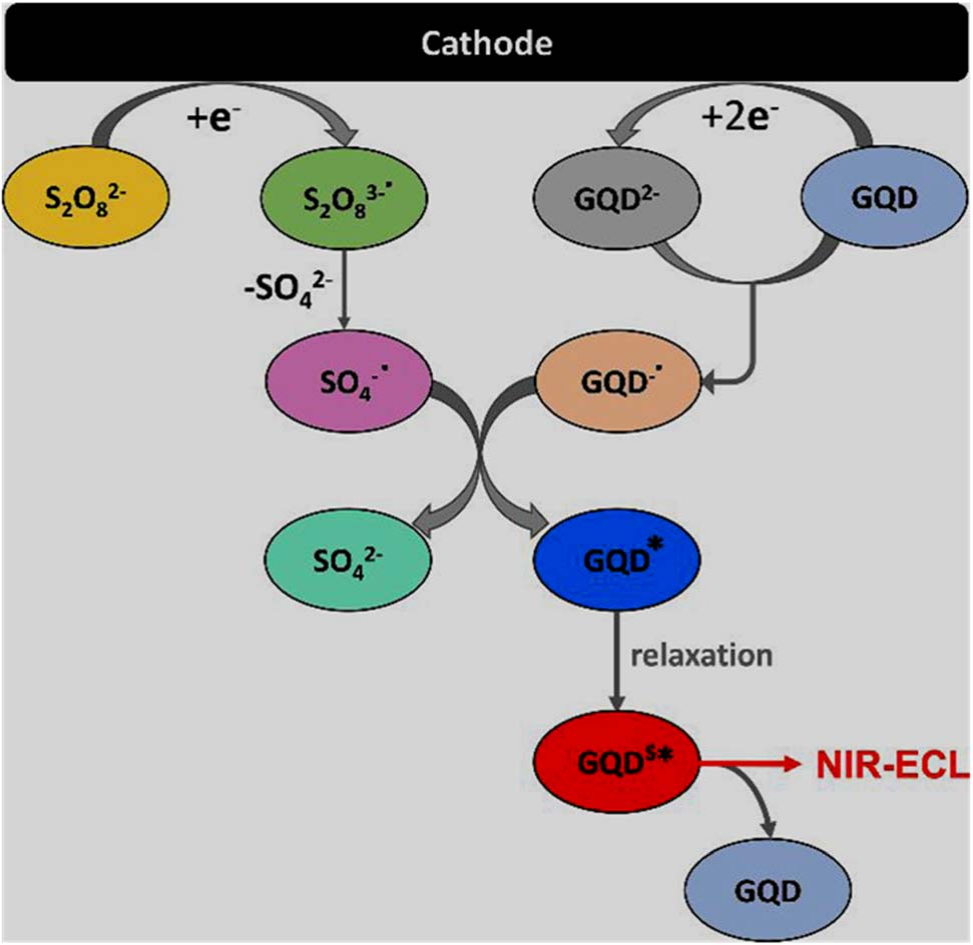
Schematic of the possible mechanism for the generation of NIR-ECL from the GQDs/K_2_S_2_O_8_ system. Reprinted (adapted) with permission from ref. 105, copyright 2021, the American Chemical Society.

ECL is a light-emitting phenomenon due to the electron transfer reaction of electrochemically generated radical species to form an excited state, and subsequent emission process, while returning back to the ground state.^105,194^ The ion-annihilation (excited state formation *via* electron transfer between the cation and anion radicals of luminophore itself) and co-reactant mediated (formation of radical species from co-reactant to react with luminophore and form excited state) routes are implemented to generate ECL systems; however, the latter is more common and can produce a strong ECL signal.^81,105^ The first observation of ECL from N-GQDs (greenish-yellow luminescence) in the presence of 0.1 M K_2_S_2_O_8_ co-reactant was reported as far back as early 2012.^195^ A schematic of NIR-ECL generation through the GQDs/K_2_S_2_O_8_ co-reactant system is shown in Fig. 5. By applying a negative potential, peroxodisulfate (S_2_O_8_^2−^) and GQDs get reduced to the corresponding radical anions nearby the cathode surface. Thereafter, the second reduction of the GQD^−^˙ radical anion to GQD^2−^ dianion and its subsequent reaction with neutral GQDs generated two GQDs^−^˙ radical anions. At the same time, the sulphate radical anion (SO_4_^−^˙) is produced from the S_2_O_8_^3−^˙ radical anion by the loss of the SO_4_^2−^ anion, which is further reduced by the GQD^−^˙ radical anion to generate excited-state GQDs* and SO_4_^2−^ anion. Furthermore, the relaxation of GQDs* to the surface excited-state GQDs^S^*, followed by returning to the ground state resulted in the emission of a strong NIR-ECL signal.^105^

### 4.4. EC characteristics

The large surface-to-volume ratio, abundant active sites, reliable electrical conductivity, and rapid electron transfer ability of GQDs/modified-GQDs qualify them as electrode modifiers for amplified EC signals. Both GQDs and modified-GQDs have been used to enhance the EC activity. For instance, a significant reduction in the electron transfer resistance with a GQDs/Nafion modified glassy carbon electrode (GCE) (GQDs/Nafion@GCE) in comparison to Nafion@GCE indicated favorable electron transfer kinetics in the GQD/Nafion electrode material, and therefore better EC activity.^196^ Around 10% nitrogen-doping and aromatic structure of N-GQDs provided surplus available electrons for enhanced conductivity and electrocatalytic activity.^197^ Covalently functionalized DMC–GQDs showed a better current response compared with bare GQDs, which is attributed to the active surface area and fast charge transfer rate of modified-GQDs.^103^ Dual-doping is also beneficial to enhance the conductivity and electrocatalytic activity of N,S-GQDs. This is possible due to the incorporation of bonding and anti-bonding sulfur orbitals between the bonding orbital of carbon and anti-bonding orbital of nitrogen, which improved the electron availability and narrowing of the band gap structure in the N,S-GQDs (band gap of N,S-GQDs/N-GQDs: 0.98/1.3 eV).^198^

## 5. Diverse applications of GQDs-based systems

Carbon-based dots are one of the preferable alternatives, especially where metal-containing QDs are employed in a specific application.^199^ The effective quantum confinement, good crystallinity, hydrophilicity, high surface-to-volume ratio, chemical stability, favourable electronic structures, facile charge transportation, and biocompatibility of GQDs permit them to be used in biological, optoelectronic, agricultural, environmental, and energy-related applications. Additionally, the application prospects/outcomes of GQDs can be further broadened by tuning their intrinsic properties through heteroatom-doping and post-functionalization. The compositing/heterostructuring of GQDs/modified-GQDs with other active counterparts is also a noteworthy route to improve their performance metrics. The wide range of applications of GQDs-based systems can be identified from the following representative examples: GQDs synthesized from neem extract showed bioactivity with better antibacterial and antioxidant performances compared to the starting extract.^151^ Nd,N-GQDs and thulium (Tm)-doped N-GQDs have shown potential as contrast agents for dual-mode biomedical imaging (ultrasound and NIR fluorescence) applications. Intravenously injected doped-GQDs (particularly, Nd,N-GQDs) in mice and animal organs significantly enhanced the response of both modes to achieve precise, accurate, and sensitive imaging for diagnostic and monitoring during therapeutic treatment.^159^ Due to the light-induced reactive oxygen species generation ability of GODs, GQDs with 132 conjugated carbon atoms (single-molecule) were applied for successful cancer eradication *via* photodynamic therapy (both *in vitro* and *in vivo*).^200^ Blue- and green-emissive GQDs were coupled with poly(sodium 4-styrenesulfonate) fibers to construct a nanocomposite with colour-tuneable and white light emission activity.^201^ Biocompatible GQDs were employed for boosting sustainable agricultural activity and showed a significant enhancement in nitrogen fixation (471.7% activities with respect to the control of *Azotobacter vinelandii*), along with an increase in the nitrogen content in soil.^202^ The nanocompositing of GQDs with graphitic carbon nitride (g-C_3_N_4_) resulted in an efficient photocatalyst for the degradation of Rhodamine B dye (optimum degradation efficiency: 95.2% within 2 h, catalyst concentration: 35 mg l^−1^).^203^ N-GQDs NiAl LDH/TiO_2_ (LDH = layered double hydroxide) heterostructures with 10 wt% N-GQDs loading exhibited efficient hydrogen production capability (1332.11 µmol g^−1^ h^−1^) *via* their photocatalytic activity under solar irradiation. Here, N-GQDs played a crucial role in mediating facile charge passage between NiAl LDH and TiO_2_ to delay electron–hole recombination.^204^ N-GQDs pillared Ta_4_C_3_T_*x*_ MXene exhibited a significant improvement in gravimetric capacitance (701 F g^−1^; bare MXene: 100 F g^−1^) and applicability to fabricate an asymmetric supercapacitor with specific capacity, energy density, power density, and cyclic stability of 110C g^−1^, 55 Wh kg^−1^, 9000 W kg^−1^, and 20 000 cycles, respectively.^205^

Sensing of pollutants, bio-related species, and other items is another fascinating area of research, which is continuously progressing with the aim of improving the performance, utility and cost of existing probes, and applicability of user friendly detection techniques. Owing to the advantage of abundant functional groups and edge sites in GQDs/modified-GQDs, along with size-based confinement origin and other intriguing features, GQD-based systems have been extensively explored as a low cost and effective platform for the targeting of various analytes. The structural/compositional characteristics of GQDs and modified-GQDs make them suitable to interact with target substances selectively and respond accordingly. GQDs-based FL sensors function based on the quenching or enhancement of their fluorescence response when they contact the analyte. A colour change in the probe solution in the presence of analyte constitutes a COL sensor, which provides an opportunity for the visual monitoring and quantification of analytes using UV-visible absorbance spectra. Changes in the CL and ECL signals (generated from GQDs-based platforms) can also be monitored to quantify various analytes. The EC sensing strategy refers to analyzing substances through the electrode surface-confined charge transfer phenomenon, and consequently changes in the current/voltage response. Voltammetry-based methods including cyclic voltammetry (CV), differential pulse voltammetry (DPV), square wave voltammetry (SWV), and anodic stripping voltammetry (ASV) have been potentially used during the EC detection process.^206^ Some recent sensing attributes of GQDs-based systems are as follows: GQDs (band gap engineered from 3.3 to 1.9 eV by varying the graphitic core size of GQDs) were used as a photo-sensitizer to tune the dynamics of carrier transfer in GQDs-decorated In_2_O_3_. Consequently, the optimum GQDs (7 nm)/In_2_O_3_ sensor system showed efficient monitoring of environmentally hazardous gas NO_2_ under the visible light (blue)-activated photocatalytic sensing strategy with a recognizable response (*R*_g_/*R*_a_) of 97.1 at 1 ppm and rapid response/recovery time of 136/100 s.^207^ N-GQDs were coupled with a Prussian blue (PB) analogue-containing PB layer to assemble a wearable biosensor for the EC detection of H_2_O_2_ with sensitivity as high as 221.29 ± 1.77 µA mM^−1^ cm^−2^. Additionally, the immobilization of glucose oxidase on the conjugated composite resulted in a nanocomposite assembly for the selective monitoring of glucose with high sensitivity (90.49 ± 1.08 µA mM^−1^ cm^−2^).^208^ An enhancement in the CL response of luminol-H_2_O_2_ by incorporating N-GQDs, and its subsequent suppression in the presence of tetrabromobisphenol A (TBBPA; an environmental contaminant) were integrated with an SiO_2_@TBBPA MIP (MIP = molecular imprinted polymer) assembly to detect TBBPA with a detection limit of 0.032 nM.^209^ Coating of Co-modified exfoliated zirconium phosphate on the functionalized GQDs (His–GQDs) resulted in a synergistic electrocatalyst for the EC detection of methyl parathion (a toxic pesticide) with a detection limit of 10 nM and sensitivity up to 0.85 mA µM^−1^.^210^

Various metals in ionic form and many of inorganic anions are potential pollutants in the environment and living organisms. Conversely, alkali/alkaline-earth MIs are biologically important to regulate and control metabolic cycles. Therefore, the identification of inorganic ions with good sensitivity, selectivity, and easily implemented detection methods using a facile probe is an obvious environmental and biological concern. Moreover, the deployment of sensors and sensing strategies for reliable, on-site, real sample/water-body, and track-ability detection in living systems is also vitally important. Here, we discuss various GQDs-based and GQDs involved systems for the detection of inorganic ions, employing various sensing approaches.

## 6. GQDs-based/involved sensors in the detection of HMIs and other MIs

### 6.1. Fe^3+^

#### 6.1.1. Doped- and undoped GQDs

In an earlier report (2013), green-emitting graphitic N-GQDs with abundant oxygen-containing functionalities specifically interacted with Fe^3+^ (high binding affinity with phenolic –OH groups) for fluorescence quenching to achieve a low limit of detection (LOD, Table 2).^211^ Subsequently, single- and dual-heteroatom doped-GQDs, MIs doped-GQDs, and undoped GQDs have been extensively explored for the assay of Fe^3+^ (Tables 2 and S1). Among the single-heteroatom doped-GQDs, N-GQDs are one of the preferred choices for the FL turn-off based detection of Fe^3+^ (Tables 2 and S1). For instance, bottom-up-synthesized N-GQDs (15.7% nitrogen) showed better sensitivity (Table 2) compared to the undoped GQDs (linear range (LR): 1–594 µM), indicating the nitrogen element-induced modification of the chemical-electronic structure for effective complexation between N-GQDs and Fe^3+^. However, due to the significant quenching effect of Hg^2+^/Cu^2+^ on the N-GQDs, masking agents were required to circumvent their interference.^212^ N-GQDs (bluish-green fluorescence) synthesized *via* the ST method showed the possibility for the selective detection of Fe^3+^ in a wide LR (Table 2), which is explained based on their strong affinity with surface-bound –NH_2_/–COOH groups to promote electron transfer between them (rapid quenching within 30 s). However, mechanistic details were beyond the scope of that investigation.^213^ Li *et al.*^191^ first employed N-GQDs (9.25% nitrogen) to sense Fe^3+^*via* the CL method. The N-GQDs catalyzed the KMnO_4_–Na_2_S redox reaction to measurably enhance CL signal (20-fold) by the involvement of ˙OH radical. Importantly, undoped GQDs did not showed this improvement in the CL response, which indicates the crucial role of nitrogen-doping (particularly, pyridinic configuration) for catalyzing the CL reaction. With the addition of Fe^3+^, the CL signal gradually decreased due to the chelation effect. Although this probe showed selectivity in the presence of other interfering MIs within the concentration of Fe^3+^ (maximum: 1 µM), the high concentrations of ionic contaminants in real samples limit its wide applicability. The utility of biomass (Marigold)-derived N-GQDs in the selective recognition of Fe^3+^ with satisfactory sensitivity (Table 2) and tracking of Fe^3+^ in HeLa cells (human epithelial cancer cells), along with its quantification in real water specimens opens a sustainable possibility for the probing of Fe^3+^.^214^

**Table 2:** GQDs, modified-GQDs, and GQDs involved with other counterparts for Fe^3+^ and Fe^2+^ sensing application^a^

GQDs-based sensor	Synthesis conditions	Size range/average size^b^ (nm)	QY (%)	Sensing process	LR (µM)	LOD (µM)	Ref.
**Fe** ^**3+**^							
**Doped-/undoped GQDs**							
N-GQDs	Carbonization of pyrene with HNO_3_:H_2_SO_4_ (1 : 3) under reflux (95 °C, 48 h); centrifugation; HT with hydrazine hydrate/25 wt% NH_3_ (180 °C, 24 h); centrifugation; dialysis	5–10/5.5	11.7	FL, turn-off	0.5–20	0.005	211
N-GQDs	Pyrolysis (CA, 200 °C, 30 min); dissolved in 10 mg per mL NaOH & pH adjusted to 8.0; HT treatment with 30% hydrazine (180 °C, 12 h); centrifugation	2.2–5.3/3.8	23.3	FL, turn-off	1–1105	0.09	212 o
N-GQDs	ST (GSH/AgNO_3_ in ethylene glycol, 200 °C, 12 h); centrifugation	1–5/2.5	—	FL, turn-off	50–2000	0.07	213 o
N-GQDs	HT (CA/urea in water, 200 °C, 6 h); filtration; dialysis	3.5–6/4.8	—	CL, turn-off	0.01–1	0.004	191 o
N-GQDs	Pyrolysis (Marigold granules, 1000 °C, 5 h, Ar); acid oxidation with HNO_3_:H_2_SO_4_ (1 : 3) under reflux (90 °C, 5 h); filtration; pH adjusted to 7.0; dialysis; drying; HT (obtained powder in ethylenediamine solution, 200 °C, 10 h)	1.5–4.5/3.2	7.84	FL, turn-off	0–20, 200–667	0.0411, 0.5	214 o ^,^ p
N-GQDs	ST (GO in DMF, 200 °C, 5 h); centrifugation; filtration	1.1–5.3/3.17	14.32^e^	FL, turn-off	0–34	0.00238	165 o ^,^ q
N-GQDs	HT (Bamboo fiber powder/urea in water, 200 °C, 8 h); filtration; dialysis	2–20/5	40.36	FL, turn-off	1–1000	0.034	215 p
N-GQDs	HT (aspartic acid/urea in water, 180 °C, 8 h); centrifugation	1–4/2.22		FL, turn-off	100–600^g^	—	216
Mg,N-GQDs	HT (aspartic acid/urea/MgCl_2_·6H_2_O in water, 180 °C, 8 h); centrifugation	0.8–2/1.31		"	150–450^g^	"	
S-GQDs	Electrolysis of graphite rod in 0.1 M SPTS aqueous solution, 3 h; filtration, dialysis	2–4/3	10.6	FL, turn-off	0.01–0.70	0.0042	175 o
B-GQDs	Electrolysis of graphite rod in 0.1 M borax aqueous solution, 2 h; filtration, dialysis	3–6/4.5	5.2	FL, turn-off	0.01–100	0.005	217 o
N,S-GQDs	HT (acid hydrotrope fractionation of *Miscanthus*/*p*-amino-benzene sulfonic acid monosodium salt in water, 200 °C, 12 h); filtration; dialysis	—/4.05	20.2	FL, turn-off	0–10.6^h^, 10.6–900^h^	0.00141^h^	218
				"	0–10.6^i^, 10.6–800^i^	0.00231^i^	
				"	0–10.6^j^, 10.6–1000^j^	0.00209^j^	
Er-GQDs	HT (Lactose/Er(NO_3_)_3_·5H_2_O in water, 200 °C, 4 h); filtration; dialysis	2–8/4.7	18	FL, turn-off	0.01–1^k^, 1–120^k^	0.0028^k^	171 o
				"	0.1–20^l^, 20–200^l^	0.028^l^	
GQDs	HT (Rice husk powder in water, 150 °C, 5 h); filtration; centrifugation	2.5–5.5/3.9	8.8	FL, turn-off	0–300	0.0058	220
GQDs-1	HT (2 mg per mL GO in water, pH adjusted to 9.5, 130 °C, 10 h); filtration; freeze drying	4–8/5.8	6	FL, turn-off	1–8.75	0.136	221
GQDs-2	HT (2 mg per mL GO in water, pH adjusted to 8.0, 175 °C, 10 h); filtration; freeze drying	"	8.9	"	1–75	1.36	

**Functionalized GQDs**							
RBD–N-GQDs	Electrolysis of graphite rod in 0.01 M TBAP/DMSO, 3 h; centrifugation & drying; acid oxidation with HNO_3_:H_2_SO_4_ (1 : 3) under reflux (100 °C, 24 h); pH adjusted to 7.0; dialysis; covalently modified with RBD	3.5–6.5/5	43^f^	FL, turn-on	0–1	0.02	133 p
DA–GQDs	Pyrolysis (CA, 200 °C, 25 min); mixed in 10 mg per mL NaOH solution & pH adjusted to 7.0; covalently modified with DA	2–9/4.5	10.2	FL, turn-off	0.02–1.5	0.0076	222
DPA–GQDs	HT (CA/DPA in water, 200 °C, 2.5 h); dissolved in water; dialysis	1–9/4.7	99.8	FL, turn-off	4–1800	1.2	187 o
Am–GQDs	Carbonization (pre-oxidized *Asphalt*, 900 °C, 1 h, He); acid oxidation with HNO_3_:H_2_SO_4_ (1 : 2) under ultrasonication (1 h) & reflux (100 °C, 23 h); diluted with water & pH adjusted to 7.0 by NH_3_; HT (180 °C, 6 h); dialysis	2–3.6/2.3	13.8	FL, turn-off	0–50	5.1 × 10^−4^	164 o
N-GQDs@xylan	Liquid phase exfoliation of graphite flake in NMP/0.1 g NaOH under bath/probe ultrasonication (8 h/4 h); dialysis; filtration; non-covalently modified with 5% xylan under HT (180 °C, 5 h)	1–3/1.97	36.63	FL, turn-off	0–75	0.0928	142
Arg,Ser–B-GQDs	Pyrolysis (CA/Arg/Ser/H_3_BO_3_, 160 °C, 4 h); diluted with water; centrifugation; dialysis	1.0–12/4.8	40.12	FL, turn-off	0–50	0.075	223 o

**GQDs involved with other counterparts**							
LS/GQDs	Pyrolysis (CA·H_2_O, 200 °C, 15 min); treated with 10 mg per mL NaOH containing 20 µL LS under stirring (2 h); pH adjusted to 7.0; dialysis	390–800/590^c^; 2–8/—d	23.3	FL, turn-off	0.005–500	0.0005	224 o
GQDs/PVA@ PETP	HT (glucose/NH_3_ in water, 200 °C, 8 h); dialysis; non-covalently modified with PVA & coated on PETP film	8–17/15.5^d^	—	FL, turn-off	0–30^m^	0.1^m^	225 o ^,^ q
AuNPs@N-GQDs	ST (GO in DMF, 250 °C, 5 h); centrifugation; *in situ* decorated with AuNPs; centrifugation	12.5–33/23.4^c^	12.3	FL, turn-off	0.1–0.75; 0.001–10^m^	0.03; 0.001^m^	226 o
GQDs/CNC_mod_	HT (GO in water, pH adjusted to 9.5, 135 °C, 10 h); filtration; dialysis; incorporated with CNC_mod_ & solvent casted over PETP substrate	<10/—d	—	FL, turn-off	1 × 10^−9^–2 × 10^−6^	8 × 10^−10^	227
AuNPs@N-GQDs	ST (GO in DMF, 200 °C, 5 h); centrifugation; *in situ* decoration with AuNPs	10–40/17^c^; 2–8/4.6^d^	—	Optical, turn-on	—	0.001^n^	228 o
GQDs-Au-Ni micromotor	GQDs solution purchased from ACS materials; electrochemical template deposition of GQDs layer followed by Au and Ni layers on Ag-coated polycarbonate membrane; etching of Ag; removal of membrane by dissolving in DCM	—	—	Solid FL, turn-off	1 × 10^−6^–10	7.0	229
				Magnetic, speed reduction	"	9.0	
GQDs-Au-Ni@ SPCE	GQDs-Au-Ni casted over SPCE electrode			EC, DPV	"	6.0	

**Fe** ^**2+**^							
N,S,I-GQDs	Pyrolysis (CA, 230 °C, 5 min); pyrolysis (melted CA/garlic extract/KI/KIO_3_, 230 °C, 5 min); mixed in 0.25 M NaOH solution	2.36–3.78/—	45	FL, turn-off	0.36–3.6, 3.6–17.98	0.4, 1.16	231 o
GQDs	MW (*Mangifera indica* leaf residue in water); dispersion in ethanol, centrifugation, filtration & drying; MW (slurry in water, 10 min); drying	1–13/7.1	45	FL, turn-off	0–2.5	4.07	124
FA,His,Ser–B,P-GQDs	Pyrolysis (CA/FA/His/Ser/H_3_BO_3_/H_3_PO_4_, 160 °C, 4 h); dissolved in water; centrifugation; dialysis	2–12/4.6	60.2	FL, turn-off	0.01–50	0.0042	166 o

a SPTS: sodium *p*-toluenesulfonate, TBAP: tetrabutylammonium perchlorate, DMSO: dimethylsulfoxide, DCM: dichloromethane.

b Measured from TEM.

c Size range/average size of GQDs involved system.

d Size range/average size of GQDs/doped-GQDs used with other counterparts.

e Absolute QY.

f QY after Fe^3+^ binding.

g LR in µg mL^−1^.

h LR/LOD measured from 358 nm emission peak.

i LR/LOD measured from 408 nm emission peak.

j LR/LOD measured from 358 nm excitation peak.

k LR/LOD measured from down-conversion PL.

l LR/LOD measured from UCPL.

m Dynamic concentration range and corresponding LOD.

n LOD predicted from machine learning algorithm.

o Analytical ability in real water/biological fluid/supplement samples.

p Analytical ability in living cells.

q Paper-based sensing capability.

**Fig. 6:**
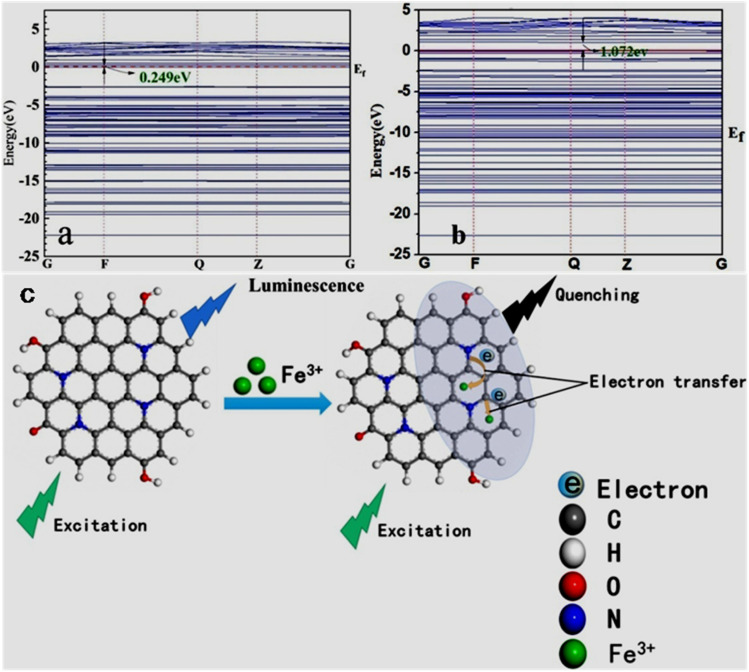
DFT calculation-based energy level pictures of N-GQDs (a) and N-GQDs along with Fe^3+^ (b). (c) Schematic of Fe^3+^ detection *via* an electron transfer-based fluorescence quenching process. Reprinted from ref. 173, copyright 2022, with permission from Elsevier.

The Fe^3+^ probing capability of N-GQDs *via* a quenching-based FL process is illustrated by theoretical calculations. DFT calculations showed a significant increase in the band gap of N-GQDs in the presence of Fe^3+^ (1.072 eV, band gap of N-GQDs: 0.249 eV, Fig. 6a and b), which illustrates the inhibition of the active sites in the nanoprobe at a higher energy level, and therefore electron transfer from N-GQDs to Fe^3+^*via* chelation kinetics, resulting in fluorescence quenching-based Fe^3+^ detection (Fig. 6c).^173^

**Fig. 7:**
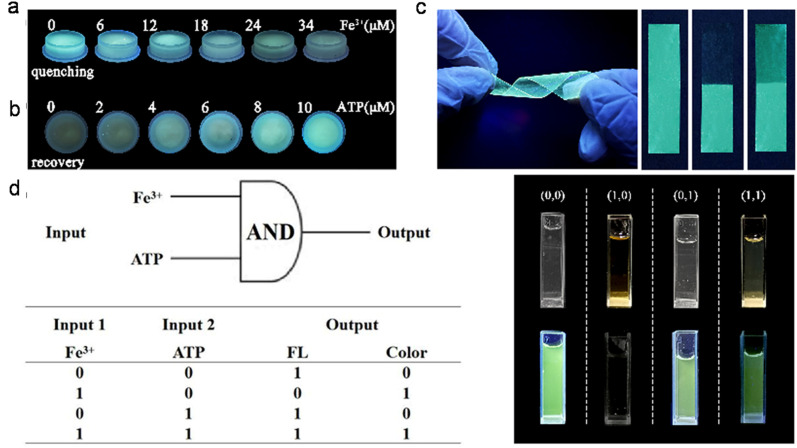
Digital images of a hydrogel kit, showing a gradual fluorescence quenching with 0 to 34 µM concentrations of Fe^3+^ (a) and recovery of fluorescence with 0 to 10 µM concentrations of ATP (b) under a 365 nm UV light. (c) Digital pictures of the membrane under UV light showing flexibility/cyan fluorescence (first two) and turn-off-on response (last two) with 34 µM Fe^3+^ and subsequent addition of 10 µM ATP. (d) Truth table using the input from Fe^3+^ and ATP and corresponding “AND” type logic scheme. Reproduced/adapted from ref. 165 with permission from The Royal Society of Chemistry, 2023.

**Fig. 8:**
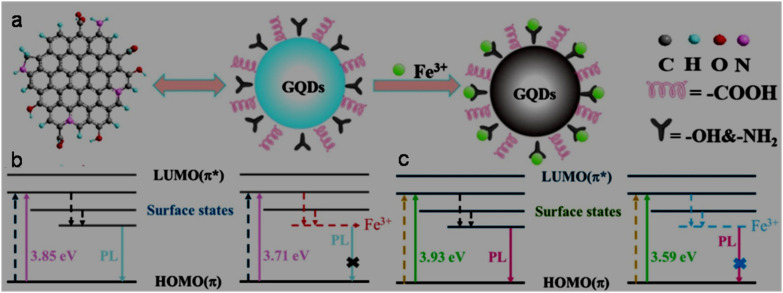
(a) Fluorescence quenching of doped-GQDs after interaction with Fe^3+^. Existing energy levels and electron transitions in (b) N-GQDs and N-GQDs + Fe^3+^ and (c) Mg,N-GQDs and Mg,N-GQDs + Fe^3+^. Reprinted from ref. 216, copyright 2025, with permission from Elsevier.

ST-synthesized N-GQDs with an excitation-independent cyan colour emission (*λ*_em_: 525 nm at 360 nm *λ*_ex_) showed a low LOD of 2.38 nM (LR: 0–34 µM) in the detection of Fe^3+^ through the synergistic effect of –OH group-driven coordination, static quenching effect (SQE), and inner filter effect (IFE). The N-GQDs could also detect Fe^3+^ in mouse serum/human urine (biological samples) with good recoveries/relative standard deviations (RSDs) (98.1–104.6/0.12–2.71%) and considerable inter-day/intra-day precision. Furthermore, the portable sensors (hydrogel kit and flexible film; stable up to 1 month under 4 °C storage conditions), conveniently fabricated by immobilizing N-GQDs in a PVA matrix, exhibited a visual as well as on-site detection capability for Fe^3+^. A gradual decrease in the cyan fluorescence of the hydrogel kit with an increase in Fe^3+^ concentration (0–34 µM) and subsequent recovery with adenosine triphosphate (ATP, 0–10 µM) can be seen in Fig. 7a and b, respectively. Fig. 7c shows the flexibility of the prepared membrane device (without obvious marks after multiple folding) and accurate colour visibility under UV light (cyan fluorescence) and in the presence of Fe^3+^ (quenched fluorescence)/ATP (recovered fluorescence). Moreover, the ‘AND’ logic gate of the portable sensor was correctly executed in the sensing operation by utilizing FL/COL dual readout to achieve good accuracy (Fig. 7d).^165^ Subsequently, Khan *et al.*^215^ demonstrated the use of bamboo fiber (biomass)-derived N-GQDs (QY: 40.36%) for the selective detection of Fe^3+^ with improved sensitivity compared to previous biomass-synthesized N-GQDs (Tables 2 and S1). The coordination between Fe^3+^ and oxygen-containing functionalities (preferably –OH groups) on the surface of N-GQDs facilitated electron transfer from N-GQDs to Fe^3+^, and therefore weakening of the inherent photo-induced electron transfer (PET) process to quench the fluorescence of N-GQDs. The involvement of SQE *via* the formation of a non-fluorescent ground-state complex is evidenced by the high *K*_SV_ calculated from the Stern–Volmer plot (1.31 × 10^4^ M^−1^). Although the fluorescence quenching is relatively greater with Fe^3+^ (∼54%) than Hg^2+^ (∼26%), the interference from Hg^2+^ in real water samples cannot be avoided. The blue luminescence of recently synthesized N-GQDs and magnesium (Mg)-doped GQDs (Mg,N-GQDs) diminished due to the coordination of Fe^3+^ with –OH and –NH_2_ functional groups present on the doped-GQDs (Fig. 8a). The incorporation of MIs in the N-GQDs did not improve their sensitivity for the detection of Fe^3+^ (Table 2) and the effect of doping on their selectivity is unclear. The various energy levels and associated electronic transitions before-after the addition of Fe^3+^ in N-GQDs and Mg,N-GQDs are shown in Fig. 8b and c, respectively, which depict the passage of photo-excited electrons from the doped-GQDs to partially filled Fe^3+^ orbitals to inhibit the routine radiative process and fluorescence signal.^216^

Single-heteroatom doped S-GQDs for Fe^3+^ sensing has rarely been reported in the literature (Tables 2 and S1). Blue-green fluorescent S-GQDs (4.25% sulfur) were employed in early years (2014) for the selective sensing of Fe^3+^ with regeneration ability after quenching operation using ethylenediamine tetraacetic acid (EDTA); however, the narrow LR (0.01–0.7 µM) of this probe limits its wider applicability. Interestingly, the N-GQDs, B-GQDs, and undoped GQDs tested in this study did not exhibit a significant decrease in fluorescence even at 0.7 µM Fe^3+^, indicating the importance of sulfur in GQDs to promote the coordination of phenolic –OH groups with Fe^3+^.^175^ Chen *et al.*^217^ first employed B-GQDs (∼3.2% boron) for the selective estimation of Fe^3+^ with considerable sensitivity (Table 2) and EDTA-induced regenerative characteristics. The quenching-based FL detection is driven by the strong adsorption ability of Fe^3+^ on the surface of B-GQDs and subsequent energy transfer between them. Subsequently, newly synthesized B-GQDs *via* the bottom-up method showed a wider LR but with the compromise of higher LOD (Table S1).

N,S-GQDs are found to be suitable fluorescent probes among the two types of dual doped-GQDs (N,S-GQDs and N,P-GQDs) tested for the sensing of Fe^3+^ (Tables 2 and S1). For instance, valorization of *Miscanthus* biorefinery waste in the form of N,S-GQDs (nitrogen/sulfur content: 2.53/2.83 wt%) was used for the selective as well as tri-channel sensitive detection of Fe^3+^. The emission peaks of N,S-GQDs centred at 358/408 nm and excitation peak at 358 nm provided a tri-channel FL platform to sense Fe^3+^ with high sensitivity and improved precision (LODs (LRs): 1.41 nM (0–900 µM)/2.31 nM (0–800 µM) and 2.09 nM (0–1000 µM)). The XPS, FTIR, and time-resolved PL (lifetime (*τ*) changed from 11.95 to 9.98 ns after adding Fe^3+^) results inferred a collision-type dynamic quenching effect (DQE) between the probe and analyte rather than the common SQE in the detection of Fe^3+^. Noticeably, the sensitivity and tri-channel-based accuracy of this biomass-derived probe for the quantification of Fe^3+^ surpassed the sensing performance of single-/dual-heteroatom doped-GQDs *via* the FL method (Tables 2 and S1); however, potential interference from Cr_2_O_7_^2−^ was detected during the selectivity test.^218^

The incorporation of MIs in the structure of GQDs to improve their optical properties and selectively detect Fe^3+^ has also been reported in the literature (Tables 2 and S1). For example, a rare-earth (Er) inclusion in the form of Er-GQDs (1.8 at% Er-doping) with down-conversion/UCPL characteristics at *λ*_ex_ of 360/730 nm showed a good Fe^3+^ sensing performance (Table 2). Interestingly, the UCPL-based detection of Fe^3+^ showed a larger LOD (28 nM) in comparison to the down-conversion-based detection process (2.8 nM), which is attributed to the weak fluorescence intensity and relatively low quenching response in the up-conversion domain. Moreover, the detection of Fe^3+^ in human serum also validated the response of the sensor in both down-conversion/up-conversion domains with LODs of 11.2/336 nM, justifying its good analytical performance towards the higher concentration side of Fe^3+^ in the biological sample.^171^

The analytical ability of bare GQDs for the detection of Fe^3+^ is also revealed in Tables 2 and S1. Zhu *et al.*^219^ presented insight into the selectivity of GQDs (containing phenolic –OH groups) towards Fe^3+^*via* the formation of GQDs-aggregates under acidic conditions (pH: 3.5). Due to the extremely lower *K*_sp_ (solubility-product constant) of Fe(OH)_3_ (2.8 × 10^−39^) in comparison to Cu(OH)_2_/Ni(OH)_2_/Co(OH)_2_ (2.2 × 10^−20^/5.0 × 10^−16^/2.3 × 10^−16^) at a lower pH, the formation of Fe(OH)_3_ induced the aggregation of GQDs, resulting in fluorescence quenching. The potentiality of biomass-derived GQDs (from rice husk powder) in the fluorescence quenching-based detection of Fe^3+^ can be appreciated by their satisfactory sensing performance (Table 2).^220^ Two HT conditions for the scissoring of GO (Table 2) showed feasibility for the synthesis of GQDs with different amounts of –COOH/–OH functional groups, and correspondingly varying sensing performance levels. It was observed that high –COOH-containing GQDs-1 resulted in a lower LOD (0.136 µM), while GQDs-2 with a higher QY (8.9%) and –OH groups at their edge were advantageous for a wider LR (1–75 µM, LOD: 1.36 µM) in the turn-off based detection of Fe^3+^. Here, –COOH groups are considered hard binding sites for Fe^3+^ (hard HMI) according to the hard-soft acid-base (HSAB) theory, and therefore a lower LOD in the case of GQDs-1.^221^ Later, biomass-based GQDs and F-rich GQDs were further utilized to sense Fe^3+^ but with an inferior performance (Table S1). Noticeably, spent tea-derived GQDs with the involvement of oxone oxidant in the synthesis process showed a lower LOD for Fe^3+^ detection rather than without the addition of acid-oxidant in the ethanol-assisted single-step ST synthesis (Table S1), which may be related to the structure and different surface states of GQDs under the two synthesis conditions.

#### 6.1.2. Functionalized GQDs

The Fe^3+^ sensing results of GQDs/doped-GQDs functionalized with various chemical moieties are summarized in Tables 2 and S1. For instance, covalently modified RBD–N-GQDs (nitrogen content: 7.08%) were used to selectively detect Fe^3+^*via* an unusual fluorescence enhancement process (Table 2). The greenish-yellow emission (*λ*_em_: 520 nm) of GQDs was red-shifted to yellow luminescence (*λ*_em_: 550 nm) after RBD functionalization. When Fe^3+^ was added to the system, a new/strong orange-red emission (*λ*_em_: 580 nm) was observed due to the spirolactam ring opening of RBD, and consequently the QY increased to 43%. The 580 nm emission peak progressively increased to trace Fe^3+^ and the probe showed biomedical applicability (track Fe^3+^ in HeLa and pancreatic cancer stem cells (CSCs) *via* bright orange-red fluorescence); however, its narrow LR and interference from aluminium ions (Al^3+^) are some of its limitations.^133^ Subsequently, DA–GQDs were successfully applied for the nanomolar-level detection of Fe^3+^ due to the strong coordination ability of DA with Fe^3+^, followed by the oxidation of the catechol moiety to *ortho*-semiquinone. However, although DA–GQDs showed a low Fe^3+^ LOD (7.6 nM), the nanoprobe was irreversible in nature (could not be regenerated after the addition of EDTA) and only applicable in a lower detection range.^222^ A wide LR of 4–1800 µM in the fluorescence quenching-based quantification of Fe^3+^ from DPA-functionalized GQDs (DPA–GQDs, QY as high as 99.8%) was achieved, which could also quantify Fe^3+^ in iron supplement oral liquids (recovery: 98.2–102.5%) without measurable interference from Fe^2+^ (2000-fold concentration). The authors proposed that the functional groups on DPA–GQDs favour the formation of a stable octahedral complex with Fe^3+^ rather than unstable tetrahedral complexation with Fe^2+^.^187^ Later, naturally available high-softening point asphalt precursor-derived Am–GQDs (yellow-emissive) with abundant amide/amino groups exhibited a considerable sensing performance for Fe^3+^ with a very low LOD (0.51 nM), which is probably the lowest LOD reported thus far among the undoped/doped-/functionalized GQDs-based sensors for Fe^3+^*via* the FL method (Tables 2 and S1). This probe also showed a reversible binding affinity with Fe^3+^ (released after adding EDTA) and promising applicability in real river water samples (LR: 0–90 µM and recovery: 95–105%).^164^

**Fig. 9:**
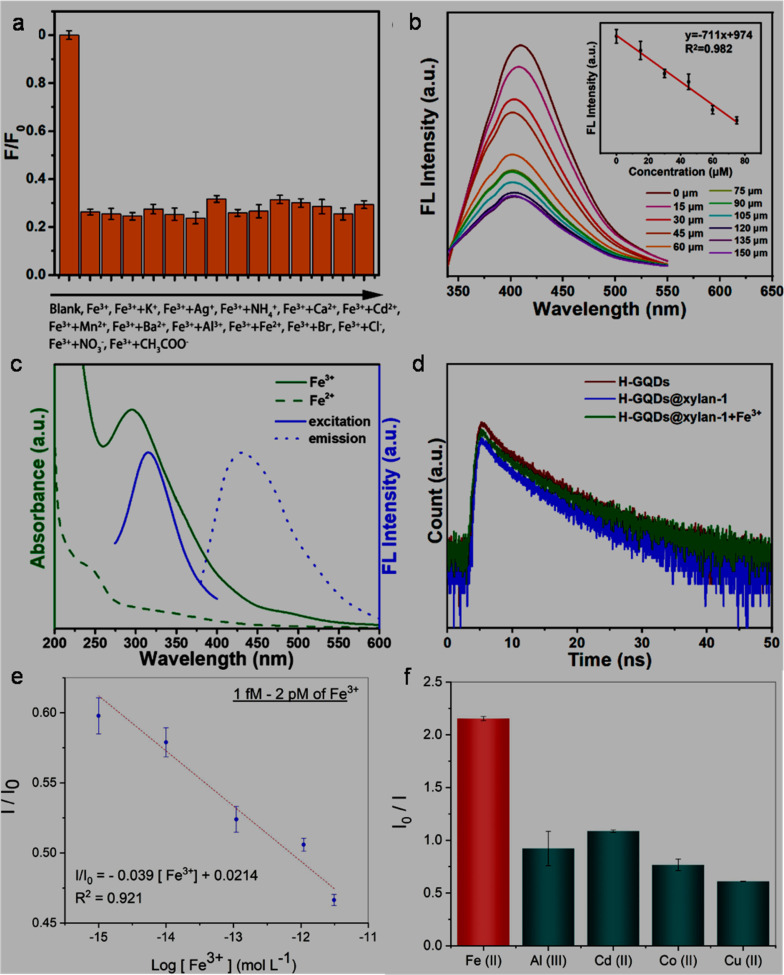
(a) Fluorescence quenching response of N-GQDs@xylan with Fe^3+^ without much effect from the presence of various ions. (b) Fluorescence spectra of N-GQDs@xylan in the presence of Fe^3+^ (0 to 150 µM), showing a gradual decrease in fluorescence intensity and a linear plot of 409 nm fluorescence intensity with respect to Fe^3+^ concentration (inset). (c) Excitation-emission spectra of N-GQDs@xylan, showing an overlap with the UV-visible absorption spectrum of Fe^3+^ rather than Fe^2+^. (d) Time-resolved fluorescence decay profiles of N-GQDs, N-GQDs@xylan, and N-GQDs@xylan in the presence of Fe^3+^. Reprinted (adapted) with permission from ref. 142, copyright 2023, the American Chemical Society. Linear calibration plot of the fluorescence intensity ratio (*I*/*I*_0_) *vs.* concentration of Fe^3+^ (e) and maximum quenching efficiency with Fe^3+^ among the tested MIs (f) using GQDs/CNC_mod_ as a fluorophore. Reprinted (adapted) with permission from ref. 227, copyright 2023, the American Chemical Society.

Cai *et al.*^142^ described the simple ultrasonication-assisted exfoliation of graphite flakes in NaOH/NMP solution to produce N-GQDs (QY: 19.12%), which were non-covalently passivated by hydrophilic saccharide (xylan) to improve their solubility/stability in aqueous medium and QY up to 36.63%. The resultant N-GQDs@xylan nanoprobe showed almost no interference from other cations/anions (Fig. 9a) and a satisfactory sensing performance for Fe^3+^ (LOD/LR: 92.8 nM/0–75 µM, Fig. 9b). The quenching mechanism in the detection of Fe^3+^ is ascribed to the combined effect of IFE (excitation-emission of N-GQDs@xylan overlaps with the absorption of Fe^3+^, Fig. 9c) and charge transfer (insignificant change in *τ* after the addition of Fe^3+^, Fig. 9d) between the fluorophore and analyte.

Recently, Ye *et al.*^223^ employed dual-emissive (*λ*_em_: 460/555 nm at *λ*_ex_: 370/480 nm) arginine (Arg) and Ser-functionalized B-GQDs (Arg,Ser–B-GQDs, QY: 40.12%) for the quantification of Fe^3+^*via* the gradual weakening of their 555 nm yellow-emission peak. Interestingly, the LOD measured at 370 nm UV excitation is much higher (12 400 nM) than the 480 nm visible excitation (75 nM), highlighting the good sensitivity of this probe in the visible light-induced sensing process. Alterations in the electronic structure of the probe after boron-doping narrowed its bandgap to improve its visible light absorption and dual functionality, further synergising its optical properties. Its relatively high selectivity (especially, against Fe^2+^) is ascribed to the strong coordination ability of Fe^3+^ with the oxygen/nitrogen-containing functional groups present on the surface of the probe to form a stable octahedral structure. Additionally, the constructed probe exhibited satisfactory reproducibility (1.7% RSD after 50 successive cycles), long-term stability (2.1% RSD after 6 weeks), and applicability in iron-fortified beverage samples (recoveries and RSDs: 99.4–100.8% and 1.2–2.5%).

#### 6.1.3. GQDs involved with other counterparts

GQDs/modified-GQDs are effectively encountered with other counterparts to construct sensing platforms for Fe^3+^ (Tables 2 and S1). For instance, a core/shell hybrid of lignin sulfonate (LS)/GQDs (LS/GQDs) showed a satisfactory performance in the FL-based sensing of Fe^3+^ (Table 2). Here, the π-rich and sulphur-containing LS molecules on the GQDs favoured a 4-fold higher fluorescence intensity in comparison to bare GQDs, in addition to their chelating ability with Fe^3+^ to achieve good sensitivity/selectivity.^224^

**Fig. 10:**
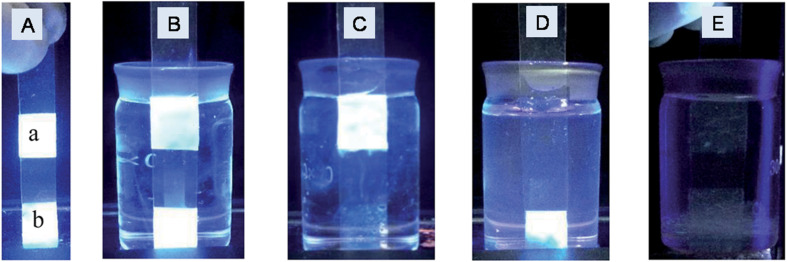
UV light illuminated digital photographs of N-GQDs (a) and GQDs (b) coated test-paper (A) and test-paper dipped in real drinking water (B). Different fluorescence quenching responses of test-paper dipped in drinking water containing 5 µM Fe^3+^ (C), 5 µM Hg^2+^ (D), and 5 µM Fe^3+^ and Hg^2+^ (E). Reproduced/adapted from ref. 225 with permission from The Royal Society of Chemistry, 2018.

GQDs or N-GQDs were non-covalently passivated with PVA and coated on a polyethylene terephthalate (PETP) film to fabricate a test paper-based convenient platform (stable and low cost) for the online detection of Fe^3+^ or Hg^2+^. The effective diffusion of HMIs in the fabricated kit showed a quick (<2 min) and real-time detection avenue; however, the kit is still not suitable for trace-level quantification. The visual fluorescence response of the test paper (simultaneously coated with N-GQDs/PVA (Fig. 10A(a)) and GQDs/PVA (Fig. 10A(b)) in real drinking water can be seen in Fig. 10B, which was separately or simultaneously quenched when 5 µM Fe^3+^ (Fig. 10C), 5 µM Hg^2+^ (Fig. 10D) or 5 µM Fe^3+^ and Hg^2+^ (Fig. 10E) was added, indicating the simultaneous and rapid detection of both HMIs in the real samples.^225^ The local optical field/edge functional groups in N-GQDs are enhanced/modified in the AuNPs@N-GQDs heterostructure to significantly improve its fluorescence intensity (∼12.1-times higher than N-GQDs). The interfacial and strong coupling of the plasmonic AuNPs with N-GQDs favoured an enhancement in electron density on N-GQDs to develop an approach for the fabrication of highly fluorescent nanoprobes. As a result, the heterostructure probe exhibited a low LOD of 1 nM for Fe^3+^ by applying the unconventional Langmuir adsorption law and non-radiative charge transfer dynamics in the entire detection range (0.001–10 µM), justifying the high sensitivity of the GQDs involved heterostructures (sensitivity of N-GQDs and N-GQDs/AuNPs mixture: 100 and 1000 nM, respectively). Their EDTA-triggered reversibility is also advantageous for multi-times sensing activity.^226^ Subsequently, a self-standing modified cellulose nanocrystal (CNC_mod_) thin film-hosted GQDs optochemical sensor (GQDs/CNC_mod_) was applied for the trace-level detection of Fe^3+^ (LR/LOD: 0.001–2/0.0008 pM, Fig. 9e), surpassing the Fe^3+^ detection limits by all other FL sensors (Tables 2 and S1). The coherent interaction between the sensing probe and Fe^3+^, resulting in the quenching phenomenon, can be ascribed to the high *K*_SV_ values of 6.7 × 10^−14^/5.8 × 10^−10^ M^−1^ at Fe^3+^ concentrations of 0.001/2.0 pM. Moreover, the approximately double the fluorescence quenching with Fe^3+^ in comparison to other HMIs (Al^3+^, Cd^2+^, Co^2+^, and Cu^2+^, Fig. 9f) justified the appropriate selectivity of the sensor device. However, although this probe achieved a high level of sensitivity in the detection of Fe^3+^, its fabrication process is very specific and involves complicated steps.^227^

Recently, Das *et al.*^228^ integrated machine learning (ML) with a solid-state photodetector to develop a suitable algorithm for the optimization of the Fe^3+^ sensing performance (experimental data for ML-based prediction is obtained from AuNPs@N-GQDs heterostructure). After optimization of the operating wavelength, the ML-trained model showed nearly 100% selectivity and nanomolar-level sensitivity (LOD: 1 nM) for Fe^3+^. Moreover, the constructed solid-state sensor could respond to Fe^3+^ in real-world samples (river water) at the lowest concentration of 10 nM. Apart from its robust stability (experimentally as well as ML-predicted), the strong affinity between Fe^3+^ and the probe facilitated the formation of Fe–O bonds and light-induced chare transfer for the current response (increasing/decreasing trends of dark/light current with an increase in the concentration of Fe^3+^) in the sensing operation, which was validated by the experimental and ML-based heatmap analyses. In another recent report, a GQDs-Au-Ni tubular micromotor exhibited a decreasing trend in its solid-state fluorescence intensity and speed (under a magnetic field) with an increase in the concentration of Fe^3+^ to quantify Fe^3+^ but with poor sensitivity (Table 2). Moreover, GQDs-Au-Ni@SPCE (SPCE: screen-printed carbon electrode) was also tested for the EC detection of Fe^3+^ (LOD: 6 µM).^229^

*Summary*: According to the above discussion, we can infer that turn-off based FL detection is common for Fe^3+^. The Fe^3+^ detection capability of N,S-GQDs is superior to that of single-heteroatom doped-GQDs and other dual-element doped alternatives. Among the single-hetero-element doped GQDs, N-GQDs are preferable probes due to their easy synthesis and good sensing performance; however, B-GQDs can also effectively sense Fe^3+^*via* the FL method. Moreover, additional functional groups existing in functionalized GQDs (particularly, amide and amino) can selectively interact with Fe^3+^ and achieve a low LOD of 0.51 nM. The selectivity of GQDs/modified-GQDs with Fe^3+^ originates from its stable octahedral complexation with their functional groups. Non-covalent functionalization of N-GQDs with biocompatible xylan can also construct a good FL probe for Fe^3+^. Heteroatom-doping as well as incorporating multiple functional groups in GQDs can build a visible light-driven sensor for good Fe^3+^ sensing activity. Specifically, the incorporation of boron and covalent functionalization with multiple amino acids in GQDs can result in intense dual emission (blue and yellow) and satisfactory sensitivity/selectivity in the detection of Fe^3+^ using low energy visible light excitation. The excellent selectivity and high sensitivity of composite/heterostructure systems composed of GQDs/modified-GQDs involved cannot be ignored. For instance, LS/GQDs core–shell composites and AuNPs@N-GQDs heterostructures may be representative platforms for effective Fe^3+^ sensing. Furthermore, the new development of ML-based predication of their sensing metrics is noticeable and opens a new direction to identify contaminants with minimum experimental efforts.

### 6.2. Fe^2+^

Saenwong *et al.*^230^ first demonstrated the indirect speciation of Fe^2+^ in the presence of H_2_O_2_*via* Fenton reaction to produce Fe^3+^ for the fluorescence quenching of GSH–GQDs (Table S1). Subsequently, the direct detection of Fe^2+^ was demonstrated using doped-/undoped/functionalized GQDs (Tables 2 and S1). For example, doped N,S,I-GQDs (nitrogen/sulfur/iodine content: 1.41/0.41/0.85%) were the first fluorescent nanoprobe that could directly determine Fe^2+^*via* an AIQ-based mechanism (Table 2). However, EDTA and AgNO_3_ are used as a general and Fe^3+^-specific masking agent during the detection process, respectively.^231^ A purely biogenic (*Mangifera indica* leaves) and MW-assisted green approach yielded undoped GQDs (QY: 45%) for the fluorescence quenching-based detection of Fe^2+^ but with low sensitivity compared to previous results (Table 2).^124^

**Fig. 11:**
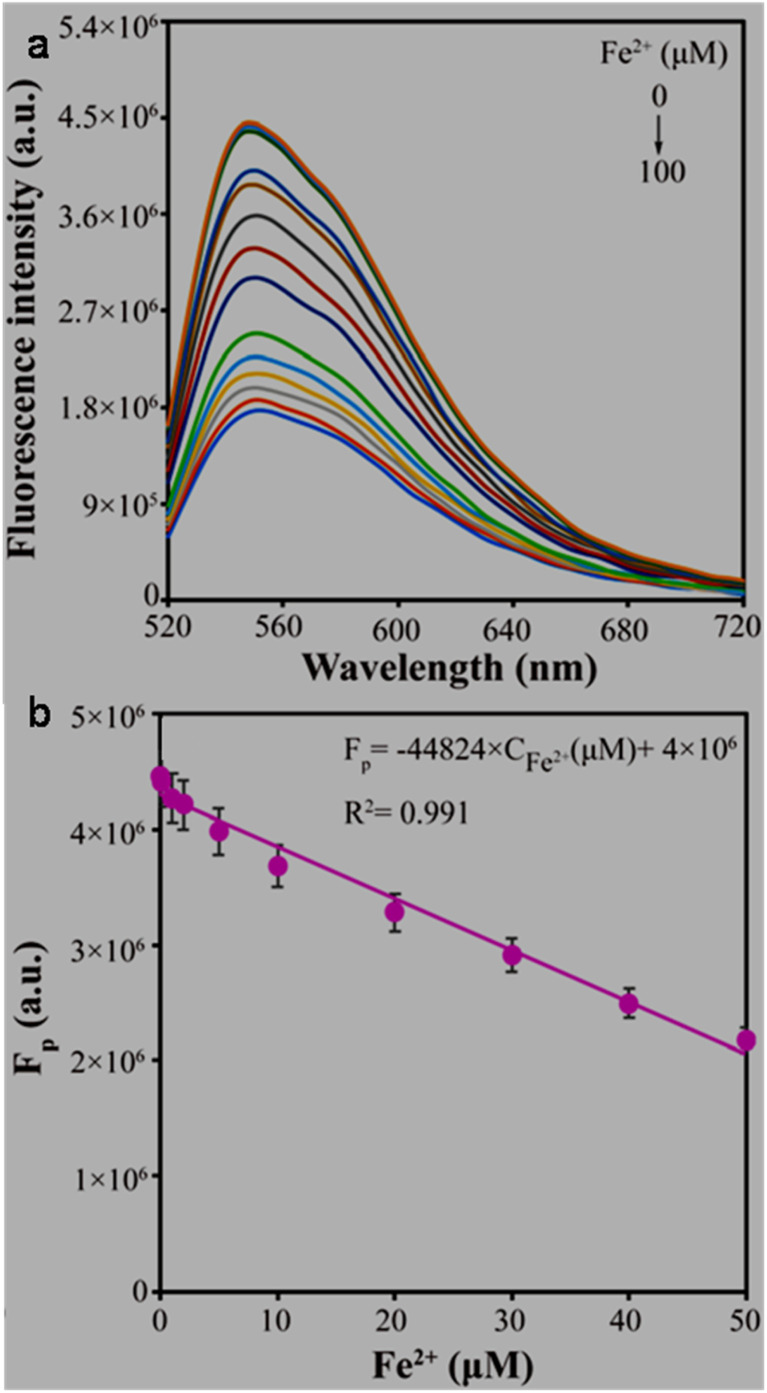
Fluorescence spectra of FA,His,Ser–B,P-GQDs with the addition of 0 to 100 µM concentration of Fe^2+^ (a) and linear plot of *F*_p_ (final to initial intensity ratio) *vs.* Fe^2+^ concentration (0 to 50 µM) (b). Reproduced/adapted from ref. 166 with permission from The Royal Society of Chemistry, 2024.

Recently, a synergistic effect of multi-functionality and dual-heteroatom doping in the FA,His,Ser–B,P-GQDs fluorophore resulted in an intense yellow-emission in aqueous solution (*λ*_em_: 550 nm at 490 nm *λ*_ex_ and QY: 60.2%) and significant yellow-emission even at a high concentration (6 mg mL^−1^) or in the solid state. The visible light-driven (490 nm excitation) sensing aptitude of Fe^2+^ with this nanoprobe in the presence of *ortho*-phenanthroline (Phen) showed a gradual decrease in fluorescence intensity (Fig. 11a), following a wide LR (0.01–50 µM, *R*^2^ = 0.991, Fig. 11b) and a low LOD of 4.2 nM. Based on the UV-visible and PL analyses (significant overlap between the absorbance of orange-red Fe–Phen complex and emission of fluorophore), it is proposed that efficient energy transfer between the generated complex (Fe–Phen) and fluorophore is responsible for fluorescence quenching. The specific complexation between Fe^2+^ and Phen rather than the other tested MIs and anions confirmed the good selectivity of this probe. Moreover, the detection of Fe^2+^ in human urine samples exhibited a good recovery in the range of 95.4–102.3%.^166^

*Summary*: The direct detection of Fe^2+^ in aqueous solution has recently witnessed new developments where the turn-off based FL detection of Fe^2+^ using multiple functional groups/dual-heteroatoms containing GQDs achieved the best performance.

### 6.3. Hg^2+^

#### 6.3.1. Undoped and doped-GQDs

The application of a GQDs-based FL sensor in the sensing of Hg^2+^ was first demonstrated in early 2013 (Table S2). Subsequently, undoped GQDs synthesized from various precursors/conditions were further used for the purpose of Hg^2+^ recognition (Tables 3 and S2). For instance, a low LOD of 0.439 nM in the quenching-based detection of Hg^2+^ and analytical applicability in real water samples were achieved from CA precursor-derived GQDs, but the probe is limited to a lower concentration range (Table 3) and its selectivity was only tested with the same concentrations of different MIs/anions as Hg^2+^ in aqueous solution.^232^

**Table 3:** GQDs, modified-GQDs, and GQDs involved with other counterparts for Hg^2+^ sensing application^a^

GQDs-based sensor	Synthesis conditions	Size range/average size^b^ (nm)	QY (%)	Sensing process	LR (µM)	LOD (µM)	Ref.
**Undoped/doped-GQDs**							
GQDs	Pyrolysis (CA, 200 °C, 30 min); dissolved in 10 mg per mL NaOH solution and pH adjusted to 8.0	7–11/—	15.4	FL, turn-off	0.001–0.05, 0.12–2	0.000439	232 i
OH-rich GQDs	Pyrolysis (CA, 200 °C, 25 min); mixed in 1% NaOH solution; centrifugation; dialysis	0.5–3/1.5	50	FL, turn-off	0–20	0.00987	233 i ^,^ j
GQDs	HT (Furfural derived CBDA-2 in NH_4_OH/H_2_O solution, 200 °C, 12 h); dialysis; centrifugation	4–7/—	45	FL, turn-off	10–100^e^	2.5^e^	235
Oxygen-rich N-GQDs	Pyrolysis (CA/l-DOPA, 230 °C, 40 min); dissolved in water and pH adjusted to 7.0; dialysis	4–25/12.5	18	FL, turn-off	0.04–3	0.0086	236 i
N-GQDs	HT (nitrogen-doped GO in water, pH adjusted to 8.0, 200 °C, 12 h); filtration	3–6.4/—	—	FL, ratiometric	0.002–0.2	0.00018	237 j
				COL	0.002–0.2	0.00032	237
N-GQDs@ITO	IR-assisted pyrolysis (CA/urea, 250 °C, 10 min); dispersed in water; centrifugation; drop coated on ITO glass	3–6/4.5	—	EC, CV	0.05–0.25	0.05	117
N-GQDs	Ar/DC microplasma treatment of chitosan in 50 mM CH_3_COOH electrolyte (pH: 4.44), 1 h; purification	4–9/6.39	30.1	FL, turn-off	0.5–60, 60–100	0.0479	163 i
N-GQDs	HT (Spent tea powder in water, 250 °C, 12 h); filtration; dialysis	0.9–2.5/1.6	22	FL, turn-off	0.1–0.5	0.004	149
N,S-GQDs	IR-assisted pyrolysis (CA/urea/ammonia sulfate, 260 °C, 10 min); dispersed in water; centrifugation	1.5–6/3–5	25.5	FL, turn-off	0.01–10^f^	0.05	118
N,S-GQDs	Ar/DC microplasma treatment of chitosan in 35 mM MSA or 0.1 M NH_4_OH aqueous solution, 1 h; purification	2.2–5.9/4.2	1.7	FL, turn-off	1–10, 10–40	0.0074	111
N-GQDs	Ar/DC microplasma treatment of lignin in 35 mM MSA or 0.1 M NH_4_OH aqueous solution, 1 h; purification	2–4.8/3.1	1.0	"	1–20, 20–50	0.0685	
N,S-GQDs	Pyrolysis (CA/Cys, 160 °C, 5 min); dissolved in water; dialysis	—/3.2	—	FL, turn-off	0.5–100^g^	0.33^g^	238 i
					0.1–10^g^^,^^h^	0.048^g^^,^^h^	
B,N-GQDs	Pyrolysis (CA/urea/H_3_BO_3_, 200 °C, 2 h); dissolved in water; centrifugation; filtration; dialysis	1.5–3.5/2	17.16	FL, turn-off	0–4	0.0043	239 i
Mn,N,S-GQDs	Acid oxidation of lignosulfonic acid sodium salt with HNO_3_ under ultrasonication (12 h); filtration; HT (obtained filtrate/2 wt% MnCl_2_ in water, 200 °C, 12 h); filtration; centrifugation	6–13/∼10	31.6	FL, turn-off	0.001–0.1, 0.2–1	0.00056	240 i

**Functionalized GQDs**							
Val–GQDs	Pyrolysis (CA/Val, 200 °C, 2.5 h); mixed in 1 M NaOH; pH adjusted to 7.0; dialysis	1–4/3	28.07	FL, turn-off	0.0008–1	0.0004	241 i
FA–GQDs	HT (maleic acid/FA in water, 180 °C, 2.5 h); dissolved in water and pH adjusted to 7.0; dialysis	2–8/5.2	—	FL, turn-off	0.000005–2	0.0000017	242 i
PEHA,DPA–GQDs	HT (CA/PEHA in water, 200 °C, 1.5 h); HT (obtained mixture/DPA, 200 °C, 2 h); diluted with water; dialysis	1–6/3.16	90.91	FL, turn-off	0.0001–200	0.000046	188 i ^,^ j
DMC–GQDs@GCE	Pyrolysis (CA, 200 °C, 30 min); dissolved in 10 mg per mL NaOH solution & pH adjusted to 7.0; covalently modified with DMC; electro-deposition on GCE	8–14/—	—	EC, DPASV	1 × 10^−6^–15 × 10^−6^	0.26 × 10^−6^	103 i

**GQDs involved with other counterparts**							
TH–GQDs	HT (GO/TH in water, pH adjusted to 8.0, 180 °C, 12 h); filtration; dialysis	2–6/—	42	FL, turn-off	0.0005–0.05	0.00015	243 i
GQDs/TH–ZnPc	Non-covalent conjugation of GQDs with TH-ZnPc	—/20^c^	9.0	FL, turn-off-on	0.0001–0.02	0.00005	
TH–GQDs/TH–ZnPc	Non-covalent conjugation of TH-GQDs with TH-ZnPc	—/31^c^	3.0	"	0.005–0.05	0.0247	
ZnNCs–N-GQDs/Au @GCE	HT (TSC·2H_2_O/urea/Zn-DTT suspension in water, 160 °C, 8 h); solid washed with ethanol and dispersed in water; drop casted on AuNPs-coated GCE	—/5^d^	—	ECL, turn-off	0.00001–1000	3 × 10^−6^	244 i ^,^ k
				COL, turn-off	0.0001–100	33 × 10^−6^	
Am–GQDs/PTH@ GCE	Pyrolysis (CA, 175 °C, 30 min); mixed in aqueous ammonia solution; dialysis; deposited on GCE along with the electro-polymerization of thionine	<10/5^d^	—	EC, CV	1 × 10^−6^–1	0.6 × 10^−6^	245 i ^,^ j
GQDs/Ce-ZnONFs@ GCE	Liquid-phase exfoliation of GO/H_2_O in NaOH/EAA suspension under probe sonication (3 h); filtration; dialysis; *in situ*-immobilized in Ce-ZnONFs; drop-casted on GCE	—	—	EC, DPV	0.1–100	0.267	246
GQDs/Gemini surfactant droplets	Pyrolysis (CA, 150 °C, 12 min); mixed in 5 mg per mL NaOH solution; pH adjusted to 7.0; non-covalently conjugated with Gemini surfactant	2.75–4.75/4.3^d^	—	FL, turn-off	0.1–0.5	0.0305	247 i

a l-DOPA: 3,4-dihydroxy-l-phenylalanine, MSA: methanesulfonic acid, and EAA: ethyl acetoacetate.

b Measured from TEM.

c Size range/average size measured from dynamic light scattering.

d Size range/average size of GQDs used with other counterparts.

e Dynamic concentration range and corresponding LOD.

f LR in ppm.

g LR/LOD in µg L^−1^.

h LR/LOD of paper-based sensor.

i Analytical ability in real water/biological fluid samples.

j Analytical ability in living cells.

k Visual detection capability.

**Fig. 12:**
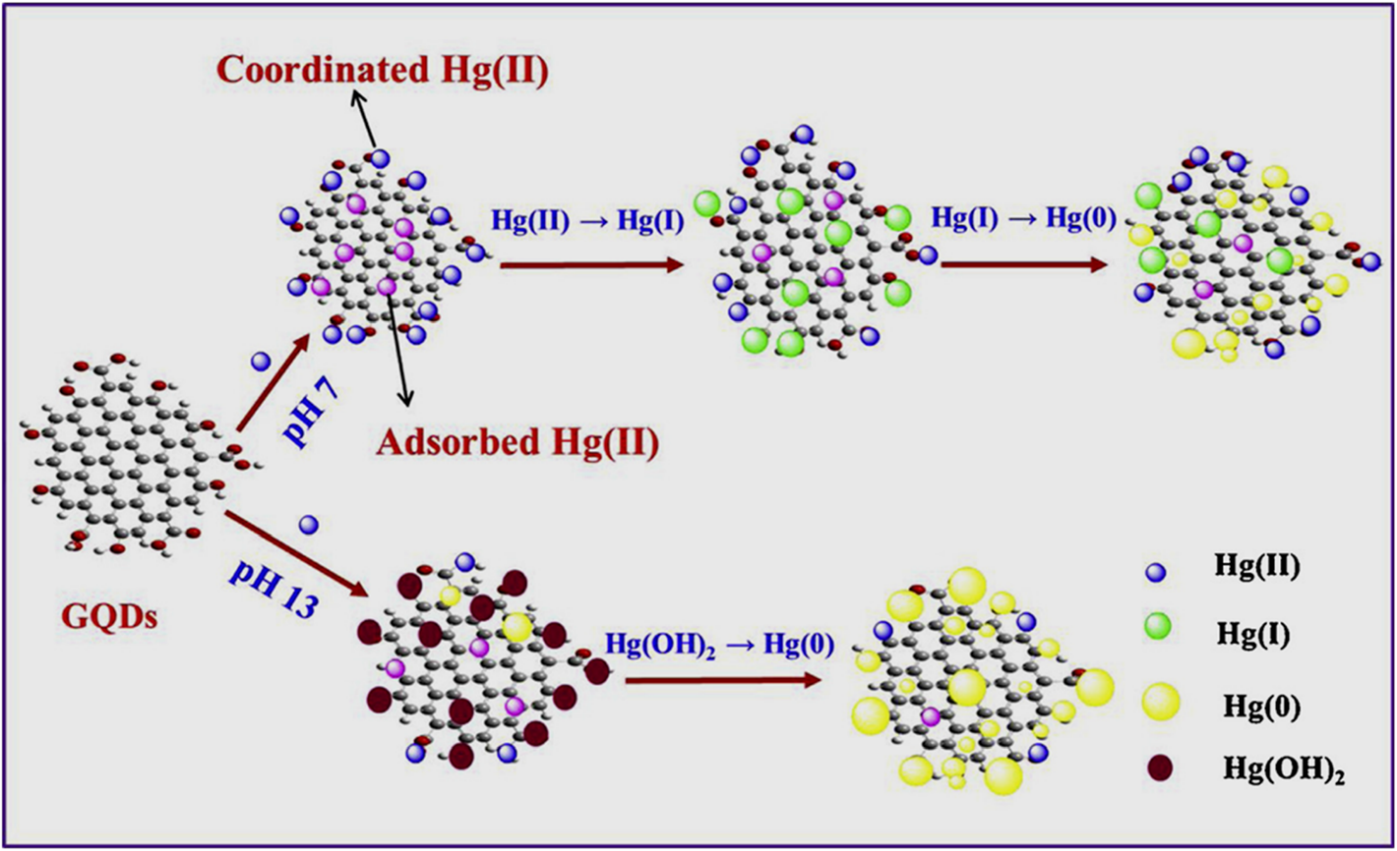
Schematic of the proposed mechanism, where Hg^2+^ is reduced to Hg^+^/Hg at pH 7.0 and Hg at pH 13 by coordination with GQDs. Reprinted from ref. 234, copyright 2020, with permission from Elsevier.

Later, OH-rich GQDs, which were synthesized under similar conditions/precursors, showed a smaller size/high QY compared to previous GQDs (Table 3). As a result, this probe achieved the selective determination of Hg^2+^ in the presence of 500-/1000-times higher concentration of Fe^3+^/other interfering MIs with an extended LR and acceptable LOD (Table 3). The authors proposed that the quenching of the fluorescence of GQDs is due to their formation of a complex with Hg^2+^ and subsequent reduction of Hg^2+^ into Hg^+^ and metallic Hg *via* electron transfer from GQDs to Hg^2+^,^233^ which is validated in another report (Fig. 12) by spectroscopic, microscopic, and DPV results.^234^ The first application of biomass (*Psidium guajava* leaves)-derived red-fluorescent GQDs (maximum intensity *λ*_em_ at 673 nm) for the sensing of Hg^2+^ showed inferior sensitivity, as can be revealed in Table S2; however, the utility of biogenic precursors to achieve high wavelength-emissive GQDs (with the advantage of brightness and minimizing auto fluorescence in biological media) and applicability for HMI detection opens a sustainable and ecofriendly research direction. Subsequently, NIR-fluorescent GQDs (two emission peaks at *λ*_em_: 440 and 850 nm by 310 nm *λ*_ex_) with a fairly high QY (45%) were synthesized from a biomass (furfural)-generated organic compound (*cis*-cyclobutane-1,2-dicarboxylic acid, CBDA-2, Table 3) for the quenching-based detection of Hg^2+^ with an acceptable performance (Table 3); however, selectivity is an issue with this probe (Fe^3+^, Fe^2+^, and Cu^2+^ are potential fluorescence quenchers at the higher concentration of 100 µM). Moreover, NIR-emitting GQDs are beneficial for biological application (tested for bioimaging) due to the minimum scattering of emissive light, small background effect, and high penetration capability in biological tissue.^235^

The relevance of nitrogen-doping in GQDs and use as an Hg^2+^ sensor started with an oxygen-rich N-GQDs FL probe, showing satisfactory sensitivity (Table 3); however, this probe required masking chemicals such as triethanolamine (TEtA) and sodium hexametaphosphate (SHMP) to circumvent the significant quenching arising from Pb^2+^/Cd^2+^/Cu^2+^/nickel ion (Ni^2+^)/Fe^3+^.^236^ Subsequently, various reports confirmed the applicability of N-GQDs in the field of Hg^2+^ sensing (Tables 3 and S2). For example, Peng *et al.*^237^ obtained low LODs (0.18/0.32 nM) for Hg^2+^*via* FL/COL dual-mode sensing methods with linearities on the smaller concentration side (Table 3). The metalloporphyrin (Mn^III^TMPyP; TMPyP = 5,10,15,20-tetrakis(1-methyl-4-pyridinio)porphyrin) formation mechanism (accelerated by Hg^2+^ and N-GQDs) occurred *via* the ability of the large Hg^2+^ to deform the TMPyP nucleus, followed by the backside incorporation of small Mn^2+^ into it through N-GQDs as a carrier. As a result, the fluorescence signal of N-GQDs and TMPyP was enhanced and suppressed with IFE-based mechanism (ratiometric design), while original (422 nm)/red-shifted (462 nm) absorbance of TMPyP/Mn^III^TMPyP decreased/increased (basis for COL method), respectively. Moreover, this strategy could be successfully applied for the ratiometric monitoring of intracellular Hg^2+^ in A549 cells (human lung cancer cells). An N-GQDs-modified indium tin oxide (ITO) glass electrode was utilized for the EC detection of Hg^2+^ with a satisfactory result (Table 3); however, the coverage of intermediate Hg states and subsequent metallic Hg clustering on N-GQDs@ITO limited the sensitive detection of Hg^2+^ at low concentrations.^117^ The allowable LOD of 47.9 nM along with broad LR up to 100 µM in Hg^2+^ sensing by N-GQDs (synthesized from green precursor (chitosan) using Ar/DC plasma treatment) is noticeable (Table 3) but this probe is also sensitive to Cu^2+^, and thus requires a masking/chelating ligand to avoid its interferance.^163^ Recently, the gram-scale synthesis of N-GQDs (QY: 22%, production of 1.3 g in one batch) was shown to be possible *via* the HT carbonization of spent tea powder without involving an extra nitrogen source. The amino- and nitro-rich N-GQDs (nitrogen content: 8.1%) showed appropriate binding affinity with Hg^2+^ (soft acid-base interaction) to promote non-radiative processes and fluorescence suppression through DQE. Consequently, this biogenic platform achieved the trace-level FL detection of Hg^2+^ (LOD: 4 nM) but with the limitation of probing in a narrow concentration range (Table 3) and some perturbations from Pb^2+^/Cd^2+^.^149^

The Hg^2+^ sensing ability of dual-heteroatom doped-GQDs, such as N,S-GQDs, B,N-GQDs, and N,P-GQDs has also been reported in the literature (Tables 3 and S2). Among them, N,S-GQDs exhibited a sub-nanomolar level detection possibility in a low concentration window for Hg^2+^ (Table S2). The high sensitivity of N,S-GQDs (*K*_SV_: 0.22 l mg^−1^) rather than N-GQDs (*K*_SV_: 0.052 l mg^−1^) in the detection of Hg^2+^ is attributed to the presence of C–SO_*x*_–C sulphone bridges and other sulfur-doping configurations (C–S–C, C–SH), apart from the nitrogen/oxygen-containing functional groups in N,S-GQDs. As a result, the adjustment of the local electronic state, Fermi level, and creation of new energy states (from defect sites) in N,S-GQDs efficiently promoted their affinity towards Hg^2+^.^118^

**Fig. 13:**
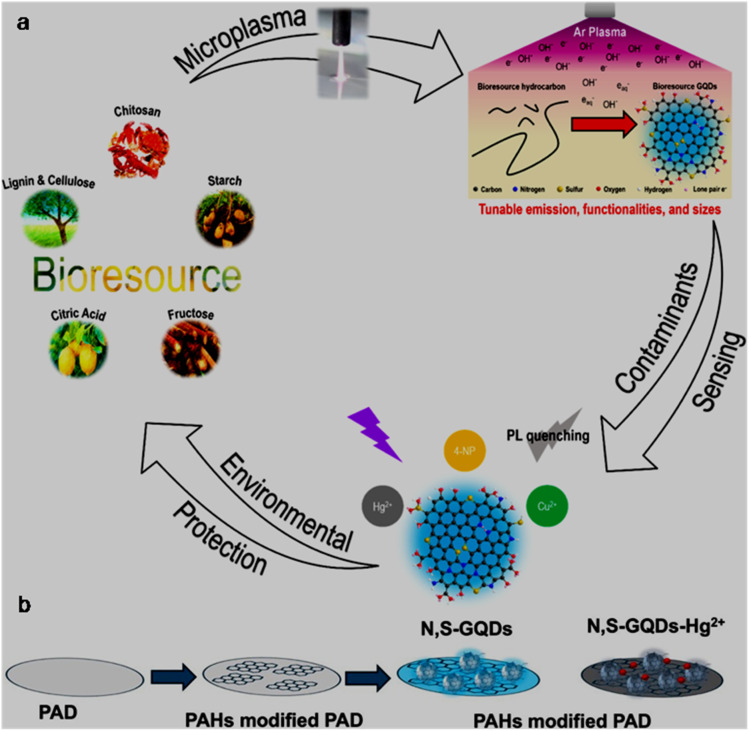
(a) Transformation of various bio-resources into heteroatom doped-GQDs through Ar microplasma treatment for the fluorescence quenching-based detection of environmental contaminants (Hg^2+^, Cu^2+^, and 4-NP). Reprinted (adapted) with permission from ref. 111, copyright 2022, the American Chemical Society. (b) Construction of paper-based device for the detection of Hg^2+^. Reprinted from ref. 238, copyright 2025, with permission from Elsevier.

Kurniawan *et al.*^111^ reported an energy-efficient DC microplasma-based method for the sustainable conversion of bio-resources (CA and saccharides such as fructose, chitosan, lignin, cellulose, and starch) into heteroatom doped-GQDs or undoped GQDs for the probing of environmental contaminants, including Hg^2+^, Cu^2+^, and 4-nitrophenol (4-NP) (Fig. 13a). Among them, chitosan-derived N,S-GQDs (nitrogen/sulfur content: 7.3/0.9%) showed notable sensitivity (LOD: 7.4 nM) in the turn-off based detection of Hg^2+^, while N-GQDs derived from lignin were found to be less sensitive for Hg^2+^ (Table 3). A recent report on N,S-GQDs (green-fluorescent) demonstrated that they not only selectively detected Hg^2+^ in the solution phase but also could be applied to construct a paper-based analytical device (PAD), and furthermore exhibited relatively high sensitivity (Table 3). The simple and cost effective construction of a biodegradable sensing device *via* the modification of PAD with polycyclic aromatic hydrocarbons (PAHs), followed the integration with N,S-GQDs and coordination interaction with Hg^2+^ is shown in Fig. 13b. The PAHs-modified PAD effectively adhered to the doped-GODs in well-dispersed manner to improve their sensitivity. This report also confirmed the PET-based quenching mechanism during the Hg^2+^ detection process and solution/paper-based detection potential in spiked-water/fermented fish samples.^238^ The incorporation of a small amount of boron (0.59%) in addition to nitrogen (9.61%) in B,N-GQDs also enabled the selective and sensitive recognition of Hg^2+^; however, it is not as good as the N,S-GQDs probe for Hg^2+^ (Tables 3 and S2).^239^

**Fig. 14:**
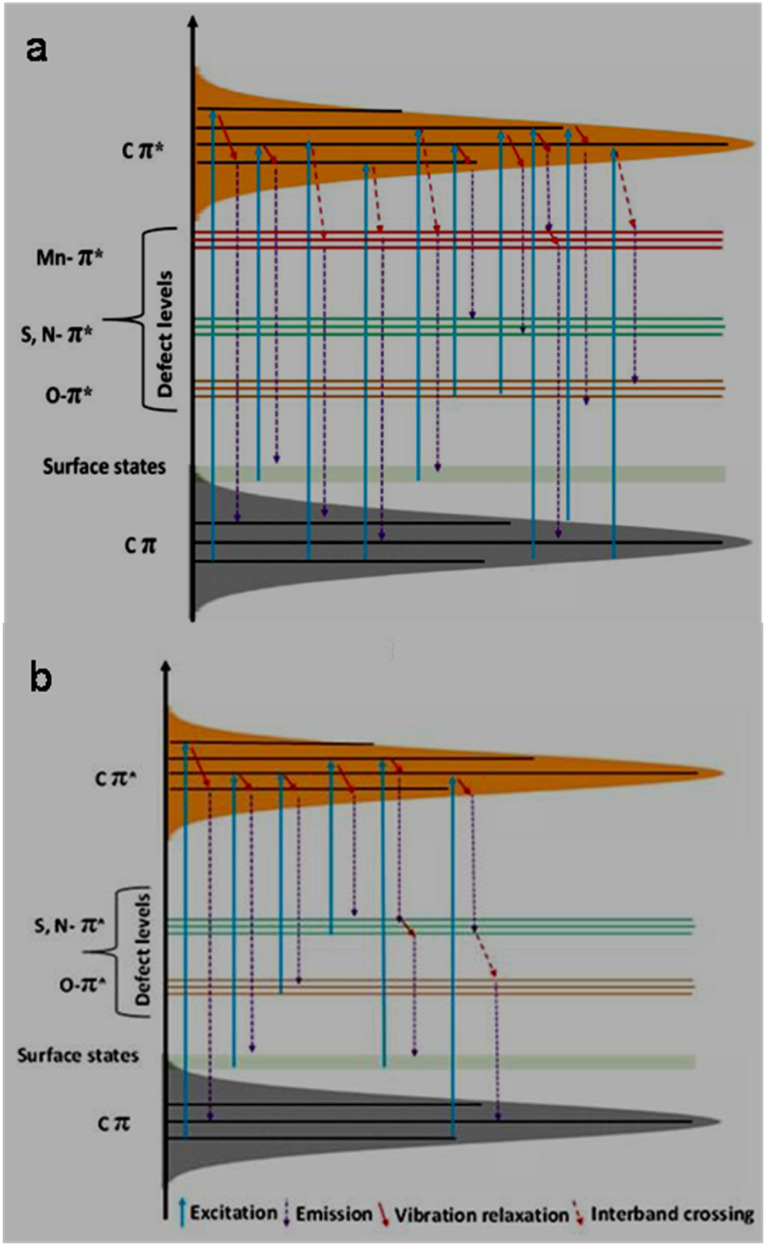
Band structures of Mn,N,S-GQDs (a) and N,S-GQDs (b), showing various possible transitions during their excitation and emission processes. Reprinted (adapted) with permission from ref. 240, copyright 2020, the American Chemical Society.

It is observed that Mn^2+^-incorporated single-/dual-heteroatom doped-GQDs can also be selective for fluorescence quenching in the presence of Hg^2+^ (Tables 3 and S2). For example, the QY of *in situ* doped N,S-GQDs (23%, synthesized from lignosulfonate biomass, Table 3) further improved up to 31.6% by incorporating Mn^2+^-dopant (0.24 at%) in their structure. The collective effect of multi-element doping and abundant defect sites created new energy/edge states between π and π* of carbon in the Mn,N,S-GQDs (Fig. 14a) rather than N,S-GQDs (Fig. 14b) for the enhancement of their PL property. As a result, Mn,N,S-GQDs showed better applicability towards the sub-nanomolar sensitive detection of Hg^2+^ (LOD: 0.56 nM) in comparison to N,S-GQDs (LOD: 7 nM) and reusability after recovering their quenched PL with EDTA.^240^

#### 6.3.2. Functionalized GQDs

Functionalized GQDs are also credited for the recognition of Hg^2+^ even at the sub-nanomolar level of sensitivity *via* the FL or EC method (Tables 3 and S2). For example, valine (Val)-functionalized GQDs (Val–GQDs) showed a 14-fold higher sensitive response (88.2% quenching) than bare GQDs (6.2% quenching) in the presence of 2 µM Hg^2+^ due to their functional groups (nitrogen- and oxygen-containing)-assisted strong interactions/complexation with Hg^2+^, and furthermore good sensitivity in the lower concentration side (Table 3).^241^ Li *et al.*^242^ designed FA–GQDs (FA groups covalently attached at the edges of GQDs; a large amount of graphitic and amine nitrogen compared to pyridinic nitrogen), which showed an LOD of 0.0017 nM again in the lower concentration range (LR: 0.005–2 µM), surpassing the LODs of all FL turn-off-based Hg^2+^ detection processes (Tables 3 and S2). Subsequently, dual-functionalized PEHA,DPA–GQDs (QY: 90.91%; amine groups dominated along with some sulfur element) were employed in the FL-based highly sensitive detection of Hg^2+^ (LR/LOD: 0.0001–200 µM/0.046 nM) without significant interference from other ions. The predominant nitrogen/oxygen-containing functionalities at the edges constitute a favourable configuration on the probe to effectively/selectively coordinate with Hg^2+^ and result fluorescence quenching.^188^

Hg^2+^ could also be sensed by the EC method using functionalized GQDs. For instance, the specific EC detection of trace-level Hg^2+^ with a DMC–GQDs-modified GCE electrode (DMC–GQDs@GCE) using differential pulse ASV (DPASV) measurement showed a minimum detection limit of 0.26 pM from the linear calibration plot. However, a high degree of sensitivity was achieved in this report *via* the pre-concentration and pre-reduction of Hg^2+^ at the active electrode surface. Here, the thiol functional groups in the DMC ligand and high surface area of the functionalized GQDs provided distinct and abundant complexation sites for Hg^2+^ during the EC operation. Besides its satisfactory repeatability, reproducibility, and stability, DMC–GQDs@GCE is also applicable for measuring the Hg^2+^ concentrations in tap and river water.^103^

#### 6.3.3. GQDs involved with other counterparts

The construction of sensor probes *via* the utilization of GQDs/modified-GQDs with other functional moieties and their application for the sensing of Hg^2+^ have also been reported (Tables 3 and S2). For instance, a turn-off-on based sensing strategy was demonstrated for the low-level detection of Hg^2+^ using a conjugate system containing GQDs or thymine (TH)-functionalized GQDs (TH–GQDs) and TH-functionalized zinc phthalocyanin (ZnPc) (TH–ZnPc). Fluorescence quenching occurred due to the π–π interaction between GQDs or TH–GQDs and TH–ZnPc (through Förster resonance energy transfer (FRET)), which again were regenerated after the addition of Hg^2+^ due to the formation of TH–Hg^2+^–TH base pairs and interaction inhibition. It was observed that the non-covalent conjugation of bare GQDs with TH–ZnPc (GQDs/TH–ZnPc) possessed a significantly higher sensitivity (LOD: 0.05 nM) compared to TH–GQDs-conjugated TH–ZnPc (TH–GQDs/TH–ZnPc) or TH–GQDs, highlighting a turn-off-on approach for better sensitivity than the turn-off route (Table 3).^243^ The ECL detection of Hg^2+^ with high sensitivity is possible with systems containing GQDs, along with Hg^2+^-specific DNA aptamers and other components (Table S2); however, the construction of this type of platform is very complex, expensive, and requires precise experimental conditions.

**Fig. 15:**
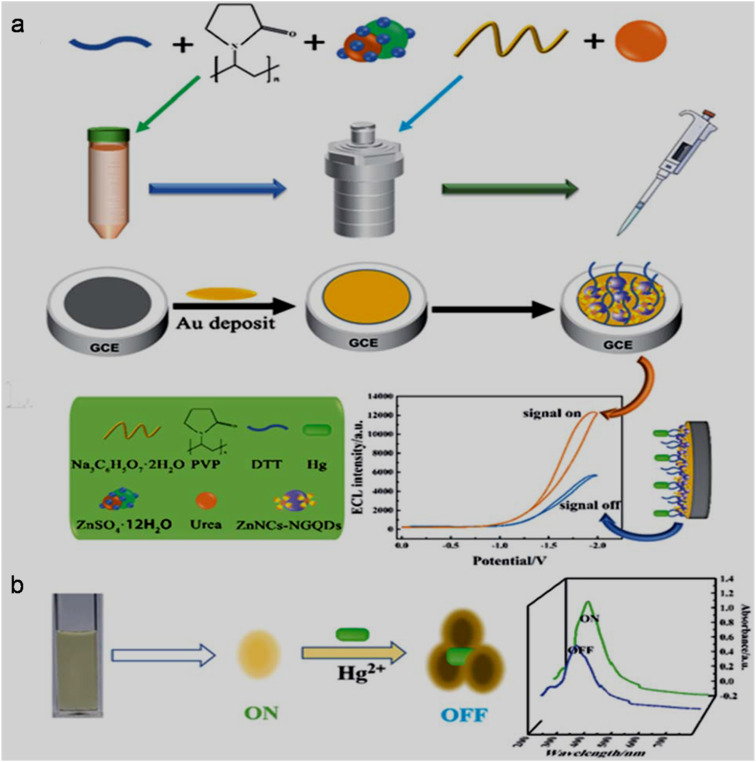
(a) Synthesis of ZnNCs–N-GQDs through HT method, and steps involved in the fabrication of ECL sensor along with the on/off signal response. (b) On/off mechanism in the absence/presence of Hg^2+^ and corresponding UV-visible spectra. Reproduced/adapted from ref. 244 with permission from The Royal Society of Chemistry, 2022.

Therefore, Wu *et al.*^244^ developed a simple zinc dithiothreitol (Zn-DTT) nanocrystals (NCs)-connected N-GQDs composite luminophore (ZnNCs–N-GQDs) for the sensitive ECL detection of Hg^2+^. The HT-based synthesis of ZnNCs–N-GQDs, their deposition over an Au-coated GCE, and ECL signal response are shown in Fig. 15a. The chelating ability of Hg^2+^ with the free S–H groups of ZnNCs turned the ECL signal off, which is validated by the decrease in absorbance after quenching (Fig. 15b). Consequently, this sensor showed an acceptable performance for the detection of Hg^2+^ (wide LR/low LOD: 0.01–1 000 000 nM/3 pM), which is much better than GQDs involved aptamer sensors (Table S2). This sensor probe also showed COL detection possibility for Hg^2+^ with reasonably good sensitivity (Table 3). Moreover, the visual detection capability (brown-coloured probe solution turned into a colourless supernatant with brown precipitate) and monitoring of Hg^2+^ in tap/lake water samples (recoveries: 96–105%) are practical attributes of this probe.

The EC detection of Hg^2+^ with high sensitivity (LOD: 0.6 pM) in the low concentration range (LR: 1 pM–1 µM) using an Am–GQDs/poly(thionine) (Am–GQDs/PTH) nanocomposite is noticeable. However, the modified electrode is very specific to PTH deposition cycles and the EC process involved complicated steps, where the Cu-catalyzed Fenton-like reaction first increased the cathodic peak current. Then, Cu^2+^ is consumed by TU (CuTU_2_^+^ complex formation) to inhibit Fenton-like reaction/EC response, followed by the displacement of Cu^+^ from the complex in the presence of Hg^2+^ to regain the current response and readout to quantify Hg^2+^.^245^ Later, Qi *et al.*^246^ applied a GQDs/Ce–ZnONFs (NFs = nanofibers) hybrid as an electrode material for the DPV-based EC detection of Hg^2+^ in a wide LR of 0.1–100 µM (LOD: 267 nM). Although the redox process during the sensing operation is based on Ce/Zn, the functional groups of GQDs in the hybrid material improve the adsorption of Hg^2+^ on the electrode surface by creating sufficient oxygen vacancies/affinity and facilitate redox reactions for a selective and sensitive EC response.

**Fig. 16:**
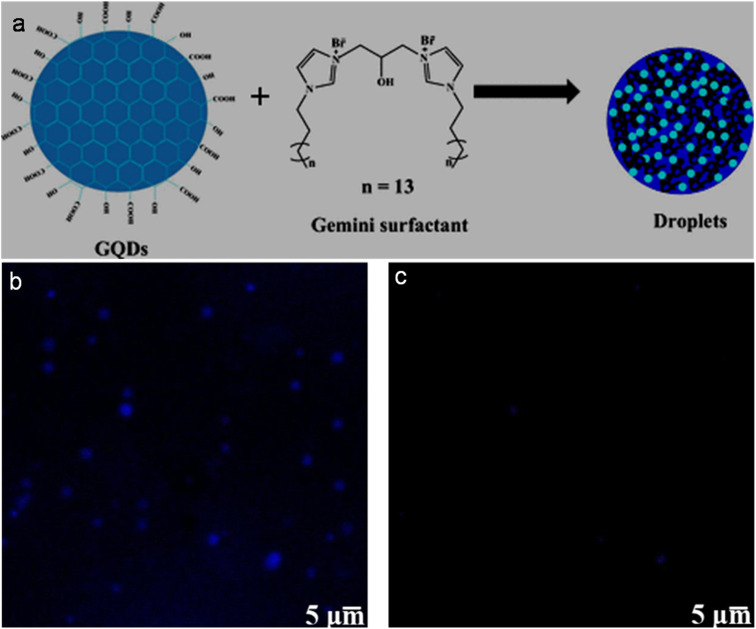
(a) Schematic of the formation of blue-luminescent droplets *via* the self-assembly of GQDs and Gemini surfactant. Confocal microscopic images of droplets, showing blue fluorescence in the absence of Hg^2+^ (b) and quenched fluorescence in the presence of 10 µM Hg^2+^ (c). Reprinted (adapted) with permission from ref. 247, copyright 2025, the American Chemical Society.

Recently, a positively charged Gemini surfactant (zeta potential: + 55.9 mV) and negatively charged GQDs (zeta potential: −25.2 mV) were self-assembled in aqueous medium *via* electrostatic interaction to construct blue-fluorescent droplets (Fig. 16a). The droplet nanoprobe was found to be highly selective towards Hg^2+^ and exhibited a fluorescence quenching response on the progressive addition of Hg^2+^ with good sensitivities both in standard aqueous solution (LOD: 30.5 nM) and in spiked-tap water (LOD: 75.2 nM). The effective binding affinity between the luminescent droplets and Hg^2+^ was justified by the high *K*_SV_ constant value (4.633 × 10^6^ M^−1^), and furthermore by analyzing the confocal microscopic images, leading to diminished inherent blue fluorescence from the droplets (Fig. 16b) in the presence of 10 µM Hg^2+^ (Fig. 16c).^247^

*Summary*: The achievement of high selectivity/sensitivity with ratiometric design (N-GQDs/Hg^2+^-mediated metalloporphyrin formation), N-GQDs/N,S-GQDs (bio-precursor (chitosan) and energy-efficient microplasma derived), and Mn^2+^-synergised N,S-GQDs is highlighted in the execution of doped-GQDs for the identification of Hg^2+^. B,N-GQDs may also be potential candidates for the FL quenching-based sensitive detection of Hg^2+^. Moreover, the highly sensitive detection of Hg^2+^ using functionalized GQDs (specifically, PEHA and DPA dual-functionalized GQDs) *via* the FL method and achievement of picomolar-level sensitivity *via* the EC method (using DMC–GQDs) are noticeable. It is also observed that the compositing strategy of GQDs/modified-GQDs with other counterparts can be one of the suitable strategies by which Hg^2+^ can be detected at trace levels *via* ECL and EC approaches. Specifically, the picomolar-level ECL detection capability in a wide concentration range using a ZnNCs–N-GQDs modified electrode is recognizable.

### 6.4. Cu^2+^

#### 6.4.1. Functionalized GQDs

The first report of Cu^2+^ sensing was achieved with functionalized GQDs (Am–GQDs; Am group introduced after the synthesis of GQDs, LOD: 6.9 nM, Table S3). Later, better sensitivity (LOD: 5.6 nM) was achieved for Cu^2+^ using Am–GQDs (Am group introduced during the synthesis of GQDs *via* the bottom-up approach, Table 4), indicating the advantage of incorporating Am groups during the growth stage of GQDs. However, the probing of Cu^2+^ by Am–GQDs followed linearity in a narrow concentration range.^248^

**Table 4:** GQDs, modified-GQDs, and GQDs involved with other counterparts for Cu^2+^ sensing application^a^

GQDs-based sensor	Synthesis conditions	Size range/average size^b^ (nm)	QY (%)	Sensing process	LR (µM)	LOD (µM)	Ref.
**Functionalized GQDs**							
Am–GQDs	HT (glucose/NH_3_/H_2_O_2_ in water, 150 °C, 4 h); filtration; dialysis	1–7/4.34	32.8	FL, turn-off	0.01–0.1	0.0056	248
Am–GQDs	Purchased from Suzhou Carbon-rich Graphene Technology Co. Ltd	10–35/21.3	—	FL, turn-off	0–80	1	249

**Undoped/doped-GQDs**							
sl-GQDs	Acid oxidation of CNOs with 5 M HNO_3_ under reflux (95 °C, 4 h); pH adjusted to 7.0; dialysis; portion left in dialysis bag	2–6/3.1		FL, turn-off	0.02–0.2	0.02	250
GQDs@GCE	Chemical oxidation of GO with 30 wt% H_2_O_2_/O_3_ under ultrasonication (3 h); reaction terminated by N_2_ purging (15 min); drop-casted on GCE	2–13/—	—	EC, DPASV	50–650^e^	0.0003	251
GQDs/DNPC	Pyrolysis (CA, 200 °C); mixed in 10 mg per mL NaOH solution and pH adjusted to 7.0	2.5–5.5/—	—	FL, turn-off-on	0.01–10	0.0045	252 f
GQDs	Ar/DC microplasma treatment of starch in 0.1 M NaOH aqueous solution, 1 h; filtration	1.5–7/3.6	21.1	FL, turn-off	0.5–25	0.5	253 f
GQDs	Pyrolysis (CA, 200 °C, 30 min); mixed in NaOH solution and pH adjusted to 8.0; centrifugation	—/2.2	55	FL, turn-off	0.01–0.5	0.0025	112
GQDs	Pyrolysis (CA, 200 °C, 45 min); mixed in 10 mg per mL NaOH solution; filtered and pH adjusted to 7.0; aged for 3 days at 4 °C and mixed in ethanol; centrifugation	1.2–3.6/2.15	—	FL, turn-off	0.04–2	0.04	254 f ^,^ g
N-GQDs	Ar/DC microplasma treatment of chitosan in 35 mM HNO_3_ electrolyte (pH: 2.69), 1 h; purification	2.5–6.5/4.36	1.74	FL, turn-off	0.5–10, 10–100	0.1465	163 f
N-GQDs/Paper	Pyrolysis (CA/urea, 200 °C, 15 min); mixed in 10 mg per mL NaOH solution and pH adjusted to 7.0; drop-coated on paper strip	1.9–3.1/2.46	—	ECL, turn-on	0.01–1000	0.18	255 f
N-GQDs	ST (Styrofoam in acetone/NH_3_/H_2_O_2_ mixture, 225 °C, 1.5 h)	5–7/—	187^d^	COL, turn-on	0–1 × 10^5^	—	256 f ^,^ g
N-GQDs	HT (nitronaphthalene/*p*-aminobenzoic acid in water, 180 °C, 12 h); filtration	1–5/2.1	29.75	FL, turn-off	0–10	—	257 h

**GQDs involved with other counterparts**							
Fe_3_O_4_@Chitosan–GQDs	Pyrolysis (CA, 200 °C, 5 min); mixed in 0.25 M NaOH solution; immobilized on Fe_3_O_4_ NPs/Chitosan	—/9^c^	—	ICP-OES	0.05–1500^e^	0.015^e^	258 f
Am–GQDs/SeNPs	Pyrolysis (CA, 175 °C, 30 min); ultrasonically mixed in aqueous NH_3_; dialysis	—/5^c^	—	FL, turn-off-on	0.001–10	0.0004	259 f ^,^ h
CdS/AuNPs/GQDs @ITO	GQDs purchased from ACS material, USA; *in situ* incorporated in AuNPs and CdS nanorods; casted on ITO glass	<5/—c	—	PEC, LSV	0.0001–0.29	0.00227	260
GQDs//g-C_3_N_4_NSs/MWCNTs@GCE	HT (CA/TU in water, 180 °C, 6 h); dispersed in water; centrifugation; dialysis; g-C_3_N_4_NSs and MWCNTs mixture is drop-coated on GCE	—	—	ECL, ratiometric	0.0005–1	0.00037	261 f
GQDs/TPPS (1 : 9) @SPCE	MW (GA/triethylenetetramine in water, 300 W, 225 °C, 5 min); non-covalently modified with TPPS	0.5–6.5/—c	—	EC, SWV	0–6, 6–13	0.172	262 f

a LSV: linear sweep voltammetry.

b Measured from TEM.

c Size range/average size of GQDs used with other counterparts.

d QY with respect to methylene blue dye.

e LR/LOD in µg L^−1^.

f Analytical ability in real water/vegetable/Thai recipe/serum samples.

g Visual sensing capability.

h Analytical ability in mouse cells/living cells/tumor cells.

**Fig. 17:**
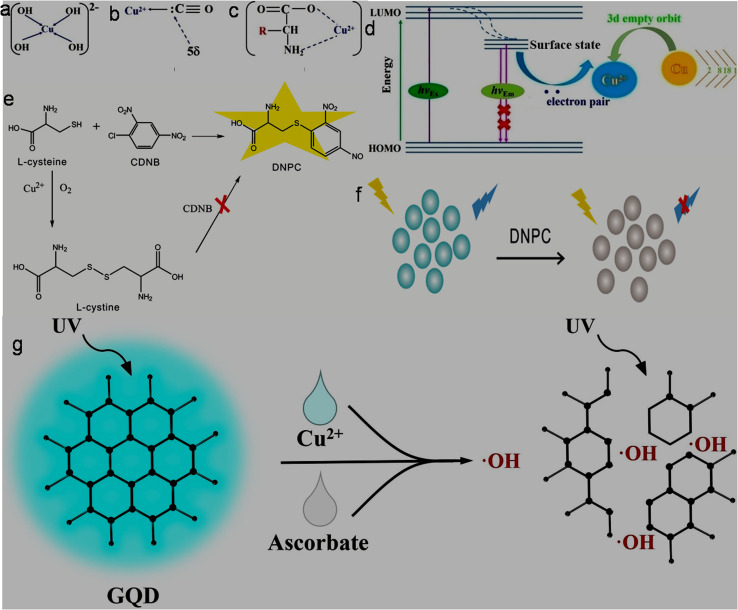
Coordination interactions of Cu^2+^ with hydroxyl (a), carbonyl (b), and amino (c) functionalities of Am–GQDs. (d) Schematic illustration of the electron transfer-based quenching process in the detection of Cu^2+^. Reprinted from ref. 249, copyright 2024, with permission from Elsevier. (e) Formation of DNPC and Cu^2+^-induced catalytic transformation of Cys to l-cystine for the inhibition of DNPC. (f) Fluorescence quenching of GQDs in the presence of DNPC. Reprinted from ref. 252, copyright 2019, with permission from Elsevier. (g) Schematic of the Cu^2+^ sensing strategy *via* ˙OH radical-induced disruption of the GQDs structure. Reprinted from ref. 254, copyright 2024, with permission from Elsevier.

Recently, Ren *et al.*^249^ again applied Am–GQDs in the sensing of Cu^2+^, which showed a broad LR of 0–80 µM in the quenching process. Fig. 17a–c show the binding affinity of Cu^2+^ with –OH (populated with electron pair), 5σ molecular orbital of C=O, and –NH_2_ (having electron pair) surface/edge groups of Am–GQDs, respectively. As a result, the photo-excited electrons in Am–GQDs are transferred to the empty 3d orbital of Cu^2+^ and inhibit radiative recombination for fluorescence quenching (Fig. 17d). However, this report did not test the quenching effect of the probe in the presence of other interfering ionic species and its applicability towards real sample analyses.

#### 6.4.2. Undoped- and doped-GQDs

The involvement of bare GQDs in the detection of Cu^2+^ can be revealed in Tables 4 and S3. For instance, blue-emitting sl-GQDs (remained in dialysis bag) with a high content of oxygenated defects are selective towards Cu^2+^ due to their structural, compositional, and energetic difference in comparison to multilayered GQDs (extracted from dialysis bag, low oxygen content, UV-emitting, and selective for Fe^3+^). The presence of more surface defects in sl-GQDs can effectively attract Cu^2+^, and furthermore the occurrence of charge transfer (from sl-GQDs to Cu^2+^) facilitated fluorescence quenching.^250^ Wen *et al.*^251^ first used GQDs as an electrode material in the EC detection of Cu^2+^ (LOD: 0.3 nM). The authors proposed that abundant –COOH groups on the GQDs are beneficial to create more negative sites for Cu^2+^ adsorption and redox reactions in the detection process. However, the EC response of GQDs@GCE with other interfering ions was not investigated. Ding *et al.*^252^ developed a turn-off-on based sensing platform for Cu^2+^. They observed that 2,4-dinitrophenylcysteine (DNPC, a reaction product of Cys and 1-chloro-2,4-dinitrobenzene (CDNB), Fig. 17e) effectively quenched the fluorescence signal of GQDs (Fig. 17f). Once Cu^2+^ is added, Cys is catalytically oxidized into l-cystine, and therefore the fluorescence recovery due to the suppression of DNPC formation (Fig. 17e) facilitated the identification of Cu^2+^ with satisfactory sensitivity (LOD: 4.5 nM) and practical applicability in real water samples. However, the sensing protocol functioned under specific conditions and is time consuming (Cu^2+^-catalyzed oxidation of Cys occurs at 90 °C, incubation time of Cys: 30 min, reaction time between Cys and CDNB: 50 min). The charge transfer-based fluorescence quenching of the GQDs in the presence of Cu^2+^ was revealed through DFT/time-dependent DFT studies. It is inferred that the photo-excited electrons from GQDs are mainly located on the Cu side, inhibiting the usual relaxation process, and therefore the forbidden-emission activity of the excited electrons is responsible for the quenching phenomenon. Additionally, the XPS and UV-visible results suggested the formation of a Cu–GQDs complex and charge transfer between them as the factors responsible for the quenching operation. It was also observed that the Cu^2+^ detection sensitivity of microplasma-synthesized GQDs is much better than that of HT-synthesized GQDs (precursor for both synthesis routes: starch), which is attributed to their high crystallinity (less defects) and facile electron transfer characteristics.^253^

Recently, Guo *et al.*^254^ realized the dual-mode detection of Cu^2+^ using undoped GQDs. Although the FL-based LOD of Cu^2+^ (40 nM) is larger than that in some previous reports (Tables 4 and S3), this probe could visually detect Cu^2+^ (minimum dose: 10 µM) under 365 nm light irradiation through the fluorescence quenching effect. The blue-emission of GQDs (EIPL characteristic, *λ*_em_: 480 nm) is effectively suppressed by the ˙OH radical-driven (produced from the Fenton-like reaction between Cu^2+^ and ascorbate) disruption of the structure of GQDs (Fig. 17g) to monitor the Cu^2+^ concentration in the solution. However, this probe was found to behave irreversibly (cannot be reused/regenerated after the process) due to its structural disintegration, and also suffered from interference from Fe^2+^/Fe^3+^ (required masking agent: TEtA/SHMP). Finally, this probe is applicable to quantify Cu^2+^ in spiked-lake water samples with 84.4–108% recovery and visibility under UV light irradiation.

N-GQDs derived from different precursors and experimental processes are exclusively used as turn-off type FL, COL, and ECL probes to sense Cu^2+^ (Tables 4 and S3). For example, N-GQDs synthesized *via* the DC microplasma irradiation of a chitosan solution under atmospheric pressure showed a satisfactory performance in the detection of Cu^2+^ (Table 4).^163^ Zhu *et al.*^255^ demonstrated a paper-based ECL sensor using N-GQDs (fabricated *via* the screen-printing technique) to trace the switch-on ECL signal with an increase in the concentration of Cu^2+^ (Table 4). Nitrogen-doping in the N-GQDs facilitated electron transfer with the co-reactant to generate a stable/strong ECL response. Furthermore, electron/free radical transfer is accelerated after the addition of Cu^2+^ to enhance the ECL signal, and subsequently Cu^2+^ quantification.

**Fig. 18:**
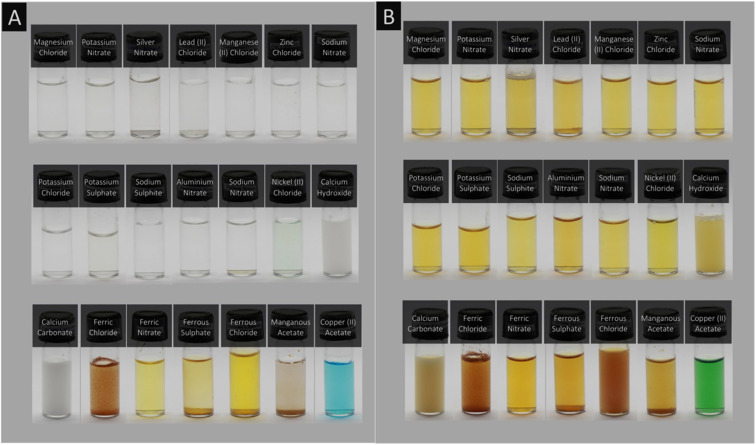
0.1 M solutions of 20 different metal salts (A) and the corresponding changes in their colour after the addition of N-GQDs (B). Reprinted (adapted) with permission from ref. 256, copyright 2024, the American Chemical Society.

In a recent observation, the utility of waste (Styrofoam) for the synthesis of N-GQDs and their further applicability for the selective quantification of Cu^2+^ by simply observing changes in their colour (blue to green among 20 tested MIs, Fig. 18A and B) without the assistance of UV light are noticeable. The variations in electronic states or surface plasmon arising from the N-GQDs after their interaction with Cu^2+^ are likely the reason for the sharp and visual colour changes. The analyses of the images in gray-mode by varying the Cu^2+^ concentration provided a calibration plot to quantify Cu^2+^ from unknown solutions, and the probe also showed its functionality in the analysis of real river water.^256^ Another recently synthesized N-GQDs probe with yellow-emissive characteristics (*λ*_ex_/*λ*_em_: 494/540 nm) showed fluorescence quenching-driven detection capability for Cu^2+^ (Table 4). This probe is nearly recyclable with an EDTA chelating agent; however, the LOD was not reported in this study. Moreover, the applicability of this probe is extended to detect Cu^2+^ in spiked-pesticide/dye wastewater as well as in biological mouse cells.^257^

#### 6.4.3. GQDs involved with other counterparts

There are various mixture/composite/heterostructure systems in which GQDs/modified-GQDs play a crucial role to detect Cu^2+^ at different levels of sensitivity (Tables 4 and S3). For instance, the trace-level detection of Cu^2+^ in Thai food samples was demonstrated *via* traditional ICP-OES (LOD: 0.015 µg l^−1^) using a magnetic nanocomposite (Fe_3_O_4_@chitosan–GQDs) as an adsorbent material for Cu^2+^ pre-concentration. However, although the magnetic probe is reusable up to seven cycles, the tedious adsorption–desorption steps for Cu^2+^ before injecting into costly the ICP-OES setup limits this sensing as a facile approach.^258^ PET between Am–GQDs and SeNPs resulted in fluorescence quenching, which again turned-on in the presence of Cu^2+^–ascorbic acid (AA) to achieve a low LOD (0.4 nM). However, the optimal fluorescence quenching-recovery was obtained after mixing SeNPs, AA, and Cu^2+^ with Am–GQDs for 2.5 h, showing a time-consuming sensing process. Moreover, the sensor platform not only detected Cu^2+^ in tap and lake water samples (recoveries: 98.7–103%) but also in HeLa and cisplatin-resistant tumor cells (less uptake of Cu^2+^ in the resistant tumor cells compared to tumor (HeLa) cells due to the down regulation of human copper transporter-1 expression).^259^ The visible light-induced photoelectrochemical (PEC) detection of Cu^2+^ using a ternary CdS/AuNPs/GQDs@ITO electrode system is also beneficial for good sensitivity (Table 4). It is observed that the loading of GQDs in CdS/AuNPs is advantageous to significantly improve the photocurrent density of ternary composites (2.3-fold) due to their high charge mobility. Additionally, the favourable alignment of the energy levels between CdS/AuNPs and GQDs inhibited charge recombination to promote the PEC performance. However, although the probe is very selective for Cu^2+^ over other tested MIs, it exhibited an interference feature with Fe^2+^.^260^ After that, Liu *et al.*^261^ developed a dual-potential ratiometric ECL platform using GQDs and g-C_3_N_4_ nanosheets (g-C_3_N_4_NSs)/MWCNTs luminophores for the selective detection of Cu^2+^ with improved accuracy and sensitivity. It was observed that the strong cathodic ECL from g-C_3_N_4_NSs/MWCNTs@GCE was progressively suppressed in the presence of Cu^2+^, while anodic ECL from the solution-phase GQDs remained intact. As a result, the cathodic/anodic ECL intensity ratio responded for the very sensitive detection of Cu^2+^ (LOD: 0.37 nM) in comparison to the non-ratiometric single probe without GQDs (LOD: 45 nM), justifying the importance of GQDs to improve the sensitivity in the detection process. The low RSDs (3%) for the measurement of Cu^2+^ from 5 independent electrodes in the ratiometric design rather than the non-ratiometric approach (RSDs: 7.2%) further justified the positive effect of ratiometric sensing. However, this system suffered from Fe^3+^ interference, which could be overcome by the chelation strategy (SHMP). The satisfactory detection of Cu^2+^ in wastewater samples is also possible using the constructed sensor. The applicability of a composite system (GQDs/TPPS (1 : 9); TPPS = 5,10,15,20-tetrakis(4-sulfonatophenyl)porphyrin; 1 : 9 implies mass ratio of GQDs and TPPS) was demonstrated recently to detect Cu^2+^*via* the EC method; however, its sensitivity was found to be inferior compared to previously reported EC detection results (Table 4). Here, the nitrogen of TPPS provided coordination sites for Cu^2+^ binding and its subsequent detection.^262^

*Summary*: We can say that Am functional groups on the surface of GQDs are advantageous for the selective/sensitive detection of Cu^2+^. Furthermore, good sensitivity for the detection of Cu^2+^ can be achieved by bare GQDs *via* EC and chemical reaction-driven FL quenching-recovery processes. Microplasma-generated crystalline GQDs and CA-derived GQDs (pyrolysis method) are also competitive probes for the FL detection of Cu^2+^, even at the visual level under UV light. Among the doped-GQDs, N-GQDs have shown potential to interact with Cu^2+^ and sense *via* the FL, COL or ECL method. Specifically, N-GQDs-deposited paper could selectively/sensitively probe Cu^2+^*via* the ECL strategy. The valorisation of waste into N-GQDs and the quantification of Cu^2+^ (under normal light) by simply analyzing smart-phone based images is another noticeable attempt. The advantage of GQDs/functionalized GQDs for the fabrication of composites/mixtures with other counterparts and their employment in the sensitive detection of Cu^2+^ cannot be underestimated. Specifically, the selective and low-quantity detection of Cu^2+^ with GQDs involved systems through the PEC sensing strategy and ratiometric ECL manner are noticeable.

### 6.5. Pb^2+^

The first demonstration of the turn-on-based FL detection of Pb^2+^ is related to a conjugated system containing 3,9-dithia-6-monoazaundecane (DMA)-functionalized GQDs (DMA–GQDs; sulfur-containing moiety) and tryptophan, which exhibited an LOD of 9 pM in the lower concentration region (Table 5). The Pb^2+^-induced formation of a rigid structure between the two conjugated components favoured energy transfer interaction for the enhancement of the fluorescence intensity. Moreover, the probe is applicable to quantify the Pb^2+^ concentration in rat brains.^263^ Subsequently, the ECL and EC detection of Pb^2+^ were demonstrated using GQDs-based systems, showing 10-fold lower LOD in EC detection compared to ECL technique (Table S4).

**Table 5:** GQDs, modified-GQDs, and GQDs involved with other counterparts for Pb^2+^ sensing application

GQDs-based sensor	Synthesis conditions	Size range/average size^a^ (nm)	QY (%)	Sensing process	LR (µM)	LOD (µM)	Ref.
DMA–GQDs/Tryptophan	HT (GO/DMA in water, 200 °C, 24 h); filtration	1–3/1.8	—	FL, turn-on	0.00001–0.001	9 × 10^−6^	263 d
GO/PDDA/G5/PDDA/GSH–GQDs@quartz SAMs	Pyrolysis (CA/GSH, 240 °C, 10 min); dissolved in water; chromatography; layer-by-layer deposition of GO, PDDA, G-rich DNA and GSH–GQDs on quartz substrate	6–10/—b	33.6^c^	FL, turn-off	0.0024–0.012	0.0022	264 d
AuCuNCs/N-GQDs@ GCE	HT (PANI/2 M NaOH in water, 220 °C, 12 h); centrifugation; used as reducing agent to synthesize CuNCs/N-GQDs; Galvanic exchange process to replace some surface CuNCs with Au using AuCl_3_, 65 °C, 4 h; drop-casted on GCE	3–5.5/—	—	EC, DPV	1 × 10^−6^–10, 20–1000	1 × 10^−6^	265 d
Hyb-BNQDs/N-GQDs@ GCE	HT (PANI/bulk boron nitride in water, two drops of 2 M NaOH, 220 °C, 24 h); filtration	5–9.9/—	—	EC, DPV	1 × 10^−6^–100	1 × 10^−6^	266 d
CuNCLs@N,S-GQDs@ GCE	HT (PANI in 0.05 M H_2_SO_4_ aqueous solution, 220 °C, 12 h); used during synthesis of CuNCLs from CuSO_4_/GSH; centrifugation; drop-casted on GCE	3–5.5/—	—	EC, DPV	1 × 10^−6^–50, 20–1000	1 × 10^−6^	267 d

a Measured from TEM.

b Size range/average size of GSH-GQDs used with other counterparts.

c QY of GSH–GQDs.

d Analytical ability in rat brain microdialysate/biological fluid/real water samples.

The employability of doped-GQDs, GQDs/functionalized GQDs along with Pb^2+^-specific DNA (aptamer-based sensor) and functionalized GQDs for the sensing of Pb^2+^ can be disclosed in Tables 5 and S4. Among the doped-GQDs, the fluorescence signal of S-GQDs was selectively quenched in the presence of Pb^2+^ and used for its quantification with satisfactory sensitivity (Table S4). A self-assembled multi-layer (SAM) device was constructed on a quartz substrate using GO/GSH–GQDs as an energy acceptor/energy donor and poly(diallydimethylammonium) chloride (PDDA)/G-rich ssDNA strand (G5) as a linker. The shortening of the distance between GO and GSH–GQDs due to the formation of a G-quadruplex (folded form of G5 DNA) in the presence of Pb^2+^ resulted in an enhancement in FRET between the energy acceptor and donor for the fluorescence quenching-based detection of Pb^2+^ with high sensitivity (Table 5) and applicability in real blood samples. However, the construction of this sensing platform required a complex procedure and expensive reagents, along with extra precaution during its sensing activity.^264^

To avoid the complicated/expensive fabrication of DNA-involved sensor systems, recently AuCuNCs/N-GQDs@GCE with temporal stability greater than one year was explored for the picomolar sensitive EC detection of Pb^2+^ (LOD: 1 pM), which explicitly surpassed the previously reported sensing performances (Tables 5 and S4). The significant current response with AuCuNCs/N-GQDs@GCE is attributed to the spontaneous reduction of Pb^2+^, which is facilitated by Au^+^ (basic electrolyte partially oxidizes Au metal to Au^+^) and electron rich N-GQDs. The effective Pb^2+^–Au^+^ interaction is responsible for the Pb^2+^ selectivity by AuCuNCs/N-GQDs. The low band gap (1.32 eV) and small charge transfer resistance (0.6 kΩ) of this electrode further support its appropriate electrocatalytic activity. Apart from its satisfactory reusability (97% retention of its current response after 50 washing cycles) and reproducibility (1.72% RSD for five different electrodes), the EC platform is also validated for the analysis of Pb^2+^-spiked environmental samples with recoveries/RSDs of 99–100.8%/<0.5%. Meanwhile, the consumption of expensive gold salt for the synthesis of the electrode materials and their degradation/oxidation during the sensing operation cannot be ignored.^265^

**Fig. 19:**
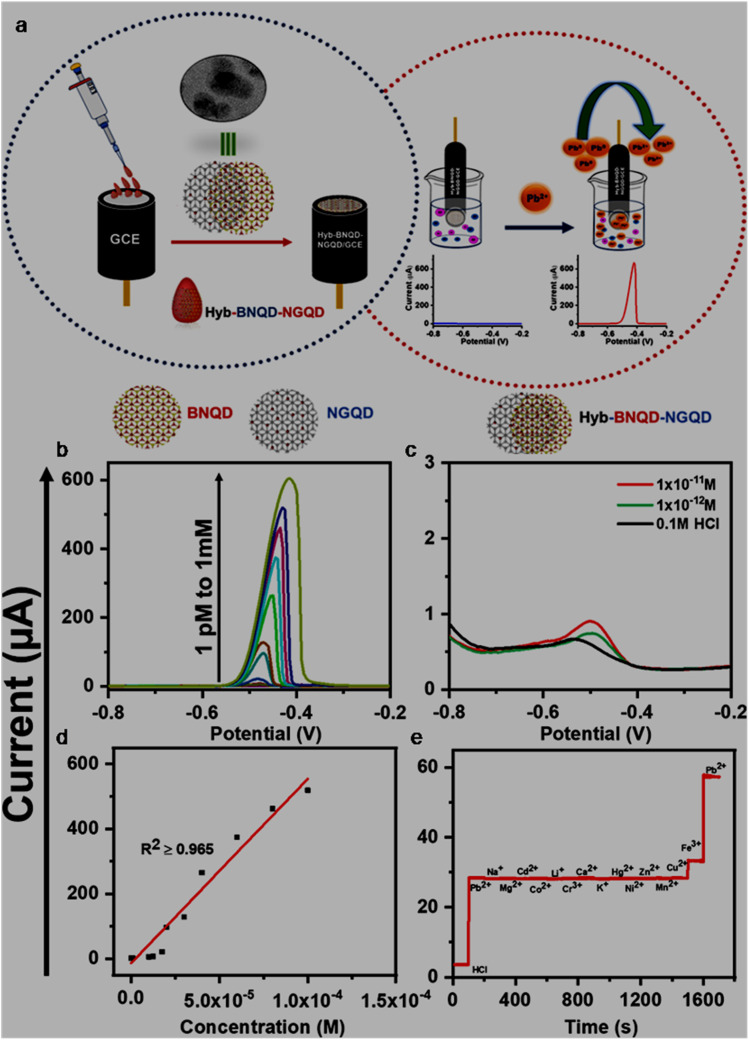
(a) Schematic showing the modification of GCE with Hyb-BNQDs/N-GQDs and its application for the DPV signal-based EC sensing of Pb^2+^. (b) DPV curve with increasing current response according to the Pb^2+^ concentration (1 pM–1 mM). (c) DPV current response with 10^−11^ and 10^−12^ M Pb^2+^, showing an LOD of 1 pM. (d) Plot of current *vs.* Pb^2+^ concentration (10^−12^ to 10^−4^ M). (e) Chronoamperometric profile showing negligible interference in the presence of other MIs. Reprinted (adapted) with permission from ref. 266, copyright 2024, the American Chemical Society.

In another recent report, a boron nitride (BN) QDs/N-GQDs hybrid system was used as an electrode modifier (Hyb-BNQDs/N-GQDs@GCE) and applied for the low-level detection of Pb^2+^. The N-GQDs in the hybrid configuration significantly improved the electrical conductivity, while BNQDs are advantageous for chemical inertness and overall stability.^266^ Unlike the previous EC electrode system, where the precious Au component of AuCuNCs facilitated the pre-reduction of Pb^2+^,^265^ here the electrode material functioned without the requirement of Au^+^-induced pre-reduction, and also excluded the use of costly chemicals in the synthesis process to achieve nearly equivalent sensitivity. Fig. 19a illustrates the fabrication of the electrode and its current response in the presence of Pb^2+^. The DPV responses of Hyb-BNQDs/N-GQDs@GCE according to the concentration of Pb^2+^ are shown in Fig. 19b, which exhibited an LOD of 1 pM (Fig. 19c) and linearly fitted in the dynamic concentration range of 1 × 10^−6^–100 µM (Fig. 19d). The chronoamperometric analyses (Fig. 19e) clearly depicted the selective response of the electrode material with Pb^2+^ and small non-interfering current from Fe^2+^. The sensing platform is well reusable (≥95% retention after 50 washing cycles), reproducible (≤4.8% RSD in the current response from five independent electrodes), and stable (∼97% retention in current after 180 days) for practical use. Moreover, this detection platform maintained its performance for the sensing of Pb^2+^ in wastewater samples (containing multiple interfering species) with good recoveries (>95%) and RSDs (≤5%).^266^ More recently, the same research group again reported the EC detection capability for Pb^2+^ using N,S-GQDs anchored with Cu nanoclusters (NCLs) (CuNCLs@N,S-GQDs) as an active electrode component. This EC probe not only featured picomolar-level sensitivity (Table 5) and good specificity (higher binding affinity for Pb^2+^ with thiol groups on the active material) but was also structurally stable, even after 365 days of storage (non-bonded and conjugated π electrons in the aromatic structure of N,S-GQDs stabilized CuNCLs). Its current response in the DPV curve did not require a pre-reduction step and Pb^2+^ was spontaneously/directly reduced to metallic Pb during the detection process due to the efficient electron-donating ability of N,S-GQDs. Additionally, this probe showed 98.67–99.80% recovery of Hg^2+^ in real water samples, appropriate reusability, reproducibility, and good temporal stability.^267^

*Summary*: The applicability of GQDs/modified-GQDs to quantify Pb^2+^*via* the FL, EC, and ECL methods is known. The presence of sulfur in GQDs can be favorable to specifically interact with Pb^2+^. Selectivity and satisfactory sensitivity in the FL detection of Pb^2+^ have been achieved by S-GQDs. GQDs involved hybrid/heterostructures are some of the appropriate choices to realize picomolar-level sensitivity and acceptable selectivity for the EC recognition of Pb^2+^. Specifically, hybrids of two QDs (Hyb-BNQDs/N-GQDs) and CuNCLs@N,S-GQDs heterostructures are suitable electrode modifiers for the EC detection of Pb^2+^ with perceptible performances.

### 6.6. Cr^6+^/Cr^3+^

N-GQDs were first employed for the label-free selective-sensitive detection of Cr^6+^ (Table 6) and simultaneous quantification of total Cr content (Cr^6+^ and Cr^3+^; Cr^3+^ is oxidized to Cr^6+^ by a chemical treatment protocol) in lake/river/domestic/industrial water samples. The functional groups in N-GQDs (nitrogen/oxygen-containing) showed stronger binding affinity and rapid chelating characteristic with Cr^6+^ in comparison to bare GQDs, indicating the suitability of the nitrogen element in the GQDs for Cr^6+^ sensing application.^268^ Subsequently, the ECL, FL, and EC detection of Cr^6+^/Cr^3+^ became possible using undoped/doped-GQDs, functionalized GQDs, and GQDs involved with other counterparts (Tables 6 and S5). For example, the IFE and SQE-based Cr^6+^ sensing performance of undoped GQDs *via* turn-off fashion with an LOD of 3.7 nM in a broad LR (0.05–500 µM) is noticeable. According to the FTIR analyses, the –OH, –COO^−^, and C–H groups present on the GQDs interact with Cr^6+^, resulting in fluorescence quenching; nevertheless, this report did not confirm the composition of GQDs using other reliable techniques such as XPS and TEM-based elemental analysis.^269^ The rapid sensing of Cr^6+^*via* the EC method (<1 min) using a GQDs/PANI composite-modified SPCE (GQDs/PANI@SPCE) exhibited satisfactory sensitivity (Table 6). This electrode was found to analyze more than 90 samples of Cr^6+^ per hour (each sample volume: 0.5 mL) and practical applicability in mineral water and deteriorated Cr-plating specimens. A report attempted to prepare the working electrode and Cr^6+^ sensing *via* automated mode, but the setup for online monitoring is very specific, not user friendly, and could not detect Cr^6+^ at the trace level. Moreover, Fe^3+^ is a potential interfering HMI (the reduction peak of Fe^3+^ effectively overlaps with Cr^6+^ even at the concentration ratio of 1 : 1) for the probe.^270^

**Table 6:** GQDs, modified-GQDs, and GQDs involved with other counterparts for Cr^6+^ sensing application^a^

GQDs-based sensor	Synthesis conditions	Size range/average size^b^ (nm)	QY (%)	Sensing process	LR (µM)	LOD (µM)	Ref.
N-GQDs	HT (CA/NH_3_, 200 °C, 10 h); diluted with water and excess NH_3_ removed by heating (100 °C, 1 h)	—/6.4	18.6	FL, turn-off	0–140	0.04	268 h
GQDs	GQDs purchased from XFNANO	0.5–2.5/1.2^c^	5	FL, turn-off	0.05–500	0.0037	269 h
GQDs/PANI@ SPCE	Pyrolysis (CA, 200 °C, 30 min); mixed in 10 mg per mL NaOH solution and pH adjusted to 4.0; diluted with water; loaded with aniline monomer onto SPCE; electro-polymerization	—	—	EC, SFA-LSV	0.1–10^d^	0.097^d^	270 h
sl-N-GQDs	HT (Xylan/urea/NaOH in water, 240 °C, 24 h); centrifugation; dialysis	—/3.2	23.8	FL, turn-off	3–150	0.43	121
sl-N-GQDs/PAAm hydrogel@MC	*In situ* immobilized in PAAm hydrogel and integrated with MC			"	3–75	0.1	121 h
N,S-GQDs	Pyrolysis (CA/TU, 180 °C, 30 min); dispersed in water; centrifugation; dialysis	1.6–5.7/2.8	22	FL, turn-off	1–100	0.01	120 h
					0.5–10^e^	0.4^e^	
CQDs@GQDs	CQDs derived from *Houttuynia cordata* extract *via* HT (180 °C, 4 h); pyrolysis (CA/CQDs, 220–240 °C, 5 min); mixed in 0.25 M NaOH solution	1–5/2.7	15	FL, turn-off	0.005–0.1^f^	0.01576^f^	271 h
				"	0.005–0.1^g^	0.00759^g^	
N-GQDs	HT (soluble starch/Arg in water, 190 °C, 4 h); centrifugation	1.4–3.4/2.4	10.9	FL, turn-off	0–50	0.8	272 h

a SFA-LSV: stopped-flow analysis with linear sweep voltammetry.

b Measured from TEM.

c Measured from AFM.

d LR/LOD in µg mL^−1^.

e LR/LOD of N,S-GQDs containing paper-based sensor.

f LR/LOD of Cr^6+^ during direct addition.

g LR/LOD of Cr^6+^ from the oxidation of Cr^3+^.

h Analytical ability in real water samples.

**Fig. 20:**
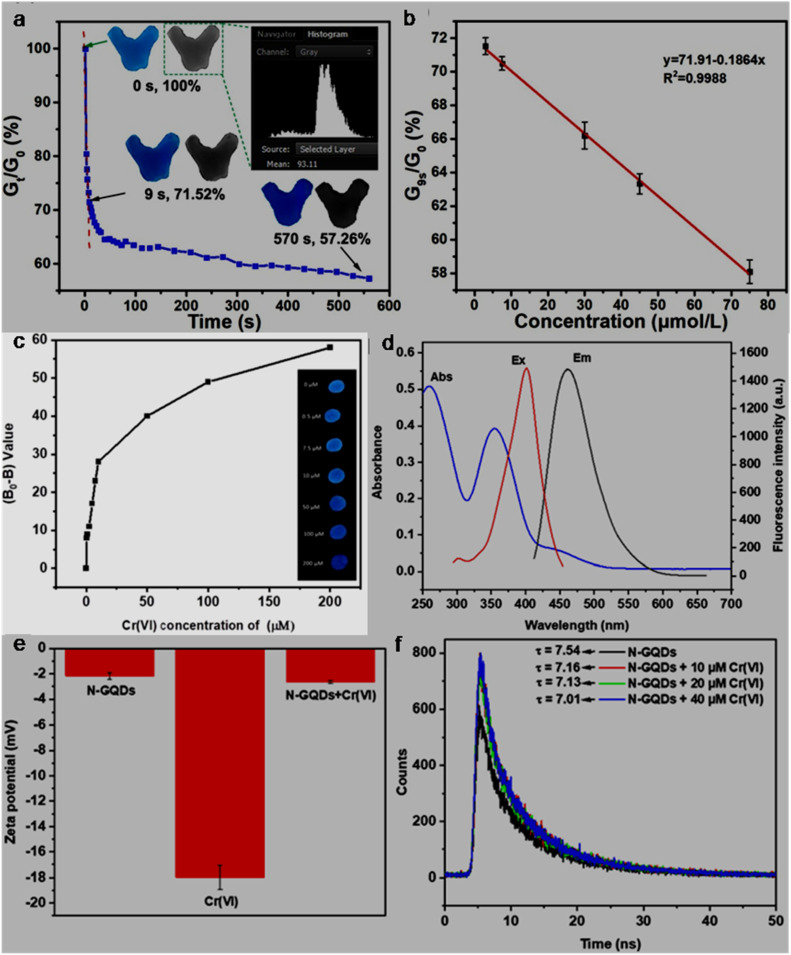
(a) Average gray-scale brightness profile after incorporating 3 µM Cr^6+^ solution into MC, showing a decreasing trend with time. (b) Decrease in gray-scale brightness (after 9 s) with different concentrations of Cr^6+^, showing linear fitting from 3 to 75 µM. Reprinted from ref. 121, copyright 2021, with permission from Elsevier. (c) Plot of the changes in blue value with respect to various concentrations of Cr^6+^ (0–200 µM), along with the corresponding fluorescence images under UV irradiation (inset). Reprinted (adapted) with permission from ref. 120, copyright 2023, the American Chemical Society. (d) Excitation-emission spectra of N-GQDs, showing overlap with the absorption spectrum of Cr^6+^. (e) Bar diagram of zeta potentials for N-GQDs, Cr^6+^, and N-GQDs in the presence of Cr^6+^. (f) PL decay curves showing *τ* value of N-GQDs without and with different concentrations of Cr^6+^. Reproduced/adapted from ref. 272 with permission from The Royal Society of Chemistry, 2024.

A self-passivated non-aromatic xylan layer on the surface of GQDs provided sl-N-GQDs (1.38% nitrogen) for application in the selective and sensitive sensing of Cr^6+^ (Table 6). The chelating effect between Cr^6+^ and nitrogen/oxygen functional groups of sl-N-GQDs destroyed the passivation boundary to cause fluorescence quenching, which was extended to a point-of-care device (portable microfluidic chip (MC)) for the smart-phone-based on-site/visual monitoring of Cr^6+^. An sl-N-GQDs-impregnated polyacrylamide (PAAm) hydrogel (*in situ* strengthened with cellulose nanofiber) was integrated with MC for the fabrication of a portable device and subsequent image-specific Cr^6+^ quantification. A significant decrease in the gray-scale brightness (100% to 71.52%) in 9 s by injecting 3 µM Cr^6+^ solution can be seen in Fig. 20a, which showed an acceptable LR/LOD of 3–75/0.1 µM (Fig. 20b) in the detection process. Additionally, the reliability of the portable platform was confirmed using real lake water-spiked samples, which showed satisfactory recoveries/RSDs (97–104/3.4–4.6%). However, due to its structural destruction during Cr^6+^ interaction, the probe lacks complete fluorescence recovery (even with excess AA, which reduces Cr^6+^ to Cr^3+^) and cannot be reused.^121^ Bezuneh *et al.*^120^ employed N,S-GQDs for the turn-off based FL sensing of Cr^6+^, which exhibited better sensitivity compared to N-GQDs (Table 6). The solution-phase sensing capability of N,S-GQDs (LOD: 10 nM) was extended to a paper-based device by evaluating smart-phone-captured changes in the blue values (*B*_0_–*B*) under UV light according to the Cr^6+^ concentration (0–200 µM, Fig. 20c), which is a simplified device attempt towards the on-site detection of Cr^6+^ in real tap/river water samples. Nevertheless, its detection sensitivity is inferior to previously reported MC-hydrogel devices (Table 6) and requires a longer incubation time (60 min) for acquiring a detectable blue value. In an observation, it was found that the LOD of Cr^6+^ (generated from the H_2_O_2_-induced oxidation of Cr^3+^) is lower (7.59 nM) than directly added Cr^6+^ (15.76 nM) in the low concentration domain when a CQDs@GQDs nanohybrid is used as a probe, showing the estimation capability of total Cr content in the water samples. This probe is applicable to achieving satisfactory Cr^6+^ recoveries in real water samples but the reason behind its improved sensitivity in the case of indirect Cr^6+^ detection is unclear.^271^

Recently, Ni *et al.*^272^ employed HT-synthesized N-GQDs (using bio-precursor: starch as a carbon source and Arg as a nitrogen source) with a high nitrogen content (11.98%) for the detection of Cr^6+^ but with an inferior performance compared to previously reported N-GQDs or N,S-GQDs (Tables 6 and S5). Based on UV-visible (overlap of Cr^6+^ absorbance with the excitation/emission spectra of N-GQDs, Fig. 20d), zeta potential (negative value similar to N-GQDs after the incorporation of Cr^6+^, Fig. 20e), and time-resolved spectroscopy (insignificant change in *τ* after Cr^6+^ addition, Fig. 20f) results, the quenching of fluorescence is attributed to the IFE and SQE mechanism. Moreover, the testing of Cr^6+^ in actual water (tap/bottled drinking/lake) samples showed satisfactory recoveries (92.6–103.3%) with RSDs less than 4.5%.

*Summary*: IFE-driven fluorescence quenching in the detection of Cr^6+^ (using GQDs-based systems) is the most common approach. The considerable selectivity and sensitivity achieved by N-GQDs/N,S-GQDs with Cr^6+^ HMI are some notable results. Furthermore, the extension of doped-GQDs to the fabrication of hydrogel-based MC devices and paper-based convenient devices is demonstrated, which served as sensing platforms (on-site level) for Cr^6+^ through smart-phone image analyses. The applicability of GQDs/PANI as electrode materials for the rapid and continuous (90 samples/h) detection of Cr^6+^ opens a new avenue to construct GQDs involved systems for the EC detection of Cr^6+^.

### 6.7. Cd^2+^

An earlier report showed ECL-based Cd^2+^ sensing using an N-GQDs/K_2_S_2_O_8_ co-reactant system with a good LOD (13 nM) in the lower concentration domain. The coordination of Cd^2+^ with the functional groups of N-GQDs caused ECL quenching, which was further recovered by the addition of EDTA.^195^ The subsequent development of GQDs-based systems and their performance in the detection of Cd^2+^ are compiled in Table 7.

**Table 7:** GQDs, modified-GQDs, and GQDs involved with other counterparts for Cd^2+^ sensing application

GQDs-based sensor	Synthesis conditions	Size range/average size (nm)^a^	QY (%)	Sensing process	LR (µM)	LOD (µM)	Ref.
N-GQDs	Acid oxidation of GO with HNO_3_:H_2_SO_4_ (4 : 1) under MW-reflux (240 W, 100 °C, 3 h); pH adjusted to 8.0; filtration; dialysis	2–7/4.5	11.7	ECL, turn-off	0.02–0.15	0.013	195
N-GQDs/TMPyP	HT (nitrogen-doped GO in water, pH adjusted to 8.0, 200 °C, 12 h); filtration	3–6.4/—	—	FL, turn-off	0.5–8	0.088	273 h
				COL	0.1–10	0.09	
N-GQDs@GCE	HT (PANI/2 M NaOH in water, 220 °C, 12 h); centrifugation; drop-casted on GCE	1.5–3.5/∼2.3	—	EC, DPV	1 × 10^−5^–100, 200–1000	1 × 10^−5^,^f^ 1 × 10^−7^^g^	197 h
N-GQDs	HT (CA/ethylenediamine in water, 180 °C, 4 h); centrifugation; dialysis	0.5–8/3.2	80^c^	FL, turn-on	1–25^d^	1.09^d^	274 h
				"	1–15^d^^,^^e^	0.59^d^^,^^e^	
GQDs/TPPS (1 : 6) @SPCE	MW (GA/triethylenetetramine in water, 300 W, 225 °C, 5 min); non-covalently modified with TPPS	0.5–6.5/—b	—	EC, SWV	0–8, 8–13	0.436	262 h
T–N-GQDs–CAA	HT (CA/urea in water, 160 °C, 4 h); dialysis; covalently modified with sodium alginate; added TMPyP and freeze dried	3.5–7.5/—b	—	COL, color change from red to green	10–2500^d^	5.10^d^	275 h

a Measured from TEM.

b Size range/average size of GQDs/N-GQDs used in the composite/aerogel.

c Absolute QY.

d LR/LOD in µg L^−1^.

e LR/LOD for N-GQDs containing paper-based sensor.

f LOD in ppb without pre-reduction.

g LOD in ppb with pre-reduction.

h Analytical ability in real water/herbal medicine samples.

**Fig. 21:**
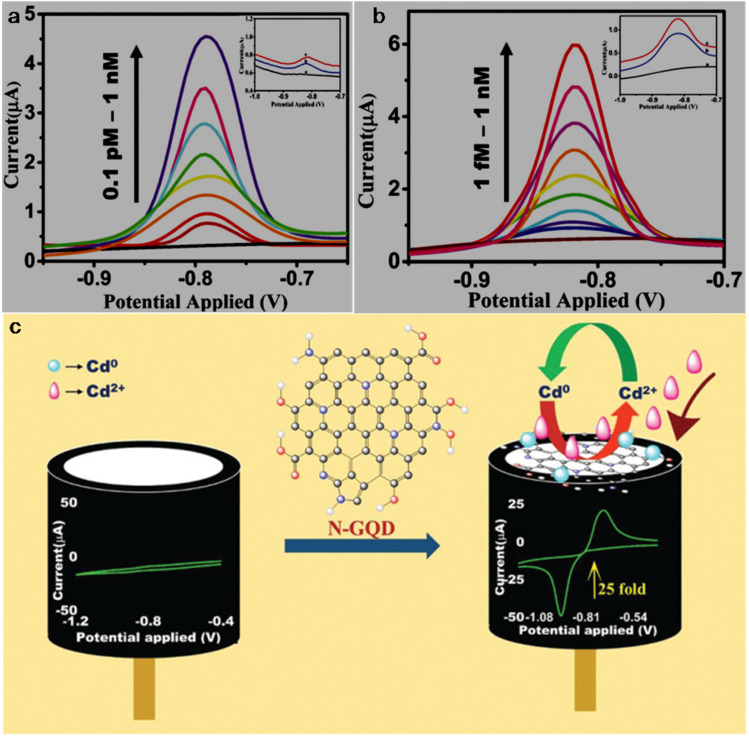
(a and b) DPV-based current response of N-GQDs@GCE in the presence of different concentrations of Cd^2+^ without and with the pre-reduction step, respectively. (c) Schematic of the deposition of N-GQDs on GCE, resulting in a 25-fold larger current response than bare GCE. Reproduced/adapted from ref. 197 with permission from The Royal Society of Chemistry, 2021.

For instance, owing to the enhanced conductivity and electrocatalytic activity of PANI-derived N-GQDs (∼10% nitrogen content, low bandgap), they showed high selectivity towards the EC detection of Cd^2+^ with a low LOD of 1 × 10^−5^ ppb (∼8.9 × 10^−5^ nM; without pre-reduction, Fig. 21a). The non-bonding electrons of the nitrogen atom and aromatic π moiety could spontaneously reduce Cd^2+^ into metallic Cd on the surface of N-GQDs for an EC response (Fig. 21c). Furthermore, by applying a pre-reduction step, N-GQDs@GCE exhibited an LOD as low as 1 × 10^−7^ ppb (∼8.9 × 10^−7^ nM; Fig. 21b), which is one of the best EC performances simply using N-GQDs without other components. The presence of nitrogen within the aromatic rings and as functional groups is crucial for the selective interaction of N-GQDs with Cd^2+^ rather than other HMIs. Moreover, the reusability/reproducibility/stability and applicability of the constructed sensor for Cd^2+^-spiked environmental samples (ground/sea/waste water) are quite satisfactory.^197^ Subsequently, the complexation of Cd^2+^ with the functional groups of N-GQDs (preferably –NH_2_) resulted in an unusual increment in fluorescence due to the inhibition of the PET process, and therefore followed the chelation-enhanced fluorescence (CHEF) mechanism. Consequently, the N-GQDs (absolute QY: 80%) showed considerable LODs of 1.09/0.59 µg l^−1^ in the solution-phase/paper-based detection process. Meanwhile, the solution/paper-based sensor systems also showed applicability in Cd^2+^-spiked real water and herbal medicine samples; however, the paper-based sensor required a large N-GQDs loading (3.4 mg mL^−1^) to achieve reasonable selectivity and the sensing platform was restricted to determining higher concentrations of Cd^2+^.^274^

**Fig. 22:**
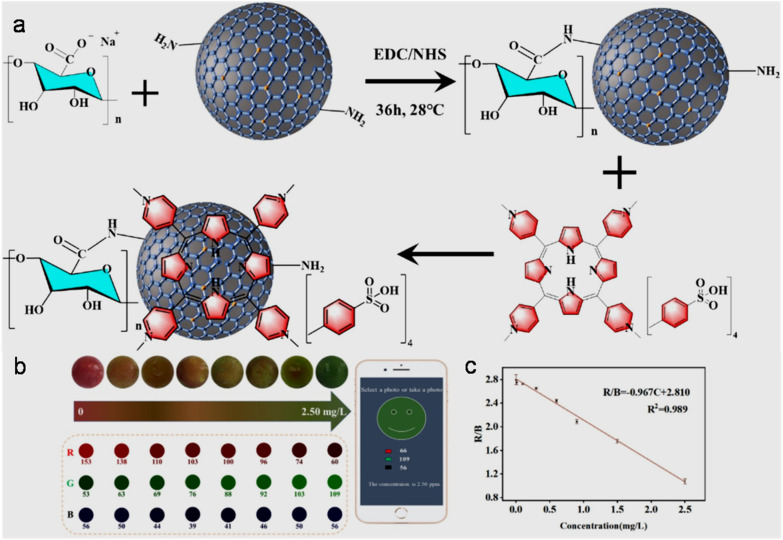
(a) Synthetic steps involved in the preparation of T–N-GQDs–CAA aerogel. (b) Changes in the colour of aerogel pellet with 0–2.5 mg per l Cd^2+^ and corresponding red (R)/green (G)/blue (B) values captured using a smart-phone. (c) Linear fitted plot between *R*/*B* ratio and concentration of Cd^2+^. Reprinted from ref. 275, copyright 2025, with permission from Elsevier.

Recently, the EC detection of Cd^2+^ using a GQDs/TPPS (1 : 6) (1 : 6 implies mass ratio of GQDs to TPPS) electrode material showed inferior sensitivity compared to previous EC results (Table 7). Additionally, the current response of Cd^2+^ with this electrode system was significantly reduced in the presence of Cu^2+^, showing a considerable interference issue.^262^ In another recent report, Tang *et al.*^275^ employed the EDC/NHS coupling reaction between N-GQDs and sodium alginate, followed by the addition of TMPyP and freeze drying to obtain a T–N-GQDs–CAA aerogel (Fig. 22a and Table 7) for the rapid (∼4 min) detection of Cd^2+^*via* the COL method. The electrostatic and π–π interactions originating from the N-GQDs facilitated the effective self-assembly of TMPyP in the aerogel and formation of ion channels for the favourable and rapid transportation of Cd^2+^ during the sensing operation. Due to the strong binding affinity of Cd^2+^ with TMPyP rather than the functional groups of N-GQDs, the aerogel pellet could trace an increasing concentration of Cd^2+^*via* the change in its colour from red to green (Fig. 22b) and showed a broad LR of 10–2500 µg l^−1^ (Fig. 22c), along with an acceptable LOD (5.10 µg l^−1^). The developed system was also validated for its practical utility to detect Cd^2+^ in tap (recoveries: 99.33–102%)/river (recoveries: 100–103%)/lake (recoveries: 102.4–104%) water with RSDs ranging from 1.08% to 4.32%.

*Summary*: According to the research developments in the GQDs-based detection of Cd^2+^, N-GQDs are the most suitable choice among doped-GQDs. The low-level detection of Cd^2+^ using the N-GQDs electrode material *via* the EC method and the applicability of N-GQDs/TMPyP-containing aerogel in the COL detection of Cd^2+^*via* smart-phone-based simple image analyses are notable results.

### 6.8. Co^2+^

The doped-GQDs encountered in the detection of Co^2+^ are summarized in Tables 8 and S6. For instance, Wang *et al.*^276^ demonstrated an AIE quenching (AIEQ) pathway in the selective and sensitive FL detection of Co^2+^ using N-GQDs with a low LOD (2 nM). The high affinity of Co^2+^ with amino functional groups present at the edges of N-GQDs induced their aggregation, and consequently fluorescence quenching. This nanoprobe was also extended to the intracellular monitoring and *in vitro* tracing of Co^2+^-induced cellular apoptosis in A549 living cells.

**Table 8:** GQDs, modified-GQDs, and GQDs involved with other counterparts for Co^2+^, Ni^2+^, Al^3+^, and As^3+^ sensing application

GQDs-based sensor	Synthesis conditions	Size range/average size^a^ (nm)	QY (%)	Sensing process	LR (µM)	LOD (µM)	Ref.
**Co** ^**2+**^							
N-GQDs	MW (PEI/l-lysine in water, 400 W, 120 °C, 5 min); dissolved in water and pH adjusted to 7.0; dialysis	4–6/5.2	—	FL, turn-off	0.01–5	0.002	276 d
N,S-GQDs	HT (CA/cysteamine·HCl in water, 160 °C, 4 h); centrifugation	1.1–5.4/3	—	FL, turn-off	0–40	1.25	277 e ^,^ f

**Ni** ^**2+**^							
EDA–GQDs	HT (GO/6 wt% H_2_O_2_/W_18_O_49_ nanowires in water, 200 °C, 96 h); covalently functionalized with EDA under HT (150 °C, 24 h)	2–6/4.2	83	FL, turn-off	0.1–50	0.03	278 d

**Al** ^**3+**^							
GQDs	MW (glucose/ethylene glycol in water, 800 W, 9 min); filtration; dialysis	1.5–7/∼3.3	2.5	FL, turn-on	0.4–500	0.0598	279 d

**As** ^**3+**^							
GQDs/GSH–rCQDs	GQDs purchased from Sigma-Aldrich; mixed with GSH–rCQDs	—	18.9^b^	FL, ratiometric	0.5–100^c^	0.5^c^	280 f

a Measured from TEM.

b QY of GQDs.

c LR/LOD in ppb.

d Analytical ability in living cells.

e Analytical ability in real water samples.

f Paper-based sensing capability.

**Fig. 23:**
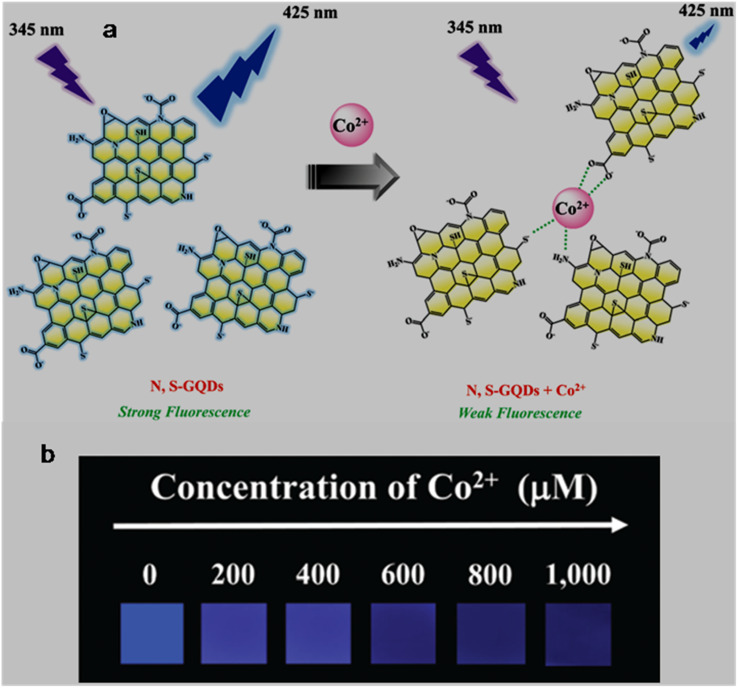
(a) Schematic showing the interaction of Co^2+^ with the functional groups of N,S-GQDs for the weakening of fluorescence. (b) N,S-GQDs-coated paper strips for the visual monitoring of Co^2+^ (0–1000 µM) under a 365 nm light exposure. Reproduced/adapted from ref. 277 with permission from The Royal Society of Chemistry, 2020.

Subsequently, the metal–ligand interaction between Co^2+^ and functional groups of N,S-GQDs (carboxyl, amino, and thiol) caused the aggregation of N,S-GQDs, and consequently the weakening of their fluorescence intensity (Fig. 23a). Moreover, N,S-GQDs showed potential to detect Co^2+^ in real water specimens (recoveries/RSDs: 91.2–108.2/0.1–7.3%) and construction of paper-based strip towards the visual monitoring of Co^2+^ (Fig. 23b). However, although this probe responded to Co^2+^ selectively in a broad LR (solution-phase), the calculated LOD (1.25 µM) is significantly higher than the normal level of Co^2+^ in human blood/urine (0.003/0.017 µM) and toxic level of 0.085 µM.^277^

*Summary*: Based on reports, the aggregation effect of N-GQDs or N,S-GQDs in the presence of Co^2+^ can result in fluorescence quenching, and furthermore quantify Co^2+^*via* the FL method. The presence of functional groups (nitrogen- and oxygen-containing) on the surface/edge of GQDs is essential for effective interactions/complexation with Co^2+^.

### 6.9. Ni^2+^

GQDs-based sensors have also been used in the detection of Ni^2+^ (Tables 8 and S6). For example, Xu *et al.*^278^ used bright yellow-fluorescent ethylenediamine (EDA)-functionalized GQDs (EDA–GQDs, QY: 83%) to sense Ni^2+^ with acceptable sensitivity (Table 8). Due to the strong coordination ability of EDA with Ni^2+^, the fluorescence signal of EDA–GQDs is quenched after the addition of Ni^2+^*via* the SQE mechanism (no change in the average lifetime (*τ*_av_) before and after the introduction of Ni^2+^). Moreover, the *in vitro* detection of Ni^2+^ in rADSC cells was confirmed by the suppressed fluorescence after internalizing biocompatible EDA–GQDs and subsequent incubation with 5 µM Ni^2+^.

*Summary*: There are few reports on GQDs-based sensors for Ni^2+^. The results inferred that Ni^2+^-specific functional group (such as EDA)-containing GQDs can be a suitable platform for the FL quenching-based detection of Ni^2+^.

### 6.10. Al^3+^

**Fig. 24:**
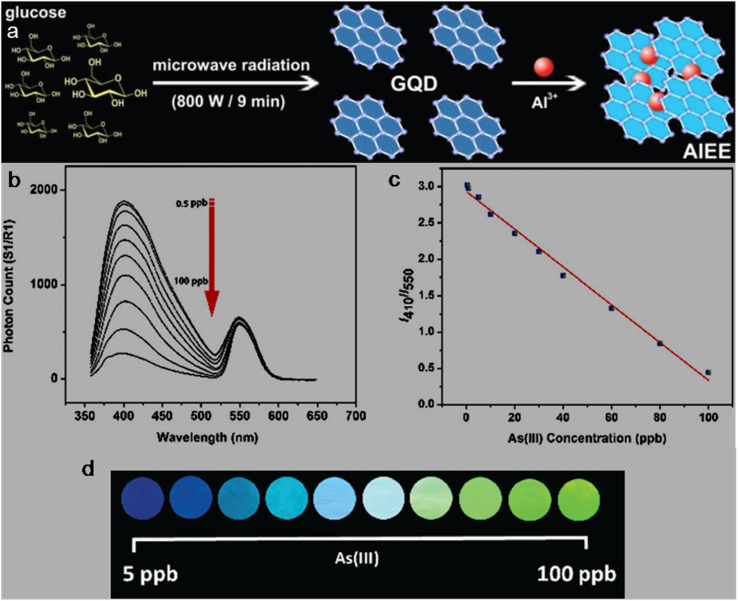
(a) Synthesis of GQDs *via* an MW-assisted method, and their analytical applicability for Al^3+^ detection *via* AIEE mechanism. Reproduced/adapted from ref. 279 with permission from The Royal Society of Chemistry, 2020. (b) Fluorescence spectra of GQDs/GSH–rCQDs with 0.5 to 100 ppb concentration of As^3+^. (c) Linear relationship between the I_410_/I_550_ intensity ratio and concentration of As^3+^. (d) Colour variations of GQDs/GSH–rCQDs-containing paper strips with As^3+^ (5 to 100 ppb) under UV light. Reproduced/adapted from ref. 280 with permission from The Royal Society of Chemistry, 2019.

The reasonable sensing performance of undoped GQDs in comparison to doped-GQDs in the fluorescence turn-on-based detection of Al^3+^ can be confirmed in Tables 8 and S6. For instance, Yao *et al.*^279^ employed a time-saving MW-assisted green protocol (800 W, 9 min) to synthesize GQDs (precursor: glucose) for the aggregation-induced enhanced emission (AIEE)-based detection of Al^3+^ (Fig. 24a). Although the QY of GQDs is quite low (2.5%), they could potentially detect Al^3+^ with a wide LR (Table 8) and LOD of 59.8 nM. Here, AIEE caused an increase in the fluorescence signal in the presence of Al^3+^ due to the restriction of GQDs to intra-molecularly rotate in the aggregated state (*via* coordination and electrostatic interaction), and therefore slowed down non-radiative decay. Moreover, satisfactory recoveries/RSDs of 96.8–109.7/4.72–8.87% in real complex samples (tab/drinking/pond/river water) validated the practical capability of the nanoprobe system.

*Summary*: The FL detection of Al^3+^ involved the fluorescence enhancement phenomenon, which is commonly caused by the creation of aggregated-state GQDs/doped-GQDs in the presence of Al^3+^. Undoped GQDs have shown high selectivity as well as sensitivity compared to doped-GQDs (B-GQDs and N-GQDs) in the turn-on type FL identification of Al^3+^.

### 6.11. As^3+^

The successful detection of toxic As^3+^ could also be possible with systems containing undoped GQDs (Tables 8 and S6). The AIEE effect in magnetic GQDs (Fe_3_O_4_NPs–GQDs; synergised by the presence of Fe_3_O_4_NPs and –OH groups) after the addition of As^3+^ was used to sense As^3+^ (satisfactory performance) in artificial and real water solutions *via* a turn-on manner (Table S6). Subsequently, a completely carbon-based ratiometric sensor was constructed (combining violet-emitting GSH-functionalized reduced CQDs (GSH–rCQDs) and green-emitting GQDs) for detecting As^3+^, which relied on the –SH group-mediated preferential interaction and subsequent quenching of violet-emission (410 nm) over green ones (550 nm) (Fig. 24b). Consequently, the dual-emissive nanohybrid probe enabled the dose-dependent sensitive quantification of As^3+^ in a wide LR of 0.5–100 ppb (Fig. 24c) and LOD of 0.5 ppb. Additionally, the GQDs/GSH–rCQDs-coated nitrocellulose test paper could visually detect As^3+^ by colour variations (blue to cyan to green, Fig. 24d) under UV illumination.^280^

*Summary*: The advantage of ratiometric probes (nanohybrids containing two carbon-based QDs) is obvious, which not only enabled the highly sensitive FL detection of As^3+^ but also a visual detection possibility by the variation in colour under UV light (semi-quantitative analysis).

### 6.12. Ag^+^

By taking the advantage of the formation of AgNPs (within 5 min) *via* the reduction of Ag^+^ on the surface of GQDs, the first FL detection of Ag^+^ using GQDs was realized in 2013.^281^ Subsequently, functionalized GQDs, doped-GQDs, bare GQDs, and systems containing doped-GQDs were successfully employed in the detection of Ag^+^ (Tables 9 and S7). For example, biomass (*Passiflora edulis sims*)-derived N-GQDs (4.6% nitrogen) showed the selective quenching of the fluorescence intensity in the presence of Ag^+^ with high sensitivity (Table 9). Based on the UV-visible (280 nm absorbance increased with the appearance of new/strong absorption at 220 nm after adding Ag^+^) and PL decay lifetime (almost unchanged *τ*_av_ before/after the addition of Ag^+^) analyses, it is proposed that the combined effect of SQE and electron transfer is accountable for the turn-off based detection process; however, deep insights into the selectivity and high sensitivity are lacking.^282^

**Table 9:** GQDs, modified-GQDs, and GQDs involved with other counterparts for Ag^+^ and Au^3+^ sensing application

GQDs-based sensor	Synthesis conditions	Size range/average size^a^ (nm)	QY (%)	Sensing process	LR (µM)	LOD (µM)	Ref.
**Ag** ^**+**^							
GQDs	Acid oxidation of GO with HNO_3_:H_2_SO_4_ (4 : 1) under MW-reflux (650 W, 100 °C, 8–10 h); pH adjusted to 8.0; filtration; reduced with NaBH_4_ (room temperature, 10 h); dialysis	5–6.2/5.5	—	FL, turn-off	0–0.1	0.0035	281
N-GQDs	HT (*Passiflora edulis sims* extract, 180 °C, 4 h); filtration	2–6/3.8	29	FL, turn-off	0.01–160	0.0012	282 d
N,S-GQDs@PtNCLs	HT (CA/TU in water, 160 °C, 4 h); dialysis; decorated with PtNCLs	18–28/21.53; 1.5–2.7/2.17^b^	—	COL, turn-off	0.0005–0.3	0.0002	283 d
N,S-GQDs/CdTeQDs	Pyrolysis (CA/GSH, 200 °C, 5 min); mixed in 0.3 M NaOH solution; drying; mixed with CdTeQDs	<5/—b	—	FL, ratiometric	0.00117–0.00588, 0.0472–0.118, 1.7–4.2	0.000226, 0.004679, 0.143	119 d ^,^ e

**Au** ^**3+**^							
N,S-GQDs	HT (CA/Cys in water, 200 °C, 8 h); centrifugation; dialysis	1–3.5/2.1	35.4	FL, turn-off	0.1–50	0.05	284 d
GQDs@CFP@PM	Pyrolysis (glucose, 200 °C, 20 min); dissolved in water; adsorbed on CFP; coated with PM solution	10–40/26.8^c^	—	COL, turn-off	200–1000	70	114 d ^,^ e

a Measured from TEM.

b Size range/average size of N,S-GQDs.

c Size range/average size of GQDs measured from dynamic light scattering.

d Analytical ability in real water/carbonated drink samples.

e Visual detection capability.

**Fig. 25:**
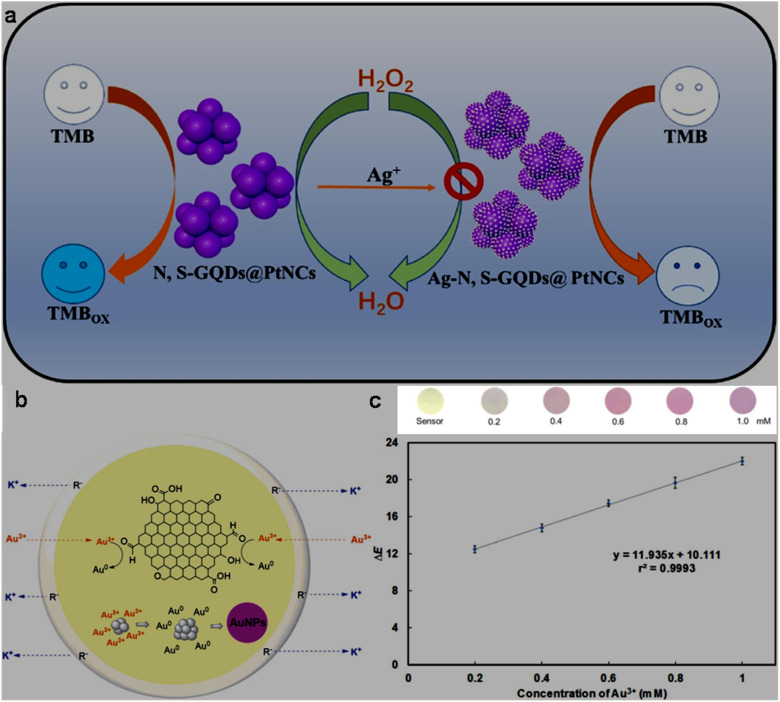
(a) Schematic of the COL sensing activity of N,S-GQDs@PtNCLs for Ag^+^ detection. Reprinted from ref. 283, copyright 2022, with permission from Elsevier. (b) GQDs-induced reduction of Au^3+^ to AuNPs. (c) Colour change in the assembled paper sensor with the addition of different concentrations of Au^3+^ and corresponding calibration plot. Reproduced/adapted from ref. 114 with permission from The Royal Society of Chemistry, 2021.

Xue *et al.*^283^ demonstrated the sensitive detection of Ag^+^*via* the COL method (LOD: 0.2 nM) using N,S-GQDs-decorated Pt NCLs (N,S-GQDs@PtNCLs). This probe catalyzed the oxidation of 3,3′,5,5′-tetramethylbenzidine (TMB) with H_2_O_2_ (peroxidase-activity, active species: ˙OH radical) to develop blue colour (652 nm absorption from oxidized TMB (TMB_ox_)), which was subsequently suppressed by the coverage of the probe surface with Ag metal (generated by the N,S-GQDs-mediated reduction of Ag^+^) (Fig. 25a). Meanwhile, the use of expensive Pt-salt for the generation of PtNCLs in the active probe cannot be neglected (N,S-GQDs did not show peroxidise-activity) and this probe is only applicable within the low concentrations of Ag^+^. Subsequently, a mixture of N,S-GQDs (nitrogen/sulfur content: 7.25/2.64%) and CdTeQDs was used to assemble a ratiometric platform (effective quenching of the 570 nm emission from CdTeQDs (93.31%) rather than the 424 nm emission from N,S-GQDs (10.76%)) for the FL as well as COL sensing of Ag^+^ with LOD up to 0.226 nM. When the concentration of Ag^+^ was low (below 0.14 µM), the quenching followed SQE due to the less possibility of collision, while both SQE and DQE were involved at higher concentrations of Ag^+^ (>0.14 µM). Moreover, the applicability of this nanoprobe in real water was justified by satisfactory recoveries/RSDs (97.85–100.6%/<3.5%). The low-level quantification and visual recognition (yellow-green to blue with increasing Ag^+^ concentration; under UV irradiation) of Ag^+^ with the ratiometric probe are notable, but the involvement of toxic semiconductor QDs and Fe^3+^/Fe^2+^ interference are some limitations.^119^

*Summary*: It can be inferred that N-GQDs are better FL probes for Ag^+^ compared to other single-heteroatom doped GQDs such as S-GQDs and undoped GQDs. The Ag^+^ detection capability of N,S-GQDs in heterostructured/mixed form with other counterparts such as PtNCLs and CdTeQDs through the COL and ratiometric manner is also considerable but with some limitations.

### 6.13. Au^3+^

The applicability of GQDs-based sensors for the recognition of Au^3+^ can be seen in Tables 9 and S7. For example, the blue-emission peak of N,S-GQDs (425 nm) is quenched in the presence of Au^3+^ due to the coverage of their surface with AuNPs, exhibiting a low LOD (50 nM) for Au^3+^. The formation of AuNPs is induced by the reactivity of Au^3+^ with the amine groups of N,S-GQDs and electron transfer from N,S-GQDs to Au^3+^. However, although this probe is very selective to Au^3+^, its optimum sensing activity occurs at elevated temperature (45 °C, 15 min incubation time) and the presence of Fe^3+^ potentially interferes with the quenching process (mercaptosuccinic acid is required to mask the interference from Fe^3+^). Moreover, this probe could successfully analyze Au^3+^ in spiked-real water samples and auranofin drug.^284^

Later, Thanomsak *et al.*^114^ fabricated a portable COL probe for the detection of Au^3+^ by adsorbing GQDs on cellulosic filter paper (CFP) and coating with a polymeric membrane (PM). The hydrophobicity created by the optimal PM coating on GQDs@CFP is important to avoid the leaching of GQDs from the paper sensor and effective ion-exchange in a short incubation time (10 min). The accumulation of Au^3+^ into the fabricated paper sensor *via* the cationic ion-exchange process and reduction to AuNPs through the electron-donating capability of GQDs (Fig. 25b) led to a visualize colour change (pale yellow to pink, without the assistance of UV light, Fig. 25c) in the LR of 200–1000 µM (Fig. 25c) with an LOD of 70 µM. Moreover, the paper-based sensor could be practically applied for the quantification of Au^3+^ in real water samples. However, the applicability of this paper sensor is limited to determining a high concentration of Au^3+^ and not suitable at trace level.

*Summary*: Dual-doped N,S-GQDs may be a good probe for the quenching-induced FL detection of Au^3+^. Although the sensitivity of bare GQDs-based paper sensors is inferior to the solution-phase FL detection results, the construction of a simple and low-cost platform for the rapid, real-time, and visual COL detection of Au^3+^ is notable.

### 6.14. Alkali/alkaline-earth MIs

Crown ether-like C-GQDs (edge possessing 78% oxygen atoms in the form of C–O–C) exhibited Ca^2+^ selectivity in the fluorescence quenching process (LOD: 2 pM) and *in vitro* detection proficiency of Ca^2+^ in hASCs (human adipose-derived stem cells). Besides, different sizes of the crown ether-like structures showed selectivity for Mg^2+^ (LOD: 20 pM), Sr^2+^ (LOD: 8 pM), and Ba^2+^ (LOD: 2 pM). Noticeably, the LODs for all the detected MIs are much lower than that of the previously reported PEG-modified N-GQDs (Table S8), which can be ascribed to the strong coordination ability of the crown ether-like structure with the respective MIs.^285^ Subsequently, GQDs were covalently modified with two crown ethers to create GQDs–15-crown-5 and GQDs–18-crown-6 composite systems for the sensing of both Na^+^ and K^+^*via* the EC method (Table 10). However, both systems suffered from selectivity issues unlike the previous system, where selectivity is achieved by confining PEG–GQDs in Na^+^/K^+^ specific ionophores (the synthesis of PEG–GQDs-confined ionophores followed a complex/expensive procedure) (Table S8). GQDs–18-crown-6 (*λ*_em_: 450/550 nm due to crown ether/GQDs components) was found to be selective up to a certain extent for K^+^ in a ratiometric manner (enhancement/suppression of 450/550 nm peaks with increasing concentration of K^+^), which is hypothesized to be due to the different interactions of K^+^ with the oxygen moieties of crown ether and GQDs. Meanwhile, detailed structural characterization of the synthesized probe, mechanism for its selectivity, interference study, and practical applicability are lacking in this report.^286^

**Table 10:** GQDs and modified-GQDs for alkali/alkaline-earth MI, rare-earth MI, and radioactive MI sensing application

GQDs-based sensor	Synthesis conditions	Size range/average size^a^ (nm)	QY (%)	Sensing process	LR (µM)	LOD (µM)	MIs	Ref.
**Alkali/alkaline-earth MIs**								
C-GQDs	HT (Alizarin in water, 150 °C, 24 h); dialysis	1–5.5/2.7	74	FL, turn-off	0–2 × 10^−4^	2 × 10^−6^	Ca^2+^	285 g
GQDs–15-crown-5@SPCE	Acid oxidation of MWCNTs with HNO_3_:H_2_SO_4_ (1 : 3) under ultrasonication (60 °C, 4 days); dilution and filtration; pH adjusted to 7.0; dialysis; covalently modified with 15-crown-5 or 18-crown-6	—/4.93^b^	—	EC, potentiometric	1–1 × 10^6^^e^	—	Na^+^	286
				"	"	"	K^+^	
GQDs–18-crown-6@SPCE		—/4.88^b^		"	"	"	Na^+^	
				"	"	"	K^+^	
GQDs–18-crown-6		—/4.88^b^		FL, ratiometric	"	"	K^+^	
DA–GQDs	Cutting of GO paste at 120 °C, 12 h; after dilution, pH adjusted to 3.0; filtration; centrifugation; dialysis; covalently modified with DA	2–5/—	—	FL, turn-on	4.93–10.61	0.05	Ca^2+^	131 g
Crown-GQDs–PEG_5_–Gd^3+^	ST (*o*-PDA/4-bromobenzo-18-crown 6 ether in ethanol, 180 °C, 50 h); ethanol replaced with water; filtration; dialysis; modified with PEG_5_; loaded with Gd^3+^; dialysis	1.84–10.12/3.73^c^		FL, turn-off	2500–25 000	3800	K^+^	287 g
		1–11/4.82^d^		Relaxometry, turn-off	5000–150 000	14 120	K^+^	

**Rare-earth MIs**								
N-GQDs	Chemical oxidation of GO/lysine with 30% H_2_O_2_ under reflux (130 °C, 4 h); removal of excess H_2_O_2_; filtration; dialysis	1.5–3.5/—	13.2	FL, turn-off	0.3–15	0.11	Eu^3+^	288
GQDs	Acid oxidation of GSs with HNO_3_:H_2_SO_4_ (3 : 1) under ultrasonication (18 h); diluted with water, Filtered & pH adjusted to 8.0; HT (200 °C, 10 h); filtration; dialysis	15–20/—	7.2	FL, turn-off	50–230	0.38	Ce^3+^	289
GQDs/*o*-PDA	GQDs purchased from XFNANO	—/4.2	—	FL, ratiometric	5–100	1.0	Ce^4+^	290 h
GQDs	Acid oxidation of GO with HNO_3_:H_2_SO_4_ (1 : 3) under reflux (110 °C, 24 h); diluted with water & pH adjusted to 8.0; filtration; dialysis	1.5–3.8/2.5	—	FL, turn-on	0–30	0.3	Tb^3+^	291

**Radioactive MIs**								
GQDs	Gamma radiolysis of GO/25% H_2_O_2_ in water (270 kGy, 11.75 kGy h^−1^); drying & dispersion in water	2.3–8.8/4.6	10.2	FL, turn-off	2.24–21.43^f^	0.56^f^	U^6+^	292
ER-GQDs	Drop-casted GQDs on Au or GCE and electrochemically reduced	—		EC, SWV	23.4–345.8^f^	2^f^	"	292 h
PA@N-GQDs	HT (CA/urea in water, 160 °C, 4 h); filtration; covalently modified with PA	4.2–8.7/6.5	—	FL, turn-off	10–80	2.01 × 10^−3^	U^6+^	132
				"	10–60	1.35 × 10^−3^	Th^4+^	

a Measured from TEM.

b Size range/average size of GQDs measured from dynamic light scattering.

c Size range/average size of crown-GQDs.

d Size range/average size of crown-GQDs–PEG_5_.

e Dynamic concentration range.

f LR/LOD in µg L^−1^ or ppb.

g Analytical ability in living cells.

h Analytical ability in real water samples.

**Fig. 26:**
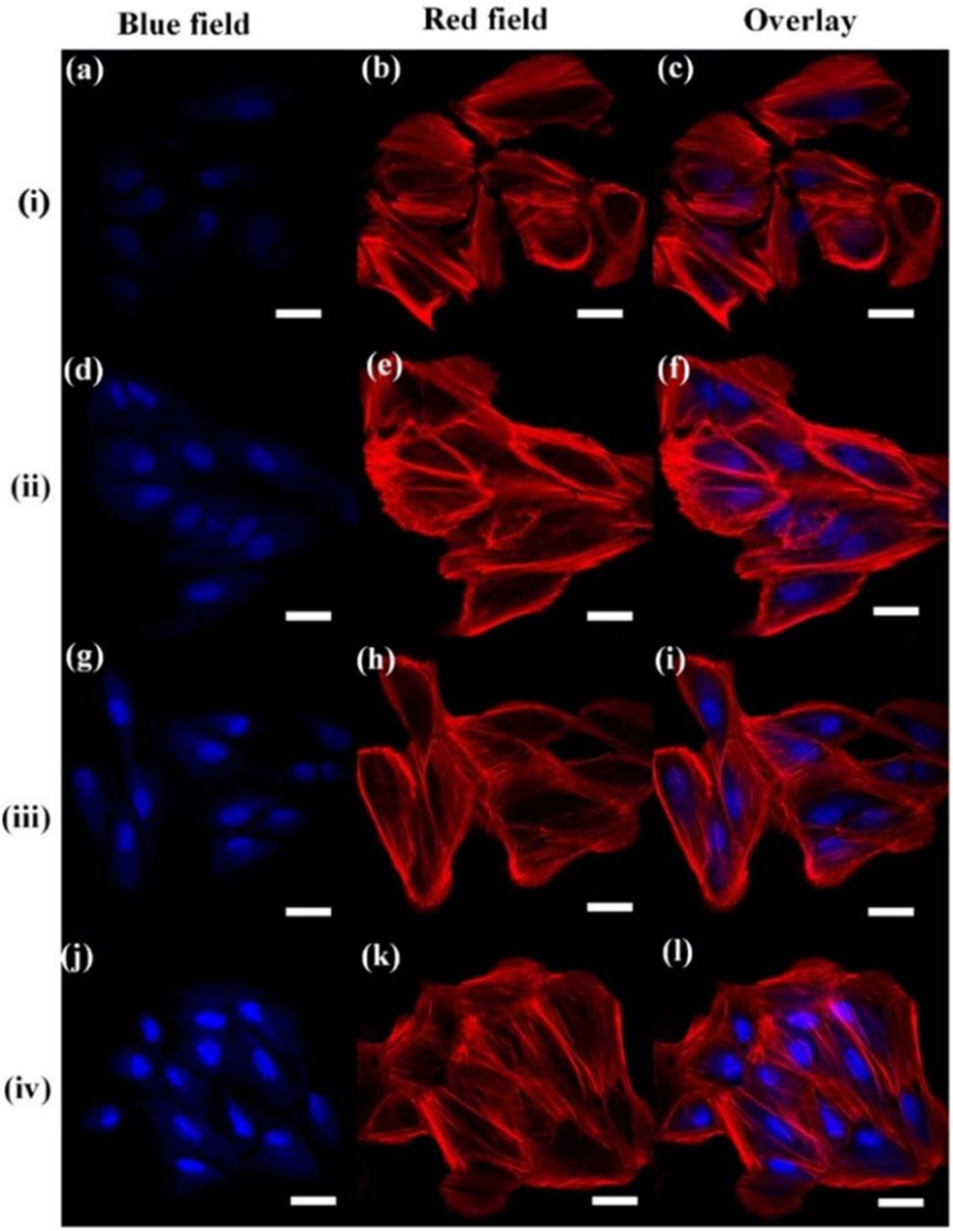
Confocal images of living cells (ARPE-19) after incubation with DA–GQDs (i) and further treatment with 2.5 µM (ii), 5.0 µM (iii), and 7.5 µM (iv) Ca^2+^ under blue field (first column; a, d, g, j), red field (middle column; b, e, h, k), and overlay (right column; c, f, i, l). Reprinted from ref. 131, copyright 2024, with permission from Elsevier.

A recent report demonstrated the detection of Ca^2+^ and its intracellular tracking using DA–GQDs through an atypical FL turn-on fashion. It was observed that Ca^2+^ is prone to coordinate with the oxygen groups of DA–GQDs to improve the selectivity and sensitivity. The blocking of PET and strengthening of internal charge transfer after the coordination of Ca^2+^ with DA–GQDs caused a fluorescence enhancement in the detection process. Although the LOD of Ca^2+^ with DA–GQDs (50 nM) is much higher in comparison to previous results (Tables 10 and S8), their wide LR (4.93–10.61 µM), selectivity from specific functional group, and turn-on type FL detection are advantageous for the analysis of a broad range of concentrations with high accuracy. This probe is also applicable to quantify Ca^2+^ in an organic compound (calcium gluconate) with slightly lower sensitivity (LR/LOD: 14.56–45.45 µM/100 nM). Moreover, the progressive intensification of the blue fluorescence with an increase in amount of Ca^2+^ (0, 2.5, 5.0, and 7.5 µM) in ARPE-19 (human retinal pigment epithelium cells) living cells (Fig. 26) confirmed the Ca^2+^ monitoring capability of the biocompatible probe (>70% cell viability at 150 µg mL^−1^ concentration, incubation time: 72 h) in biological matrices.^131^

**Fig. 27:**
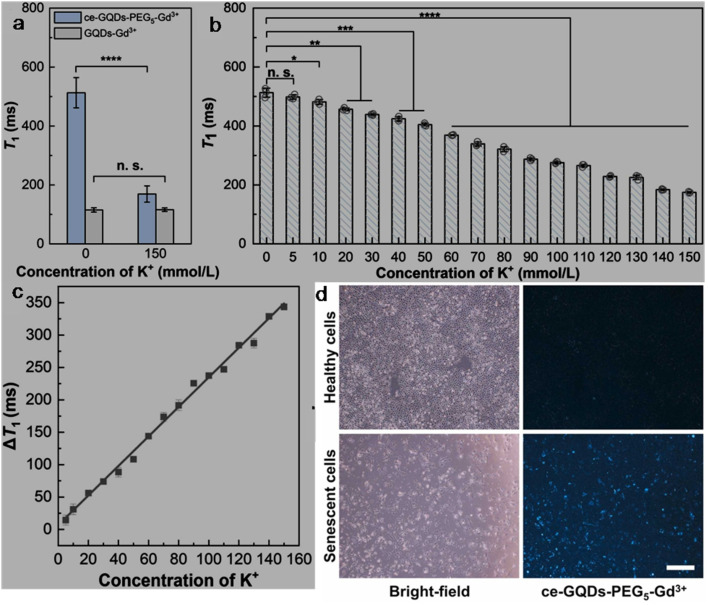
(a) Changes in T_1_ for crown-GQDs–PEG_5_–Gd^3+^ and crown-GQDs–Gd^3+^ probes after adding 150 mM K^+^. Decreasing trend in T_1_ with an increase in the concentration of K^+^ (b) and linear fitting plot between Δ*T* and concentration of K^+^ (c) for crown-GQDs–PEG_5_–Gd^3+^. (d) Healthy and senescent cells incubated with crown-GQDs–PEG_5_–Gd^3+^ and imaged under bright-field (left) and fluorescence mode (right). Reprinted from ref. 287, copyright 2025, with permission from Elsevier.

Recently, Chen *et al.*^287^ linked Gd^3+^ (magnetic site) and crown ether-possessing GQDs *via* bridging with PEG_5_ molecules to construct a crown-GQDs–PEG_5_–Gd^3+^ probe for the dual-mode (FL and nuclear magnetic resonance (NMR)-based relaxometry) identification of K^+^. Due to the binding selectivity of crown ether with K^+^, the fluorescence intensity gradually decreased in the FL sensing process. Additionally, the magnetic probe showed a significant change in the relaxometry response (T_1_; measured from NMR analyses) rather than the probe where Gd^3+^ is directly attached to crown-GQDs (crown-GQDs–Gd^3+^) (Fig. 27a). The gradual decrease in T_1_ with an increase in the concentration of K^+^ (5–150 mM, Fig. 27b) and linearly fitted curve (Fig. 27c) by the probe showed the relaxometry-based successful detection of K^+^. The changes in T_1_ with K^+^ are ascribed to the variations in proton concentration at the paramagnetic centre (Gd^3+^), which is facilitated by the PEG_5_ chains (proton transporter with low energy barrier). Although the sensitivity of K^+^ with ^1^H NMR-based detection is lower than the FL method (Table 10), it provides a new sensing opportunity to detect alkali MIs and other MIs in the future. After verifying the insignificant toxic effect, the probe effectively differentiated senescent cells (related to K^+^ concentration, high fluorescence and different morphology) from healthy ones (amount of probe: 200 µg mL^−1^, incubation time: 24 h; Fig. 27d) and exhibited easy penetration capability within the blood–brain barrier, which is a crucial achievement to monitor K^+^-induced aging effects and other related diseases.

*Summary*: The crown ether/crown ether-like structure in GQDs can significantly improve their coordination ability with alkali/alkaline-earth MIs to achieve promising selectivity as well as sensitivity in the FL detection process. Specifically, the presence of DA functionality in GQDs is advantageous for the selective and turn-on-type sensitive detection of Ca^2+^ even in biological media. Additionally, the selective/sensitive detection of K^+^ using the crown-GQDs–PEG_5_–Gd^3+^ probe *via* dual sensing techniques (FL and NMR) and identifying K^+^-induced senescence in healthy cells are considerable achievements.

### 6.15. Rare-earth MIs

Various rare-earth MIs such as Eu^3+^, Ce^3+^, Ce^4+^, and Tb^3+^ could be recognized by GQDs/doped-GQDs (Tables 10 and S8). For example, Ce^4+^ catalyzed the oxidation of *o*-PDA (oxidase-like activity), resulting in a strong emission from the oxidized *o*-PDA (*o*-PDA_ox_, 562 nm) and quenching of the fluorescence of GQDs (444 nm) through IFE. As a result, the ratiometric detection of Ce^4+^ by analyzing the *I*_562_/*I*_444_ intensity ratio showed a satisfactory performance (Table 10) and practical ability in lake water samples. However, the Ce^4+^-induced oxidase-like reaction required a long incubation time (1 h) under dark conditions, and therefore the whole sensing process is time consuming. The sensing activity also exhibited significant interference in the presence of Cu^2+^ and Ag^+^, which required EDTA and I^−^ to inhibit their interference, respectively.^290^

**Fig. 28:**
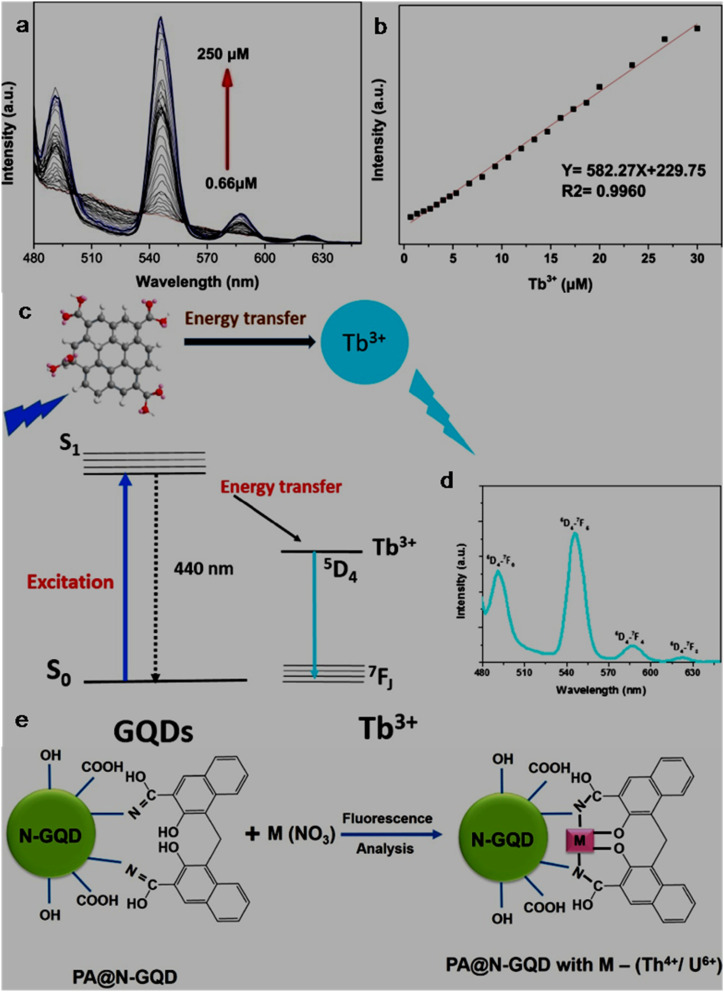
Fluorescence spectra of GQDs in the presence of different amounts of Tb^3+^ (0 to 250 µM) (a) and linear plot corresponding to 546 nm peak intensity *vs.* Tb^3+^ concentration (b). Schematic of the energy transfer steps involved in the sensing operation (c) and corresponding fluorescence spectrum (d). Reprinted from ref. 291, copyright 2019, with permission from Elsevier. (e) Schematic of the Th^4+^/U^6+^ interaction with PA@N-GQDs during the sensing process. Reprinted from ref. 132, copyright 2024, with permission from Elsevier.

Wang *et al.*^291^ demonstrated the selective and sensitive FL sensing of Tb^3+^ using GQDs, which involved a significant antenna effect to increase all four fluorescence peak intensities (490 (^5^D_4_ → ^7^F_6_)/546 (^5^D_4_ → ^7^F_5_)/585 (^5^D_4_ → ^7^F_4_)/620 (^5^D_4_ → ^7^F_3_) nm, originating from Tb^3+^–GQDs) with Tb^3+^ concentration (Fig. 28a) and LR of 0–30 µM (Fig. 28b). Fig. 28c and d depict the energy transfer operation (from GQDs to Tb^3+^) and resulting fluorescence in the Tb-GQDs *via* the antenna effect. The excitation of an electron from the ground state (S_0_) to the excited state (S_1_) of GQDs by the absorption of light, followed by the transfer of its energy to Tb^3+^ further excites and emits long-living fluorescence *via* a line-type f–f transition. Although the low fluorescence from Tb^3+^ in aqueous medium is reasonably enhanced by GQDs, the effective emission characteristic using a high energy *λ*_ex_ (230 nm) and requirement of long sensing time (30 min for reaction between GQDs and Tb^3+^) are limiting factors.

*Summary*: Both undoped and doped-GQDs (N-GQDs) are applied for the FL detection of rate-earth MIs. Good sensitivity in the FL detection of Ce^4+^ is achieved with bare GQDs through their oxidase-like activity and ratiometric manner rather than N-GQDs *via* a turn-off manner. Moreover, the specific antenna effect between GQDs and Tb^3+^ qualified Tb^3+^ according to the FL turn-on principle with a satisfactory performance.

### 6.16. Radioactive MIs

The applicability of GQDs-based platforms for the sensing of radioactive MIs is presented in Tables 10 and S8. For instance, GQDs synthesized from gamma radiolysis were used for the low-level detection of U^6+^ (LOD: 0.56 ppb) through electrostatic interaction and a FRET-based fluorescence quenching process. However, the FL detection strongly suffered from interference from Fe^3+^ (98% quenching). Furthermore, the electrochemically reduced GQDs (ER-GQDs) were utilized for the EC detection of U^6+^ (*via* cathodic SWV response, which is superior to the cathodic DPV signal). The estimated LOD (2 ppb) was found to be higher than FL method but EC detection is advantageous for higher concentration measurement (Table 10), elimination of Fe^3+^ interference (Fe^3+^ is precipitated to red-coloured Fe(OH)_3_ in saturated Na_2_CO_3_ electrolytic solution), and sensing of U^6+^ in ground water.^292^ The extraction of large-sized GQDs clusters (∼290 nm) from a supramolecular hydrogel and their utilization for the wide LR detection of U^6+^ can be revealed in Table S8. Subsequently, PA-functionalized N-GQDs (PA@N-GQDs, 7.4 at% nitrogen) were applied for the turn-off based FL detection of U^6+^ as well as Th^4+^ due to the strong binding interactions between them (Fig. 28e). Based on the *K*_SV_, binding constants, and association/dissociation constants analyses, it is deduced that Th^4+^ possesses higher binding affinity with PA@N-GQDs in comparison to U^6+^, resulting in 99/60% instant fluorescence quenching (within one minute) with the addition of Th^4+^/U^6+^. As a result, the probe could detect Th^4+^ in a more sensitive manner than U^6+^ (Table 10). It is also noticeable that the achieved LOD for U^6+^ is much lower than that in previous reports (Tables 10 and S8). However, the reversibility of the probe with EDTA is not satisfactory and cannot be used respectively.^132^

*Summary*: Nitrogen-doping and the presence of additional functional groups (*e.g.*, PA) in GQDs are advantageous for their selective interaction with radioactive cations (U^6+^ and Th^4+^), and consequently the attainment of high sensitivity in FL detection. Moreover, the U^6+^ detectability of ER-GQDs *via* the EC method opens the possibility for the development of GQDs-based EC platforms to quantify hazardous radioactive MIs.

### 6.17. Multiple HMIs

#### 6.17.1. EC detection

The GQDs-based electrode materials involved in the EC detection of multiple HMIs are consolidated in Tables 11 and S9. For instance, the highly selective and sensitive EC detection of Hg^2+^/Cu^2+^/Cd^2+^ (separately, LODs of Hg^2+^/Cu^2+^/Cd^2+^: 9.8/8.3/4300 pM) using vertically aligned mesoporous silica-nanochannel film (VMSF)-confined OH-GQDs and NH_2_-GQDs through the reduction of HMIs, followed by DPV-based anodic stripping at specific potentials is inspiring (Table 11); however, the complex/precise experimental conditions in the fabrication of modified-electrodes and the potential-specific electrodeposition of different HMIs before reduction/stripping operations cannot be avoided. Functional group-containing GQDs significantly amplified the signal response after their selective interaction with the analytes in the nanoconfined region *via* effective charge transfer, while the VMSF layer on the ITO electrode served as an anti-fouling and interference inhibitor. Besides Cd^2+^ recognition in soil-leached solution with NH_2_-GQDs@VMSF/ITO, OH-GQDs@VMSF/ITO was used for the quantification of Hg^2+^ in the presence of Cu^2+^ and Hg^2+^/Cu^2+^ sensing in seafood/human serum, dictating the practical approach of the fabricated probes.^293^

**Table 11:** GQDs, modified-GQDs, and GQDs involved with other counterparts for multiple HMI sensing application

GQDs-based sensor	Synthesis conditions	Size range/average size^a^ (nm)	QY (%)	Sensing process	LR (µM)	LOD (µM)	HMIs	Ref.
**EC sensor**								
OH–GQDs@ VMSF/ITO	HT (TNP in 0.125 M NaOH aqueous solution, 200 °C, 2 h); dialysis; filtration; electrophoresis confinement in VMSF/ITO electrode	0.9–2.9/1.83^b^	21^c^	EC, DPV	1 ×10^−5^–0.001, 0.001–0.5	9.8 × 10^−6^	Hg^2+^	293 f ^,^ g
				"	1 × 10^−5^–0.001, 0.001–1.5	8.3 × 10^−6^	Cu^2+^	293 f
NH_2_–GQDs@ VMSF/ITO	HT (TNP in 0.4 M NH_3_/1.5 M hydrazine hydrate aqueous solution, 200 °C, 2 h); dialysis; filtration; electrophoresis confinement in VMSF/ITO electrode	1.3–2.9/1.9^b^	29.8^c^	"	0.02–1, 1–20	0.0043	Cd^2+^	293 f
N,S-GQDs@ GCE	HT (PANI in 0.05 M H_2_SO_4_ aqueous solution, 220 °C, 12 h); drop-casted on GCE	3–5/5.4	—	EC, DPV	0.0001–100	1 × 10^−6^	Cd^2+^	198 f ^,^ g
				"	0.0001–100	1 × 10^−5^	Pb^2+^	
				"	0.0001–100	1 × 10^−6^	Hg^2+^	

**FL sensor**								
**Undoped/doped-GQDs**								
N,S-GQDs	HT (TNP/TU in 10 mM NaOH aqueous solution/10% DMF, 200 °C, 10 h); dialysis	1.6–2.8/2.1	23.2^c^	FL, turn-off	0.01–25	0.008	Fe^3+^	295 f ^,^ g
				"	0.4–180	0.25	Cu^2+^	
				"	0.1–140	0.05	Ag^+^	
N-GQDs	Plasma-contacting liquid synthesis (glucosamine in water, plasma irradiation, atmospheric pressure, below 80 °C, 10 min); filtration; dialysis	2–8/4.8	—	FL, turn-off	0–95	—	Fe^3+^	296
				"	"	"	Pd^2+^	
				"	"	"	Hg^2+^	
				"	"	"	Cu^2+^	
				"	"	"	Pb^2+^	
				"	"	"	Co^2+^	
N-GQDs	HT (Bean dregs power in water, 180 °C, 12 h); filtration; dialysis	0.38–3.74/1.63	21.3	FL, turn-off	0–2000	2.5	Ce^4+^	297
				"	0–1600	1.9	Fe^3+^	
N-GQDs	HT (GO obtained from spent graphite/NH_3_·H_2_O in water, 200 °C, 1.5 h); filtration; dialysis	0.5–4.5/2.44	11.04	FL, turn-off	60–200	0.23	Fe^3+^	298 f
				FL, turn-on	20–200	1.101	Al^3+^	

**Functionalized GQDs**								
Am–GQDs	Electrolysis of graphite rod in NaOH/ethanol, 24 h; dialysis; pH adjusted to 7.0 and re-dispersed in water; gamma irradiation (25 kGy, Ar) with 4 vol% ethylenediamine/3 vol% isopropyl alcohol; dialysis	—/16	5.82	FL, turn-off	0–7.5	1.79	Co^2+^	300 f
				"	0–4.0	0.657	Pd^2+^	
				"	0–45	2.55	Fe^3+^	
PEG–Pb-GQDs	HT (Cane molasses/lead acetate in water, 190 °C, 24 h); filtration; mixed with PEG-200	1–1.8/1.4	30.31	FL, turn-off	28–44	0.29	Fe^3+^	301 f ^,^ g
				"	20–140	1.08	Cu^2+^	301 g
				"	20–160	3.24	Ag^+^	301 g
NN–GQDs	Electrolysis of graphene foam in 0.1 M NaOH/urea ethanolic solution, 30 V; centrifugation; filtration; covalent modification with NN	2–7/∼3^b^	—	FL, turn-off	—	1^e^	Hg^2+^	302 f ^,^ g
				"	—	3^e^	Fe^3+^	

**GQDs involved with other counterparts**								
DPA–GQDs/Amino acid	HT (CA/DPA in water, 200 °C, 2.5 h); diluted with water; combined with different amino acids	—/0.8^b^	—	FL/COL, turn-off	0.01–1^d^	0.1^d^	Cu^2+^	303 f ^,^ h
				"	"	"	Hg^2+^	
				"	"	"	Fe^3+^	
N-GQDs/GSH–AuNCLs	HT (GO/NH_3_·H_2_O in water, 170 °C, 6 h); filtration; mixed with GSH–AuNCLs aqueous solution	1.3–3.32/2.26^b^	24.42	FL, ratiometric	0.08–6	0.00412	Cu^2+^	304 f ^,^ h
				"	1–40	0.943	Cd^2+^	

a Measured from TEM.

b Size range/average size of GQDs in confined system/before functionalization/involved with other counterparts.

c Absolute QY.

d LR/LOD in ppm.

e LOD in µg L^−1^ predicted from machine learning-based algorithm.

f Analytical ability in real water/other real samples.

g Simultaneous detection capability of multiple HMIs.

h Paper-based sensing capability.

**Fig. 29:**
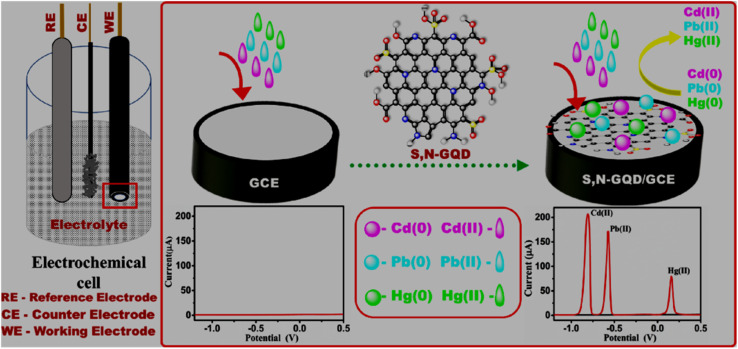
Schematic showing the deposition of N,S-GQDs on GCE surface to fabricate a working electrode for the EC cell, and its DPV response in the simultaneous presence of Cd^2+^, Pb^2+^, and Hg^2+^, resulting in peaks at different stripping voltages. Reprinted (adapted) with permission from ref. 198, copyright 2023, the American Chemical Society.

**Fig. 30:**
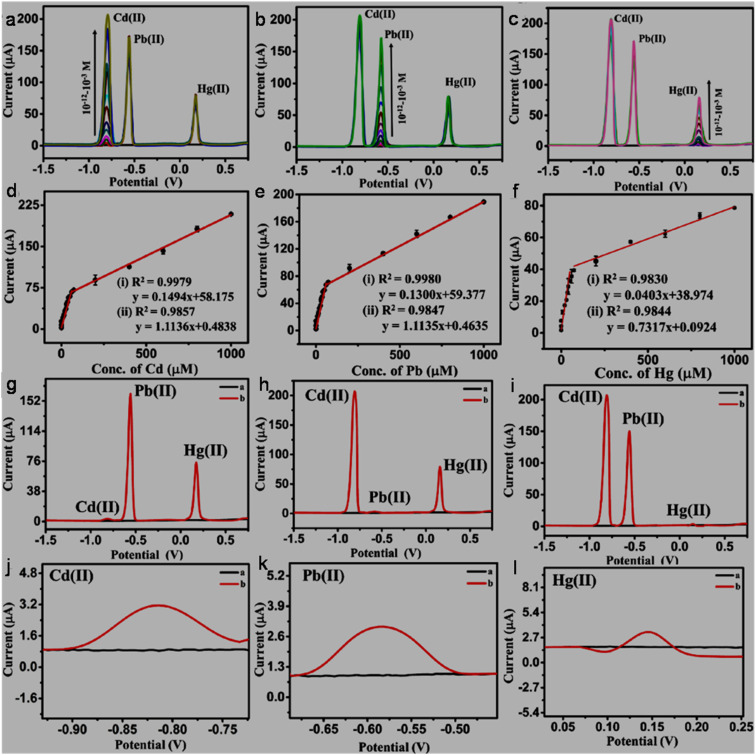
DPV signals of Cd^2+^ (a), Pb^2+^ (b) and Hg^2+^ (c) within the concentrations of 10^−12^ to 10^−3^ M in the presence of two other HMIs (1 mM). Plots of current *vs.* concentration of Cd^2+^ (d), Pb^2+^ (e), and Hg^2+^ (f). DPV signals of 0.1 M PBS, along with the minimum concentrations (LOD) of Cd^2+^ (g), Pb^2+^ (h), and Hg^2+^ (i). (j, k, l) Enlarged view of (g, h, i) showing LODs of 1/10/1 pM for Cd^2+^/Pb^2+^/Hg^2+^ during their simultaneous detection. Reprinted (adapted) with permission from ref. 198, copyright 2023, the American Chemical Society.

Later, Saisree *et al.*^198^ employed N,S-GQDs@GCE for the detection of three highly toxic HMIs (Cd^2+^/Pb^2+^/Hg^2+^) with good sensitivities (12/13/5 µA µM^−1^) and experimental LODs of 1/10/1 pM during single HMI sensing (Table 11). The modified-electrode was fabricated by the simple-drop casting of an N,S-GQDs dispersion on a freshly polished GCE. Benefitting from the improved conductivity and electrocatalytic activity of N,S-GQDs, HMIs are directly reduced to the corresponding metal species with a well-separated EC response (Fig. 29). The high current response in the DPV curves with different concentrations of Cd^2+^ (Fig. 30a), Pb^2+^ (Fig. 30b), and Hg^2+^ (Fig. 30c) in the presence of two other HMIs and the corresponding broad LRs in the two concentration ranges (Fig. 30d–f) exhibited the simultaneous detection capability of the modified-electrode. The minimum concentration analyses (Fig. 30g–l) indicated that the LODs in the simultaneous detection are equivalent to that of the individual HMI measurement. Moreover, good reusability (∼98% current response after 30 cycles), reproducibility (≤2% RSDs from five independent electrodes), stability (≥80% after 60 days), and ground/sea/waste water sample analytical capability (∼100%/≤ 0.5% recoveries/RSDs) are promising aspects of this sensor system.

#### 6.17.2. FL detection

##### 6.17.2.1. Undoped/doped-GQDs

Multiple HMIs are also detected by GQDs/doped-GQDs through the FL method (Tables 11 and S9). For instance, the fluorescence of starch-derived GQDs was quenched in the presence of Fe^3+^/Cu^2+^/Cr^3+^, and further recovered after the addition of group IIIA MIs (Al^3+^, gallium ion (Ga^3+^), and indium ion (In^3+^)), highlighting the cationic-controlled fluorescence switching behaviour of the GQDs due to the cation-driven changes in the π-conjugated region. The Ga^3+^ cation showed the highest fluorescence recovery of 67/81/70% after quenching operation with Fe^3+^/Cu^2+^/Cr^3+^. However, the authors could not quantify the sensitivity metrics.^294^ HT-synthesized N,S-GQDs (87.8% production yield) showed potential for the parallel detection of Fe^3+^, Cu^2+^, and Fe^3+^/Ag^+^ under different masking agents, namely, Cys, AA, and EDTA, respectively, with considerable sensitivities (Table 11). It was found that N,S-GQDs are more selective towards the three HMIs rather than undoped GQDs (containing abundant –OH groups). The –OH groups of N,S-GQDs are specific to interact with Fe^3+^, while the nitrogen-dopants of N,S-GQDs preferably interacted with Cu^2+^ and the electron-donating nature of N,S-GQDs reduced Ag^+^ to AgNPs. However, Ag^+^ detection required a longer incubation time (30 min) compared to Fe^3+^ (8 min) and Cu^2+^ (4 min) due to the contradictory HSAB interactions (N,S-GQDs: hard base, Ag^+^/Fe^3+^/Cu^2+^: soft acid/hard acid/moderate acid).^295^ The rapid synthesis of N-GQDs (∼10 min; 7.4% nitrogen content; 54% pyrrolic and graphitic nitrogen, 40% amine/pyridine/C_3_N_4_ configuration) using the plasma-contacting liquid (PCL) approach (Table 11) has become advantageous to incorporate plentiful oxygen/nitrogen-containing functional groups (both at the edges and on the surfaces) and numerous defect sites (Stone–Wales and trivacancy) for interaction with multiple HMIs. As a result, the N-GQDs showed capability for the successful detection of Fe^3+^, palladium ion (Pd^2+^), Hg^2+^, Cu^2+^, Pb^2+^, and Co^2+^ with good LRs (Table 11). PCL-synthesized N-GQDs showed a quenching effect with multiple HMIs (individual basis, minimum detectable concentration: 10 µM), showing the possibility to identify HMI-containing polluted water specimens; however, the authors did not demonstrate the simultaneous detection capability and practical utility of the probe.^296^

Recently, biomass (waste bean dregs)-derived N-GQDs were used in the FL method to determine two MIs (Ce^4+^ and Fe^3+^, Table 11). The fluorescence quenching-based identification capability of Ce^4+^ showed a broad LR (0–2000 µM) with the negotiation of larger LOD compared to previous reports on the detection of Ce^4+^ using chemical precursor-derived GQDs or N-GQDs (Tables 10 and S8). Meanwhile, this report did not provide the interference study, mechanistic investigation, and real-time applicability of the probe.^297^

**Fig. 31:**
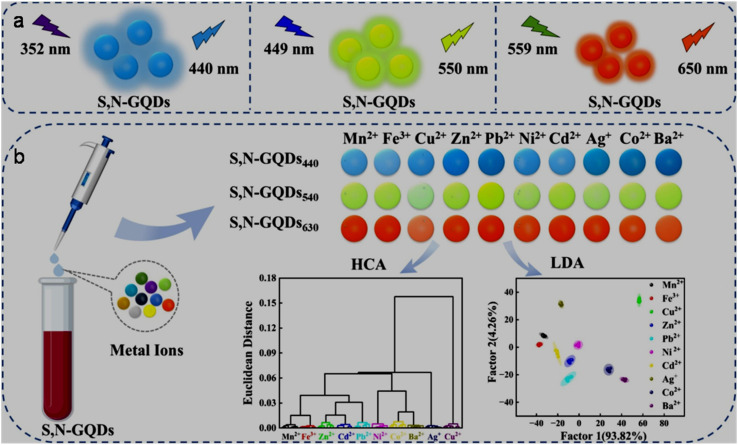
(a) Schematic of three colour (blue/green/red) emitting N,S-GQDs (a) and their application in the quantification and discrimination of 10 HMIs through LDA and HCA approach (b). Reprinted from ref. 299, copyright 2025, with permission from Elsevier.

Another very recent report utilized spent graphite (from waste lithium ion batteries) for its economical upcycling into crystalline N-GQDs (EIPL characteristic, QY: 11.04%). Their defective surface, small size (average size: 2.5 nm), and large amount of nitrogen-containing functionalities (nitrogen content: 2.67%; pyridinic and pyrrolic) facilitated active sites for the coordination of HMIs. Consequently, the N-GQDs could detect Fe^3+^ and Al^3+^ through the FL turn-off (98.4% quenching) and FL turn-on (38% enhancement) routes, respectively, and within the permissible limits according to the EPA (Table 11). The recovery tests of both HMIs in real water samples are satisfactory but the report lacks the detailed sensing mechanism.^298^ The distinct behaviours of the triple-colour emissive N,S-GQDs (440 nm (blue)/550 nm (green)/650 nm (red) emission at *λ*_ex_ = 352/449/559 nm, Fig. 31a; QY: 65.4/61.4/24.6% at 440/540/630 nm emission) with 10 HMIs (Mn^2+^, Fe^3+^, Cu^2+^, Zn^2+^, Pb^2+^, Ni^2+^, Cd^2+^, Ag^+^, Co^2+^, and Ba^2+^, Fig. 31b) were explored to differentiate these HMIs using linear discriminant analysis (LDA) and hierarchical cluster analysis (HCA) (Fig. 31b). The analytical method could successfully quantify Fe^3+^/Cu^2+^/Pb^2+^/Cd^2+^/Ni^2+^/Co^2+^/Mn^2+^/Zn^2+^/Ba^2+^/Ag^+^ at a minimum concentration of 0.50/0.11/0.55/2.10/1.14/1.14/2.03/3.92/0.96/0.29 µM, discriminated HMIs (Fe^3+^ and Cd^2+^) from mixtures, and showed applicability in environmental water bodies (tap and lake water) with satisfactory recoveries.^299^

##### 6.17.2.2. Functionalized GQDs

The utility of functionalized GQDs for the identification of multiple HMIs can be ascertained from Tables 11 and S9. For example, apart from Co^2+^ and Fe^3+^ recognition, Am–GQDs showed the FL detection of Pd^2+^ (first report) with an LOD of 657 nM. Complexation of the HMIs with the functional groups of Am–GQDs and cation–π interactions between them induced their aggregation, and consequently fluorescence quenching. However, the sensitivity/selectivity of this probe is very specific to its preparation conditions *via* gamma irradiation and a proper understanding is lacking.^300^ Pb-doping along with PEG-modification in biomass (cane molasses)-derived GQDs (PEG–Pb-GQDs) showed a significant enhancement in fluorescence intensity (QY: 30.31%) compared to bare GQDs or PEG–GQDs (QY: 10.44 or 21.32%). Consequently, the PEG–Pb-GQDs fluorescent probe selectively detected Fe^3+^, Cu^2+^, and Ag^+^ from a mixed HMI solution using EDTA + TU, F^−^ + SCN^−^ +Cl^−^, and EDTA + F^−^ masking agents, respectively, but with inferior sensitivities in comparison to the previous masking-based strategies using chemically synthesized N,S-GQDs probes (Table 11). Furthermore, the interference effect of Co^2+^, Ni^2+^, Mn^2+^, and Pb^2+^ on this probe (showed significant quenching) was not addressed.^301^ The introduction of specific functional groups such as Alizarine Red S and Eriochrome Black T on the surface of GQDs can be advantageous to develop simple COL sensors for the analysis of different HMIs according to colour variations (Table S9).

**Fig. 32:**
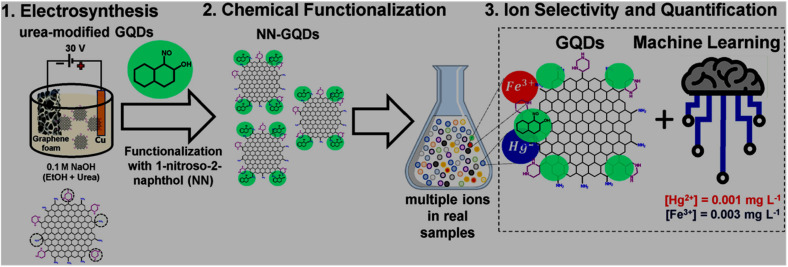
Step 1: synthesis of urea-modified GQDs *via* the electrolysis of graphene foam in urea-containing electrolyte. Step 2: covalent modification with NN chelating molecules to obtain NN–GQDs for the selective and sensitive detection of Hg^2+^ and Fe^3+^ in a mixed solution. Step 3: employing ML algorithm to improve the quantification of HMIs. Reproduced/adapted from ref. 302 with permission from The Royal Society of Chemistry, 2024.

Recently, Llaver *et al.*^302^ developed an ML-enabled algorithm using a functionalized GQDs nanoprobe to selectively detect two HMIs in a standard solution as well as in a complex real water matrix. A schematic illustration of the synthesis of the urea-modified GQDs *via* an electrochemical method, followed by their chemical-functionalization with 1-nitroso-2-naphthol (NN) to obtain the NN–GQDs fluorescent probe for the discrimination as well as quantification of Hg^2+^ and Fe^3+^ with the accreditation of MI algorithm is shown in Fig. 32. The distinct and intense emission from the NN–GQDs (456 nm at 326 nm *λ*_ex_) exhibited a slight blue-shift, along with a quenching effect in the presence of Fe^3+^ rather than only quenched fluorescence with Hg^2+^, enabling the basis of assimilation in the algorithm to discriminate and quantify these HMIs. Based on the data analyses, the LODs for Hg^2+^ and Fe^3+^ were predicted to be 1.0 (9.0) and 3.0 (8.0) µg l^−1^ under single HMI (in the presence of other HMIs), respectively, indicating a good merit of quantification and simultaneous detection possibility. Moreover, the ML model was successfully applied to predict the HMI contents simultaneously in natural (tap, river, and dam) water systems with an accuracy close to that measured from standard instrumental methods.

##### 6.17.2.3. GQDs involved with other counterparts

Systems are also constructed by combining functionalized/doped-GQDs with other counterparts for multi HMI recognition (Table 11). For example, DPA–GQDs-supported amino acids were applied to recognize Cu^2+^/Hg^2+^/Fe^3+^*via* the FL as well as COL method (LOD: 0.1 ppm). Cys amino acid is suggested to amplify the colour response in the COL detection process. As a result, a microfluidic paper-based platform (fabricated using DPA–GQDs and Cys) could recognize Cu^2+^/Fe^3+^ under natural light and quantify Cu^2+^ in environmental fluids/human urine samples.^303^ The quantification of Cu^2+^ and Cd^2+^ in a ratiometric manner with the assistance of N-GQDs and GSH-functionalized AuNCLs (GSH–AuNCLs) showed a satisfactory performance (Table 11). The fluorescence quenching/enhancement of GSH–AuNCLs with Cu^2+^/Cd^2+^ and insignificant effect on the fluorescence behaviour of N-GQDs resulted in the design of a sensitive ratiometric system. Apart from its recognition capacity in scallop samples, the fabricated paper-based platform using N-GQDs/GSH–AuNCLs showed possibility for the visual detection of both HMIs (distinct colour with Cu^2+^ and Cd^2+^ under UV light).^304^

*Summary*: The achievement of picomolar-level detection capability for multiple toxic HMIs (Pb^2+^, Hg^2+^, and Cd^2+^) in broad LRs without observable interference by the EC method using dual-doped N,S-GQDs is inspiring. FL methods also showed the simultaneous detection of multiple HMIs using GQDs-based platforms, but their selectivity is limited to the use of masking agents and their performance metrics are inferior in comparison to the EC method. N-GQDs and N,S-GQDs are effective probes compared to undoped GQDs for the efficient sensing of multiple HMIs through the FL method. Moreover, the creation of abundant defects and nitrogen/oxygen-containing covalent groups on GQDs are advantageous to achieve strong affinity with HMIs. The recent demonstration of LDA/HCA methods to discriminate multiple HMIs according to different colour responses with N,S-GQDs is notable. The functional group-enabled dissimilar interactions of functionalized GQDs with different HMIs can provide FL or COL detection platforms. Specifically, NN functional groups on GQDs can interact differently with Fe^3+^/Hg^2+^ to obtain a non-identical fluorescence outcome for the development of an ML algorithm and prediction of HMIs at very low concentrations with high accuracy. The ratiometric detection of Cu^2+^/Cd^2+^ with a binary system containing N-GQDs and GSH–AuNCLs is also considerable, which is extended to a paper-based device for the detection/discrimination according to different colour responses under UV light.

## 7. GQDs-based/involved sensors in the detection of anions

### 7.1. PO_4_^3−^

**Fig. 33:**
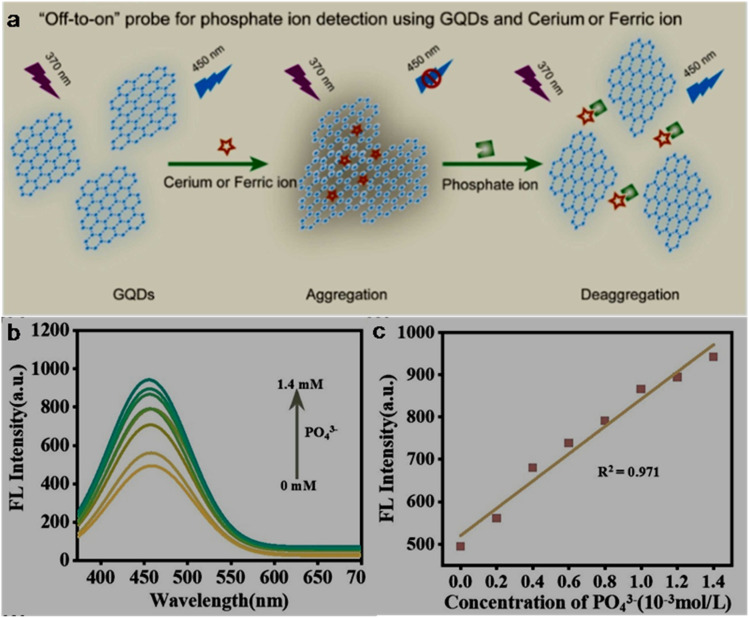
(a) Schematic showing the aggregation-based quenching of the fluorescence of GQDs in the presence of Ce^4+^/Fe^3+^ and further switch-on of their fluorescence due to the inhibition of their aggregation in the presence of PO_4_^3−^. Reprinted from ref. 305, copyright 2022, with permission from Elsevier. Fluorescence spectra (b) and corresponding fluorescence intensity plot (c) of N-GQDs/Ce^4+^ system according to different concentrations of PO_4_^3−^ (0–1.4 mM). Reprinted from ref. 297, copyright 2025, with permission from Elsevier.

The GQDs-based recognition of the PO_4_^3−^ anion can be accessed from Tables 12 and S10, where turn-off-on based strategies are the most common. The initial fluorescence quenching of GQDs/doped-GQDs is generally induced by rare-earth MIs or HMIs (Tables 12 and S10), but anion (Mo_7_O_24_^6−^)-mediated quenching has also been reported in the sensitive sensing of PO_4_^3−^ (Table S10). For example, biomass (corn straw)-derived GQDs showed fluorescence quenching with Ce^4+^/Fe^3+^*via* the AIQ mechanism and its recovery with PO_4_^3−^ due to the obstruction of their aggregation (Fig. 33a). The prominent recovery of the fluorescence with GQDs/Ce^4+^ rather than the GQDs/Fe^3+^ system in the presence of PO_4_^3−^ indicated the higher stability of the GQDs–Fe^3+^ complex. Consequently, the GQDs/Ce^4+^ system showed much better sensitivity in the detection of PO_4_^3−^compared with GQDs/Fe^3+^ (Table 12) and applicability towards the analysis of real water samples as well as the construction of paper-based sensors.^305^

**Table 12:** GQDs, modified-GQDs, and GQDs involved with other counterparts for anion sensing application^a^

GQDs-based sensor	Synthesis conditions	Size range/average size^b^ (nm)	QY (%)	Sensing process	LR (µM)	LOD (µM)	Ref.
**PO**_**4**_^**3**^ **^−^**							
GQDs/Ce^4+^	HT (Corn straw powder in water, 170 °C, 12 h); centrifugation; filtration	1.5–4/2.67	15.65	FL, turn-off-on	0.1–2, 2–20	0.06	305 l ^,^ m
GQDs/Fe^3+^				"	0.1–1.4	0.09	305 l
N-GQDs/Ce^4+^	HT (Bean dregs power in water, 180 °C, 12 h); filtration; dialysis	0.38–3.74/1.63	21.3	FL, turn-off-on	0–1400	—	297

**P**_**2**_ **O**_**7**_^**4**^ **^−^**							
N,S-GQDs/Fe^3+^	Pyrolysis (CA/GSH, 200 °C, 15 min); dissolved in water and pH adjusted to 5.0; dialysis	<8/3	36.3	FL, turn-off-on	1–1000	0.81	306 l

**ClO^−^**							
DAP–GQDs	Acid oxidation of graphite flake with HNO_3_:H_2_SO_4_ (1 : 3) under ultrasonication (2 h) and reflux (120 °C, 24 h); pH adjusted to 7.0; filtration; dialysis; covalent modification with DAP	1–5/2.9	13.4	FL, turn-off	0–8	0.0126	130 l ^,^ n

**S** ^**2**^ **^−^**							
Eu-GQDs/ZIF-8	ST (GO/EuCl_3_·6H_2_O in DMF, 200 °C, 7 h); gel permeation chromatography; non-covalent adsorption on ZIF-8; centrifugation	<10/—c	—	FL, turn-on	0–600^f^	0.12^k^	307

**CN^−^**							
GQDs@ZIF-11	Pyrolysis (CA, 200 °C, 15 min); mixed in 10 mg per mL NaOH solution and pH adjusted to 7.0; *in situ* encapsulation in ZIF-11	2.5–8/∼5.2^c^	27^d^	FL, turn-off	0.15–30	0.0145	308 l
N-GQDs/Ag^+^	HT (CA/tris(hydroxymethyl)-aminomethane in water, 205 °C, 2.5 h); dialysis	1–12/5.4	57.9^e^	FL, turn-off-on	0.5–25^g^	0.08^g^	309 l ^,^ m

**NO**_**2**_ **^−^(FL sensor)**							
N,P-GQDs	HT (THPC/PEI-EC in water, 230 °C, 8 h); pH adjusted to 7.0; dialysis	1.5–7.5/4.2	9.4^e^	FL, turn-off	0.005–0.03	0.0025	310 n
N-GQDs	Pyrolysis (Onion slice, 220 °C, 4 h, N_2_); ST (obtained solid in DMF/H_2_O, 190 °C, 4 h); dialysis	<15/10	15.7	FL, turn-off	0.3–1400	0.1	311

**NO**_**2**_ **^−^(EC sensor)**							
GQDs/PCN-222 @FTO	Ar/DC microplasma treatment of starch in 0.1 M NaOH aqueous solution, 1 h; filtration; impregnated in mesoporous PCN-222; drop-casted on FTO	1.5–5/3.1^c^	—	EC, Amp	40–18000	6.4	312
CoPc/GQDs@ GCE	HT (CA/NaOH in water, 160 °C, 4 h); centrifugation, dialysis; non-covalently conjugated with CoPc; drop-casted on GCE	2–6/∼3.5^c^	—	EC, ChAmp	0–1000	0.17	313
CoPc/N-GQDs @ GCE	HT (CA/urea in water, 160 °C, 4 h); centrifugation, dialysis; non-covalently conjugated with CoPc; drop-casted on GCE	2–5/∼3.2^c^	—	"	0–1000	0.25	

**I^−^**							
N,S-GQDs/Ce^4+^	Pyrolysis (CA/Cys, 200 °C); diluted with water	2–4/—	85.6	FI-CL, turn-off	0.04–3	0.00423	192 l

**F^−^**							
Gd^3+^-loaded PEG–GQDs	Purchased from CASYUEDA materials Technology	2–7.2/4.2	—	ULF-NMR relaxometry	0.01–100	0.01	314 l

**SCN^−^**							
GQDs/AuNPs hybrid	Chemical oxidation of graphite powder with KMnO_4_/H_2_SO_4_; HT (obtained solid/TSC in water, 150 °C, 2 h); filtration; dialysis; GQD-assisted synthesis of AuNPs	3–5/—c	9.6^d^	COL, turn-on	0.01–0.1	0.003	315 l

**Other anions**							
N-GQDs/I_2_	ST (julolidine/acetic acid in ethanol, 200 °C, 12 h); filtration; dialysis	—/4.8	53	FL, turn-off-on	0.002–0.01^h^	9.3 × 10^−5^^h^	316 l ^,^ n
Cy5.5–N-GQDs	Acid oxidation of graphene with HNO_3_:H_2_SO_4_ (1 : 4) under reflux (90 °C, 10 h); diluted with water and pH adjusted to 7.0; filtration; covalently modified with Cy5.5 dye	1–5/3.5^c^	11.6	FL, ratiometric	0–6^i^	0.03^i^	317 n
OH-GQDs/PPy-Br	HT (TNP in 0.2 M NaOH aqueous solution, 200 °C, 10 h); filtration; dialysis; non-covalently conjugated with PPy-Br dye	<5/—c	21	FL, ratiometric	0.1–2^j^	0.036^j^	141 l ^,^ n

a THPC: tetrakis(hydroxymethyl)phosphonium chloride, PEI-EC: ethylenediamine-end-capped-polyethylenimine.

b Measured from TEM.

c Size range/average size of GQDs involved with other counterparts.

d QY of GQDs involved with other counterparts.

e Absolute QY.

f LR in µL of 1 ppm analyte.

g LR/LOD in µg mL^−1^ from paper-based sensor.

h LR/LOD in the detection of S_2_O_3_^2−^.

i LR/LOD in the detection of ONOO^−^.

j LR/LOD in the detection of HSO_3_^−^.

k LOD in ppm.

l Analytical ability in real samples.

m Paper-based sensing capability.

n Analytical ability in living cells.

Recently, PO_4_^3−^ was again sensed with the same strategy using a biomass (Bean dregs)-derived N-GQDs/Ce^4+^ system *via* the gradual enhancement of its fluorescence intensity as the PO_4_^3−^ concentration increased step-by-step (Fig. 33b) and exhibited a very broad LR (0–1400 µM, Fig. 33c) in comparison to previous results (Tables 12 and S10).^297^

*Summary*: The turn-off-on-based strategy is the most common for the detection of PO_4_^3−^, where the quenching of the fluorescence of GQDs or doped-GQDs with cheap Ce^4+^ rather than costly rare-earth cations (Eu^3+^ and Dy^3+^) is notable. Among the doped-GQDs, N-GQDs can be employed as an efficient probe for the sensing of PO_4_^3−^. Moreover, the effective detection capability for PO_4_^3−^ using biomass-derived GQDs or N-GQDs is considerable.

### 7.2. P_2_O_7_^4−^

The turn-off-on-based sensing attributes for P_2_O_7_^4−^ using doped-GQDs can be revealed from Tables 12 and S10. For instance, the Fe^3+^-mediated fluorescence quenching of N,S-GQDs and its recovery in the presence of P_2_O_7_^4−^ (Fe^3+^ possesses high affinity for P_2_O_7_^4−^ rather than the nitrogen/sulphur-containing functional groups of N,S-GQDs) avoided the use of expensive Eu^3+^ (used earlier, Table S10) and demonstrated a wide LR of 1–1000 µM (LOD: 810 nM) in the detection of P_2_O_7_^4−^. Moreover, a clinical diagnosis of arthritis is possible with the sensor due to the P_2_O_7_^4−^ assay in human synovial fluid.^306^

*Summary*: N,S-GQDs have shown better potential compared to single-heteroatom doped N-GQDs in the turn-off-on-type FL detection of P_2_O_7_^4−^. Moreover, fluorescence quenching with cheap Fe^3+^ rather than the costly Eu^3+^, and subsequent P_2_O_7_^4−^-driven recovery is an applicable sensing approach to attain a reasonable selectivity/sensitivity for P_2_O_7_^4−^.

### 7.3. Hypochlorite (ClO^−^)

**Fig. 34:**
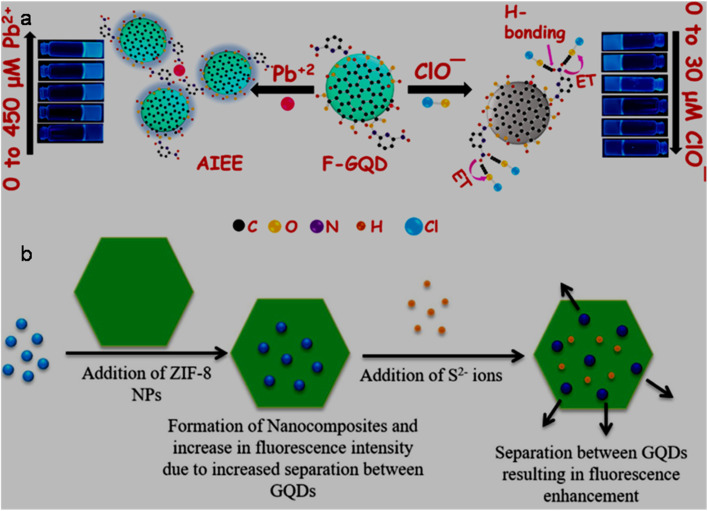
(a) Schematic of the AIEE-based sensing of Pb^2+^ and hydrogen-bonding/energy transfer-induced turn-off detection of ClO^−^. Reprinted (adapted) with permission from ref. 130, copyright 2021, the American Chemical Society. (b) Schematic of the sensing process of S^2−^ using Eu-GQDs confined in a ZIF-8 framework. Reprinted from ref. 307, copyright 2017, with permission from Elsevier.

GQDs-based systems have been successfully used for the sensing of ClO^−^*via* FL, CL, and COL methods (Tables 12 and S10). For instance, the CL signal produced by the oxidation of GQDs with ClO^−^ greatly increased (∼18-fold) in the presence of cetyltrimethylammonium bromide, which enabled the detection of ClO^−^ in a wide LR (Table S10). Later, edge-functionalized GQDs (DAP–GQDs) exhibited acceptable sensitivity (Table 12) in the identification of ClO^−^. The nanoprobe triggered AIEE-based turn-on behaviour with Pb^2+^, while a turn-off response to ClO^−^ with the participation of hydrogen-bonding-induced energy transfer (Fig. 34a). The presence of amine moieties in DAP favoured the formation of a hydrogen-bonded framework between DAP–GQDs and O–Cl. Consequently, the probe and analyte reached in close proximity, resulting in little aggregation and energy migration. The recoveries of 92–123% for ClO^−^ in tap/lake water samples and the monitoring of exogenic ClO^−^ in HeLa cells justified the practicability of this sensor.^130^

*Summary*: Although ClO^−^ can trigger CL enhancement or fluorescence switch-off of bare GQDs/modified-GQDs, the utility of functionalized GQDs and FL method for the purpose of ClO^−^ sensing is advantageous to achieve nanomolar-level sensitivity. Nitrogen-rich functionalities (specifically, DAP) on GQDs have shown capability for the selective and sensitive detection of ClO^−^*via* an energy transfer process.

### 7.4. S^2−^

GQDs-based sensors have also been fabricated to monitor toxic S^2−^ (Tables 12 and S10). Besides the previous turn-off-on-based approach (Table S10), Sammi *et al.*^307^ explored a new approach to sense S^2−^ with a satisfactory performance (Table 12). The non-covalent host–guest interaction between Eu-GQDs and zeolitic imidazole framework (ZIF-8) NPs significantly increased the fluorescence intensity of the Eu-GQDs/ZIF-8 nanocomposite due to the better separation/dispersion of Eu-GQDs in the host matrix (Fig. 34b). After the addition of S^2−^, the separation of Eu-GQDs became more prominent in ZIF-8, resulting in the further intensification of fluorescence and analysis of S^2−^*via* the turn-on mode.

*Summary*: The turn-on detection of S^2−^*via* the host–guest interaction between metal-doped GQDs (Eu-GQDs) and ZIF-8 is an effective sensing approach. Additionally, the S^2−^ sensing performance of dual-functionalized GQDs (SA, GSH–GQDs; SA: sulfanilic acid) *via* Cu^2+^-mediated fluorescence quenching and S^2−^-driven recovery is also considerable.

### 7.5. Cyanide (CN^−^)

Various sensing platforms have been constructed with the participation of GQDs/doped-GQDs to monitor CN^−^ (Tables 12 and S10). For instance, a GQDs-encapsulated ZIF-11 (GQDs@ZIF-11) composite was utilized for the selective/sensitive detection of CN^−^ with a low LOD (14.5 nM). Here, CN^−^ effectively suppressed the interaction between the functional groups of GQDs and benzimidazole moiety present on ZIF-11 to turn-off the fluorescence signal. Moreover, the detection of CN^−^ in real samples (apple seeds/bitter almonds) was also accomplished by this probe with reasonable recoveries (96.7–102.7%).^308^

**Fig. 35:**
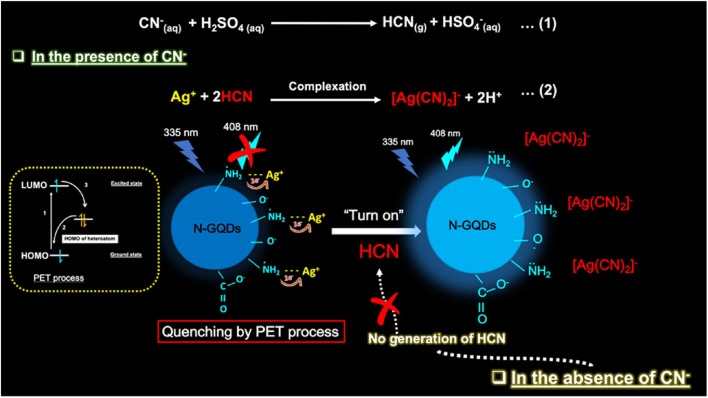
Schematic of the possible mechanism involved in the detection of CN^−^ using an N-GQDs-containing test paper kit. Reprinted (adapted) with permission from ref. 309, copyright 2023, the American Chemical Society.

Malahom *et al.*^309^ developed a fluorescent paper-based test kit using N-GQDs (absolute QY: 57.9%) for the selective quantification of CN^−^ with considerable sensitivity (Table 12). A schematic representation of the turn-off-on-based detection process is shown in Fig. 35, where the fluorescence signal of N-GQDs is quenched by Ag^+^*via* the PET mechanism, followed by switched-on behaviour in the presence of CN^−^ (production of HCN and [Ag(CN)_2_]^−^ complex formation according to eqn (1) and (2), respectively, Fig. 35) *via* the leaving-off of Ag^+^ from the N-GQDs surface. Additionally, the fabricated kit was found to be a promising analytical tool for the quantification of CN^−^ in real juice/food samples (recoveries: 102.6–109.3/97.1–109.4%), along with satisfactory storage capability (30 days) and inter-/intra-day precision below 2% RSD.

*Summary*: Both N-GQDs and N,S-GQDs are applicable to detect CN^−^ with the involvement of Ag^+^ or AgNPs; however, the construction of portable paper kits using N-GQDs in the CN^−^ detection process up to real sample level is a good attempt. Additionally, the compositing of GQDs with a ZIF moiety can also build a selective/sensitive platform for CN^−^.

### 7.6. NO_2_^−^

#### 7.6.1. FL detection

Although a 2.5 nM LOD was achieved for NO_2_^−^ (first report) using N,P-GQDs, this probe is limited to a very narrow concentration range (Table 12). Electron transfer between the –NH_2_ groups of N,P-GQDs (nitrogen/phosphorus content: 5.85/8.71%) and NO_2_^−^ quenched the inherent fluorescence of N,P-GQDs (incubation time: 20 min). Moreover, the fluorescence imaging-based detection of NO_2_^−^ in living cells (T24 cells) using N,P-GQDs demonstrated their intracellular detection possibility.^310^ Later, a similar interaction/electron transfer-based quenching mechanism using N-GQDs (onion biomass-derived) could detect NO_2_^−^ in a broad LR with satisfactory LOD (Table 12). However, effective quenching activity with this probe required a relatively longer interaction time (30 min) compared to previous reports.^311^

#### 7.6.2. EC detection

The EC method is also used in the detection of NO_2_^−^ using GQDs involved systems (Tables 12 and S10). For instance, GQDs were directly impregnated into a porphyrinic zirconium-based metal–organic framework (PCN-222) to construct a GQDs/PCN-222 mesoporous structure, which exhibited 100-times higher electrical conductivity (9 × 10^−11^ S cm^−1^) in comparison to bare PCN-222 (6 × 10^−13^ S cm^−1^). As a result, the amperometric (Amp) response of GQDs/PCN-222@FTO (FTO: F-doped tin oxide) showed a superior EC sensing performance for NO_2_^−^ (Table 12) in comparison to PCN-222@FTO (LR/LOD: 200–20 000/50 µM). The response current in the EC detection is the result of NO_2_^−^ oxidation, which is effectively electrocatalyzed by the GQDs involved composite material.^312^ Subsequently, Ndebele *et al.*^313^ conjugated GQDs or N-GQDs (*via* π stacking) with tris(4-*tert*-butylphenoxy)-(5-phenoxylpicolinic acid)phthalocyanato cobalt(ii) (CoPc) to obtain two electrocatalyst materials, namely, CoPc/GQDs and CoPc/N-GQDs, respectively, for the chronoamperometric (ChAmp)-based EC detection of NO_2_^−^ with improved LODs in comparison to previous reports (Tables 12 and S10). The GQDs/N-GQDs significantly magnified the electrocatalytic activity of CoPc for a better EC response. The electrode materials were also tested for 20 consecutive cycles (≤10% reduction in peak currents) to access their satisfactory stability.

*Summary*: The existence of nitrogen-containing functional groups in doped-GQDs is favourable to interact with NO_2_^−^ and execute electron transfer for the quenching of their fluorescence signal. Although the N,P-GQDs achieved the nanomolar-level detection of NO_2_^−^, the achievement of a wide sensitivity range with N-GQDs is noticeable. The sensitivity in the EC detection of NO_2_^−^ using conjugate systems (GQDs or N-GQDs with CoPc counterparts) is also considerable. GQDs-based/involved systems can effectively sense NO_2_^−^ through the FL as well as EC method.

### 7.7. Halide ions

#### 7.7.1. I^−^

The initial detection of I^−^*via* the FL turn-off-on approach using the N-GQDs/Ag^+^ system (Table S10) showed the possibility to utilize GQDs-based systems for this purpose. As a result, the sensing of I^−^ was performed again with an entirely new flow-injection CL (FI-CL) method (Table 12). The redox reaction between N,S-GQDs (nitrogen/sulfur content: 5.31/5.12%) and Ce^4+^ under acidic pH produced a strong CL signal, which was quenched by I^−^ and exhibited a good LOD (4.23 nM) in the detection process. The production of the redox reaction energy required for the excitation of N,S-GQDs facilitated the generation of a strong CL response. Moreover, this system showed applicability to determine I^−^ in kelp/tea samples. However, although the detection of I^−^*via* the FI-CL approach resulted in a good performance, its complicated setup and precise sensing operation cannot be ignored.^192^

#### 7.7.2. Fluoride (F^−^)

Besides the turn-off-on-based FL method to detect F^−^ using the B,N-GQDs/Hg^2+^ platform with low sensitivity (Table S10), Li *et al.*^314^ developed a highly sensitive ultra-low-field NMR (ULF-NMR, 118 µT) relaxometry method to sense F^−^ with a low LOD of 10 nM in a wide LR (Table 12). An aqueous solution of magnetic GQDs (Gd^3+^-loaded PEG–GQDs) showed a decrease in relaxation time with an increase in the concentration of F^−^. Here, the probe-F^−^-selective coordination and localized polarization/ionization effect of the surrounding water facilitated the proton exchange rate and relaxivity in the sensing operation, which is evidenced by the large negative electrostatic potential of the probe in the presence of F^−^ (−304.1 kJ mol^−1^ with F^−^ and −52.7 kJ mol^−1^ without F^−^). Moreover, this sensor probe was found to be reliable for the analysis of F^−^ in domestic water samples.

*Summary*: Doped-GQDs, particularly, N-GQDs and N,S-GQDs have shown potential to detect I^−^ with considerable selectivity/sensitivity through the FL and FI-CL approach, respectively. Between these two sensing methods, the FL turn-off-on route is straightforward and user friendly. The selective and reasonably sensitive identification of F^−^ through the NMR relaxometry technique by utilizing the magnetic nature of Gd^3+^-loaded PEG–GQDs is recognizable.

### 7.8. SCN^−^

After the qualitative demonstration of the recovery of fluorescence in the presence of SCN^−^ using a quenched system (GQDs/Hg^2+^) (Table S10), the quantitative detection of SCN^−^ was realized with a GQDs/AuNPs hybrid. The oxidation of TMB by scavenging more ˙OH radicals and growth of small-sized AuNPs in the presence of SCN^−^ collectively boosted the peroxidise-like nanozymatic activity to intensify the blue colour (from TMB_o*x*_), and therefore COL sensing of SCN^−^ with a low LOD (3 nM) in a narrow LR (Table 12). Moreover, the detection of SCN^−^ in spiked-milk samples showed a convenient LOD of 8 nM and recoveries/RSDs of 93.8–104.8%/<4.0%.^315^

*Summary*: The COL detection of SCN^−^ with GQDs/AuNPs hybrids is a considerable sensing activity, which dictates the utility of GQDs involved systems in the selective-sensitive detection of SCN^−^.

### 7.9. Other anions

GQDs-based systems have also been used for the sensing of persulfate (S_2_O_8_^2−^), sulfite (SO_3_^2−^), ferricyanide (Fe(CN)_6_^3−^), ethyl xanthate (EtX^−^), thiosulfate (S_2_O_3_^2−^), peroxynitrite (ONOO^−^), and bisulfite (HSO_3_^−^) anions (Tables 12 and S10). IL@GQDs showed selective fluorescence quenching with Fe(CN)_6_^3−^, and consequently Fe(CN)_6_^3−^ detection capability in a standard aqueous solution as well as in real river water samples (Table S10). The anion exchange nature of the IL (contains BF_4_^−^) favoured the ferricyanide anion to reach close to the probe for effective interaction and quenching-based analytical activity. However, the requirement of a long equilibrium time (30 min; for significant quenching) and interference effect from Fe^3+^ (suggested to manage by AA) are some drawbacks in this sensing approach. The association of IFE suppressed the fluorescence intensity of N-GQDs with I_2_; however, the iodimetry reaction between S_2_O_3_^2−^ and I_2_ caused weakening of IFE to switch-on the fluorescence signal. As a result, this probe could achieve a low LOD for S_2_O_3_^2−^ (0.093 nM) in a narrow LR (Table 12) and practical utility in real water/biological fluid (human blood, serum, and urine) samples as well as in RAW cells. However, although this probe is reusable for at least up to 7 repeated cycles without much compromise in its selectivity-sensitivity, the interferences from S^2−^ and SO_3_^2−^ cannot be neglected.^316^ FRET between N-GQDs and cyanine 5.5 (Cy5.5) in the covalently functionalized Cy5.5–N-GQDs, and furthermore the disappearance of FRET after the addition of ONOO^−^ built a ratiometric probe for the sensitive detection of ONOO^−^ (Table 12). However, the selectivity result of the sensor is missing in this report. This probe could be internalized in the mitochondria of living cells (Cy5.5 possesses mitochondria-targeting groups) to ratio metrically image exogenous as well as endogenous ONOO^−^.^317^

**Fig. 36:**
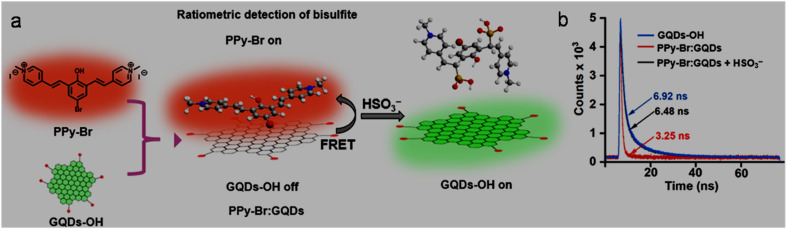
(a) Schematic of the ratiometric detection of HSO_3_^−^ using the OH-GQDs/PPy-Br FRET conjugate system. (b) Time-resolved fluorescence decay curves of OH-GQDs, OH-GQDs/PPy-Br, and OH-GQDs/PPy-Br in the presence of HSO_3_^−^. Reprinted (adapted) with permission from ref. 141, copyright 2023, the American Chemical Society.

Subsequently, a similar type of ratiometric detection was realized for HSO_3_^−^ (LOD: 36 nM, Table 12) using a nanoconjugate system containing a red-emissive dye (PPy-Br, *λ*_em_: 750 nm) and green-emissive OH-GQDs (*λ*_em_: 535 nm). Fig. 36a illustrates the sensing process, where the FRET between the dye molecules and OH-GQDs enhanced/diminished the corresponding red/green fluorescence, and furthermore switch-off (due to the formation of non-fluorescent Michael adducts)/switch-on (termination of FRET) behaviour in the presence of HSO_3_^−^. The FRET-involved sensing mechanism was validated by time-resolved fluorescence spectra (reduction of OH-GQDs *τ*_av_ from 6.92 to 3.25 ns after conjugation with PPy-Br, and further increase to 6.48 ns after the addition of HSO_3_^−^, Fig. 36b). Moreover, the good recovery range of 95–100% in biological samples and ratiometric monitoring of HSO_3_^−^ in MDA-MB-231 living cells confirmed the practical utility of this sensor.^141^

*Summary*: Undoped GQDs/N-GQDs have shown potential to detect many other inorganic anions. Specifically, the FRET–weakened FRET-type FL turn-off-on process in a ratiometric manner (with GQDs or N-GQDs) may result the sensitive detection of ONOO^−^/HSO_3_^−^ anions, even up to the living cell tracking level. The high selectivity/sensitivity in the turn-off detection of Fe(CN)_6_^3−^ using IL@GQDs is also a considerable achievement.

## 8. Conclusion and future prospects

A variety of readily available precursors (from bulk carbon to common laboratory chemicals/biomass) has made it viable to attain the facile and low-cost synthesis of GQDs and their modified-derivatives. The experimental parameters, starting precursors, and post-modifications played crucial roles in attaining various physiochemical characteristics, compositional/structural modulations, and tuneable photo-physical properties in the GQDs/modified-GQDs. Consequently, GQDs/modified-GQDs have gained the necessary enhancement in optical features, electronic conductivity, and electrocatalytic activity for analytical purposes. Microplasma (at room temperature) and atmospheric pressure reflux synthesis are representative examples that showed feasibility to convert green precursors (such as chitosan, starch, and lignin) and economical aromatic compounds (phloroglucinol; nearly 100% conversion) into high-quality GQDs/doped-GQDs. The presence of nitrogen-heteroatoms in the lattice as well as in the form of amino/amido functional groups at the edge/surface of GQDs is found to be supportive for the nanoprobing of cationic MIs. Moreover, some amount of sulfur or boron along with nitrogen is also favourable to prepare N,S-GQDs or B,N-GQDs as effective probes for MIs.

Benefitting from their favourable features, along with limitations, GQDs/modified-GQDs and many of the platforms that involve GQDs/modified-GQDs have been shown as examples to selectively and sensitively detect many types of MIs and anions (brief summary presented after the discussion of each ions). Broadly speaking, electron density/functional groups enriched N-GQDs and N,S-GQDs have shown significant opportunities to selectively interact with many positively charged MIs, resulting in a sensitive detection performance. The sulfur-containing functional groups in GQDs can have specific affinity with Hg^2+^ and Pb^2+^. Also, additional functional moieties on functionalized GQDs such as DMC, Am, DA and crown ether exhibited specificity in the sensitive quantification of Hg^2+^, Cu^2+^, Ca^2+^, and alkali MIs, respectively. Conversely, negatively charged anions are frequently detected using GQDs-based/involved systems through a mediation process. GQDs-based/involved systems not only specifically interacted with inorganic ions to alter their inherent fluorescence signals (FL-based detection) but also emerged as a superior platform for EC, ECL, and COL detection. The sensing of inorganic ions through ratiometric design exhibited better sensitivity, reliability, and accuracy compared to analyzing *via* single-signal (emission or ECL response) upturn/downfall. Recent developments have enabled the quantification of hazardous HMIs such as Hg^2+^, Cd^2+^ and Pb^2+^ using GQDs-based platforms to sub-nanomolar/picomolar magnitudes, and even in the presence of other interfering analytes. Moreover, the detection of HMIs by fabricated test-paper strips, hydrogel kits, and aerogel pellets through simple smart-phone captured image analyses, assimilation of LDA/HCA to distinguish multiple HMIs, selectivity/tracking of bio-relevant alkali/alkaline-earth MIs in living cells, realization of worthwhile selectivity-sensitivity for rare-earth/radioactive MIs, integration of ML for the accomplishment of nanomolar-level accuracy, and sensing capability of various anions are some of the notable results.

However, although the sensing of inorganic ions by GQDs-based/involved systems has gained significant attention in the past, it is still in the developing stage, opening an opportunity for new and deeper-level research. Some of the foreseeable challenges/prospects where attention may be paid for a bright future are as follows:

(1) Obtaining nearly monodispersed GQDs with precise control over the number of layers and chemical composition is vital for understanding their structure–property relationship. Here, the choice of precursors and fine-tuning of the experimental conditions need further improvement, especially through the bottom-up approach.

(2) There are a few examples where red-/NIR-emissive or UCPL-featured GQDs/modified-GQDs have been explored for the detection of inorganic ions. Considering the importance of these GQD structures (particularly, owing to their high brightness, easy penetration capability in biological components, and low background effect), their easy/repeatable production with sufficiently high QY may be a suitable choice especially for application in living systems.

(3) It is surprising why GQDs/modified-GQDs with similar compositions and structures (synthesized using different processes/conditions/precursors) show selectivity with different HMIs and a range of sensitivity metrics with a particular HMI. Therefore, much work is required to divulge the genesis of the selectivity and sensitivity of GQDs-based systems with molecular-level observations, and also with the assistance of advanced algorithms-artificial intelligence.

(4) Surface functionalities, heteroatom-doping in their lattice, and active surface of GQDs are crucial for their selective interactions with inorganic ions. Moreover, fine-tuning of the functional groups at the edge rather than on their surface or *vice versa*, controllable sp^2^/sp^3^ carbon content, tunable defects/vacancies in their structure, and incorporation of chirality features may give some fruitful insights about their specificity/sensitivity mechanism for inorganic ions.

(5) GQDs-based platforms have shown promising relevance for the selective/sensitive detection of bio-related alkali/alkaline-earth MIs. Recently, Ca^2+^ detection/monitoring in different cell lines *via* functional group (DA)-mediated uncommon turn-on fluorescence has prompted researchers to develop higher wavelength-emitting GQDs/modified-GQDs for the purpose of safe bio-implantation.

(6) The NMR-based relaxometry detection of K^+^ (alkali MI) and F^−^ (anion) inspired the exploration of a new sensing approach for inorganic ions. However, the employment of GQDs-containing probes with toxic/expensive Gd^3+^ cannot be ignored. Therefore, the fabrication of NMR-active sensor systems by creating a paramagnetic center in GQDs-based probes with a benign element may be an interesting task.

(7) It is worthwhile to combine GQDs/modified-GQDs with other functional counterparts (*e.g.*, LDH, PB analogues, metal–organic frameworks, and covalent-organic frameworks) at the molecular level to improve their chemical/mechanical stability and optical–electronic properties for the fabrication of advanced and reliable sensor devices.

(8) The well-dispersed and *in situ* implantation of GQDs/modified-GQDs in an interlocked polymeric matrix (smart wearable hydrogels/aerogels) can furnish self-transportation channels for the selective/rapid migration of inorganic ions. Therefore, the development of highly porous and low-density platforms (with high stability, easy processability, degradability, and tolerance against harsh environments) for the sensing of inorganic ions is worthwhile and should be explored.

(9) According to the available literature, the detection of toxic Cr^6+^/Cr^3+^, As^3+^, alkali/alkaline-earth MIs, rare-earth/radioactive MIs, and many anions is at the very early state of investigation, and thus there is much hope in the search of GQDs-based/involved advanced platforms, especially suitable using the EC, ECL, and COL sensing methods.

(10) The simultaneous detection/discrimination of multiple HMIs using GQDs-based/involved systems is another area of expansion, which should pay special attention to meet the requirement of robust sensors in real complex systems. Here, ML- and LDA/HCA-enabled semiempirical quantification with the requirement of minimum experimental data is showing a new future direction.

(11) Most inorganic anions are detected by GQDs-based systems through the involvement of mediating steps (because of their similar surface charge). Therefore, post-functionalization of GQDs/doped-GQDs with different cationic moieties may enable them to directly interact with selective anions and sense them with a high performance output.

## Conflicts of interest

There are no conflicts to declare for financial interests or personal relationships.

## Supplementary Material

RA-015-D5RA04935K-s001

## Data Availability

Data availability statement is not applicable as no new data were generated or analyzed in this study. Supplementary information is available. See DOI: https://doi.org/10.1039/d5ra04935k.
