# Supplementary material for: A comprehensive review on the capability of graphene quantum dots-based/involved platforms for the detection of inorganic ions
Source: RSC Adv. 2025 Nov 24;15(54):46079–151. doi: 10.1039/d5ra04935k (PMC12642511; doi:10.1039/d5ra04935k)
Supplement: RA-015-D5RA04935K-s001 [file RA-015-D5RA04935K-s001.pdf]

Supplementary Information (SI) for RSC Advances.
This journal is © The Royal Society of Chemistry 2025

A comprehensive review on the detection capability of inorganic ions by graphene quantum dots-based platforms

Prashant Dubey

Centre of Material Sciences, Institute of Interdisciplinary Studies (IIDS), University of Allahabad, Prayagraj-211002, Uttar Pradesh (INDIA)

Email: pdubey@allduniv.ac.in, pdubey.au@gmail.com, ORC id: 0000-0001-8159-6528

Table S1. GQDs, modified-GQDs, and GQDs-involved with other counterparts for Fe^{3+} and Fe^{2+} sensing application.

GQD-based sensor	Synthesis condition	Size distribution/ average size (nm)[a]	QY (%)	Sensing process	LR (μM)	LOD (μM)	Ref.
**Fe^{3+}**							
**Doped-/undoped GQDs**							
N-GQDs	Electrolysis of 3D Graphene in $BMIMPF_6$/acetonitrile; Centrifugation; Dialysis	1–5/3	10	FL, Turn-off	0–80	7.22	[1]
N-GQDs	HT (CA/25wt% NH_3 in water, 200°C, 3h); pH adjusted to 8.0; Dialysis	1–8/3.5	30.7	FL, Turn-off	0–10	—	[2]
N-GQDs	HNO_3 vapor cutting of MOF-derived nitrogen rich porous carbon (ZIF-8C), 160°C, 5h; *In-situ* filtration	1.3–2.7/2	—	FL, Turn-off	1–70	0.08	[3][f]
N-GQDs	MW (Aspartic acid/NH_4HCO_3 in water, 560W, 10min); Dialysis	1.8–2.4/2.1	14	FL, Turn-off	0–50	0.26	[4]
N-GQDs	HT (GO/$NH_3.H_2O$ in water, 120°C, 6h); Filtration; HT (Supernatant/THF, 100°C, 2h); THF portion separated by adding $(NH_4)_2SO_4$, dried & dispersed in water	5–10/6	—	FL, Turn-off	0.2–30	0.06	[5][f]
N-GQDs	HNO_3 vapor cutting of mesoporous nitrogen/carbon/silica composite (N/C/SBA-15), 150°C, 3h; *In-situ* filtration	1.6–3.5/2.4	—	FL, Turn-off	0.5–40	—	[6][f]
N-GQDs	MW plasma CVD (C_{60}, 2.45GHz, 800W, 3kPa, 440°C, 15min, N_2)	2–6.5/4.5	7.4	FL, Turn-off	0–2	0.4	[7]
N-GQDs	Electrolysis of graphite rod in 0.1M NaOH/0.1M semicarbazide aqueous solution, 4h; Centrifugation; Dialysis	5–30/20	18.95	FL, Turn-off	0–200	0.87	[8]

N-GQDs	HT (DA.HCl in water, 180°C, 24h); Filtration; Dialysis; Centrifugation	2–5.5/3.4	34	FL, Turn-off	0–80	0.063	[9][f]
N-GQDs	Electrolysis of NG@CC in NaOH/H_2O/ethanol electrolyte solution, 24h; Dialysis	1.2–4.2/3.1	10	FL, Turn-off	10–1000	0.19	[10][f]
N-GQDs	Ar/DC microplasma treatment of *chitosan* in 35mM HCl electrolyte (pH: 2.57), 1h; Purification	2–5.2/3.79	4.68	FL, Turn-off	1–300	0.0667	[11][f]
N-GQDs	ST (GO in DMF, 200°C, 8h); Centrifugation	1–3/2	31.2	FL, Turn-off	0–0.9	0.017	[12][f]
N-GQDs	Acid oxidation of *lignin* with 17.5% HNO_3 under reflux (80°C, 8h); Centrifugation; HT (above solid residue in 0.2M NaOH aqueous solution, 200ºC, 12h); Filtration; Dialysis	1.5–5.5/~3.3	28	FL, Turn-off	0–500	1.49	[13][f]
N-GQDs	Pyrolysis (ACTB, 180°C, 4h); Dispersed in water	2–4/2.99	14.4	FL, Turn-off	0–1000	0.016	[14][g]
N-GQDs	HT (CA.H_2O/NH_3 in water, 200°C, 6h); Dissolved in water & pH adjusted to 7.0; Centrifugation; Dialysis	4–6/—	19.1	FL, Turn-off	0–100	0.74	[15]
B-GQDs	HT (TNP/borax in 0.125M NaOH aqueous solution, 200°C, 6h); Dialysis	< 5/2	16.8[c]	FL, Turn-off	0.05–220, 220–420	0.0312	[16][f]
N,S-GQDs	HNO_3 vapor cutting of polythiophene-derived carbon, 180°C, 1h; *In-situ* filtration	3.9–6.3/4.7	2.35	FL, Turn-off	0–130	0.07	[17][f]
N,S-GQDs	Pyrolysis (CA/L-cysteine, 200°C, 5min); Mixed in water; Centrifugation; Dialysis	3–11/7	74.5[c]	FL, Turn-off	0.01–3	0.0033	[18][f]
N,P-GQDs	ST (CA/PNCT in alcohol, 180°C, 48h); Centrifugation; Dialysis	1–6/3.4	34.8	FL, Turn-off	0–0.15	0.146	[19][h]
N,Fe-GQDs	HT (Ammonium iron citrate/urea in water, 180°C, 6h); pH adjusted to 7.0; Centrifugation; Dialysis	2–8/4.5	30.57	FL, Turn-off<br>COL, Turn-on	10–110<br>0–450	3.21<br>1.34	[20][f]
Mn^{2+}–B,N-GQDs	HT (CA/urea/borax/$MnCl_2$.4H_2O in water, 180°C, 8h); Centrifugation; Dialysis	1.5–6.5/3.7	30.52	FL, Turn-off	10–100, 100–800	0.78, 9.08	[21]
GQDs	HNO_3 vapor cutting of ordered mesoporous carbon/silica (C/SBA-15), 160°C, 5h; *In-situ* filtration	2.5–5.2/3.6	—	FL, Turn-off	3–60	0.3	[22][f]
GQDs	Acid oxidation of carbon black with 6M HNO_3 under reflux (130°C, 24h); Centrifugation; Drying & dissolution in water	5–10/—	—	FL, Turn-off	0–60	0.45	[23]
GQDs	Chemical oxidation of GO with $KMnO_4$ under MW-ultrasonication (400W, 90°C, 0.5h); Centrifugation; Filtration; Dialysis	1–6/2	23.8	FL, Turn-off	10–120	10	[24]
GQDs cluster	MW (CA/urea/zinc precursor in water); Dialysis	—/10	18	FL, Turn-off	0–10	0.03914	[25]
GQDs	Electrochemical trimming of GO aqueous dispersion, 5h; Filtration; Dialysis	0.5–6/2	6.6	FL, Turn-off	0.1–20	0.23	[26]

GQDs	Pyrolysis (*Spent tea leaves*, 500°C, 3h); ST (Biochar/oxone in DMF, 250°C, 12h); Filtration; Dialysis	1.25–4.75/3	—	FL, Turn-off	0.5–6	0.29	[27][f]
GQDs	Pyrolysis (*Eucalyptus tree leaves* extract, 300°C, 8h); Filtration	1–5/3	6.9	FL, Turn-off	1–6[d]	—	[28]
Fluorine-rich GQDs	HT (HFG/30% H_2O_2 in water, 180°C, 1h); Centrifugation	1.3–2.4/—	2.9	FL, Turn-off	0–90	1.93	[29]
GQDs	ST (*Spent tea leaves* dispersed in ethanol, 200°C, 12h); Filtration; Dialysis	0.75–4.25/2.3	21	FL, Turn-off	1–6	0.5	[30]
**Functionalized GQDs**							
GL–N-GQDs	Thermolysis (GL in ethylene glycol, 190°C, 20min); Dialysis	1.8–4.6/3.24	16.2	FL, Turn-off	0.5–500	0.1	[31][f]
BCP–GQDs	Acid oxidation of carbon black with HNO_3:H_2O (1:1.5) under reflux (100°C, 48h); Centrifugation; Filtration; Covalently modified with BCP (P7AC-*b*-PNIPAAm)	12–44/26.6	—	FL, Turn-off	0–50	—	[32]
AL–GQDs<br><br>AS–GQDs<br>GL–GQDs	Reflux (DMF dispersion of GO in dichlorosulfoxide, 80°C, 36h); Centrifugation; Reflux with AL/AS/GL (80°C, 48h); Filtration, Dialysis	2.9–6.1/4.5<br><br>3.5–5.3/4.1<br>3.6–6.3/5.3	12.67<br><br>11.27<br>10.92	FL, Turn-off<br><br>″<br>″	0.05–7.5, 10–400<br>0.5–200<br>0.5–200	0.05<br><br>0.1<br>0.1	[33][f]
GSH–GQDs	Pyrolysis (*Lime oil* extract/glutathione, 260°C, 5min); Dissolved in 0.25M NaOH	—	—	FL, Turn-off	1–150	0.1	[34][f]
CD–sl-GQDs	HT (CA/CD in water, 160ºC, 6h); Centrifugation	3.5–6.5/4.8	5.34	FL, Turn-off	0–85	0.26	[35][f]
PEG–GQDs	HT (*Cane molasses* in water, 190°C, 24h); Filtration; Non-covalently modified with PEG-200	1.5–3.5/2.5	21.32	FL, Turn-off	8–24	5.77	[36][f]
Modified–GQDs	Pyrolysis (*Spent tea*, 500°C, 3h, N_2); Acid oxidation of biochar with HNO_3 under MW-reflux (500W, 100°C, 2h); Diluted with water; Filtration; pH adjusted to 7.0; HT (200°C, 8h); Filtration; Dialysis	0.5–3.5/1.6	23	FL, Turn-off	1–50	2.5	[37]
Luminol–GQDs	Pyrolysis (CA, 200°C, 5min); Mixed in 4 mg/mL NaOH solution; pH adjusted to 7.0; Filtration; Dialysis; Functionalized with luminol under HT condition (180°C, 8h); Filtration	6–18/~12	—	FL, Turn-off	50–300	0.08	[38]
Luminol–GQDs	Pyrolysis (CA, 200°C, 25min); Mixed in 10 mg/mL NaOH solution; pH adjusted to 7.0; Filtration; Dialysis; Functionalized with luminol under HT condition (180°C, 8h); Filtration	—/20	—	FL, Turn-off	50–400	1.5	[39][f]
**GQDs-involved with other counterparts**							
PS-AER/GQDs	Acid oxidation of graphite with HNO_3:H_2SO_4 (1:3) under ultrasonication (3h) & HT (120°C, 24h); Filtration & pH adjusted to 7.0; Non-covalent attachment on PS-AER.	< 10/—[b]	—	FL, Turn-off	1–7	0.65	[40]

Chitosan/OH-GQDs/ Au@glass slip	OH-GQDs purchased from ASC Material; Spin coated along with the chitosan on Au-modified glass slip	—	—	SPR, Change in resonance angle	0–1[e], 1–10[e]	0.5[e]	[41]
GQDs/poly(AM-*co* - AA) hydrogel	Pyrolysis (CA, 200°C, 20min); Mixed in 10 mg/mL NaOH solution & pH adjusted to 7.0; Centrifugation; *In-situ* incorporated in poly(AM-co-AA) hydrogel	2.7–4.8/3.62[b]	—	FL, Turn-off	10–160	—	[42]
**Fe^{2+}**							
GSH–GQDs/H_2O_2	Pyrolysis (*Lime oil* extract/glutathione, 260°C, 5min); Dissolved in 0.25M NaOH	—	—	FL, Turn-off	1–150	0.1	[34][f]
B,N-GQDs	MW (CA/urea/boric acid, 720W, 180°C 15min); Dispersed in water; Centrifugation	5–10/—	30.2	FL, Turn-off	0–200[e]	—	[43][f]

Abbreviations: GL: glycine, BCP: block copolymer, AL: L-alanine, AS: asparagine, GSH: glutathione, CD: β-cyclodextrin, sl: single-layered, PEG: polyethylene glycol, PS-AER: polystyrenic anion-exchange resin, poly(AM-*co*-AA): poly(acrylamide-*co*-acrylic acid), BMIMPF_6: 1-butyl-3-methylimidazolium hexafluorophosphate, HT: hydrothermal, CA: citric acid, MOF: metal organic framework, MW: microwave, GO: graphene oxide, THF: tetrahydrofuran, CVD: chemical vapour deposition, DA.HCl: dopamine hydrochloride, NG: nitrogen-doped nanomesh graphene, CC: carbon cloth, Ar: argon, DC: direct current, ST: solvothermal, DMF: dimethylforamide, ACTB: ammonium citrate tribasic, TNP: 1,3,6-trinitropyrene, PNCT: phosphonitrilic chloride trimer, HFG: hydroxylated fluorographene, P7AC-*b*-PNIPAAm: poly(7-(4-(acryloyloxy)butoxy)coumarin)-b-poly(N-isopropylacrylamide), QY: quantum yield, FL: fluorometric, COL: colorimetric, SPR: surface plasmon resonance, LR: linear range, LOD: limit of detection.

Note: [a]: measured from transmission electron microscopy, [b]: size distribution/average size of GQDs used with other counterparts, [c]: absolute QY, [d]: dynamic concentration range, [e]: LR/LOD in ppm, [f]: analytical ability in real water/biological fluid/supplement samples, [g]: paper-based sensing capability, [h]: analytical ability in living cells.

Table S2. GQDs, modified-GQDs, and GQDs-involved with other counterparts for Hg^{2+} sensing application.

GQD-based sensor	Synthesis condition	Size distribution/average size (nm)[a]	QY (%)	Sensing process	LR (µM)	LOD (µM)	Ref.
**Hg^{2+}**							
**Undoped/doped-GQDs**							
GQDs	Pyrolysis (CA, 200°C, 30min); Dissolved in 10 mg/mL NaOH solution & pH adjusted to 7.0	7–14/10.5	—	FL, Turn-off	0–60[g]	3.36[g]	[44]
GQDs	Acid oxidation of graphene with HNO_3/H_2SO_4 under ultrasonication (12h); Drying; Diluted with water; Filtration; Centrifugation	< 15/—	—	FL, Turn-off	0.8–9	0.1	[45]
GQDs	Pyrolysis (CA, 190°C, 45min); Mixed in 10 mg/mL NaOH solution & pH adjusted to 8.0	—	—	FL, Turn-off	1–10	0.09	[46]
Red-fluorescent GQDs	ST (*Psidium guajava* leave extract in ethanol, 180°C, 18h); Filtration	—	—	FL, Turn-off	60–380	82	[47]
GQDs	Pyrolysis (CA, 200°C, 30min); Mixed in 10 mg/mL NaOH solution & pH adjusted to 7.0	15–25/—	4.54	FL, Turn-off	2.5–45	0.23	[48]
N-GQDs	Pyrolysis (CA/glycine, 200°C, 20min); Dissolved in 10 mg/mL NaOH solution & pH adjusted to 7.5	0.5–4/2.2	28.1	FL, Turn-off	0–5	0.032	[49][i]
N-GQDs	HT (CA/NH_3 in water, 200°C, 3h); Dialysis	1.3–2.93/2.03	—	FL, Turn-off	0.02–1	0.0047	[50][i, j]
N-GQDs	N-GQDs purchased from JCNANO Technology	1.5–4.5/2.7	—	FL, Turn-off	2.5–800[g]	2.5[g]	[51][i]
N-GQDs	HT ($GO/NH_3.H_2O$ in water, 150°C, 7h); Filtration; Dialysis	4–6/5	8.16	FL, Turn-off	0–4.31, 4.31–12.32	0.023	[52][i]
N-GQDs	HT (CA/ethylenediamine in water, 180°C, 10h); Dialysis	2–10/5	—	FL, Turn-off	0–0.01, 0.1–1	0.00045, 0.0673	[53]
N-GQDs	HT (1,3,6-trinitropyrene/L-tryptophan in 125mM NaOH solution, 200°C, 6h); Filtration; Dialysis	1.5–3/2.1	24.8[d]	FL, Turn-off	0.05–15	0.019	[54][i]
N-GQDs	Acid oxidation of nitrogen-doped graphene with piranha under reflux (80°C, 1.5h); Diluted with water & pH adjusted to 4.0; Filtration; Dialysis	2.2–4/2.9	7.43	FL, Turn-off	0.0005–0.11	0.00008	[55][i]
N-GQDs/Filter paper	Infrared-assisted pyrolysis (CA/urea 260°C, 10min); Dispersed in water; Drop coated on cellulose filter paper	—/~4	—	FL, Turn-off	0–6[h], 6–10[h]	0.5[h]	[56]

N-GQDs	HT (HTC prepared from microcrystalline cellulose extract of *pineapple* leaf fiber/ethylenediamine in water, pH adjusted to 14, 200°C, 10h); Filtration; Dialysis	3–8/~5.1	—	FL, Turn-off	0–30	0.00025	[57][i]
N,S-GQDs	HT (CA/thiourea in water, 160°C, 4h); Centrifugation	2–6/3.5	41.9	FL, Turn-off	0.001–0.05, 0.05–15	0.00014	[58][i, j]
N,S-GQDs	Pyrolysis (CA/D-penicillamine, 160°C, 9min); Mixed in 1 mg/mL NaOH solution; Centrifugation; Filtration	1–6.5/3.15	56.4	FL, Turn-off	0.0009–0.03	0.00069	[59][i, k]
N,S-GQDs/Ce^{4+}/I^{-}	Pyrolysis (CA/L-cysteine, 200°C,); Diluted with water	2–4/—	85.6	FI-CL, Turn-on	0.3–7	0.0247	[60][i]
B,N-GQDs	HT (CA/ethylenediamine/phenylboronic acid in water, 200°C, 5h); Filtration	1.04–4.81/2.73	75	FL, Turn-off	0.2–2.6	0.16	[61][i]
B,N-GQDs	HT (GO/NH_3/boric acid in water, 180°C, 20h); Filtration; Dialysis	2.2–7.6/3.8	5.13[d]	FL, Turn-off	0.2–1	0.0064	[62][i]
N,P-GQDs	Pyrolysis (*Lemon* salt/glycine/diammonium phosphate/ phosphoric acid, 225°C, 10min); Dissolved in water & pH adjusted to 7.0; Centrifugation; Filtration	10–20/—	30[e]/ 17[f]	FL, Turn-off	1.59–16.3	0.13	[63][i]
Mn^{2+}–N-GQDs	HT (Sodium citrate/glycine/$MnCl_2$ in water, 180°C, 10h); Centrifugation; Dialysis	1–5.5/4.5	42.16	FL, Turn-off	0.2–3.5	0.034	[64]
**Functionalized GQDs**							
Thymine-rich DNA–GQDs	Acid oxidation of rGO with HNO_3:H_2SO_4 (3:1) under stirring (24h) & ultrasonication (24h); Dilution, filtration & pH adjusted to 8.0; HT (200°C, 24h); Filtration; Dialysis; Covalent modification with thymine-rich DNA	—/~20	—	FL, Turn-off	0.001–10	0.00025	[65][k]
SR–GQDs	Chemical oxidation of GO with H_2O_2/O_3 (0°C, 1h); Acid oxidation with H_2SO_4 under MW (800W, 5min); Diluted with water; Filtration; Dialysis; Covalently modified with SR	3.2–6.4/4.38	—	FL, Ratiometric	0.6–12	0.23	[66][i, k]
Cysteine–GQDs	HT (CA in water, pH adjusted to 10, 160°C, 4h); Covalently modified with L-cysteine under reflux (80°C, 24h); Dialysis	1–8/3.8	28	FL, Turn-off	0–10	0.02	[67][i]
MEA–GQDs	Pyrolysis (CA, 190°C, 25min); Pyrolysis (Obtained paste/ MEA, 120°C, 1h); Dissolved in water & pH adjusted to 7.4	1–6/—	23.7	FL, Turn-off	0.05–5	0.01	[68][i]
RhB–GQDs	HT (CA/RhB/ethylenediamine in water, 180°C, 10h); Centrifugation; Dialysis	2.5–6.5/4.5	41.6	FL, Turn-off	0–0.01, 0.05–1	0.00016, 0.04087	[69][i]
Glycine–GQDs	Pyrolysis (CA, 200ºC, 30min); Mixed in 10 mg/mL NaOH solution & pH adjusted to 8.0; Covalently modified with GL	4.5–7/5.9	35.7	FL, Turn-off	0–3	0.0083	[70][i]
TUD–GQDs-	Cutting of GO under probe ultrasonication (10kHz, 2h);	5–40/18	—	EC, DPV	0.05–23	0.0235	[71][i]

IIP@GCE	Centrifugation; Dialysis; Covalently modified with TUD; Suspension polymerization using TUD–GQDs as a monomer with Hg^{2+}; Leaching of Hg^{2+}; Drop casted on GCE			EC, CV	0.06–0.85, 1.4–7	0.0302	
8-HQ–GQDs	Electrolysis of graphene in 0.1M NaOH ethanoic solution, 1h; Centrifugation; Drying; Covalent modification with 8-HQ	—	—	FL, Turn-off	0–0.2	0.0024	[72][i]
**GQDs-involved with other counterparts**							
GQDs/CdTe@SiO_2	Pyrolysis (CA, 200°C, 30min); Mixed in 0.25M NaOH solution & pH adjusted to 7.0; Non-covalently conjugated on CdTe@SiO_2	—/10[b]	—	FL, Ratiometric	0.01–22	0.0033	[73][i, l]
GQDs/ZnPc (1)<br>GQDs/ZnPc (2)<br>GQDs/ZnPc (3)<br>GQDs/AlPc (4)	HT (GO dispersed in HNO_3/H_2SO_4 solution, pH adjusted to 8.0, 200°C, 12h); Filtration; Dialysis; Non-covalently conjugated with mercaptopyridine-substituted Zn-phthalocyanine or Al-phthalocyanine	—/14[c]<br>—/21[c]<br>—/27[c]<br>—/22[c]	18<br>16<br>12<br>16	FL, Turn-off-on<br>″<br>″<br>″	0.0005–0.05<br>0.0005–0.05<br>0.0005–0.05<br>0.0005–0.05	0.0015<br>0.00052<br>0.00012<br>0.0006	[74]
GQDs/Fe_3O_4@SiO_2	Pyrolysis (CA, 200°C, 30min); Mixed in 10 mg/mL NaOH solution & pH adjusted to 7.0; Covalently modified with amino-functionalized Fe_3O_4@SiO_2	—/20;<br>3–7/—[b]	—	FL, Turn-off	0.1–70	0.03	[75][i]
N-GQDs/PVA@ PETP	HT (CA/glycine in water, 200°C, 5h); Dialysis; Mixed with PVA & coated on PETP film	7–12/10.5	—	FL, Turn-off	0–30[g]	0.1[g]	[76][i, j]
GQDs-ssDNA-AuNPs	Pyrolysis (CA, 200°C, 30min); Mixed in 10 mg/mL NaOH solution & pH adjusted to 7.0; Covalently modified with amine-containing DNA S3; Conjugated with AuNPs	—/20[b]	—	ECL	0.00001–0.1	$2.48x10^{-6}$	[77][i]
NH_2-Ru@SiO_2/N-GQDs/aptamer/AuNPs @GCE	Pyrolysis (CA/H_2O, 200°C, 30min); Dialysis; Non-covalently conjugated with NH_2-Ru@SiO_2; Covalently conjugated with aptamer; Casted on AuNPs electrodeposited GCE	—/49.3;<br>—/2.3[b]	—	ECL	0.00005–1	$30x10^{-6}$	[78][i]

Abbreviations: SR: spirolactam rhodamine, MEA: mono-ethanolamine, RhB: rhodamine B, TUD: thiourea derivative, IIP: ion imprinted polymer, GCE: glassy carbon electrode, 8-HQ: 8-hydroxyquinoline, Pc: phthalocyanine, PVA: polyvinyl alcohol, PETP: polyethylene terephthalate, NPs: nanoparticles, CA: citric acid, ST: solvothermal, HT: hydrothermal, GO: graphene oxide, HTC: hydrothermal carbon, rGO: reduced graphene oxide, MW: microwave, GL: glycine, QY: quantum yield, FL: fluorometric, FI-CL: flow-injection chemiluminescence, EC: electrochemical, DPV: differential pulse voltammetry, CV: cyclic voltammetry, ECL: electrochemiluminescence, LR: linear range, LOD: limit of detection.

Note: [a]: measured from transmission electron microscopy, [b]: size distribution/average size of GQDs used with other counterparts, [c]: measured from dynamic light scattering, [d]: absolute QY, [e]: QY corresponding to 350 nm excitation wavelength, [f]: QY corresponding to 470 nm excitation wavelength, [g]: dynamic concentration range and corresponding LOD, [h]: LR/LOD in ppm, [i]: analytical ability in real water/biological fluid samples, [j]: paper-based sensing capability, [k]: analytical ability in living cells, [l]: visual detection capability.

Table S3. GQDs, modified-GQDs, and GQDs-involved with other counterparts for Cu^{2+} sensing application.

GQD-based sensor	Synthesis condition	Size distribution /average size (nm)[a]	QY (%)	Sensing process	LR (µM)	LOD (µM)	Ref.
**Cu^{2+}**							
**Functionalized GQDs**							
Amino–GQDs	Acid oxidation of GO dispersion in water with HNO_3:H_2SO_4 (4:1) under MW-reflux (650W, 100°C, 9h); pH adjusted to 8.0; Filtration; Dialysis; Covalent modification with amine under HT (28wt% NH_3, 200°C, 10h); Evaporation of NH_3; Dialysis	3–9/6	16.4	FL, Turn-off	0.01–0.1	0.0069	[79][d]
**Undoped/doped-GQDs**							
GQDs	Acid oxidation of GO with HNO_3:H_2SO_4 (1:3) under ultrasonication (5h); Centrifugation; pH adjusted to 12.0; HT (180°C, 10h); Filtration; Dialysis	10–30/19.6	—	FL, Turn-off	0–15	0.226	[80]
Oxidized GQDs	Electrolysis of graphite rod in 0.1M PBS; Filtration; Dialysis; Chemical oxidation with $K_2S_2O_8$ under ultra-violet irradiation (18W, 365nm, 72h); Dialysis	1–4/2.4	—	FL, Turn-off	0–20	2	[81]
GQDs	Chemical/acid oxidation of 3D NGFs with $KMnO_4$/HNO_3:H_2SO_4 (1:3.3) under reflux (80°C, 48h); pH adjusted to 7.0; Reduced with sodium borohydride (room temperature, 4h); Dialysis	0.7–2.3/1.4	4.5	FL, Turn-off	0.1–1	0.067	[82][d]
GQDs	Ultrasonication (*Anthracite coal* in DMF, 21kHz, 650W, 2h); Filtration; Dialysis	1.5–6/3.2	5.98	FL, Ratiometric	0–8	0.29	[83]
GQDs	HT (Humic acid in 0.15M NH_4OH aqueous solution, pH adjusted to 10, 200°C, 12h); Centrifugation; Dialysis	3–10/—	20	FL, Turn-off	1–40	0.44	[84][e]
GQDs	Ar/DC microplasma treatment of *starch* in 0.6M NaOH aqueous solution, 1h; Purification	2–6.3/4.1	27.5	FL, Turn-off	1–50	0.1109	[85]
Eu-GQDs	Acid oxidation of Eu-doped graphene with HNO_3:H_2SO_4 (1:3) under reflux (75°C, 7.5h); Mixed with 1M NaOH solution & pH adjusted to 7.0; Dialysis	2.3–5.4/—	2.4	FL, Turn-off	0.1–10	0.056	[86]
N-GQDs	ST (*p*-phenylenediamine/*p*-coumaric acid in ethanol, 180°C, 24h); Diluted in water & ethanol removed; Filtration; Dialysis	1.5–8.5/3.8	34.4	FL, Turn-off	0–10	0.057	[87]
N-GQDs	Electrolysis of graphite rod in NaOH/ethanol, 24h; Dialysis; pH	—/71.65[b]	18.4	FL, Turn-off	0.3–16.1	0.153	[88]

	adjusted to 7.0 & re-disperse in water; Gamma irradiation (200 kGy, Ar) with 4v% EDA/3v% IPA; Dialysis						
N-GQDs	Pyrolysis (Glutamic acid/tyrosine, 320°C, 1min); Dilution & centrifugation; Dialysis	—/5.0	12	FL, Turn-off	0.1–10	0.06	[89][d, e]
**GQDs-involved with other counterparts**							
GSH–GQDs/ CdTeQDs	Pyrolysis (Citric acid/GSH, 240°C, 10min); Diluted with water; Chromatography; Mixed with pre-synthesized CdTeQDs	—	—	FL, Ratiometric	0.1–1	0.053	[90][d, e]
N-GQDs/TryIL	HT (Citric acid/NH_3 in water, 180°C, 8h); Filtration; Dialysis; Assembly on TryIL micelles	50–350/ 139.8	37	FL, Turn-off	51–1600[c]	17[c]	[91][e]

Abbreviations: GSH: glutathione, TryIL: 1-hexyl-3-methylimidazolium tryptophan ionic liquid, GO: graphene oxide, MW: microwave, PBS: phosphate buffer saline, 3D NGFs: three-dimensional nanomesh graphene frameworks, DMF: dimethylforamide, HT: hydrothermal, Ar: argon, DC: direct current, ST: solvothermal, EDA: ethylenediamine, IPA: isopropyl alcohol, QY: quantum yield, FL: fluorometric, LR: linear range, LOD: limit of detection.

Note: [a]: measured from transmission electron microscopy, [b]: measured from dynamic light scattering, [c]: LR/LOD in µg/L, [d]: analytical ability in living cells/rat brain, [e]: analytical ability in real water/vegetable/serum samples.

Table S4. GQDs, modified-GQDs, and GQDs-involved with other counterparts for Pb^{2+} sensing application.

GQD-based sensor	Synthesis condition	Size distribution /average size (nm)[a]	QY (%)	Sensing process	LR (µM)	LOD (µM)	Ref.
**Pb^{2+}**							
sl-GQDs/L-Cysteine	Acid oxidation of carbon black with 6M HNO_3 under reflux (130°C, 24h); Centrifugation; Drying	—/10	—	ECL, Turn-off	0.1–10	0.07	[92][e]
GQDs@GCE	Acid oxidation of GO with periodic acid under heating (60°C, 24h); Centrifugation & washing; Dispersed in water with SPSS as a dispersant under ultrasonication (2h); Reduced by AA under stirring & heating (50°C, 24h); Filtration; Dialysis; Drop casted on GCE	5–15/—	—	EC, DPV	0.8–10	0.007	[93]
DNA aptamer–rGQDs /GO	Acid oxidation of graphite powder with HNO_3:H_2SO_4 (1:3) under ultrasonication (2h) & reflux (80°C, 24h); pH adjusted to 7.0; Dialysis; Reduced with sodium borohydride under heating (70°C, 8h); Dialysis; Covalently modified with a DNA aptamer; Mixed with GO	3–5/—[b]	10[c]	FL, Turn-off-on	0.0099–0.435	0.0006	[94]

S-GQDs	HT (TNP/Na_2S in 0.2M aqueous NaOH solution, 200°C, 10h); Dialysis	—/3	11.6	FL, Turn-off	0.1–1, 1–140	0.03	[95]
GQDs–DNA–AuNPs	GQDs purchased from XFNANO; Covalently modified with amine-containing DNA strand; Covalently conjugated with AuNPs containing thiol-modified DNA strand	—/5[b]	—	FL, Turn-on	0.05–4	0.0167	[96]
N,P,S-GQDs	Acid oxidation of *Anthracite coal* with HNO_3:H_2SO_4 (1:3) under ultrasonication (2h) & reflux (100°C, 24h); Mixed with 3M NaOH solution & pH adjusted to 7.0; Filtration; Dialysis	1–7/—	—	FL, Turn-off	1–20	0.75	[97]
DDTC–GQDs	Pyrolysis (CA/DDTC, 250°C, 5min); Mixed in 0.25M NaOH solution	—	—	RLS, Turn-on	1–10[d]	0.8[d]	[98][e]
DAP–GQDs	Acid oxidation of graphite flake with HNO_3:H_2SO_4 (1:3) under ultrasonication (2h) & reflux (120°C, 24h); pH adjusted to 7.0; Filtration; Dialysis; Covalently modified with DAP	1–5/2.9	13.4	FL, Turn-on	0–300	1.2	[99][e]

Abbreviations: sl: single-layered, GCE: glassy carbon electrode, rGQDs: reduced graphene quantum dots, GO: graphene oxide, NPs: nanoparticles, DDTC: diethyl dithiocarbamate, DAP: 2,6-diaminopyridine, SPSS: sodium polystyrene sulfonate, AA: L-ascorbic acid, HT: hydrothermal, TNP: 1,3,6-trinitropyrene, CA: citric acid, QY: quantum yield, ECL: electrochemiluminescence, EC: electrochemical, DPV: differential pulse voltammetry, FL: fluorometric, RLS: resonance light scattering, LR: linear range, LOD: limit of detection.
Note: [a]: measured from transmission electron microscopy, [b]: size distribution/average size of rGQDs/GQDs used with other counterparts, [c]: QY of rGQDs, [d]: LR/LOD in µg/L, [e]: analytical ability in real water samples.

Table S5. GQDs and modified-GQDs for Cr^{6+}/ Cr^{3+} sensing application.

GQD-based sensor	Synthesis condition	Size distribution /average size (nm)[a]	QY (%)	Sensing process	LR (µM)	LOD (µM)	Ref.
**Cr^{6+}/Cr^{3+}**							
GQDs/$S_2O_8^{2-}$	Acid oxidation of carbon black with HNO_3 under reflux (100°C, 24h); Centrifugation	—/10	—	ECL, Turn-off	0.05–60	0.02	[100][e]
N-GQDs<br><br>N-GQDs/Nylon membrane	HT (1,3,6-trinitropyrene/NH_3/hydrazine hydrate in water, 200°C, 8h); Filtration; Dialysis<br>Immobilized on nylon membrane	2–3.6/2.7	—	FL, Turn-off<br><br>″<br>″	1–500<br><br>1–500[b]<br>1–500[c]	0.02<br><br>0.19[b]<br>0.17[c]	[101]
PVP-passivated N-GQDs	HT (Citric acid/glutamic acid/PVP-K90 in water, 180°C, 12h); Centrifugation; Filtration; Dialysis	1.5–4/1.9	64.2	FL, Turn-off	0.1–100	0.091	[102][e]

GQDs cluster	MW (Citric acid/urea/Zn precursor in water); Dialysis	—/20	19	FL, Turn-off	0–10	0.04354	[25]
DA–N-GQDs	HT (Citric acid/urea in water, 160°C, 4h); Dialysis; Covalently modified with DA	—/3	—	FL, Ratiometric	166–2380[d]	4.56[d]	[103][e]

Abbreviations: PVP: polyvinyl pyrrolidone, DA: dopamine, HT: hydrothermal, MW: microwave, QY: quantum yield, ECL: electrochemiluminescence, FL: fluorometric, LR: linear range, LOD: limit of detection.

Note: [a]: measured from transmission electron microscopy, [b]: LR/LOD in single-piece test, [c]: LR/LOD in multiple-piece test, [d]: LR/LOD for Cr^{3+}, [e]: analytical ability in real water samples.

Table S6. GQDs, modified-GQDs, and GQDs-involved with other counterparts for Co^{2+}, Ni^{2+}, Al^{3+}, and As^{3+} sensing application.

GQD-based sensor	Synthesis condition	Size distribution /average size (nm)[a]	QY (%)	Sensing process	LR (µM)	LOD (µM)	Ref.
			**Co^{2+}**				
N-GQDs	HT (NGO/hydrazine in water, 180°C, 6h); Filtration	4–6/—	—	ECL, Ratiometric	1–70	0.2	[104][e]
			**Ni^{2+}**				
GQDs	Acid oxidation of carbon fiber with HNO_3:H_2SO_4 (3:1) under ultrasonication (2h) & reflux (150°C, 24h); pH adjusted to 8.0; Filtration; Dialysis	2.5–5/3.9	—	FL, Turn-on	0–90	4.1	[105][e]
			**Al^{3+}**				
B-GQDs	Electrolysis of graphite rod in 0.1M borax aqueous solution, 2h; Filtration, Dialysis	2–8/—	13	FL, Turn-on	0–100	3.64	[106]
N-GQDs	ST (Graphene oxide in dimethylforamide, 200°C, 5h); Filtration	20–30[b]	23.1	FL, Turn-on	2.5–75	1.3	[107][e, f]
			**As^{3+}**				
Fe_3O_4NPs–GQDs	HT (Functionalized graphene oxide/3wt% H_2O_2 in water, 180°C, 2h); Centrifugation; pH adjusted to 6.0; Covalently modified with amine-functionalized Fe_3O_4NPs	1–7/~3.1[c]	—	FL, Turn-on	5–100[d]	5.1[d]	[108][e]

Abbreviations: NPs: nanoparticles, HT: hydrothermal, NGO: nitrogen-doped graphene oxide, ST: solvothermal, QY: quantum yield, ECL: electrochemiluminescence, FL: fluorometric, LR: linear range, LOD: limit of detection.

Note: [a]: measured from transmission electron microscopy, [b]: measured from atomic force microscopy, [c]: size distribution/average size of GQDs, [d]: LR/LOD in ppb, [e]: analytical ability in real water samples, [f]: analytical ability in living cells.

Table S7. GQDs and modified-GQDs for Ag^{+} and Au^{3+} sensing application.

GQD-based sensor	Synthesis condition	Size distribution/ average size (nm)[a]	QY (%)	Sensing process	LR (µM)	LOD (µM)	Ref.
				**Ag^{+}**			
Amino–GQDs	Pyrolysis (*Neem leaves*, 1000°C, 5h, Ar); Acid oxidation with HNO_3:H_2SO_4 (1:3) under reflux (90°C , 5h); Diluted with water; Filtration; pH adjusted to 7.0; Covalently modified with amine under HT (30% NH_3, 200°C, 12h)	—/5–6	2	FL, Turn-off	—	0.033–0.1[b]	[109]
N-GQDs	MW (Glucose/NH_3 in water, 300W, 5min); Dissolved in water	—/20	—	FL, Turn-off	0.2–40	0.168	[110][c]
S-GQDs	HT (TNP/MPA in water, 200°C, 10h); Filtration; Dialysis	1.5–3.5/2.5	9.2	FL, Turn-off	0.1–130	0.03	[111][c]
GQDs	GQDs purchased from XFNANO	1–5/2.9	—	FL, Ratiometric	0–115.2	0.25	[112][c]
GQDs/Hg^{2+}/TAA	Pyrolysis (Citric acid, 200°C, 5min); Mixed in 0.25M NaOH solution & pH adjusted to 7.0	3.33–8.33/—	—	FL, Turn-on-off	0.5–10	0.18	[113][c]
				**Au^{3+}**			
GQDs<br>GQDs	Pyrolysis (Glucose, 200°C, 1.5min); Diluted with water<br>Pyrolysis (Citric acid, 200°C, 1.5min); Mixed in 10 mg/mL NaOH solution	3–33/22<br>2–12/8	—<br>—	FL, Turn-off<br>″	1–80<br>5–30	0.5<br>1.45	[114][c]

Abbreviations: TAA: thioacetamide, MW: microwave, HT: hydrothermal, TNP: 1,3,6-trinitropyrene, MPA: 3-mercaptopropionic acid, QY: quantum yield, FL: fluorometric, LR: linear range, LOD: limit of detection.
Note: [a]: measured from transmission electron microscopy, [b]: LOD in g/L, [c]: analytical ability in real water samples.

Table S8. GQDs, modified-GQDs, and GQDs-involved with other counterparts for alkali/alkaline-earth, rare-earth, and radioactive MIs sensing application.

**GQD-based sensor**	**Synthesis condition**	**Size distribution /average size (nm)**[a]	**QY (%)**	**Sensing process**	**LR (μM)**	**LOD (μM)**	**MIs**	**Ref.**
**Alkali/alkaline-earth MIs**								
PEG-modified N-GQDs-1	ST (Graphene oxide in dimethylforamide, 200°C, 4h); Filtration; Covalently modification with organic halides of 2/3/4/5/6/7 number of ethylene glycol units	1.5–8.5/4.7	71	FL, Turn-off	0.0005–0.05	0.0002	Mg^{2+}	[115]
PEG-modified N-GQDs-2		< 5/—	73	″	0.0003–0.01	0.0001	Ca^{2+}	
PEG-modified N-GQDs-3		< 6/—	69	″	0.0008–0.02	0.0004	Sr^{2+}	
PEG-modified N-GQDs-4		< 5/—	72	″	0.001–0.02	0.0005	Li^{+}	
PEG-modified N-GQDs-5		< 6/—	71	″	0.0003–0.01	0.0001	Na^{+}	
PEG-modified N-GQDs-6		< 8/—	71	″	0.0008–0.04	0.0003	K^{+}	
PEG–GQDs/Na^{+} selective ionophore	GQDs purchased from Sigma-Aldrich; Covalently modified with propargyl group & cross-linked by azide-functionalized poly(ethylene oxide); Integrated into Na^{+}/K^{+} selective ionophores	—/10[b]	—	FL, Ratiometric	100–1x10^{6} [d]	—	Na^{+}	[116][e, f]
PEG–GQDs/K^{+} selective ionophore		—/10[b]	—	″	3–1x10^{6} [d]	—	K^{+}	[116][e]
**Rare-earth MIs**								
GQDs	Acid oxidation of graphene with HNO_3:H_2SO_4 (3:1) at 25°C (12h) & under ultrasonication (12h); Pyrolysis (350°C, 20min); Diluted with water; Filtration; Dialysis	3–5/—	—	FL, Turn-off	1–30	—	Eu^{3+}	[117]
N-GQDs	Reflux (Graphene oxide dispersion in *N*-methyl-2-pyrrolidone, 202°C, 24h); Filtration; Dialysis	1.2–3.2/1.8	—	FL, Turn-off	1–44	0.8352	Ce^{4+}	[118][e]
**Radioactive MIs**								
GQDs clusters	HT (Graphene oxide/citric acid/$NH_3.H_2O$ in water, 150°C, 48h); Obtained hydrogel soaked in NaOH aqueous solution (pH: 13), 4h; Filtration	20–1300/ 290.1[c]	—	FL, Turn-off	0–1260	2.1	U^{6+}	[119]

Abbreviations: PEG: polyethylene glycol, ST: solvothermal, HT: hydrothermal, QY: quantum yield, FL: fluorometric, LR: linear range, LOD: limit of detection.

Note: [a]: measured from transmission electron microscopy, [b]: size distribution/average size of PEG–GQDs in ionophores, [c]: measured from dynamic light scattering, [d]: dynamic concentration range, [e]: analytical ability in real samples, [f]: analytical ability in living cells.

Table S9. GQDs, modified-GQDs, and GQDs-involved with other counterparts for multiple HMIs sensing application.

GQD-based sensor	Synthesis condition	Size distribution/ average size (nm)[a]	QY (%)	Sensing process	LR (μM)	LOD (μM)	HMIs	Ref.
**Multiple HMIs (EC sensor)**								
GQDs–AuNPs @GCE	Acid oxidation of carbon black with HNO_3 under reflux (100°C, 24h); Centrifugation; Covalently conjugated with cysteamine-capped AuNPs; Drop casted on GCE	—/2[b]	—	EC, ASV<br>″	0.00002–0.1<br>0.00005–0.5	0.00002<br>0.00005	Hg^{2+}<br>Cu^{2+}	[120][g]
GQDs/Chitosan @Bi-GCE	Pyrolysis (Citric acid, 200°C, 30min); Dissolved in 10 mg/mL NaOH solution & pH adjusted to 7.0; Non-covalently modified with Chitosan; Drop casted on Bi-plated GCE	1.7–3/2.5[b]	—	EC, SWASV<br>″<br>″	50–450[d]<br>50–450[d]<br>50–450[d]	8.84[d]<br>1.99[d]<br>3.10[d]	Zn^{2+}<br>Cd^{2+}<br>Pb^{2+}	[121]
GQDs/Nafion @GCE	Pyrolysis (Citric acid, 200°C, 30min); Mixed in 10 mg/mL NaOH solution & pH adjusted to 7.0; Mixed with Nafion & drop coated on GCE	—/3[b]	—	EC, SWASV<br>″	20–200[d]<br>20–200[d]	11.3[d]<br>8.49[d]	Cd^{2+}<br>Pb^{2+}	[122][g]
**Multiple HMIs (FL sensor)**								
**Undoped/doped-GQDs**								
GQDs	Pyrolysis (Citric acid, 200°C, 25min); Mixed in 10 mg/mL NaOH solution & pH adjusted to 7.0	—/20	—	FL, Turn-off	10–200<br>10–200	10<br>10	Fe^{3+}<br>Hg^{2+}	[123][g, h]
GQDs	Chemical oxidation of graphene oxide with sodium hypochlorite under UV irradiation (365nm, 6-7h); HT (180°C, 2h); Centrifugation; Dialysis	2–10/—	—	FL, Turn-off<br>″	0–50000[e]<br>0–100000[e]	1<br>4	Fe^{3+}<br>Cr^{3+}	[124]
GQDs	Acid oxidation of graphene oxide with HNO_3:H_2SO_4 (4:1) under reflux (120°C, 24h); pH adjusted to 8.0; Centrifugation; Filtration; Dialysis	2–10/—	—	FL, Turn-off<br>″<br>″	0.01–0.1, 0.1–0.5<br>0.01–0.05, 0.1–0.5<br>0.01–0.05, 0.1–1	0.047<br>0.098<br>0.098	Fe^{3+}<br>Cr^{3+}<br>Pb^{2+}	[125]
GQDs	HT (*Starch* powder in water, 180°C, 2h); Centrifugation	1–25/—	—	FL, Turn-off<br>″<br>″<br>FL, Turn-off-on<br>″<br>″	—<br>″<br>″<br>″<br>″<br>″	—<br>″<br>″<br>″<br>″<br>″	Fe^{3+}<br>Cu^{2+}<br>Cr^{3+}<br>Al^{3+}<br>Ga^{2+}<br>In^{3+}	[126]
GQDs	Acid oxidation of reduced graphene oxide with	35–75/50	—	FL, Turn-off	0–5[f]	1.171	Hg^{2+}	[127]

	HNO_3:H_2SO_4 (3:1) under ultrasonication (8h); Filtration, washing, dispersion in water & pH adjusted to 8.0; HT (200°C, 10h); Filtration			″<br>″	0–5[f]<br>0–5[f]	2.011<br>2.455	Pb^{2+}<br>Cd^{2+}	
N,S-GQDs	HT (Citric acid/thioacetamide in water, 180°C, 10h); Centrifugation; Dialysis	1.5–4/2.5	—	FL, Turn-off<br>″	0.001–0.09, 0.1–30<br>0.001–0.03, 0.1–1	0.00288, 0.05549<br>0.00027, 0.03685	Fe^{3+}<br>Hg^{2+}	[128][g]
N,S-GQDs	Pyrolysis (Citric acid/thiourea, 300°C, 20min); Diluted in water	2.3–8.3/4–5	—	FL, Turn-off<br>″	30–140<br>12–125	9.14<br>12.9	Hg^{2+}<br>Ag^{+}	[129]
N-GQDs	Pyrolysis (Citric acid/3%w/v HNO_3, 250°C, 5min); Mixed in 0.25M NaOH solution	—	83.42	FL, Turn-off<br>″	20–100<br>100–500	0.42<br>13.19	Hg^{2+}<br>Cu^{2+}	[130][g, h]
**Functionalized GQDs**								
PEI–GQDs	HT (*Coffee ground*/hydrazine hydrate in water, 180°C, 8h); Filtration; Dialysis; Covalently modified with PEI under HT (120°C, 10h); Filtration	1–5/2.67	24	FL, Turn-off<br>″	0–1<br>0–1	—<br>—	Fe^{3+}<br>Cu^{2+}	[131]
DPA–GQDs	HT (Citric acid/DPA in water, 200°C, 2.5h); Diluted with water	—/1.11[c]	—	FL, Turn-off<br>″<br>″<br>″<br>″<br>FL, Turn-on	10–150[f]<br>1–9[f]<br>1–150[f]<br>1–70[f]<br>1–70[f]<br>1–45[f]	10[f]<br>1[f]<br>1[f]<br>1[f]<br>1[f]<br>1[f]	Hg^{2+}<br>Au^{3+}<br>Pb^{2+}<br>Co^{2+}<br>Cu^{2+}<br>Ag^{+}	[132][g]
ARS–GQDs	Pyrolysis (Citric acid, 180°C, 15min); Mixed in acetone & 0.1N NaOH solution to separate oily part; Covalently modified with ARS	15–20/—	—	COL, Turn-on<br>″	1–30<br>″	0.31<br>0.35	Co^{2+}<br>Fe^{3+}	[133]
EBT–GQDs	Pyrolysis (Citric acid, 180°C, 15min); Mixed in acetone & 0.1N NaOH solution to separate oily part; Covalently modified with EBT	4–8/—[b]	—	COL, Turn-on<br>″	$2x10^4$–$8x10^4$<br>$1.5x10^4$–$20x10^4$	14000<br>18000	Co^{2+}<br>Cu^{2+}	[134]

Abbreviations: NPs: nanoparticles, GCE: glassy carbon electrode, PEI: polyethylene imine, DPA: D-penicillamine, ARS: alizarine red S, EBT: eriochrome black T, UV: ultra-violet, HT: hydrothermal, QY: quantum yield, EC: electrochemical, ASV: anodic stripping voltammetry, SWASV: square wave anodic stripping voltammetry, FL: fluorometric, COL: colorimetric, LR: linear range, LOD: limit of detection.

Note: [a]: measured from transmission electron microscopy, [b]: size distribution/average size of GQDs used with other counterparts or before functionalization, [c]: measured from dynamic light scattering, [d]: LR/LOD in μg/L during simultaneous detection, [e]: dynamic concentration range, [f]: LR/LOD in ppm, [g]: analytical ability in real water/other real samples, [h]: simultaneous detection capability of multiple HMIs.

Table S10. GQDs, modified-GQDs, and GQDs-involved with other counterparts for anion sensing application.

GQD-based sensor	Synthesis condition	Size distribution / average size (nm)[a]	QY (%)	Sensing process	LR (μM)	LOD (μM)	Ref.
**Anions**							
**Phosphate (PO_4^{3-})**							
GQDs/Eu^{3+}	Acid oxidation of graphene sheets with HNO_3:H_2SO_4 (3:1) under ultrasonication (17h); HT (Oxidized graphene sheets in water & pH adjusted to 8.0, 200°C, 12h); Filtration; Dialysis	10–20/—	—	FL, Turn-off-on	0.5–190	0.1	[135][i]
GQDs/Eu^{3+}	Acid oxidation of graphene with HNO_3:H_2SO_4 (3:1) at 25°C (12h) & under ultrasonication (12h); Pyrolysis (350°C, 20min); Diluted with water; Filtration; Dialysis	3–5/—	—	FL, Turn-off-on	1–12	0.1	[117][i]
sl-GQDs/Al^{3+}	Hydroquinone in water treated with 20%H_2O_2 followed by triethylenetetramine (25°C, 5min); Dialysis	2.5–6/4.5	—	FL, Turn-on-off	0.25–7.5	0.1	[136][i]
sl-GQDs/Dy^{3+}	HT (PTCDA/triethylamine in water, 220°C, 48h); Filtration; Dialysis; Exfoliated in methanol; Drying	1.3–3.4/2.1	—	FL, Turn-off-on	0.2–30	0.1	[137][i]
B-GQDs/Fe^{3+}	HT (1,3,6-trinitropyrene/borax in 0.125M NaOH aqueous solution, 200°C, 6h); Dialysis	< 5/2	16.8[d]	FL, Turn-off-on	3–40	0.34	[16][i]
N-GQDs/ $Mo_7O_{24}^{6-}$	ST (Julolidine/acetic acid in ethanol, 200°C, 12h); Filtration; Dialysis	—/4.8	53	FL, Turn-off-on	7–30	0.05	[138][i, j]
**Pyrophosphate ($P_2O_7^{4-}$)**							
N-GQDs/Eu^{3+}	Chemical oxidation of graphene oxide/lysine with 30% H_2O_2 under reflux (130°C, 4h); Removal of excess H_2O_2; Filtration; Dialysis	1.5–3.5/—	13.2	FL, Turn-off-on	0.3–5	0.074	[139][i]
**Hypochlorite (ClO^-)**							
GQDs	Pyrolysis (Glucose, 180°C, 1.5min); Diluted with water	—/20	—	CL, Turn-on	0.5–1000	0.3	[140][i]
o-PD–GQDs	HT (GO/KO_2 in water, 200°C, 24h); pH adjusted to 7.0; Filtration; Dialysis; Covalently modified with *o*-PD	2–8/~5.2	6.2	FL, Turn-off	0.1–1	0.069	[141][i, j]
GQDs	Pyrolysis (Citric acid, 200°C, 30min); Mixed in 10 mg/mL NaOH solution & pH adjusted to 7.0	10–25/15	—	COL, Turn-on	5–240	2.1	[142][i]
GQDs	Pyrolysis (Citric acid, 200°C, 30min); Mixed in 10	15–25/—	4.54	FL, Turn-off	0.25–5	0.0221	[48]

	mg/mL NaOH solution & pH adjusted to 7.0						
BSA–GQDs	HT (Citric acid/BSA in water, 180°C, 3h)	2–11/5.4	—	FL, Turn-off	500–1000	35.39	[143]
**Sulfide (S^{2-})**							
GQDs/Cu^{2+}	Acid oxidation of graphene oxide with HNO_3:H_2SO_4 (1:3) under ultrasonication (5h); Centrifugation; Dispersed in water and pH adjusted to 12.0; HT (180°C, 10h); Filtration; Dialysis	10–30/20.22	—	FL, Turn-off-on	0.2–20	0.1	[144][i]
SA,GSH–GQDs/ Cu^{2+}	Chemical oxidation of GO with H_2SO_4/$KMnO_4$/H_2O_2; Dialysis; Covalently modified with SA and GSH	—/10.3	3.37	FL, Turn-off-on	0.05–3.75	0.0126	[145][i]
**Cyanide (CN^-)**							
AuNPs–Peptides@ GQDs	ST (Graphene oxide in dimethylforamide, 200°C, 4.5h); Filtration; Dialysis; Non-covalent modification with AuNPs–Peptides; Centrifugation	—/3.8[b]	28	FL, Turn-on	1–200	0.52	[146][j]
N,S-GQDs/AgNPs	Pyrolysis (Citric acid/L-cysteine, 240°C, 10min); Dissolved in water	1.2–3.2/2.2	67	FL, Turn-off-on<br>COL, Turn-off	10–500<br>10–400	0.52<br>0.78	[147][i]
GQDs/Hg^{2+}	Pyrolysis (Citric acid, 220°C, 5min); Mixed in 0.25M NaOH solution	—/7.12	28.7	FL, Turn-off-on	5–15	3.1	[148][i]
**Nitrite (NO_2^-)**							
N-GQDs/NCNFs @GCE	HT (Citric acid/dicyandiamide/NCNFs in water, 180°C, 12h); Filtration; Washing; Drop-casted on GCE	—	—	EC, DPV	5–300, 400–3000	3	[149][i]
S,P-GQDs	HT (Citric acid/anhydrous sodium sulfate/sodium phytate in water, 180°C, 7h); Centrifugation; Dialysis	2.5–4.25/3.5	15.69	FL, Turn-off	0.7–9	0.3	[150][i]
**Iodide (I^-)**							
N-GQDs/Ag^+	HT (Citric acid/urea in water, 160°C, 4h); Centrifugation	2–8/4.6	15.3	FL, Turn-off-on	0.0125–0.35, 0.35–4	0.0056	[151][i]
**Fluoride (F^-)**							
B,N-GQDs/Hg^{2+}	HT (Citric acid/ethylenediamine/phenylboronic acid in water, 200°C, 5h); Filtration	1.04–4.81/ 2.73	75	FL, Turn-off-on	0.25–7	0.18	[61][i]
**Thiocyanate (SCN^-)**							
GQDs/Hg^{2+}	Pyrolysis (Citric acid, 200°C, 14min); pH adjusted to 7.0; Dialysis	—/1.8[c]	—	FL, Turn-off-on	—	—	[152]
**Other anions**							
GQDs/RF@GCE	Pyrolysis (Citric acid, 200°C, 30min); Mixed in 10 mg/mL NaOH solution & pH adjusted to 7.0; Drop-	—	—	EC, Amperometry	1–1000[e]	0.2[e]	[153]

	casted on GCE; Electrochemical deposition of RF						
N-GQDs/Fe^{3+}	Pyrolysis (Citric acid/NH_3, 210°C, 1h); Diluted with water	—	15.5	FL, Turn-off-on	$4–200^f$	1.43^f	[154][i]
IL@GQDs	HT (1,3,6-trinitropyrene in 5 mg/mL aqueous NaOH solution, 200°C, 4h); Filtration; Dialysis; Non-covalently modified with IL ($BMIMBF_4$); Dialysis	1.6–2.4/2	11.8	FL, Turn-off	$0.1–50^g$, $50–2500^g$	0.04^g	[155][i]
GQDs/Hg^{2+}/CN^-	Pyrolysis (Citric acid, 220°C, 5min); Mixed in 0.25M NaOH solution	—/7.12	28.7	FL, Turn-off	$10–50^g$	9.48^g	[148][i]
MEA-GQDs/ Hg^{2+}	Pyrolysis (Citric acid, 190°C, 25min); Pyrolysis (Obtained paste/MEA, 120°C, 1h); Dissolved in water & pH adjusted to 7.4	1–6/—	23.7	FL, Turn-off-on	$0.05–3^h$	0.03^h	[68][i]

Abbreviations: sl: single-layered, *o*-PD: 1,2-diaminobenzene/*o*-phenylenediamine, BSA: bovine serum albumin, SA: sulfanilic acid, GSH: glutathione, NPs: nanoparticles, NCNFs: nitrogen-doped carbon nanofibers, GCE: glassy carbon electrode, RF: riboflavin, IL: ionic liquid, MEA: mono-ethanolamine, HT: hydrothermal, PTCDA: perylene-3,4,9,10-tetracarboxylic dianhydride, ST: solvothermal, GO: graphene oxide, $BMIMBF_4$: 1-butyl-3-methylimidazolium tetrafluoroborate, QY: quantum yield, FL: fluorometric, CL: chemiluminescence, COL: colorimetric, EC: electrochemical, DPV: differential pulse voltammetry, LR: linear range, LOD: limit of detection.

Note: [a]: measured from transmission electron microscopy, [b]: size distribution/average size of GQDs used with other counterparts, [c]: measured from AFM, [d]: absolute QY, [e]: LR/LOD in the detection of persulfate ($S_2O_8^{2-}$), [f]: LR/LOD in the detection of sulfite (SO_3^{2-}), [g]: LR/LOD in the detection of ferricyanide ($Fe(CN)_6^{3-}$), [h]: LR/LOD in the detection of ethyl xanthate (EtX^-), [i]: analytical ability in real samples, [j]: analytical ability in living cells.

References

1. A. Ananthanarayanan, X. Wang, P. Routh, B. Sana, S. Lim, D. H. Kim, K. H. Lim, J. Li and P. Chen, Facile synthesis of graphene quantum dots from 3D graphene and their application for Fe^{3+} sensing. *Adv. Funct. Mater.*, 2014, **24**, 3021–3026.
2. T. V. Tam, N. B. Trung, H. R. Kim, J. S. Chung and W. M. Choi, One-pot synthesis of N-doped graphene quantum dots as a fluorescent sensing platform for Fe^{3+} ions detection. *Sens. Actuators B*, 2014, **202**, 568–573.
3. H. Xu, S. Zhou, L. Xiao, H. Wang, S. Li and Q. Yuan, Fabrication of a nitrogen-doped graphene quantum dot from MOF-derived porous carbon and its application for highly selective fluorescence detection of Fe^{3+}. *J. Mater. Chem. C*, 2015, **3**, 291–297.
4. C. Zhang, Y. Cui, L. Song, X. Liu and Z. Hu, Microwave assisted one-pot synthesis of graphene quantum dots as highly sensitive fluorescent probes for detection of iron ions and pH value. *Talanta*, 2016, **150**, 54–60.
5. W. Zhang, S. Tang, S. Zhang and Y. Chen, Purification of nitrogen-doped graphene quantum dots via the liquid–liquid extraction system of tetrahydrofuran–$(NH_4)_2SO_4$–water and its application to sensitive iron(III) ions determination. *Anal. Methods*, 2017, **9**, 5691–5696.
6. H. Xu, S. Zhou, J. Liu and Y. Wei, Nanospace-confined preparation of uniform nitrogen-doped graphene quantum dots for highly selective fluorescence dual-function determination of Fe^{3+} and ascorbic acid. *RSC Adv.*, 2018, **8**, 5500–5508.
7. H. Zheng, P. Zheng, L. Zheng, Y. Jiang, Z. Wu, F. Wu, L. Shao, Y. Liu and Y. Zhang, Nitrogen-doped graphene quantum dots synthesized by C_{60}/nitrogen plasma with excitation-independent blue photoluminescence emission for sensing of ferric ions. *J. Phys. Chem. C*, 2018, **122**, 29613–29619.
8. Y. Fu, G. Gao and J. Zhi, Electrochemical synthesis of multicolour fluorescent N-doped graphene quantum dots as a ferric ion sensor and their application in bioimaging. *J. Mater. Chem. B*, 2019, **7**, 1494–1502.
9. Z. Wang, D. Chen, B. Gu, B. Gao, Z. Liu, Y. Yang, Q. Guo, X. Zheng and G. Wang, Yellow emissive nitrogen-doped graphene quantum dots as a label-free fluorescent probe for Fe^{3+} sensing and bioimaging. *Diam. Relat. Mater.*, 2020, **104**, 107749.
10. F. Yang, W. Bao, T. Liu, B. Zhang, S. Huang, W. Yang, Y. Li, N. Li, C. Wang, C. Pan and Y. Li, Nitrogen-doped graphene quantum dots prepared by electrolysis of nitrogen-doped nanomesh graphene for the fluorometric determination of ferric ions. *Microchim. Acta*, 2020, **187**, 322.
11. D. Kurniawan, R. J. Weng, O. Setiawan, K. (Ken) Ostrikov and W. H. Chiang, Microplasma nanoengineering of emission-tuneable colloidal nitrogen-doped graphene quantum dots as smart environmental-responsive nanosensors and nanothermometers. *Carbon*, 2021, **185**, 501–513.
12. N. Sohal, S. K. Bhatia, S. Basu and B. Maity, Nanomolar level detection of metal ions by improving the monodispersity and stability of nitrogen-doped graphene quantum dots. *New J. Chem.*, 2021, **45**, 19941–19949.
13. L. Zhu, D. Li, H. Lu, S. Zhang and H. Gao, Lignin-based fluorescence-switchable graphene quantum dots for Fe^{3+} and ascorbic acid detection. *Int. J. Biol. Macromol.*, 2022, **194**, 254–263.
14. Q. Yao, H. Wu, Y. Jin, C. Wang, R. Zhang, Y. Lin, S. Wu and Y. Hu, One-pot synthesis of fluorescent nitrogen-doped graphene quantum dots for portable detection of iron ion. *Curr. Appl. Phys.*, 2022, **41**, 191–199.

15. H. Zhou, M. Ou, D. Sun and C. Yang, Facile preparation of highly fluorescent nitrogen-doped graphene quantum dots for sensitive Fe^{3+} detection. *Optics Laser Technol.*, 2022, **156**, 108542.
16. S. Ge, J. He, C. Ma, J. Liu, F. Xi, and X. Dong, One-step synthesis of boron-doped graphene quantum dots for fluorescent sensors and biosensor. *Talanta*, 2019, **199**, 581–589.
17. H. Xu, S. Zhou, L. Xiao, Q. Yuan and W. Gan, Time-efficient syntheses of nitrogen and sulfur co-doped graphene quantum dots with tunable luminescence and their sensing applications. *RSC Adv.*, 2016, **6**, 36554–36560.
18. C. Xia, X. Hai, X. W. Chen and J. H. Wang, Simultaneously fabrication of free and solidified N, S-doped graphene quantum dots via a facile solvent-free synthesis route for fluorescent detection. *Talanta*, 2017, **168**, 269–278.
19. Y. Yang, B. Gu, Z. Liu, D. Chen, Y. Zhao, Q. Guo and G. Wang, Hydrothermal synthesis of N, P co-doped graphene quantum dots for high-performance Fe^{3+} detection and bioimaging. *J. Nanopart. Res.*, 2021, **23**, 40.
20. X. X. Gao, X. Zhou, Y. F. Ma, C. P. Wang and F. X. Chu, A fluorometric and colorimetric dual-mode sensor based on nitrogen and iron co-doped graphene quantum dots for detection of ferric ions in biological fluids and cellular imaging. *New J. Chem.*, 2018, **42**, 14751–14756.
21. B. Li, X. Xiao, M. Hu, Y. Wang, Y. Wang, X. Yan, Z. Huang, P. Servati, L. Huang and J. Tang, Mn, B, N co-doped graphene quantum dots for fluorescence sensing and biological imaging. *Arab. J. Chem.*, 2022, **15**, 103856.
22. H. Xu, S. Zhou, L. Xiao, S. Li, T. Song, Y. Wang and Q. Yuan, Nanoreactor-confined synthesis and separation of yellow-luminescent graphene quantum dots with a recyclable SBA-15 template and their application for Fe(III) sensing. *Carbon*, 2015, **87**, 215–225.
23. X. Zhu, Z. Zhang, Z. Xue, C. Huang, Y. Shan, C. Liu, X. Qin, W. Yang, X. Chen and T. Wang, Understanding the selective detection of Fe^{3+} based on graphene quantum dots as fluorescent probes: the K_{sp} of a metal hydroxide-assisted mechanism. *Anal. Chem.*, 2017, **89**, 12054–12058.
24. R. V. Nair, R. T. Thomas, V. Sankar, H. Muhammad, M. Dong and S. Pillai, Rapid, acid-free synthesis of high-quality graphene quantum dots for aggregation induced sensing of metal ions and bioimaging. *ACS Omega*, 2017, **2**, 8051–8061.
25. G. Bharathi, D. Nataraj, S. Premkumar, P. Saravanan, D. T. Thangadurai, O. Y. Khyzhun, K. Senthilkumar, R. Kathiresan, P. Kolandaivel, M. Gupta and D. Phase, Insight into the photophysics of strong dual emission (blue & green) producing graphene quantum dot clusters and their application towards selective and sensitive detection of trace level Fe^{3+} and Cr^{6+} ions. *RSC Adv.*, 2020, **10**, 26613–26630.
26. R. Qiang, W. Sun, K. Hou, Z. Li, J. Zhang, Y. Ding, J. Wang and S. Yang, Electrochemical trimming of graphene oxide affords graphene quantum dots for Fe^{3+} detection. *ACS Appl. Nano Mater.*, 2021, **4**, 5220–5229.
27. A. Abbas, Q. Liang, S. Abbas, M. Liaqat, S. Rubab and T. A. Tabish, Eco-friendly sustainable synthesis of graphene quantum dots from biowaste as a highly selective sensor. *Nanomaterials*, 2022, **12**, 3696.
28. A. Saud, H. Saleem, N. Munira, A. A. Shahab, H. R. Siddiqui and S. J. Zaidi, Sustainable preparation of graphene quantum dots for metal ion sensing application. *Nanomaterials*, 2023, **13**, 148.
29. K. Sreeja, M. Usha, U. Rajeena, P. Raveendran and R. M. Ramakrishnan, Fluorine-rich graphene quantum dots by selective oxidative cutting of hydroxy fluorographene and their application for sensing of Fe (III) ions. *J. Fluor. Chem.*, 2023, **268**, 110130.

30. A. Abbas, S. Rubab, A. Rehman, S. Irfan, H. M. A. Sharif, Q. Liang and T. A. Tabish, One-step green synthesis of biomass-derived graphene quantum dots as a highly selective optical sensing probe. *Mater. Today Chem.*, 2023, **30**, 101555.
31. L. Li, L. Li, C. Wang, K. Liu, R. Zhu, H. Qiang and Y. Lin, Synthesis of nitrogen-doped and amino acid-functionalized graphene quantum dots from glycine, and their application to the fluorometric determination of ferric ion. *Microchim. Acta*, 2015, **182**, 763–770.
32. C. H. Park, H. Yang, J. Lee, H. H. Cho, D. Kim, D. C. Lee and B. J. Kim, Multicolor emitting block copolymer-integrated graphene quantum dots for colorimetric, simultaneous sensing of temperature, pH, and metal ions. *Chem. Mater.*, 2015, **27**, 5288–5294.
33. Q. Ma, J. Song, S. Wang, J. Yang, Y. Guo and C. Dong, A general sensing strategy for detection of Fe^{3+} by using amino acid-modified graphene quantum dots as fluorescent probe. *Appl. Surf. Sci.*, 2016, **389**, 995–1002.
34. K. Saenwong, P. Nuengmatcha, P. Sricharoen, N. Limchoowong and S. Chanthai, GSH-doped GQDs using citric acid rich-lime oil extract for highly selective and sensitive determination and discrimination of Fe^{3+} and Fe^{2+} in the presence of H_2O_2 by a fluorescence "turn-off" sensor. *RSC Adv.*, 2018, **8**, 10148–10157.
35. H. Wang, X. Wu, W. Dong, S. L. Lee, Q. Yuan and W. Gan, One-step preparation of single-layered graphene quantum dots for the detection of Fe^{3+}. *Spectrochim. Acta A*, 2020, **226**, 117626.
36. Y. Lou, J. Ji, A. Qin, L. Liao, Z. Li, S. Chen, K. Zhang and J. Ou, Cane molasses graphene quantum dots passivated by PEG functionalization for detection of metal ions. *ACS Omega*, 2020, **5**, 6763–6772.
37. A. Abbas, T. A. Tabish, S. J. Bull, T. M. Lim and A. N. Phan, High yield synthesis of graphene quantum dots from biomass waste as a highly selective probe for Fe^{3+} sensing. *Sci. Rep.*, 2020, **10**, 21262.
38. N. H. Harran and B. F. Alfarhani, Luminol-modified graphene quantum dots as an efficient nano-sensor for Fe^{3+} sensing in aqueous solution. *Chem. Africa*, 2024, **7**, 3377–3385.
39. N. H. Harran and B. F. Alfarhani, Sensitive and selective fluorescent on/off switch for detection of Fe (III) ion in human urine using luminol‑functionalized graphene quantum dots. *Chem. Papers*, 2024, **78**, 8739–8748.
40. W. Zhang and J. Gan, Synthesis of blue-photoluminescent graphene quantum dots/polystyrenic anion-exchange resin for Fe (III) detection. *Appl. Surf. Sci.*, 2016, **372**, 145–151.
41. N. A. A. Anas, Y. W. Fen, N. A. S. Omar, N. S. M. Ramdzan, W. M. E. M. M. Daniyal, S. Saleviter and A. A. Zainudin, Optical properties of chitosan/hydroxyl-functionalized graphene quantum dots thin film for potential optical detection of ferric (III) ion. *Optics Laser Technol.*, 2019, **120**, 105724.
42. J. Du, W. Zhu, X. She, Q. Yu, Q. Yang, H. Huang, C. Tsou and D. G. Manuel, A tough fluorescent nanocomposite hydrogel probe based on graphene quantum dots for the selective detection of Fe^{3+} ions. *Mater. Adv.*, 2022, **3**, 7579–7589.
43. C. T. Hsieh, P. Y. Sung, Y. A. Gandomi, K. S. Khoo and J. K. Chang, Microwave synthesis of boron- and nitrogen-codoped graphene quantum dots and their detection to pesticides and metal ions. *Chemosphere*, 2023, **318**, 137926.
44. H. Chakraborti, S. Sinha, S. Ghosh and S. K. Pal, Interfacing water soluble nanomaterials with fluorescence chemosensing: graphene quantum dot to detect Hg^{2+} in 100% aqueous solution. *Mater. Lett.*, 2013, **97**, 78–80.

45. B. Wang, S. Zhuo, L. Chen and Y. Zhang, Fluorescent graphene quantum dot nanoprobes for the sensitive and selective detection of mercury ions. *Spectrochim. Acta A*, 2014, **131**, 384–387.
46. M. Roushani, S. Kohzadi, S. Haghjoo and A. Azadbakht, Dual detection of malation and Hg (II) by fluorescence switching of graphene quantum dots. *Environ. Technol. Monit. Manage.*, 2018, **10**, 308–313.
47. R. V. Khose, G. Chakraborty, M. P. Bondarde, P. H. Wadekar, A. K. Ray and S. Some, Red-fluorescent graphene quantum dots from guava leaf as a turn-off probe for sensing aqueous Hg(II). *New J. Chem.*, 2021, **45**, 4617–4625.
48. H. Zhang, Z. Yuan, M. Wang, L. Zhu, X. Cheng, D. Cao, R. Guan and C. Zhou, Application of graphene quantum dots in the detection of Hg^{2+} and ClO^{-} and analysis of detection mechanism. *Diam. Relat. Mater.*, 2021, **117**, 108454.
49. Z. Yan, X. Qu, Q. Niu, C. Tian, C. Fan and B. Ye, A green synthesis of highly fluorescent nitrogen-doped graphene quantum dots for the highly sensitive and selective detection of mercury(II) ions and biothiols. *Anal. Methods*, 2016, **8**, 1565–1571.
50. D. Su, M. Wang, Q. Liu, Z. Qu and X. Su, A novel fluorescence strategy for mercury ion and trypsin activity assay based on nitrogen-doped graphene quantum dots. *New J. Chem.*, 2018, **42**, 17083–17090.
51. Y. Liu, X. Tang, M. Deng, Y. Cao, Y. Li, H. Zheng, F. Li, F. Yan, T. Lan, L. Shi, L. Gao, L. Huang, T. Zhu, H. Lin, Y. Bai, D. Qu, X. Huang and F. Qiu, Nitrogen doped graphene quantum dots as a fluorescent probe for mercury(II) ions. *Microchim. Acta*, 2019, **186**, 140.
52. F. Du, L. Sun, Q. Zen, W. Tan, Z. Cheng, G. Ruan and J. Li, A highly sensitive and selective “on-off-on” fluorescent sensor based on nitrogen doped graphene quantum dots for the detection of Hg^{2+} and paraquat. *Sens. Actuators B*, 2019, **288**, 96–103.
53. Y. Yang, X. Xiao, X. Xing, Z. Wang, T. Zou and Z. Wang, One-pot synthesis of N-doped graphene quantum dots as highly sensitive fluorescent sensor for detection of mercury ions water solutions. *Mater. Res. Express*, 2019, **6**, 095615.
54. Y. Mao, C. Zhao, S. Ge, T. Luo, J. Chen, J. Liu, F. Xi and J. Liu, Gram-scale synthesis of nitrogen doped graphene quantum dots for sensitive detection of mercury ions and L-cysteine. *RSC Adv.*, 2019, **9**, 32977–32983.
55. S. M. Du, B. B. Shang, X. R. Zhang, F. Feng, S. H. Zhang and B. P. Qi, A Facile Hg^{2+}-related quenching photoluminescence sensor based on nitrogen-doped graphene quantum dots. *Bull. Korean Chem. Soc.*, 2020, **41**, 948–953.
56. S. Yeom, K. D. Kihm, C. T. Hsieh and T. S. Oh, Facile optical quantification of mercury ion concentration using graphene quantum dot coated filter paper disks. *Mater. Chem. Phys.*, 2021, **260**, 124168.
57. Z. H. Xiong, Y. N. Zou, X. C. Cao and Z. H. Lin, Color-tunable fluorescent nitrogen-doped graphene quantum dots derived from pineapple leaf fiber biomass to detect Hg^{2+}. *Chin. J. Anal. Chem.*, 2022, **50.2**, 69–76.
58. N. T. N. Anh, A. D. Chowdhury and R. A. Doong, Highly sensitive and selective detection of mercury ions using N, S-codoped graphene quantum dots and its paper strip based sensing application in wastewater. *Sens. Actuators B*, 2017, **252**, 1169–1178.
59. C. Qu, D. Zhang, R. Yang, J. Hu and L. Qu, Nitrogen and sulfur co-doped graphene quantum dots for the highly sensitive and selective detection of mercury ion in living cells. *Spectrochim. Acta A*, 2019, **206**, 588–596.

60. J. Zhang, Y. Li and S. Han, Simultaneous detection of iodide and mercuric ions by nitrogen-sulfur co-doped graphene quantum dots based on flow injection "turn off-on" chemiluminescence analysis system. *Microchem. J.*, 2019, **147**, 1141–1146.
61. P. Yang, J. Su, R. Guo, F. Yao and C. Yuan, B,N-co-doped graphene quantum dots as fluorescence sensor for detection of Hg^{2+} and F^{-} ions. *Anal. Methods*, 2019, **11**, 1879–1883.
62. Z. Liu, Z. Mo, X. Niu, X. Yang, Y. Jiang, P. Zhao, N. Liu and R. Guo, Highly sensitive fluorescence sensor for mercury(II) based on boron- and nitrogen-co-doped graphene quantum dots. *J. Colloid Interface Sci.*, 2020, **566**, 357–368.
63. A. M. Senol, S. B. Kassa and Y. Onganer, A simple fluorescent "Turn off-on" sensor based on P, N-doped graphene quantum dots for Hg^{2+} and cysteine determination. *Sens. Actuators A*, 2023, **356**, 114362.
64. L. Yang, A. Qin, S. Chen, L. Liao, J. Qin and K. Zhang, Manganese(II) enhanced fluorescent nitrogen-doped graphene quantum dots: a facile and efficient synthesis and their applications for bioimaging and detection of Hg^{2+} ions. *RSC Adv.*, 2018, **8**, 5902–5911.
65. X. Zhao, J. Gao, X. He, L. Cong, H. Zhao, X. Li and F. Tan, DNA-modified graphene quantum dots as a sensing platform for detection of Hg^{2+} in living cells. *RSC Adv.*, 2015, **5**, 39587–39591.
66. M. Liu, T. Liu, Y. Li, H. Xu, B. Zheng, D. Wang, J. Du and D. Xiao, A FRET chemsensor based on graphene quantum dots for detecting and intracellular imaging of Hg^{2+}. *Talanta*, 2015, **143**, 442–449.
67. T. V. Tam, S. H. Hong and W. M. Choi, Facile synthesis of cysteine–functionalized graphene quantum dots for a fluorescence probe for mercury ions. *RSC Adv.*, 2015, **5**, 97598–97603.
68. M. H. Amini, F. Faridbod, M. R. Ganjali and P. Norouzi, Functionalized graphene quantum dots as a fluorescent "off-on" nanosensor for detection of mercury and ethylxanthate. *Res. Chem. Intermed.*, 2017, **43**, 7457–7470.
69. Y. Yang, X. Xiao, X. Xing, Z. Wang, T. Zou, Z. Wang, R. Zhao and Y. Wang, Rhodamine B assisted graphene quantum dots flourescent sensor system for sensitive recognition of mercury ions. *J. Lumin.*, 2019, **207**, 273–281.
70. Q. Zhu, H. Mao, J. Li, J. Hua, J. Wang, R. Yang and Z. Li, A glycine-functionalized graphene quantum dots synthesized by a facile post-modification strategy for a sensitive and selective fluorescence sensor of mercury ions. *Spectrochim. Acta A*, 2021, **247**, 119090.
71. S. Soman, Aswathy P.V. and Kala R, Covalently modified graphene quantum dot using a thiourea based imprinted polymer for the selective electrochemical sensing of Hg(II) ions. *J. Polym. Res.*, 2021, **28**, 359.
72. M. Llaver, S. D. Barrionuevo, E. Prieto, R. G. Wuilloud and F. J. Ibanez, Functionalized graphene quantum dots obtained from graphene foams used for highly selective detection of Hg^{2+} in real samples. *Anal. Chim. Acta*, 2022, **1232**, 340422.
73. M. Hua, C. Wang, J. Qian, K. Wang, Z. Yang, Q. Liu, H. Mao and K. Wang, Preparation of graphene quantum dots based core-satellite hybrid spheres and their use as the ratiometric fluorescence probe for visual determination of mercury(II) ions. *Anal. Chim. Acta*, 2015, **888**, 173–181.
74. O. J. Achadu and T. Nyokong, Graphene quantum dots coordinated to mercaptopyridine-substituted phthalocyanines: characterization and application as fluorescence "turn ON" nanoprobes. *Spectrochim. Acta A*, 2017, **174**, 339–347.

75. M. Alvand and F. Shemirani, A Fe_3O_4@SiO_2@graphene quantum dot core-shell structured nanomaterial as a fluorescent probe and for magnetic removal of mercury(II) ion. *Microchim. Acta*, 2017, **184**, 1621–1629.
76. C. Wang, Y. Sun, J. Jin, Z. Xiong, D. Li, J. Yao and Y. Liu, Highly selective, rapid-functioning and sensitive fluorescent test paper based on graphene quantum dots for on-line detection of metal ions. *Anal. Methods*, 2018, **10**, 1163–1171.
77. Y. Tang, J. Li, Q. Guo and G. Nie, An ultrasensitive electrochemiluminescence assay for Hg^{2+} through graphene quantum dots and poly(5-formylindole) nanocomposite. *Sens. Actuators B*, 2019, **282**, 824–830.
78. L. Li, B. Chen, L. Luo, X. Liu, X. Bi and T. You, Sensitive and selective detection of Hg^{2+} in tap and canal water via self-enhanced ECL aptasensor based on NH_2–Ru@SiO_2-NGQDs. *Talanta*, 2021, **222**, 121579.
79. H. Sun, N. Gao, L. Wu, J. Ren, W. Wei and X. Qu, Highly photoluminescent amino-functionalized graphene quantum dots used for sensing copper ions. *Chem. Eur. J.*, 2013, **19**, 13362–13368.
80. F. Wang, Z. Gu, W. Lei, W. Wang, X. Xia and Q. Hao, Graphene quantum dots as a fluorescent sensing platform for highly efficient detection of copper(II) ions. *Sens. Actuators B*, 2014, **190**, 516–522.
81. Y. Li, X. Liu, Q. Li, J. Ge, H. Liu, S. Li, L. Wang, J. Wang and N. Ma, Post-oxidation treated graphene quantum dots as a fluorescent probe for sensitive detection of copper ions. *Chem. Phys. Lett.*, 2016, **664**, 127–132.
82. C. Wang, F. Yang, Y. Tang, W. Yang, H. Zhong, C. Yu, R. Li, H. Zhou, Y. Li and L. Mao, Graphene quantum dots nanosensor derived from 3D nanomesh graphene frameworks and its application for fluorescent sensing of Cu^{2+} in rat brain. *Sens. Actuators B*, 2018, **258**, 672–681.
83. Y. Zhang, K. Li, S. Ren, Y. Dang, G. Liu, R. Zhang, K. Zhang, X. Long and K. Jia, Coal-derived graphene quantum dots produced by ultrasonic physical tailoring and their capacity for Cu(II) detection. *ACS Sustainable Chem. Eng.*, 2019, **7**, 9793–9799.
84. X. Liu, J. Han, X. Hou, F. Altincicek, N. Oncel, D. Pierce, X. Wu and J. X. Zhao, One-pot synthesis of graphene quantum dots using humic acid and its application for copper (II) ion detection. *J. Mater. Sci.*, 2021, **56**, 4991–5005.
85. D. Kurniawan, N. Sharma, M. R. Rahardja, Y. Y. Cheng, Y. T. Chen, G. X. Wu, Y. Y. Yeh, P. C. Yeh, K. K. Ostrikov and W. H. Chiang, Plasma nanoengineering of bioresource-derived graphene quantum dots as ultrasensitive environmental nanoprobes. *ACS Appl. Mater. Interfaces*, 2022, **14**, 52289–52300.
86. L. Lin, X. Song, Y. Chen, M. Rong, Y. Wang, L. Zhao, T. Zhao and X. Chen, Europium-decorated graphene quantum dots as a fluorescent probe for label-free, rapid and sensitive detection of Cu^{2+} and L-cysteine. *Anal. Chim. Acta*, 2015, **891**, 261–268.
87. B. Gao, D. Chen, B. Gu, T. Wang, Z. Wang, F. Xie, Y. Yang, Q. Guo and G. Wang, Facile and highly effective synthesis of nitrogen-doped graphene quantum dots as a fluorescent sensing probe for Cu^{2+} detection. *Curr. Appl. Phys.*, 2020, **20**, 538–544.
88. S. Jovanović, S. Dorontić, D. Jovanović, G. Ciasca, M. Budimir, A. Bonasera, M. Scopelliti, O. Marković and B. T. Marković, Gamma irradiation of graphene quantum dots with ethylenediamine: antioxidant for ion sensing. *Ceram. Int.*, 2020, **46**, 23611–23622.
89. X. Liu and B. Sun, One-pot synthesis of nitrogen-doped graphene quantum dots and their applications in bioimaging and detecting copper ions in living cells. *ACS Omega*, 2023, **8**, 27333–27343.

90. X. Sun, P. Liu, L. Wu and B. Liu, Graphene-quantum-dots-based ratiometric fluorescent probe for visual detection of copper ion. *Analyst*, 2015, **140**, 6742–6747.
91. W. Liu and X. Zhu, A novel fluorescence "turn off-on" sensor based on N-doped graphene quantum dots in amino acid ionic liquid medium and its application. *Talanta*, 2019, **197**, 59–67.
92. Y. Dong, W. Tian, S. Ren, R. Dai, Y. Chi and G. Chen, Graphene quantum dots/L-cysteine coreactant electrochemiluminescence system and its application in sensing lead(II) ions. *ACS Appl. Mater. Interfaces*, 2014, **6**, 1646–1651.
93. C. Zhou, W. Jiang and B. K. Via, Facile synthesis of soluble graphene quantum dots and its improved property in detecting heavy metal ions. *Colloids Surf. B*, 2014, **118**, 72–76.
94. Z. S. Qian, X. Y. Shan, L. J. Chai, J. R. Chen and H. Feng, A fluorescent nanosensor based on graphene quantum dots–aptamer probe and graphene oxide platform for detection of lead (II) ion. *Biosens. Bioelectron.*, 2015, **68**, 225–231.
95. S. Bian, C. Shen, H. Hua, L. Zhou, H. Zhu, F. Xi, J. Liu and X. Dong, One-pot synthesis of sulfur-doped graphene quantum dots as a novel fluorescent probe for highly selective and sensitive detection of lead(II). *RSC Adv.*, 2016, **6**, 69977–69983.
96. X. Niu, Y. Zhong, R. Chen, F. Wang, Y. Liu and D. Luo, A "turn-on" fluorescence sensor for Pb^{2+} detection based on graphene quantum dots and gold nanoparticles. *Sens. Actuators B*, 2018, **255**, 1577–1581.
97. Y. Xu, S. Wang, X. Hou, Z. Sun, Y. Jiang, Z. Dong, Q. Tao, J. Man and Y. Cao, Coal-derived nitrogen, phosphorus and sulfur co-doped graphene quantum dots: a promising ion fluorescent probe. *Appl. Surf. Sci.*, 2018, **445**, 519–526.
98. C. Kaewprom, P. Sricharoen, N. Limchoowong, P. Nuengmatcha and S. Chanthai, Resonance light scattering sensor of the metal complex nanoparticles using diethyl dithiocarbamate doped graphene quantum dots for highly Pb(II)-sensitive detection in water sample. *Spectrochim. Acta A*, 2019, **207**, 79–87.
99. N. Nandi, S. Gaurav, P. Sarkar, S. Kumar and K. Sahu, Hit multiple targets with one arrow: Pb^{2+} and ClO^{-} detection by edge functionalized graphene quantum dots and their applications in living cells. *ACS Appl. Bio Mater.*, 2021, **4**, 7605–7614.
100. Y. Chen, Y. Dong, H. Wu, C. Chen, Y. Chi and G. Chen, Electrochemiluminescence sensor for hexavalent chromium based on the graphene quantum dots/peroxodisulfate system. *Electrochim. Acta*, 2015, **151**, 552–557.
101. P. M. Carrasco, I. García, L. Yate, R. T. Zaera, G. Cabañero, H. J. Grande and V. Ruiz, Graphene quantum dot membranes as fluorescent sensing platforms for Cr (VI) detection. *Carbon*, 2016, **109**, 658–665.
102. L. Sheng, B. Huangfu, Q. Xu, W. Tian, Z. Li, A. Meng and S. Tan, A highly selective and sensitive fluorescent probe for detecting Cr(VI) and cell imaging based on nitrogen-doped graphene quantum dots. *J. Alloys Compd.*, 2020, **820**, 153191.
103. L. Qian, Z. Zhen, S. Tang, C. Zhou, M. Ji, B. Liu, Y. Fang, S. Ou and R. Cheng, A coordination-driven fluorescent platform for selective detection of valine in living cells and food samples based on dopamine-functionalized nitrogen doped graphene quantum dots and its construction of molecular logic gate. *Sens. Actuators B*, 2022, **367**, 132168.
104. H. Chen, W. Li, Q. Wang, X. Jin, Z. Nie and S. Yao, Nitrogen doped graphene quantum dots based single-luminophor generated dual-potential electrochemiluminescence system for ratiometric sensing of Co^{2+} ion. *Electrochim. Acta*, 2016, **214**, 94–102.

105. H. Huang, L. Liao, X. Xu, M. Zou, F. Liu and N. Li, The electron-transfer based interaction between transition metal ions and photoluminescent graphene quantum dots (GQDs): a platform for metal ion sensing. *Talanta*, 2013, **117**, 152–157.
106. Z. Fan, Y. Li, X. Li, L. Fan, S. Zhou, D. Fang, and S. Yang, Surrounding media sensitive photoluminescence of boron-doped graphene quantum dots for highly fluorescent dyed crystals, chemical sensing and bioimaging. *Carbon*, 2014, **70**, 149–156.
107. B. Y. Fang, C. Li, Y. Y. Song, F. Tan, Y. C. Cao and Y. D. Zhao, Nitrogen-doped graphene quantum dot for direct fluorescence detection of Al^{3+} in aqueous media and living cells. *Biosens. Bioelectron.*, 2018, **100**, 41–48.
108. S. Pathan, M. Jalal, S. Prasad and S. Bose, Aggregation-induced enhanced photoluminescence in magnetic graphene oxide quantum dots as a fluorescence probe for As(III) sensing. *J. Mater. Chem. A*, 2019, **7**, 8510–8520.
109. A. Suryawanshi, M. Biswal, D. Mhamane, R. Gokhale, S. Patil, D. Guin and S. Ogale, Large scale synthesis of graphene quantum dots (GQDs) from waste biomass and their use as an efficient and selective photoluminescence on–off–on probe for Ag^{+} ions. *Nanoscale*, 2014, **6**, 11664–11670.
110. R. Tabaraki and A. Nateghi, Nitrogen-doped graphene quantum dots: "turn-off" fluorescent probe for detection of Ag^{+} ions. *J. Fluoresc.*, 2016, **26**, 297–305.
111. S. Bian, C. Shen, Y. Qian, J. Liu, F. Xi and X. Dong, Facile synthesis of sulfur-doped graphene quantum dots as fluorescent sensing probes for Ag^{+} ions detection. *Sens. Actuators B*, 2017, **242**, 231–237.
112. X. E. Zhao, C. Lei, Y. Gao, H. Gao, S. Zhu, X. Yang, J. You and H. Wang, A ratiometric fluorescent nanosensor for the detection of silver ions using graphene quantum dots. *Sens. Actuators B*, 2017, **253**, 239–246.
113. P. Kaewanan, P. Sricharoen, N. Limchoowong, T. Sripakdee, P. Nuengmatch and S. Chanthai, A fluorescence switching sensor based on graphene quantum dots decorated with Hg^{2+} and hydrolyzed thioacetamide for highly Ag^{+}-sensitive and selective detection. *RSC Adv.*, 2017, **7**, 48058–48067.
114. M. Amjadi, R. Shokri and T. Hallaj, A new turn-off fluorescence probe based on graphene quantum dots for detection of Au(III) ion. *Spectrochim. Acta A*, 2016, **153**, 619–624.
115. S. Yang, J. Sun, C. Zhu, P. He, Z. Peng and G. Ding, Supramolecular recognition control of polyethylene glycol modified N-doped graphene quantum dots: tunable selectivity for alkali and alkaline-earth metal ions. *Analyst*, 2016, **141**, 1052–1059.
116. R. Wang, X. Du, Y. Wu, J. Zhai and X. Xie, Graphene quantum dots integrated in ionophore-based fluorescent nanosensors for Na^{+} and K^{+}. *ACS Sens.*, 2018, **3**, 2408–2414.
117. S. Zhuo, L. Chen, Y. Zhang and G. Jin, Luminescent phosphate sensor based on upconverting graphene quantum dots. *Spectrosc. Lett.*, 2016, **49**, 1–4.
118. X. Chu, S. Wang and Y. Cao, A new fluorescence probe comprising nitrogen-doped graphene quantum dots for the selective and quantitative determination of cerium(IV). *New J. Chem.*, 2020, **44**, 797–806.
119. J. Ding, X. Zhou, Y. Huang, B. Chen, S. Chen, Y. Jin, Y. Yang, N. Pan, C. Xu, J. Chen and C. Xia, An innovative strategy for construction of pH-responsive supramolecular hydrogel from graphene quantum dots clusters toward integration of detection and removal of uranium. *Appl. Surf. Sci.*, 2022, **583**, 152492.

120. S. L. Ting, S. J. Ee, A. Ananthanarayanan, K. C. Leong and P. Chen, Graphene quantum dots functionalized gold nanoparticles for sensitive electrochemical detection of heavy metal ions. *Electrochim. Acta*, 2015, **172**, 7–11.
121. J. Ou, Y. Tao, J. Ma and Y. Kong, Well-dispersed chitosan-graphene quantum dots nanocomposites for electrochemical sensing platform. *J. Electrochem. Soc.*, 2015, **162**, H884–H889.
122. J. Pizarro, R. Segura, D. Tapia, F. Navarro, F. Fuenzalida and M. J. Aguirre, Inexpensive and green electrochemical sensor for the determination of Cd (II) and Pb(II) by square wave anodic stripping voltammetry in bivalve molluscs. *Food Chem.*, 2020, **321**, 126682.
123. F. Xu, H. Shi, X. He, K. Wang, D. He, L. Yan, X. Ye, J. Tang, J. Shangguan and L. Luo, Masking agent-free and channel-switch-mode simultaneous sensing of Fe^{3+} and Hg^{2+} using dual-excitation graphene quantum dots. *Analyst*, 2015, **140**, 3925–3928.
124. S. K. Raj, A. Rajput, H. Gupta, R. Patidar and V. Kulshrestha, Selective recognition of Fe^{3+} and Cr^{3+} in aqueous medium via fluorescence quenching of graphene quantum dots. *J. Dispersion Sci. Technol.*, 2019, **40**, 250–255.
125. K. R. Savan, V. Yadav, G. R. Bhadu, R. Patidar, M. Kumar and V. Kulshrestha, Synthesis of highly fluorescent and water soluble graphene quantum dots for detection of heavy metal ions in aqueous media. *Environ. Sci. Pollut. Res.*, 2021, **28**, 46336–46342.
126. J. Li, Z. Wang, J. Yang, X. Xia, R. Yi, J. Jiang, W. Liu, J. Chen, L. Chen and J. Xu, "On-off-on" fluorescence switch of graphene quantum dots: a cationic control strategy. *Appl. Surf. Sci.*, 2021, **546**, 149110.
127. T. Anusuya, V. Kumar and V. Kumar, Hydrophilic graphene quantum dots as turn-off fluorescent nanoprobes for toxic heavy metal ions detection in aqueous media. *Chemosphere*, 2021, **282**, 131019.
128. Y. Yang, T. Zou, Z. Wang, X. Xing, S. Peng, R. Zhao, X. Zhang and Y. Wang, The fluorescent quenching mechanism of N and S co-doped graphene quantum dots with Fe^{3+} and Hg^{2+} ions and their application as a novel fluorescent sensor. *Nanomaterials*, 2019, **9**, 738.
129. E. Sharma, D. Vashisht, A. Vashisht, V. K. Vats, S. K. Mehta and K. Singh, Facile synthesis of sulfur and nitrogen codoped graphene quantum dots for optical sensing of Hg and Ag ions. *Chem. Phys. Lett.*, 2019, **730**, 436–444.
130. C. Kaewprom, Y. Areerob, W. C. Oh, K. L. Ameta and S. Chanthai, Simultaneous determination of Hg(II) and Cu(II) in water samples using fluorescence quenching sensor of N-doped and N,K co-doped graphene quantum dots. *Arab. J. Chem.*, 2020, **13**, 3714–3723.
131. L. Wang, W. Li, B. Wu, Z. Li, S. Wang, Y. Liu, D. Pan and M. Wu, Facile synthesis of fluorescent graphene quantum dots from coffee grounds for bioimaging and sensing. *Chem. Eng. J.*, 2016, **300**, 75–82.
132. P. Pashazadeh-Panahi, M. Hasanzadeh and R. Eivazzadeh-Keihan, A novel optical probe based on D-penicillamine-functionalized graphene quantum dots: Preparation and application as signal amplification element to minoring of ions in human biofluid. *J. Mol. Recognit.*, 2020, **33**, e2828.
133. H. Ahmadi, S. Keshipour and F. Ahour, New water-soluble colorimetric pH and metal ione sensor based on graphene quantum dot modified with alizarine red S. *Sci. Rep.*, 2020, **10**, 14185.

134. L. Vahab and S. Keshipour, Novel nanosensor of cobalt(II) and copper(II) constructed from graphene quantum dots modified with eriochrome black T. *Sci. Rep.*, 2022, **12**, 13179.
135. J. M. Bai, L. Zhang, R. P. Liang and J. D. Qiu, Graphene quantum dots combined with europium ions as photoluminescent probes for phosphate sensing. *Chem. Eur. J.*, 2013, **19**, 3822–3826.
136. B. B. Chen, R. S. Li, M. L. Liu, H. Y. Zou, H. Liu and C. Z. Huang, Highly selective detection of phosphate ion based on a single-layered graphene quantum dots-Al^{3+} strategy. *Talanta*, 2018, **178**, 172–177.
137. S. Q. Chai, J. H. He, L. Zhan, Y. F. Li, C. M. Li and C. Z. Huang, Dy(III)-induced aggregation emission quenching effect of single-layered graphene quantum dots for selective detection of phosphate in the artificial wetlands. *Talanta*, 2019, **196**, 100–108.
138. Y. Wang, W. Weng, H. Xu, Y. Luo, D. Guo, D. Li and D. Li, Negatively charged molybdate mediated nitrogen-doped graphene quantum dots as a fluorescence turn on probe for phosphate ion in aqueous media and living cells. *Anal. Chim. Acta*, 2019, **1080**, 196–205.
139. L. Lin, X. Song, Y. Chen, M. Rong, T. Zhao, Y. Jiang, Y. Wang and X. Chen, One-pot synthesis of highly greenish-yellow fluorescent nitrogen-doped graphene quantum dots for pyrophosphate sensing via competitive coordination with Eu^{3+} ions. *Nanoscale*, 2015, **7**, 15427–15433.
140. T. Hallaj, M. Amjadi, J. L. Manzoori and R. Shokri, Chemiluminescence reaction of glucose-derived graphene quantum dots with hypochlorite, and its application to the determination of free chlorine. *Microchim. Acta*, 2015, **182**, 789–796.
141. L. Ma, S. Sun, Y. Wang, K. Jiang, J. Zhu, J. Li and H. Lin, A graphene quantum dot-based fluorescent nanoprobe for hypochlorite detection in water and in living cells. *Microchim. Acta*, 2017, **184**, 3833–3840.
142. S. B. Sefidan, H. Eskandari and A. N. Shamkhali, Rapid colorimetric flow injection sensing of hypochlorite by functionalized graphene quantum dots. *Sens. Actuators B*, 2018, **275**, 339–349.
143. E. Sharma, D. Vashisht, V. Thakur, A. Vashisht, S. K. Mehta and K. Singh, Graphene quantum dots functionalized with bovine serum albumin for sensing of hypochlorite ions. *Mater. Chem. Phys.*, 2021, **273**, 125088.
144. N. Yu, H. Peng, H. Xiong, X. Wu, X. Wang, Y. Li and L. Chen, Graphene quantum dots combined with copper(II) ions as a fluorescent probe for turn-on detection of sulfide ions. *Microchim. Acta*, 2015, **182**, 2139–2146.
145. W. Na, Z. Qu, X. Chen and X. Su, A turn-on fluorescent probe for sensitive detection of sulfide anions and ascorbic acid by using sulfanilic acid and glutathione functionalized graphene quantum dots. *Sens. Actuators B*, 2018, **256**, 48–54.
146. L. Wang, J. Zheng, S. Yang, C. Wu, C. Liu, Y. Xiao, Y. Li, Z. Qing and R. Yang, Two-photon sensing and imaging of endogenous biological cyanide in plant tissues using graphene quantum dot/gold nanoparticle conjugate. *ACS Appl. Mater. Interfaces*, 2015, **7**, 19509–19515.
147. C. Chen, D. Zhao, T. Hu, J. Sun and X. Yang, Highly fluorescent nitrogen and sulfur co-doped graphene quantum dots for an inner filter effect-based cyanide sensor. *Sens. Actuators B*, 2017, **241**, 779–788.
148. N. Kongsanan, N. Pimsin, C. Keawprom, P. Sricharoen, Y. Areerob, P. Nuengmatcha, W. C. Oh, S. Chanthai and N. Limchoowong, A fluorescence

switching sensor for sensitive and selective detections of cyanide and ferricyanide using mercuric cation-graphene quantum dots. *ACS Omega*, 2021, **6**, 14379–14393.
149. L. Li, D. Liu, K. Wang, H. Mao and T. You, Quantitative detection of nitrite with N-doped graphene quantum dots decorated N-doped carbon nanofibers composite-based electrochemical sensor. *Sens. Actuators B*, 2017, **252**, 17–23.
150. W. Wang, S. Xu, N. Li, Z. Huang, B. Su and X. Chen, Sulfur and phosphorus co-doped graphene quantum dots for fluorescent monitoring of nitrite in pickles. *Spectrochim. Acta A*, 2019, **221**, 117211.
151. B. Zhang, Y. He and Z. Fan, Nitrogen-doped graphene quantum dots as highly sensitive and selective fluorescence sensor detection of iodide ions in milk powder. *J. Photochem. Photobiol. A*, 2018, **367**, 452–457.
152. F. Askari, A. Rahdar, M. Dashti and J. F. Trant, Detecting mercury (II) and thiocyanate using "turn-on" fluorescence of graphene quantum dots. *J. Fluoresc.*, 2020, **30**, 1181–1187.
153. M. Roushani and Z. Abdi, Novel electrochemical sensor based on graphene quantum dots/riboflavin nanocomposite for the detection of persulfate. *Sens. Actuators B*, 2014, **201**, 503–510.
154. S. Chen, Y. Song, Y. Li, Y. Liu, X. Su and Q. Ma, A facile photoluminescence modulated nanosensor based on nitrogen-doped graphene quantum dots for sulfite detection. *New J. Chem.*, 2015, **39**, 8114–8120.
155. X. Sun, Y. Qian, Y. Jiao, J. Liu, F. Xi and X. Dong, Ionic liquid-capped graphene quantum dots as label-free fluorescent probe for direct detection of ferricyanide. *Talanta*, 2017, **165**, 429–435.
